# How Pol α-primase is targeted to replisomes to prime eukaryotic DNA replication

**DOI:** 10.1016/j.molcel.2023.06.035

**Published:** 2023-08-17

**Authors:** Morgan L. Jones, Valentina Aria, Yasemin Baris, Joseph T.P. Yeeles

**Affiliations:** 1 MRC Laboratory of Molecular Biology, Cambridge CB2 0QH, UK

**Keywords:** DNA replication, replisome, priming, Pol α-primase, genome stability, helicase, DNA polymerase, CMG, cryo-EM

## Abstract

During eukaryotic DNA replication, Pol α-primase generates primers at replication origins to start leading-strand synthesis and every few hundred nucleotides during discontinuous lagging-strand replication. How Pol α-primase is targeted to replication forks to prime DNA synthesis is not fully understood. Here, by determining cryoelectron microscopy (cryo-EM) structures of budding yeast and human replisomes containing Pol α-primase, we reveal a conserved mechanism for the coordination of priming by the replisome. Pol α-primase binds directly to the leading edge of the CMG (CDC45-MCM-GINS) replicative helicase via a complex interaction network. The non-catalytic PRIM2/Pri2 subunit forms two interfaces with CMG that are critical for *in vitro* DNA replication and yeast cell growth. These interactions position the primase catalytic subunit PRIM1/Pri1 directly above the exit channel for lagging-strand template single-stranded DNA (ssDNA), revealing why priming occurs efficiently only on the lagging-strand template and elucidating a mechanism for Pol α-primase to overcome competition from RPA to initiate primer synthesis.

## Highlights

• Structures of DNA engaged yeast and human replisomes containing Pol α-primase
• A conserved mechanism targets Pol α-primase to the replisome for priming
• A multisite interaction network targets primase to the lagging-strand template
• Direct interactions between CMG and Pol α-primase are critical for priming

## Graphical abstract

**Figure:**
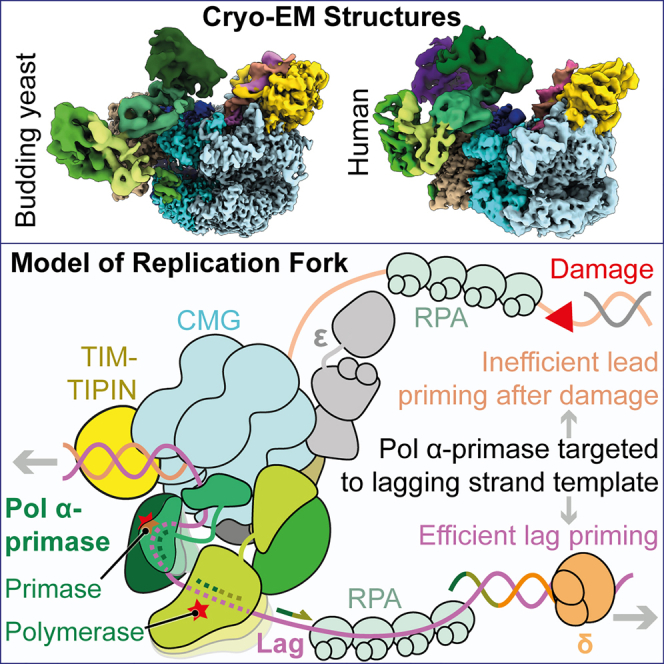

## Teaser

By determining cryo-EM structures of budding yeast and human replisomes containing the Pol α-primase complex, Jones et al. reveal a conserved mechanism for the coordination of nascent-strand priming in the eukaryotic replisome. The mechanism explains why priming by Pol α-primase is highly efficient on the lagging-strand but not leading-strand template.

## Introduction

Following replisome assembly and template unwinding at bi-directional origins of DNA replication, Pol α-primase is recruited to the two advancing replisomes where it primes the lagging-strand template.^1^ To start coupled leading-strand replication, these primers are extended across the origin by the main lagging-strand polymerase, Pol δ,^2^ before a polymerase switch transfers the nascent strand to the principal leading-strand polymerase, Pol ε.^1^^,^^3^^,^^4^^,^^5^^,^^6^ As replication forks progress primers are synthesized every few hundred nucleotides to support discontinuous lagging-strand replication. If leading-strand synthesis is interrupted due to DNA damage, biochemical reconstitution experiments have demonstrated that *S. cerevisiae* (budding yeast) replisomes continue lagging-strand replication but do not frequently reinitiate leading-strand replication due to a failure to support efficient primer synthesis on this strand.^7^^,^^8^^,^^9^^,^^10^ Collectively, these observations indicate that the replisome efficiently targets Pol α-primase to the lagging-strand template but not the leading-strand template, which likely explains why some eukaryotes, including humans, encode a second primase-polymerase, PRIMPOL, to restart leading-strand replication.^11^^,^^12^^,^^13^^,^^14^ Currently the mechanistic basis underlying the preference of Pol α-primase for lagging- rather than leading-strand priming is unknown.

**Figure 1:**
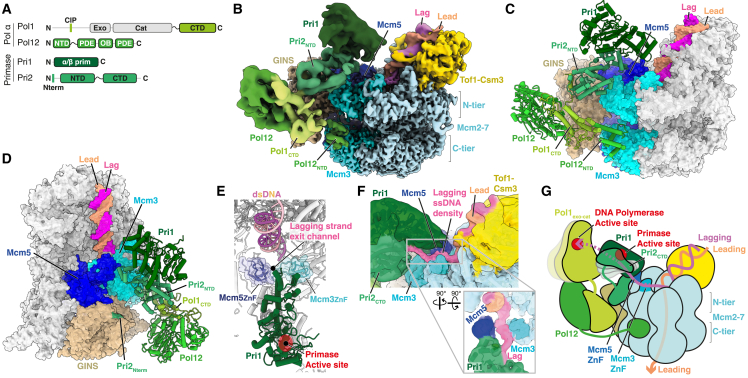
Structure of Pol α-primase in the budding yeast replisome (A) Domain architecture of yeast Pol α-primase. exo, exonuclease domain; cat, catalytic domain; CIP, Ctf4-interacting peptide; NTD, N-terminal domain; CTD, C-terminal domain; PDE, phosphodiesterase domain; OB, oligonucleotide/oligosaccharide-binding domain. (B) Composite cryo-EM map of the budding yeast Pol α-primase associated replisome bound to replication fork DNA containing a 60 nucleotide 5′ flap. Density for Ctf4 is not observed in this map. The map was derived from combining individual focused refinements and is colored according to chain occupancy. (C and D) Atomic model of the budding yeast Pol α-primase associated replisome lacking Ctf4 derived from cryo-EM data displayed in (B). Regions of CMG that physically interact with Pol α-primase are colored. (E) Focused view of the Pri1 catalytic subunit of primase, showing how it is positioned above the exit channel for lagging-strand template ssDNA. (F) Cryo-EM reconstruction displaying continuous density for lagging-strand template ssDNA extending from the point of dsDNA strand separation toward the active site region of Pri1. Map colored by chain occupancy with the density assigned to the lagging-strand template post-strand separation colored manually. (G) Schematic illustrating the organization of Pol α-primase in the budding yeast replisome. The path of lagging-strand template ssDNA visualized in the structure immediately following strand separation is illustrated (solid pink line). The putative path of the lagging-strand template between the Pri1 and Pol1 active sites is also illustrated (dashed pink line).

Pol α-primase is a constitutive heterotetramer composed of a dimeric primase (PRIM1 and PRIM2 in *H. sapiens* [human], Pri1 and Pri2 in budding yeast) and a dimeric DNA polymerase (POLA1 and POLA2 in human, Pol1 and Pol12 in budding yeast) (Figure 1A). Primase synthesizes 8–10 nucleotides (nt) of RNA that are transferred to the Pol α DNA polymerase for limited extension to a total primer length about 20–35 nt.^15^^,^^16^^,^^17^^,^^18^ *In vitro*, the ability of budding yeast^7^ and human^19^^,^^20^^,^^21^ Pol α-primase to initiate primer synthesis on single-stranded DNA (ssDNA) templates is blocked when the template is saturated with RPA, indicating that a mechanism exists to target primase to ssDNA at the eukaryotic replication fork. Consistent with this idea, Pol α-primase interacts with several core components of the replisome including AND-1 (Ctf4 in budding yeast), MCM10, GINS, and CMG.^22^^,^^23^^,^^24^^,^^25^^,^^26^^,^^27^

In budding yeast, Pol α-primase is tethered to replisome progression complexes (RPCs) via interaction between its Pol1 subunit and Ctf4, a trimeric scaffold protein that binds directly to CMG.^28^^,^^29^^,^^30^^,^^31^ Similarly, human Pol α-primase associates with AND-1 but does so primarily via the N-terminal domain (NTD) of POLA2, which binds a C-terminal HMG-box in AND-1.^32^^,^^33^ However, considerable evidence indicates that AND-1/Ctf4 does not provide a pivotal link between Pol α-primase and the replisome to support priming: Ctf4 is a non-essential protein in budding yeast; disruption of the Pol1:Ctf4 interaction does not result in obvious DNA replication defects *in vivo*^34^; Pol α-primase localizes to replisomes in yeast cells lacking Ctf4^35^; depletion of AND-1 in DT40 cells does not prevent the completion of bulk replication^36^; AND-1 and Ctf4 are dispensable for lagging-strand synthesis in DNA replication reactions reconstituted with purified proteins.^27^^,^^37^^,^^38^^,^^39^ Similarly, reconstituted budding yeast and human replisomes perform lagging-strand replication in the absence of MCM10.^27^^,^^39^^,^^40^

Accumulating evidence suggests that Pol α-primase is recruited to the replisome for priming via direct interactions with CMG. Yeast Pol α-primase can execute lagging-strand replication when functioning only with CMG and RPA.^39^ We recently found that minimal human replisomes consisting of CMG, Pol α-primase, Pol ε, CTF18-RFC, PCNA, and RPA support lagging-strand replication and that human Pol α-primase comigrates with CMG in glycerol gradient sedimentation experiments.^27^ However, because there are no structures of Pol α-primase in the eukaryotic replisome, we do not know how Pol α-primase binds to CMG, how these putative interactions contribute to lagging-strand DNA replication, how Pol α-primase might overcome competition with RPA for exposed ssDNA, where in the replisome Pol α-primase is positioned, and why Pol α-primase efficiently primes the lagging-strand but not leading-strand template. To address these questions, we have determined cryoelectron microscopy (cryo-EM) structures of budding yeast and human replisomes bound to Pol α-primase and replication fork DNA.

## Results

### Yeast Pol α-primase replisome structure

To assemble budding yeast replisomes associated with Pol α-primase for cryo-EM analysis, CMG was bound to a model replication fork containing a 39 nt 3′ flap, onto which CMG loads, and a 60 nt 5′ ssDNA flap to mimic the unwound lagging-strand template (Figure S1A). Fork-bound CMG was incubated with Pol α-primase and the replisome accessory factors Mrc1 and Tof1-Csm3. Tof1-Csm3 binds to the leading edge of CMG where it engages and stabilizes the parental DNA duplex and fork junction,^30^ which we reasoned might be important to aid the visualization of template DNA in cryo-EM reconstructions. After glycerol gradient sedimentation, complexes were isolated containing all replisome and Pol α-primase subunits and used to prepare grids for cryo-EM data collection and analysis (Figures S1 and S2; Table 1).

**Table 1:** Cryo-EM statistics

	Budding yeast	Budding yeast	Budding yeast	Human	Human
	replisome:Pol α-primase	replisome:Pol α-primase	replisome:Pol α-primase	replisome:Pol α-primase	replisome:Pol α-primase
	60 nt 5′ flap (−) Ctf4	60 nt 5′ flap (+) Ctf4. CIP #1	60 nt 5′ flap (+) Ctf4. CIP #2	60 nt 5′ flap	15 nt 5′ flap
	(EMDB-16322)	(EMDB-15902)	(EMDB-15902)	(EMDB-15341)	(EMD-15922)
	(PDB: 8B9C)	(PDB: 8B9A)	(PDB: 8B9B)	(PDB: 8B9D)	–

**Data collection and processing**					

Magnification	81,000 ×	81,000×	81,000×	81,000×	81,000×
Voltage (kV)	300	300	300	300	300
Electron exposure (e^−^/Å^2^)	40.184	40.184	40.184	37.8	88.5
Defocus range (μm)	1.5–3	1.5–3	1.5–3	1.5–3	3.5–0.9
Pixel size (Å) (super resoution)	0.86	0.86	0.86	1.23	1.07
Symetry imposed	none	none	none	none	none
Movies collected	12,819	12,819	12,819	7,355	6,718
Initial particle images (no.)	2,003,322	2,003,322	2,003,322	1,535,548	724,557
Final particle images (no.)	100,179	54,970	44,970	174,696	258,339
Map resolution (Å) (0.143 FSC threshold)	3.34	3.5	3.5	3.4	3.3
Map resolution range (Å)	2.8–12	2.9–12	2.9–12	2.8–12	3.0–12

**Refinement**					

Initial model used (PDB code)	PDB: 6SKL	PDB: 6SKL	PDB: 6SKL	PDB: 7PFO	–
Model resolution (Å) (0.5 FSC threshold)	4.1	4.2	4.2	4.1	–
Map sharpening *B* factor (Å^2^)	−20 to −50	−20 to −50	−20 to −50	−20 to −50	–

**Model composition**					

Non-hydrogen atoms	59,119	69,886	69,866	66,207	–
Protein residues	7,193	8,528	8,528	8,108	–
Ligands	4 AMP-PNP	4 AMP-PNP	4 AMP-PNP	3 AMP-PNP	–
	4 Mg^2+^, 4 Zn^2+^	4 Mg^2+^, 4 Zn^2+^	4 Mg^2+^, 4 Zn^2+^	3 Mg^2+^, 4 Zn^2+^	–

**RMSDs**					

Bond lengths (Å)	0.023	0.011	0.01	0.027	–
Bond angles (°)	1.913	1.396	1.101	2.66	–

**Validation**					

MolProbity score	0.73	0.75	0.77	0.78	–
Clashscore	0.32	0.62	0.69	0.32	–
Poor rotamers (%)	0.29	0.5	0.53	0.66	–

**Ramachandran plot**					

Favored (%)	97.42	97.81	97.81	97.12	–
Allowed (%)	2.58	2.19	2.19	2.88	–
Disallowed (%)	0	0	0	0	–

Three-dimensional (3D) reconstructions revealed well resolved cryo-EM density for CMG and Tof1-Csm3. Multiple additional densities were also apparent extending from the N-tier face of CMG atop Mcm3 and Mcm5 toward the MCM C-tier beside Mcm3 and GINS (Figure 1B). The resolution of these densities was typically lower than for CMG and displayed considerable variability (Figure S1E), indicating large conformational flexibility. Nonetheless, following extensive focused classification and refinement (see Figure S2), these densities could be unambiguously attributed to Pol α-primase (Figures 1B, S1E–S1O, and S3B–S3D), enabling us to construct a model of a DNA engaged yeast replisome encompassing CMG, Tof1-Csm3, several small sections of Mrc1 (Figure S3A) and regions of all four Pol α-primase subunits (Figures 1C and 1D; Video S1). The Pol α-primase model comprises the primase catalytic subunit Pri1 aside from its flexible N and C termini, the N terminus (residues 1–5) and NTD (residues 44–177 and 181–299) of the primase accessory subunit Pri2, the C-terminal domain (CTD) of the Pol α catalytic subunit Pol1 (Pol1_CTD_) (residues 1,271–1,468) and the majority of the Pol α accessory subunit Pol12 (residues 1–79, 203–582, and 604–705) (Figures 1C, 1D, and S3B–S3D).

Video S1. Overview of the structure of pol α-primase in the budding yeast replisome, related to Figure 1 First, individual replisome components are sequentially colored and labeled to aid in their visualisation and the interpretation of the overall structure. The video then highlights each pol α-primase interface with the replisome (sites a-e) using different views. Regions of the model forming an interaction interface are displayed using transparent surface rendering.

Pri1 is positioned close to the incoming parental double-stranded DNA (dsDNA) above a channel between the Mcm3 and Mcm5 zinc-finger (ZnF) domains, through which lagging-strand template ssDNA is extruded after strand separation (Figure 1E).^41^^,^^42^^,^^43^^,^^44^ It is localized to the replisome through its interaction with the Pri2 NTD (Pri2_NTD_), which sits on the periphery of the MCM N-tier straddling Mcm3 and Mcm5 (Figures 1C and 1D). Pol α (Pol1_CTD_ and Pol12) is situated between the CMG N- and C-tiers, close to Mcm3 and GINS (Figures 1C and 1D) and is coupled to primase via an interaction between Pol1_CTD_ and Pri2_NTD_.^45^ Pol12 interacts extensively with Pol1_CTD_ and is anchored to the MCM C-tier through an interface involving its flexibly tethered NTD and Mcm3 (Figure 1C). Although Ctf4 was not included in replisome reconstitutions, we identified a 3D class containing both Pol α-primase and Ctf4 (Figure S3E). The presence of Ctf4 likely resulted from endogenous Ctf4 co-purifying with CMG due to the extensive interface between the two complexes.^30^^,^^31^ Comparison of the structures with and without Ctf4 revealed no substantial changes in the conformation of Pol α-primase (Figures S3E and S3F).

Clear densities for the Pol1 exonuclease-catalytic (exo-cat) domain (Pol1_exo-cat_) and Pri2 CTD (Pri2_CTD_) were not observed in our consensus refinement (Figures 1B and S1E), indicating that neither domain adopts a single stable conformation when Pol α-primase is bound to the replisome. This behavior contrasts with the crystal structure of human apo Pol α-primase, where both domains were well ordered.^46^ However, we recovered several rare 3D classes with low-resolution densities of the appropriate shape and volume to accommodate Pol1_exo-cat_ and Pri2_CTD_, although the precise orientation of each domain could not be assigned (Figures S3G and S3H). In these reconstructions, Pri2_CTD_ is adjacent to Pri1 close to the primase active site, while Pol1_exo-cat_ sits above Pri2_NTD_ on the periphery of the replisome adjacent to the Pri2_CTD_. This configuration more closely resembles the architecture of human Pol α-primase bound to CST (CTC1-STN1-TEN1) and telomeric ssDNA^47^ (Figure S3I), and a very recent structure of a human Pol α-primase elongation complex,^48^ than the human Pol α-primase apo structure.^46^ This led us to consider that Pol α-primase conformation in the yeast replisome might be modulated by protein-protein interactions and/or DNA engagement. Because DNA binding was heterogeneous across the dataset, we obtained a 3D replisome reconstruction lacking DNA (Figure S3J). Here, the positioning of Pol1_exo-cat_ and Pri2_CTD_ resembled the human apo crystal structure^46^ (Figure S3K), indicating that Pol α-primase undergoes DNA-dependent conformational changes when associated with CMG in the budding yeast replisome.

To further explore the putative DNA engagement state of Pol α-primase, we performed additional classification focusing on regions of primase close to the replication fork junction (Figure S2). Strikingly, this strategy revealed a 3D class with continuous density extending from the parental DNA duplex at the point of strand separation, through the channel between the Mcm3 and Mcm5 ZnF domains and alongside Pri1 in the direction of the primase active site (Figure 1F). The density between the Mcm3 and Mcm5 ZnF domains is in an equivalent position to the previously identified path of the lagging-strand template following strand separation in the human replisome,^41^^,^^42^ strongly suggesting that it corresponds to lagging-strand template ssDNA. Moreover, the close proximity of the density to Pri1 and its continuation beyond the Mcm3-Mcm5 ZnF channel—which has not been observed in prior human and yeast replisome structures lacking Pol α-primase—indicate that Pri1 engages lagging-strand template ssDNA in the yeast replisome structure. We hypothesize that this configuration functions to ensure a minimal length of ssDNA is required for the lagging-strand template to reach the primase active site, thereby enabling primase to outcompete RPA for access to the template to initiate primer synthesis. Moreover, the positioning of Pri1 and Pol1_exo-cat_ arranges the primase and DNA polymerase catalytic centers in synthesis order along the template (Figures 1G and S3L), suggesting a possible mechanism for transfer of the RNA primer to the Pol α DNA polymerase as the replisome advances, similar to the mechanism proposed for human Pol α-primase during telomere C strand fill-in.^47^

### Pol α-primase replisome interactions

**Figure 2:**
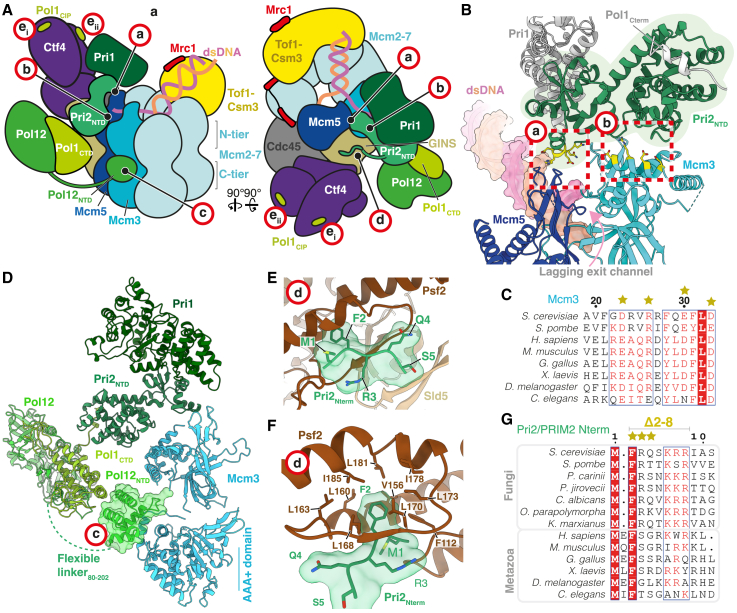
The structural basis for Pol α-primase recruitment to the budding yeast replisome (A) Schematic of the budding yeast replisome highlighting Pol α-primase-binding sites (red circles labeled a–e). (B) Atomic model highlighting the interfaces between Pri2_NTD_ (green) and the Mcm5 (blue) zinc finger (site a) and Mcm3 (cyan) N-terminal helical domain (site b). Residues colored yellow with side chains displayed represent those targeted for mutational analysis. (C) Multiple sequence alignment indicating the conservation of Mcm3 residues contacting Pri2_NTD_ (site b), colored according to conservation. Stars correspond to the Mcm3 residues colored yellow in (B) that were mutated. (D) Atomic model highlighting the interface between the Pol12_NTD_ (green) and the Mcm3 (cyan) AAA+ domain in the MCM C-tier (site c). (E) Atomic model highlighting the interface between the Pri2_Nterm_ (green) and the Psf2 subunit of GINS (brown) (site d). (F) Atomic model showing how Pri2-F2 projects into a hydrophobic pocket on Psf2, colored as in (E). (G) Multiple sequence alignment of Pri2_Nterm_ residues contacting Psf2. The alignment is grouped into fungal and metazoan sequences and colored according to conservation. Stars indicate residues mutated to alanine in the Pri2-AAA mutant.

Four small interaction sites, labeled sites a–d in Figure 2A, tether Pol α-primase directly to CMG and position primase to engage lagging-strand template ssDNA (Video S1). Pri2_NTD_ forms electrostatic interfaces with both the Mcm5 ZnF domain (site a) and the Mcm3 N-terminal helical domain (site b) (Figure 2B). The interface with the Mcm5 ZnF is mediated by a small insertion in Pri2_NTD_ that appears confined to a subset of fungal species (Figure S4A), while the interface with Mcm3 involves three flexible loops within the Pri2_NTD_ (between helices α3-4, α4-5, an α6-7) that are positioned to interact with conserved surface-exposed charged residues on the first alpha helix (α1) of Mcm3 (Figures 2B, 2C, and S4B). 3D variability analysis^49^ shows that Pri2_NTD_ adopts a continuum of rotational states with respect to Mcm3 while remaining engaged, likely due to the electrostatic nature of the interface (Figure S4C).

The remaining two interfaces between Pol α-primase and CMG (sites c and d) involve regions of the Pri2 and Pol12 subunits situated at the ends of regions of polypeptide predicted to be unstructured,^50^ indicating that they form flexible tethering points (Figures 1A, 1D, 2A, and 2D–2F). The 79 amino acid (aa) Pol12 NTD (Pol12_NTD_) adopts a compact helical fold connected to the phosphodiesterase (PDE) domain via a 130 aa unstructured linker region. Pol12_NTD_ binds the Mcm3 AAA+ domain in the MCM C-tier (site c), where helices α8 and α18 of Mcm3 form an electrostatic cradle into which α4 of the Pol12_NTD_ docks (Figures 2D and S4D). During ATP-dependent DNA translocation the MCM C-tier adopts multiple conformational states dependent on nucleotide occupancy and DNA engagement.^30^^,^^44^ While we observe only one C-tier conformation when Pol12_NTD_ is bound to Mcm3, the Pol12_NTD_ can be docked without clashes onto Mcm3 via the same interface through a range of C-tier configurations, indicating it might remain associated with Mcm3 throughout active replication (Figure S4E). Site d involves the N-terminal 5 amino acids of Pri2 (Pri2_Nterm_), where Pri2-F2—invariant in fungal species—docks into a surface-exposed hydrophobic pocket on the GINS subunit Psf2 (Figures 2E–2G). Pri2_Nterm_ is connected to Pri2_NTD_ via a 40 aa linker and, although this linker is predicted to be unstructured, at low map thresholds, continuous density is visible between the last modeled residue of Pri2_Nterm_ (S5) and the first modeled residue of the Pri2_NTD_ (S44), indicating that a section of the linker might adopt a structured conformation (Figure S4F).

In reconstructions containing Ctf4, local refinement revealed the presence of Pol α-primase-dependent density on the surface of the C-terminal α-helical domains of Ctf4 at the previously identified Pol1 binding site,^29^ into which the Pol1 Ctf4-interacting peptide (CIP box) (Pol1_CIP_) can be docked (PDB: 4C93) (Figures 2A, S4G, and S4H). Although we see no evidence for the presence of multiple copies of Pol α-primase in our dataset, density for Pol1_CIP_ was observed on two Ctf4 monomers (the third monomer is bound to the Sld5 CIP box^30^^,^^31^), suggesting mixed occupancy within our reconstructions. Thus, Pol1_CIP_ can bind to either Ctf4 monomer while Pol α-primase is associated with CMG (labeled sites e_i_ and e_ii_ in Figure 2A), presumably because the CIP box is linked to Pol1_exo_ via ∼200 aa of largely unstructured polypeptide.

Although we obtained 3D reconstructions where Pol α-primase was bound to CMG at all 4 sites, a substantial fraction of the dataset lacking Ctf4 displayed binding at just the Mcm5 ZnF and GINS interfaces (sites a and d), demonstrating that only a subset of binding sites are necessary to anchor Pol α-primase to CMG (Figures S4I–S4K). Inspection of the cryo-EM density in reconstructions where only sites a and d were engaged reveals that Pri2_NTD_ and Pol1_CTD_-Pol12 are less well resolved, indicating that these regions are stabilized by the binding of Pri2_NTD_ and Pol12_NTD_ to Mcm3 (sites b and c, respectively). These data indicate that Pol α-primase can utilize only a subset of interaction sites for replisome association, which might be important to permit conformational changes during the priming cycle.^18^^,^^46^

### Pol α-primase interaction mutants

**Figure 3:**
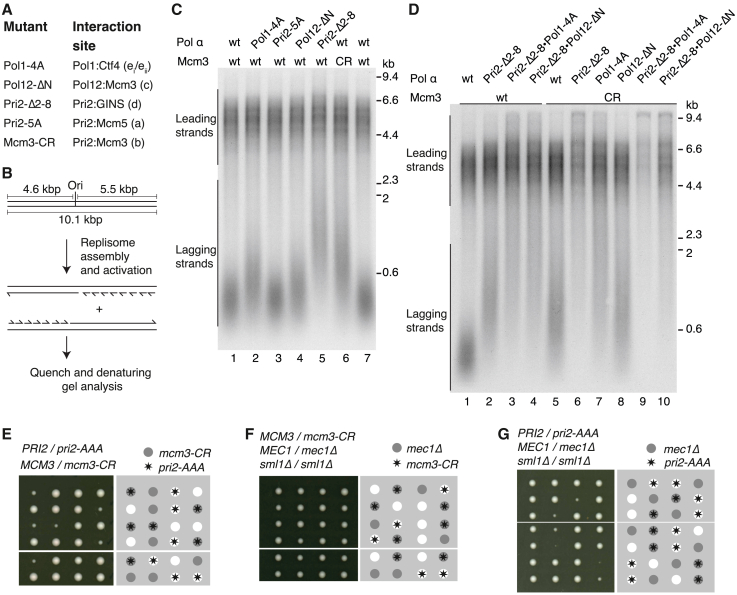
Pol α-primase CMG binding sites are critical for DNA replication (A) Summary of Pol α-primase and Cdt1-Mcm2-7 mutants and the interaction sites that are targeted. CR, charge reversal. (B) Schematic of the DNA template and anticipated products for origin-dependent budding yeast *in vitro* DNA replication reactions. (C and D) Denaturing agarose gel analysis of origin-dependent DNA replication reactions performed as illustrated in (B) for 20 min. (E–G) Diploid budding yeast cells of the indicated genotype were sporulated and the resulting tetrads were dissected and grown on YPD medium for 3 days at 25°C. Dissections that displayed abnormal segregation patterns were cropped from plate images.

To examine the contributions of the Pol α-primase:CMG interfaces during DNA replication, we purified Pol α-primase mutants and truncations designed to disrupt the Pri2:Mcm5 (Pri2-5A), Pol12:Mcm3 (Pol12-ΔN) and Pri2:GINS (Pri2-Δ2-8) interfaces (sites a, c, and d, respectively) and a Cdt1-Mcm2-7 charge reversal (CR) mutant (Mcm3-CR) designed to disrupt the Pri2_NTD_:Mcm3 binding site (site b) (Figures 3A and S5A). We also purified a Pol α-primase complex in which the Pol1 CIP box was mutated to abrogate its interaction with Ctf4^29^ (Pol1-4A) (targeting sites e_i_ and e_ii_) (Figures 3A and S5A). Figure S5B shows that all Pol α-primase mutants displayed similar priming and DNA synthesis activities to the wild-type protein on ssDNA templates. Origin-dependent DNA replication reactions that generate leading- and lagging-strand products were reconstituted with purified budding yeast proteins on a 10.1 kbp linear DNA template with the origin positioned roughly at its center (Figure 3B).^1^^,^^37^^,^^38^ In reconstituted replication reactions in which the lagging-strand maturation machinery is omitted, the length distribution of lagging-strand products is dependent on Pol α-primase concentration, with less frequent priming resulting in the synthesis of longer lagging strands.^37^^,^^39^^,^^51^

Replication with wild-type proteins produced a population of ∼4.5–6.5 kb leading strands and lagging strands of less than 0.6 kb (Figure 3C, lanes 1 and 7). Figure 3C shows that all mutant proteins with a single binding site targeted were competent for leading and lagging-strand DNA replication, demonstrating that no single interface is essential for Pol α-primase function. However, the distribution of lagging-strand products varied considerably among mutants, indicating that each interface does not contribute equally to productive primer synthesis. Surprisingly, mutations designed to target the Pri2:Mcm5 interface (Pri2-5A, site a) did not affect the length of lagging-strand products (Figure 3C, lane 3). Disruption of the Pol12:Mcm3 (Pol12-ΔN, site c) and Pol1:Ctf4 (Pol1-4A, sites e_i_/e_ii_) binding sites resulted in slightly longer lagging-strand products than reactions containing wild-type Pol α-primase under these conditions (Figures 3C, lanes 1, 2, and 4 and S5C), indicating that these interfaces make relatively minor contributions to primer synthesis in the replisome. Strikingly, there was a marked lengthening of lagging-strand products when the Pri2:Mcm3 (Mcm3-CR, site b) and Pri2:GINS (Pri2-Δ2-8, site d) interfaces were perturbed (Figure 3C, lanes 5 and 6). Loss of the Pri2:GINS interaction had the most pronounced effect, with lagging-strand products displaying a broad length distribution of between 0.6 and 2 kb (Figure 3C, lane 5). These data indicate that the Pri2:GINS (site d) and Pri2:Mcm3 (site b) interfaces are the most important interaction sites for priming in the budding yeast replisome.

To gain further insight into the hierarchy of Pol α-primase:replisome interactions during DNA replication, we purified additional complexes harboring combinations of mutations/truncations (Figures S5A and S5B). Figures 3D, S5C, and S5D show that, in almost all cases, disrupting multiple Pol α-primase binding sites resulted in further increases in the length of lagging-strand products. Notably, lagging-strand synthesis was all but abolished in a reaction where both the Pri2:Mcm3 and Pri2:GINS interfaces (sites b and d) were disrupted, and there was a reduction in intensity and subtle lengthening of leading-strand products (Figure 3D, lane 6), which is indicative of delayed synthesis of the primers used to start leading-strand replication.^1^ Leading-strand replication was further compromised in reactions where the Pri2:Mcm3, Pri2:GINS, and Pol1:Ctf4 interfaces (sites b, d, and e_i_/e_ii_) were targeted simultaneously (Figures 3D, lane 9 and S5D, lane 7). Similar defects were observed when the Pri2:GINS interface was disrupted together with both Pol12:Mcm3 and Pol1:Ctf4 (Figure S5D, lane 5) and when the four sites that individually contribute to DNA replication were simultaneously disrupted (Figure S5D, lane 10). In contrast, we observed robust leading-strand replication and some long lagging-strand products when the Pri2:Mcm3, Pol12:Mcm3, and Pol1:Ctf4 interfaces were simultaneously targeted (Figures S5C, lane 8 and S5D, lane 9), indicating that the interaction between Pri2_Nterm_ and GINS is sufficient to support the necessary priming to start leading-strand replication. These data demonstrate that four distinct interfaces between Pol α-primase and the replisome contribute to nascent-strand priming and that collectively they are essential for efficient *in vitro* DNA replication. Importantly, the data also indicate that the contribution of each interface is not equal: disruption of the interface between Pri2_Nterm_ and GINS (site d) is most deleterious for lagging-strand replication followed by the interface between Pri2_NTD_ and Mcm3 (site b), whereas the interactions between Pol1 and Ctf4 (sites e_i_/e_ii_) and Pol12_NTD_ and Mcm3 (site c) make more minor contributions.

### Pol α-primase mutants *in vivo*

Priming at replication forks is an essential function of Pol α-primase and therefore the key interactions we have identified should be critical for cell growth. To test this, we generated budding yeast strains with mutations targeting the Pri2:GINS (Pri2-AAA) and Pri2:Mcm3 (Mcm3-CR) interfaces (Figure 2A, sites b and d). In the Pri2-AAA allele, amino acids F2, R3, and Q4 are substituted to alanine. Figure S5E shows that Pol α-primase complexes containing Pri2-AAA and Pri2-Δ2-8 displayed almost indistinguishable behavior in *in vitro* replication assays. Colony growth of both *pri2-AAA* and *mcm3-CR* cells was comparable to control cells, indicating that priming was occurring at sufficient levels to permit relatively normal DNA replication (Figure S5F). We therefore combined the *pri2-AAA* and *mcm-CR* mutations. This resulted in a profound reduction in colony size relative to control cells, consistent with these cells having DNA replication defects (Figures 3E and S5G), which is concordant with the near absence of lagging-strand products in *in vitro* reactions when these interfaces are disrupted (Figures 3D, lane 6 and S5E, lanes 5 and 6).

Although the lack of obvious growth defects for *pri2-AAA* and *mcm3-CR* cells was somewhat surprising, previous work has shown that budding yeast are reasonably tolerant of reduced Pol α-primase levels.^52^ Moreover, colony growth of *pol1-F1463A* cells in which the interaction between primase and the Pol1 C terminus is disrupted, was comparable to control cells.^45^ However, *pol1-F1463A* is synthetic lethal with deletion of the gene encoding the apical checkpoint kinase Mec1, the ortholog of ATR, indicating that these cells do in fact have DNA replication defects.^45^ We therefore wondered if *pri2-AAA and mcm3-CR* might have subtle DNA replication defects that render cells dependent on checkpoint activation. Figure 3F shows that deletion of *MEC1* in combination with *mcm3-CR* had minimal effect on colony growth. In contrast there was a notable reduction in colony size when *mec1Δ* was combined with *pri2-AAA* (Figure 3G), revealing that tethering of Pol α-primase to CMG via the Pri2:GINS interface is essential for unperturbed DNA replication in budding yeast.

### Human Pol α-primase replisome structure

**Figure 4:**
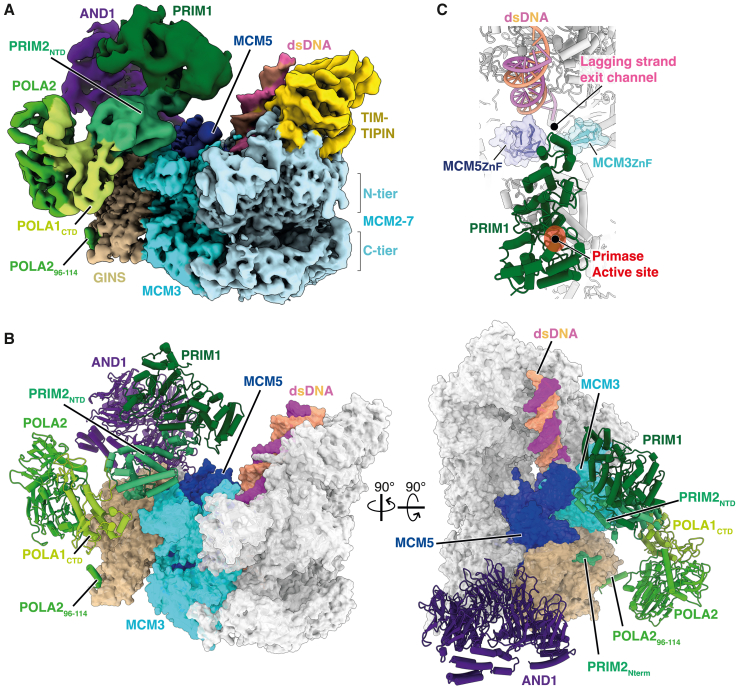
Structure of Pol α-primase in a human replisome assembled on fork DNA with a 60-nt 5′ flap (A) Composite cryo-EM map of the human replisome containing Pol α-primase, assembled on forked DNA containing a 60 nucleotide 5′ flap (Figure S1A). The map was derived from combining individual focused refinements and is colored according to chain occupancy. (B) Atomic model for the human Pol α-primase associated replisome, derived from cryo-EM data displayed in (A). Regions of CMG that interact directly with Pol α-primase are colored. (C) Focused view of PRIM1 showing its position at the mouth of the exit channel for lagging-strand ssDNA.

Because priming is fundamental for genome duplication, we considered it likely that key features of the mechanism targeting Pol α-primase to prime DNA synthesis were conserved. To examine this directly we determined the cryo-EM structure of a human replisome containing CMG, TIMELESS-TIPIN, AND-1, CLASPIN, Pol α-primase and a DNA replication fork with a 60 nt 5′ ssDNA flap (Figures S6A–S6D; Table 1). Similar to the yeast replisome, in addition to well resolved cryo-EM density for CMG, TIMELESS-TIPIN, and AND-1, poorly resolved density extended from the N-tier face of CMG atop MCM3 (Figures S6E–S6J). Following focused classification and refinement (Figure S6C) this density could be unambiguously assigned to Pol α-primase enabling assignment of PRIM1 (residues 9–349 and 386–408), the N terminus (residues 1–5) and NTD (residues 17–252) of PRIM2, the CTD of POLA1 (residues 1,279–1,445 and 1,448–1,458) and the majority POLA2 (residues 96–114 and 170–598) (Figures 4A, 4B, and S7A–S7C; Video S2).

Video S2. Overview of the structure of Pol α-primase in the human replisome, related to Figure 4 First, individual replisome components are sequentially colored and labeled to aid in their visualisation and the interpretation of the overall structure. The video then highlights each pol α-primase interface with the replisome (sites b, d, g, and e) using different views. Regions of the model forming an interaction interface are displayed using transparent surface rendering.

**Figure 5:**
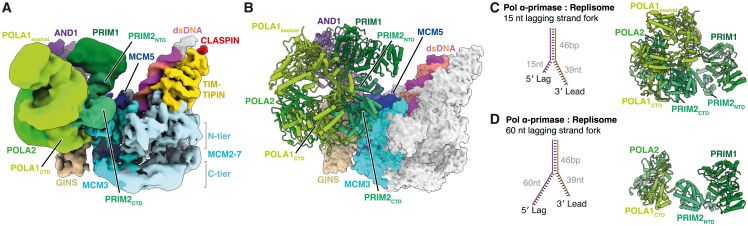
Structure of Pol α-primase in a human replisome assembled on fork DNA with a 15-nt 5′ flap (A) Cryo-EM reconstruction of the Pol α-primase associated human replisome engaged on a DNA fork containing a 15 nucleotide 5′ flap. Map colored according to subunit occupancy. (B) Atomic model for the human Pol α-primase associated replisome, derived from cryo-EM data displayed in (A). Regions of CMG that interact directly with Pol α-primase are colored. (C and D) Comparison of Pol α-primase from human replisomes bound to forked DNA with a 15 nt 5′ flap (C) and 60 nt 5′ flap (D) as illustrated.

The conformation of human Pol α-primase and its positioning in the replisome are remarkably similar to yeast (Figures S7D and S7E), with the catalytic PRIM1 subunit again positioned above the mouth of the lagging-strand template exit channel (Figures 4A–4C). Similar to yeast, POLA1_exo-cat_ is invisible when CMG is bound to replication fork DNA but is visualized stably engaging Pol α-primase in reconstructions lacking DNA, where it adopts the conformation observed for apo human Pol α-primase^46^ (Figures S7F and S7G). This suggested that the conformation of Pol α-primase in the human replisome might represent a DNA engaged state. To investigate this further, we repeated our human replisome sample preparation and cryo-EM analysis as before, but with a replication fork containing a 15 nt 5′ ssDNA flap that we reasoned would be too short to fully engage Pol α-primase (Figures S8A–S8H; Table 1). In the resulting 3D reconstructions, clear density is observed for the POLA1_exo-cat_ domain, both when CMG is bound to the replication fork and when CMG is not engaging DNA (Figures 5, S8I, and S8J). In both situations, Pol α-primase adopts the same conformation as observed for the human apo structure,^46^ strongly suggesting that Pol α-primase is bound to lagging-strand template ssDNA when the human replisome is assembled on a replication fork with a 60 nt 5′ flap.

### Conservation of Pol α-primase tethering

**Figure 6:**
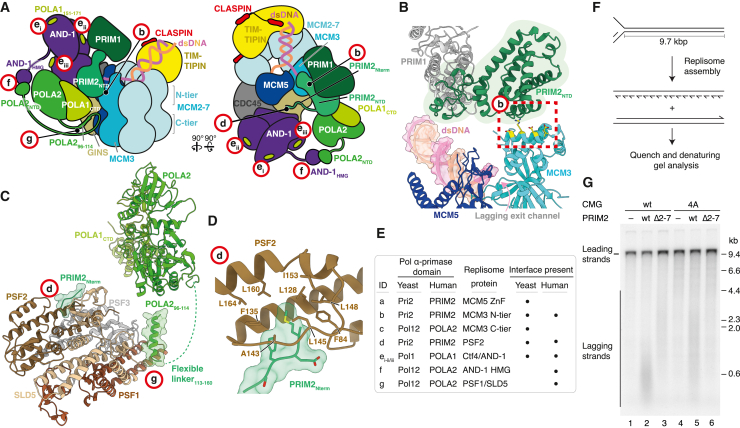
Structural basis for Pol α-primase recruitment to the human replisome for priming (A) Schematic of the human replisome engaged by Pol α-primase. Red circled labels indicate protein-protein interaction sites between Pol α-primase and the replisome. (B) Atomic model highlighting the interface between PRIM2_NTD_ (green) and the MCM3 (cyan) N-terminal helical domain (site b). Residues colored yellow with side chains displayed are those targeted for mutational analysis. (C) Atomic model highlighting the interfaces between PRIM2_Nterm_ and the PSF2 subunit of GINS (site d) and the POLA2 N-terminal helix (residues 96–114) and both PSF1 and SLD5 (site g). (D) Zoomed in view of the PRIM2_Nterm_:PSF2 interface (site d). (E) Table summarizing the protein-protein interfaces between Pol α-primase and the replisome in both budding yeast and human. Each discrete site is assigned a letter identifier corresponding to the labeling in (A) and Figure 2A. (F) Schematic of the forked DNA template and anticipated products of *in vitro* DNA replication with purified human proteins.^27^ (G) Denaturing agarose gel analysis of an *in vitro* DNA replication reaction performed as in (A) with the indicated proteins for 20 min.

Human Pol α-primase is tethered directly to CMG via three small interfaces that are all occupied independently of DNA engagement state (Figures 6A–6D and S9A–S9C; Video S2). PRIM2 binds to MCM3 (site b) and GINS (site d) in an analogous manner to Pri2 in the budding yeast replisome (Figures 6B–6D, S9D, and S9E), consistent with these binding sites being the most important for priming in yeast (Figures 3C and 3D). The PRIM2:MCM3 interface involves charged residues on α4 and the α3-4 linker of the PRIM2_NTD_ that form electrostatic contacts with four conserved residues on α1 of MCM3 (Figures 2C, 6B, and S9D). Binding of PRIM2 to GINS is mediated by the N terminus of PRIM2, with amino acids M1 and F3—invariant in Metazoa—projecting into a surface-exposed hydrophobic pocket on PSF2 in a comparable manner to Pri2-F2 in yeast (Figure 6D). Continuous cryo-EM density links the last modeled residue of PRIM2_Nterm_ (G5) and the first modeled residue of PRIM2_NTD_ (Q17) indicating this interface spatially constrains the position of the PRIM2_NTD_ and PRIM1 (Figure S9E). In addition to sites b and d, a flexibly tethered helix in POLA2 (residues 96–114) interacts with PSF1 and SLD5 (site g, Figures 6A, 6C, and S9F). Although this helix is predicted to be absent from Pol12, we note the presence of low-resolution Pol α-primase-dependent density on the surface of Psf1 in our budding yeast replisome maps, suggesting a similar binding site could be present in the yeast replisome (Figure S9G). We also note that binding of the POLA2 helix to PSF1 and SLD5 will localize the POLA2 NTD—that binds to the C-terminal AND-1 HMG-box^32^ (labeled site f in Figure 6A)—to this region of the replisome because the POLA2 helix and NTD are separated by a short (12 aa) linker. In contrast to the budding yeast replisome, we find no evidence of PRIM2_NTD_ binding to the MCM5 ZnF, indicating it is not a conserved mode of interaction and perhaps explaining why the yeast Pri2-5A mutant did not display a lagging-strand replication defect (Figure 3C). Finally, consistent with reports that AND-1 binds the unstructured POLA1 N terminus (residues 151–171),^32^^,^^33^ we observe a small region of Pol α-primase dependent density on the C-terminal α-helical domain of each AND-1 monomer (labeled sites e_i–iii_, Figures S9H and S9I), indicating that Pol1 can access all available binding sites on the AND-1 trimer.

The conservation of the PRIM2:GINS and PRIM2:MCM3 interfaces (Figure 6E) suggested they would be important for priming in the human replisome. To test this directly we purified a Pol α-primase complex lacking the PRIM2 N terminus (PRIM2-Δ2-7) and a CMG complex where four conserved residues on helix α1 of MCM3 were mutated to alanine (MCM3-4A) (Figures S9J–S9L) and analyzed them in an *in vitro* human DNA replication system that we recently developed (Figure 6F).^27^ Here, replisomes assembled around purified CMG at model replication forks perform leading and lagging-strand DNA replication at rates comparable to those measured in cultured human cells. Figures 6G and S9M show that lagging-strand products distributed around ∼0.6 kb in length were synthesized with wild-type proteins. These products were substantially longer when the PRIM2:GINS interface (PRIM2-Δ2-7) was disrupted (Figure 6G, lanes 2 and 6). While disruption of the PRIM2:MCM3 (MCM3-4A) interface was less severe, there was still a notable increase in the length of lagging-strand products, which were longer compared with when the AND-1_HMG_:POLA2 interface was abolished (AND-1-ΔHMG) (Figures 6G and S9M). Disruption of multiple interfaces in the same reaction further compromised lagging-strand replication compared with single site mutants (Figure S9M). Collectively, these data indicate that key anchor points for attaching Pol α-primase to CMG to facilitate primer synthesis at replication forks are structurally and functionally conserved between yeast and human.

## Discussion

By determining cryo-EM structures of budding yeast and human replisomes that are poised to initiate primer synthesis, we have elucidated a conserved mechanism for targeting Pol α-primase to replication forks for priming. The positioning of the catalytic Pri1/PRIM1 subunit at the mouth of the exit channel for lagging-strand template ssDNA explains how Pol α-primase functions so efficiently on this template strand and reveals a mechanism for primase to overcome competition with RPA for access to the DNA template. By contrast, the unwound leading-strand template exits CMG ∼150 Å away from Pri1/PRIM1 on the opposite side of the replisome, which is presumably not conducive for leading-strand priming, thus explaining why the core yeast replisome cannot efficiently restart leading-strand replication by repriming downstream of DNA damage^7^^,^^8^ or secondary structures^10^ and why lagging-strand primers are used to start leading-strand replication.^1^^,^^3^^,^^4^

Pol α-primase is targeted to the lagging-strand template for priming via a complex multisite interaction network involving several direct interactions with CMG. These interactions explain why Pol α-primase tethering by Ctf4/AND-1 is dispensable for DNA replication.^27^^,^^34^^,^^36^^,^^37^ The primary function of Ctf4/AND-1-dependent tethering of Pol α-primase is to facilitate the transfer of parental histones to the lagging strand.^34^^,^^53^ It will be interesting to discover why Ctf4/AND-1-dependent tethering is required for this activity and why the interactions between Pol α-primase and CMG that we have identified cannot fulfill this role. In both the yeast and human replisomes, the majority of Pol α-primase docking sites—including the crucial interaction between Pri2/PRIM2 and GINS—are mediated by regions of Pol α-primase situated at the end of, or within, unstructured linker regions, thereby providing flexible tethering points. Of the interactions that contribute to priming, only binding of the Pri2/PRIM2 NTD to MCM3 involves the association of two large rigid bodies. However, this interface is frequently disengaged and, due to its electrostatic nature, permits considerable motion between the two domains. This suggests that, although the positioning of Pol α-primase at the mouth of the lagging-strand template exit channel is crucial for priming, it is also important that primase is not rigidly fixed in this location. We hypothesize that flexible tethering of Pol α-primase in the replisome is required to allow other proteins access to key binding sites on CMG. For example, the E3 ubiquitin ligase that regulates replisome disassembly (Cul2^LRR1^ in human and SCF^Dia2^ in budding yeast) binds across the lagging-strand template exit channel^42^ and this binding site is inaccessible when Pri2/PRIM2 is bound to MCM3. Flexible tethering may also function to enable Pol α-primase to remain associated with the replisome while it undergoes conformational changes during the primer synthesis reaction.

Our structures indicate that incorporation of Pol α-primase into the replisome does not induce conformational changes in CMG that are likely to modulate helicase activity. Consequently, concomitant primer synthesis and template unwinding will result in increasing lengths of ssDNA being formed between the primase/DNA polymerase active sites and the point of template unwinding, thereby generating what has been termed a “priming loop.”^54^^,^^55^ Currently, we do not know whether Pol α-primase remains fully engaged with CMG throughout the priming reaction, or whether the multiple docking sites are utilized dynamically. We consider it likely that Pol α-primase remains associated with CMG via at least one docking site for the entirety of the priming cycle given the prolonged replisome association kinetics that have been observed in single molecule experiments.^35^^,^^56^ The conformational dynamics of Pol α-primase and its utilization of docking sites during primer synthesis are interesting subjects for future investigation that we anticipate will also influence the disposition of priming loops in the replisome.

Considerable recent progress has been made in delineating the mechanisms of primer synthesis including the molecular basis for DNA primer initiation,^57^ how Pol α-primase activity is coordinated by the CST complex during telomeric C strand fill-in.^47^^,^^58^^,^^59^ Our work represents another important advance by revealing a conserved mechanism for targeting Pol α-primase to replisomes to prime eukaryotic DNA replication and also provides a platform to visualize additional key intermediates during this fundamental process.

### Limitations of the study

Our structures of budding yeast and human Pol α-primase bound to the replisome likely only represent a small subset of conformations that Pol α-primase adopts during the priming cycle. Moreover, while our data strongly support the conclusion that Pol α-primase is engaging ssDNA in both the yeast and human replisomes, it is not possible to determine precisely which step of the priming cycle the structures represent. Although the structures provide important insights into how Pol α-primase is targeted to the replisome for priming, including identifying key protein:protein interaction sites, additional proteins that were not included in our replisome preparations might also modulate Pol α-primase activity at replication forks. For example, subunits of Pol α-primase have been reported to interact directly with Mcm10, RPA, and Pol δ. Therefore, an important future goal will be to determine structures of more complete replisomes performing lagging-strand replication to visualize intermediates along the primer synthesis pathway, the handoff of primers from Pol α-primase to Pol δ, and gain insights into how proteins such as RPA modulate the activity of Pol α-primase in the context of the replisome.

## STAR★Methods

### Key resources table

**Table:** 

REAGENT or RESOURCE	SOURCE	IDENTIFIER
**Bacterial and virus strains**		

*E. coli* 5-alpha Competent (High Efficiency)	New England Biolabs	Cat# C2987H
*E. coli*: Rosetta™ 2(DE3) strain: F^-^*ompT hsdS*_B_(r_B_^-^ m_B_^-^) *gal dcm* (DE3) pRARE2 (Cam^R^)	Novagen / Merck Millipore	Cat# 71400
*E. coli* DH10 EMBacY	Geneva Biotech	https://geneva-biotech.com/product_category/insect-cell-expression/multibac/

**Chemicals, peptides, and recombinant proteins**		

3X FLAG peptide	Sigma	Cat# F4799
Adenosine 5’-(β,γ-imido)triphosphate lithium salt hydrate (AMP-PNP)	Sigma	Cat# A2647
dNTP set	Invitrogen	Cat# 10297018
NTP set	Invitrogen	Cat# R0481
[alpha-P32]dCTP	Hatmann analytic	Cat# SRP-205
Glutaraldehyde	Sigma	Cat# G5882
Nonidet P-40 substitute (NP-40-S)	Roche	Cat# 11754599001
Glutathione Sepharose 4B	GE Healthcare	Cat# 17-0756-01
Suberic acid bis(3-sulfo-N-hydroxysuccinimide ester) sodium salt (BS^3^)	Sigma	Cat# S5799
TWEEN® 20	Sigma	Cat# P8341
Biotin	Sigma	Cat# B4501
cOmplete™, Mini, EDTA-free protease inhibitor cocktail	Sigma	Cat# 11873580001
FuGENE® HD	Promega	Cat# E2311
Insect-XPRESS protein-free insect cell media with L-glutamine	Lonza	Cat# BELN12-730Q
Sf-900™ II serum-free media	GIBCO	Cat# 10902-088
SilverQuest™ staining kit	Invitrogen	Cat# LC6070
Bovine Serum Albumin	Invitrogen	Cat# AM2616
Phusion® High-Fidelity DNA Polymerase	New England Biolabs	Cat# E0553
SphI	New England Biolabs	Cat# R0182
SapI	New England Biolabs	Cat# R0569
FspI	New England Biolabs	Cat# R0135
BsaHI	New England Biolabs	Cat# R0556
β-Glucuronidase	Sigma	Cat# G7017
TEV protease	Nagai laboratory	N/A
Proteinase K	New England Biolabs	P8107

**Recombinant proteins**		

(see also Table S2)		
Budding yeast (*S. cerevisiae*)		
Cdt1-Mcm2-7	Coster et al.^60^	N/A
ORC	Frigola et al.^61^	N/A
Cdc6	Frigola et al.^61^	N/A
DDK	On et al.^62^	N/A
Sld3/7	Yeeles et al.^38^	N/A
Cdc45	Yeeles et al.^38^	N/A
Dpb11	Yeeles et al.^38^	N/A
Sld2	Yeeles et al.^38^	N/A
GINS	Yeeles et al.^38^	N/A
Pol ε	Yeeles et al.^38^	N/A
S-CDK	Yeeles et al.^38^	N/A
Mcm10	Yeeles et al.^38^	N/A
Pol α	Yeeles et al.^38^	N/A
Ctf4	Yeeles et al.^38^	N/A
RPA	Baretić et al.^30^	N/A
Mrc1	Baretić et al.^30^	N/A
Tof1-Csm3	Baretić et al.^30^	N/A
RFC	Yeeles et al.^37^	N/A
PCNA	Yeeles et al.^37^	N/A
Pol δ	Yeeles et al.^37^	N/A
CMG	Baretić et al.^30^	N/A
Cdt1-Mcm2-7: Mcm3-CR	This study	N/A
Pol α-primase: Pol1-4A	This study	N/A
Pol α-primase: Pol12ΔN	This study	N/A
Pol α-primase: Pol1-4A⋅Pol12ΔN	This study	N/A
Pol α-primase: Pri2-Δ2-8	This study	N/A
Pol α-primase: Pri2-5A	This study	N/A
Pol α-primase: Pri2-AAA	This study	N/A
Pol α-primase: Pol1-4A⋅Pri2-Δ2-8	This study	N/A
Pol α-primase: Pol1-4A⋅Pol12-ΔN⋅Pri2-Δ2-8	This study	N/A
Pol α-primase: Pol12-ΔN⋅Pri2-Δ2-8	This study	N/A

**Human (*H. sapiens*)**		

CMG	Jones et al.^41^	N/A
CLASPIN	Jones et al.^41^	N/A
TIMELESS-TIPIN	Jones et al.^41^	N/A
AND-1	Jones et al.^41^	N/A
Pol ε	Jones et al.^41^	N/A
Pol α-primase	Baris et al.^27^	N/A
PCNA	Jones et al.^41^	N/A
RPA	Jones et al.^41^	N/A
Ctf18-RFC	Baris et al.^27^	N/A
Pol δ	Baris et al.^27^	N/A
Pol α-primase: PRIM2-Δ2-7	This study	N/A
CMG: MCM3-4A	This study	N/A
AND1: ΔHMG (Δ1017)	Baris et al.^27^	N/A

**Deposited data**		

Budding yeast (*S. cerevisiae*)		
Co-ordinate file for the Pol α-primase associated replisome in the absence of Ctf4	This study	8B9C
Co-ordinate file for the Pol α-primase associated replisome in the presence of Ctf4, CIP box site #1	This study	8B9A
Co-ordinate file for the Pol α-primase associated replisome in the presence of Ctf4, CIP box site #2	This study	8B9B
Pol α-primase associated replisome consensus refinement in the absence of Ctf4 (binned)	This study	EMD-16320
Pol α-primase associated replisome consensus refinement in the absence of Ctf4 (un-binned)	This study	EMD-16322
Tof1-Csm3 local refinement	This study	EMD-15304
Mcm2-7 C-tier local refinement	This study	EMD-15305
Pol12, Pol1CTD, Pri2NTD local refinement	This study	EMD-15306
Pol12, Pol1CTD local refinement	This study	EMD-16885
Pol α-primase associated replisome consensus refinement in the presence of Ctf4 (binned)	This study	EMD-15309
Pol α-primase associated replisome consensus refinement in the presence of Ctf4 (un-binned)	This study	EMD-15902
Ctf4 local refinement	This study	EMD-15310
Pri1, Pri2CTD local refinement	This study	EMD-16247
Pol α-primase associated replisome consensus refinement containing density for the Pol1exo/cat and Pri2CTD domains	This study	EMD-16248
Pol α-primase associated replisome consensus refinement containing density for the lagging strand DNA template and the Pri1CTD (binned)	This study	EMD-15924
Pol α-primase associated replisome consensus refinement containing density for the lagging strand DNA template and the Pri1CTD (un-binned)	This study	EMD-15303
Pol α-primase associated replisome consensus refinement where only the Pri2:Mcm5ZnF interface is engaged	This study	EMD-16323

**Human (*H. sapiens*)**		

Co-ordinate file for the Pol α-primase associated replisome	This study	8B9D
Pol α-primase associated replisome consensus refinement (un-binned)	This study	EMD-15341
MCM2-7 C-tier local refinement	This study	EMD-15340
AND-1 local refinement	This study	EMD-15342
Pol α-primase associated replisome consensus refinement containing strong PRIM1 density (binned)	This study	EMD-15349
PRIM1, POLA2, PolA1CTD, Pri2NTD local refinement	This study	EMD-15351
TIMELESS-TIPIN local refinement	This study	EMD-15356
Composite map assembled from EMD-15342:15341:15340:15349:15351:15356	This study	EMD-15904
Pol α-primase associated replisome consensus refinement, not engaged on DNA derived from dataset including a 15 nucleotide 5ʹ-flap DNA fork	This study	EMD-15918
Pol α-primase associated replisome consensus refinement, not engaged on DNA derived from dataset including a 60 nucleotide 5ʹ-flap DNA fork	This study	EMD-15923
Pol α-primase associated replisome consensus refinement, engaged on a 15 nucleotide 5ʹ-flap DNA fork	This study	EMD-15922

**Experimental Models: Cell Lines**		

Hi5	Thermo Fisher	B85502

**Experimental models: Organisms/strains**		

*S. cerevisiae* strains (See also Table S3 for additional details of strains constructed as part of this study)		
*yAM33 (Cdt1-Mcm2-7 purification)*	Coster et al.^60^	N/A
*ySDORC (ORC purification)*	Frigola et al.^61^	N/A
*ySDK8 (DDK purification)*	On et al.^62^	N/A
*yTD6 (Sld3/7 purification)*	Yeeles et al.^38^	N/A
*yTD8 (Sld2 purification)*	Yeeles et al.^38^	N/A
*yJY13 (Cdc45 purification)*	Yeeles et al.^38^	N/A
*yJY26 (Dpb11 purification)*	Yeeles et al.^38^	N/A
*yAJ2 (Pol epsilon purification)*	Yeeles et al.^38^	N/A
*yAE88 (S-CDK purification)*	Jake et al.^63^	N/A
*yAE95 (Pol alpha purification)*	Jake et al.^63^	N/A
*yAE40 (Ctf4 purification)*	Yeeles et al.^38^	N/A
*yJY106 (RPA purification)*	Baretić et al.^30^	N/A
*yJY32 (Mrc1 purification)*	Yeeles et al.^37^	N/A
*yAE48 (Tof1-Csm3 purification)*	Yeeles et al.^37^	N/A
*yAE41 (RFC purification)*	Yeeles et al.^37^	N/A
*yAE34 (Pol delta purification)*	Yeeles et al.^37^	N/A
*yJY197 (CMG purification)*	Jenkyn-Bedford et al.^42^	N/A
*yVA87 (Cdt1-Mcm2-7: Mcm3-CR)*	This study	N/A
*yVA96 (Pol α-primase: Pol1-4A)*	This study	N/A
*yJY239 (Pol α-primase: Pol12-ΔN)*	This study	N/A
*yJY232 (Pol α-primase: Pri2-5A)*	This study	N/A
*yJY241 (Pol α-primase: Pri2-Δ2-8)*	This study	N/A
*yJY242 (Pol α-primase: Pri2-AAA)*	This study	N/A
*yJY381 (Pol α-primase: Pol1-4A⋅Pol12ΔN)*	This study	N/A
*yMJ12* (*Pol α-primase*: Pol1-4A⋅Pri2-Δ2-8)	This study	N/A
*yMJ13* (*Pol α-primase*: Pol1-4A⋅Pol12-ΔN⋅Pri2-Δ2-8)	This study	N/A
*yMJ18* (*Pol α-primase*: Pol12-ΔN⋅Pri2-Δ2-8)	This study	N/A
yJY244	This study	N/A
yJY297	This study	N/A
yJY321	This study	N/A
yJY300	This study	N/A
yJY301	This study	N/A
yJY365	This study	N/A
yJY367	This study	N/A
yJY345	This study	N/A
yJY350	This study	N/A
yJY351	This study	N/A
yJY356	This study	N/A
yJY357	This study	N/A
yJY255	This study	N/A
yJY302	This study	N/A
yJY326	This study	N/A
yJY328	This study	N/A
yJY313	This study	N/A
yJY315	This study	N/A
yJY317	This study	N/A
yJY352	This study	N/A
yJY354	This study	N/A

**Oligonucleotides**		

Leading strand: 5ʹ-(Cy3)-TAGAGTAGGAAG TGAGGTAAGTGATTAGAGAATTGGAGAGT GTG(T)34T∗T∗T∗T∗T∗T – 3ʹ (∗ - phosphorothioate)	Integrated DNA Technologies (IDT)	N/A
15 Nucleotide 5ʹ-flap lagging strand: 5ʹ-GGCAGG CAGGCAGGCACACACTCTCCAATTCTCTAATCA CTTACCACACTTCCTACTCTA – 3ʹ	Integrated DNA Technologies (IDT)	N/A
60 nucleotide 5ʹ-flap lagging strand: (T)_60_ACACAC TCTCCAATTCTCTAATCACTTACCATCACTTCCT ACTCTA – 3ʹ	Integrated DNA Technologies (IDT)	N/A
MT096: 5′phos/GCTATGTGGTAGGA AGTGAGAATTGGAGAGTGTGTTTTT TTTTTTTTTTTTTTTTTTTTTTTTTTTT TTTTTTTGAGGAAAGAATGTTGGTG AGGGTTGGGAAGTGGAAGGATGG GCTCGAGAGGTTTTTTTTTTTTTTTT TTTTTTTTTTTTTTTTTT	Integrated DNA Technologies (IDT)	N/A
JY197: 5′- TTTTTTTTTTTTTTTTTTTTCACA CTCTCCAATTCTCACTTCCTACCACAT	Integrated DNA Technologies (IDT)	N/A
JY195: 5′ - CCTCTCGAGCCCATC CTTCCACTTCCCAACCCTCACC	Integrated DNA Technologies (IDT)	N/A
JY104: 5′ - GAATTGCGCTCTATGAAGTTGAC	Merck	N/A
JY105: 5′ - GAACTGCGGCTTGATAATGG	Merck	N/A
JY370: 5′ - GGACTAGGATGAGTAGCAGC	Merck	N/A
JY491: 5′ - GAGTCAGACAACCAGCAAGC	Merck	N/A
JY604: 5′ - GGTTGAAGAGCAGGCCAAGG	Merck	N/A
JY609: 5′ - TCAGGCCAAAGGTGATACGAC	Merck	N/A
VA212: 5′ - GACCTGTCGAATTCTCTCAA	Merck	N/A

**Recombinant DNA**		

vVA20	Aria and Yeeles^1^	N/A
M13mp18 ssDNA	New England Biolabs	Cat# N4040S
ZN3	Taylor and Yeeles^7^	N/A
pJFDJ5 (yeast GINS purification)	Yeeles et al.^38^	N/A
vJY19 (yeast PCNA purification)	Yeeles et al.^37^	N/A
pAM3 (yeast Cdc6 purification)	Coster et al.^60^	N/A
pET28a-Mcm10 (yeast Mcm10 purification)	Yeeles et al.^38^	N/A
YB_X1 (human RPA purification)	This study	N/A
MT_EB1 (human PCNA purification)	Jones et al.^41^	N/A
YB_2 (human CMG purification)	Jones et al.^41^	N/A
YB_1 (human CMG purification)	Jones et al.^41^	N/A
MT_01 (human CMG purification)	Jones et al.^41^	N/A
MT_BF1 (AND-1 purification)	Jones et al.^41^	N/A
MT_DB1 (CLASPIN purification)	Jones et al.^41^	N/A
MT_DF1 (TIMELESS-TIPIN purification)	Jones et al.^41^	N/A
MT_BD1 (TIMELESS-TIPIN purification)	Jones et al.^41^	N/A
MT_BH1 (human RFC purification)	Jones et al.^41^	N/A
MT_BJ1 (human RFC purification)	Jones et al.^41^	N/A
MT_BK1 (human RFC purification)	Jones et al.^41^	N/A
MT_BL1 (human RFC purification)	Jones et al.^41^	N/A
MT_BI1 (human RFC purification)	Jones et al.^41^	N/A
YB_7 (CTF18-RFC purification)	Baris et al.^27^	N/A
YB_5 (CTF18-RFC purification)	Baris et al.^27^	N/A
YB_6 (CTF18-RFC purification)	Baris et al.^27^	N/A
YB_4 (CTF18-RFC purification)	Baris et al.^27^	N/A
MT_CF1 (human Pol delta purification)	Baris et al.^27^	N/A
MT_CH1 (human Pol delta purification)	Baris et al.^27^	N/A
YB_3 (human Pol delta purification)	Baris et al.^27^	N/A
MT_FC1 (human Pol delta purification)	Baris et al.^27^	N/A
MT_BC3 (human Pol alpha-primase purification)	Baris et al.^27^	N/A
MT_AE1 (human Pol alpha-primase purification)	Baris et al.^27^	N/A
MT_AF1 (human Pol alpha-primase purification)	Baris et al.^27^	N/A
MT_AG1 (human Pol alpha-primase purification)	Baris et al.^27^	N/A
MT_U2 (human Pol epsilon purification)	Baris et al.^27^	N/A
MT_L1 (human Pol epsilon purification)	Baris et al.^27^	N/A
MT_M1 (human Pol epsilon purification)	Baris et al.^27^	N/A
MT_N1 (human Pol epsilon purification)	Baris et al.^27^	N/A
YB_8 (AND-1-ΔHMG purification)	Baris et al.^27^	N/A
YB_X2 (MCM3-4A construction)	This study	N/A
YB_X3 (CMG: MCM3-4A purification)	This study	N/A
vVA62 (CMG: MCM3-4A purification)	This study	N/A
vMJ9 (human Pol alpha-primase: PRIM2-Δ2-7)	This study	N/A
vVA52 (Cdt1-Mcm2-7: Mcm3-CR strain construction)	This study	N/A
vVA58 (yeast Pol alpha-primase: Pol1-4A strain construction)	This study	N/A
vJY186 (yeast Pol alpha-primase: Pol12-ΔN) strain construction)	This study	N/A
vJY187 (yeast Pol alpha-primase: Pol1-4A⋅Pol12-ΔN) strain construction)	This study	N/A
vJY196 (yeast Pol alpha-primase: Pri2-Δ2-8) strain construction)	This study	N/A
vJY183 (yeast Pol alpha-primase: Pri2-5A) strain construction)	This study	N/A
vJY199 (yeast Pol alpha-primase: Pri2-AAA strain construction)	This study	N/A
vJY177 (construction of Mcm3-CR (Ura3) strain)	This study	N/A
vJY206 (construction of Pri2-AAA (Ura3) strain)	This study	N/A

**Software and algorithms**		

Chimera (v1.13)	UCSF Resource for Biocomputing, Visualization, and Informatics	https://www.cgl.ucsf.edu/chimera/
ChimeraX (v1.52)	UCSF Resource for Biocomputing, Visualization, and Informatics	https://www.cgl.ucsf.edu/chimerax/
Coot (v1.0)	Paul Emsley (Medical Research Council Laboratory of Molecular Biology)	https://www2.mrc-lmb.cam.ac.uk/personal/pemsley/coot/
EPU (v2.0)	ThermoFisher Scientific (FEI)	https://www.fei.com/software/epu-automated-single-particles-software-for-life-sciences
ESPript (v3.0.7)	Patrice Gouet (Lyon University); Xavier Robert (Centre national de la recherche scientifique)	http://espript.ibcp.fr/ESPript/ESPript/
FIJI (v1.0)	National Institute of Health	https://imagej.net/Fiji/Downloads
Gautomatch (v0.53)	Kai Zhang (Medical Research Council Laboratory of Molecular Biology)	https://www.mrc-lmb.cam.ac.uk/kzhang/Gautomatch/
ImageJ (v1.50i)	National Institute of Health	https://imagej.nih.gov/ij/
ISOLDE (v1.4)	Tristan Croll (Cambridge Institute for Medical Research)	https://isolde.cimr.cam.ac.uk/
Phenix (v1.20-4459)	Cambridge University; Duke University; Lawrence Berkeley National Laboratory; Los Alamos National Laboratory	https://www.phenix-online.org/
Photoshop 2020	Adobe	https://www.adobe.com/uk/products/photoshop.html
Prism (v9.0.0)	GraphPad	https://www.graphpad.com/scientific-software/prism/
RELION (v2.1 & v3.1)	Sjors Scheres (Medical Research Council Laboratory of Molecular Biology)	https://www3.mrc-lmb.cam.ac.uk/relion/
MUSCLE	European Molecular Biology Laboratory -European Bioinformatics Institute (EMBL-EBI)	https://www.ebi.ac.uk/Tools/msa/muscle/
cryoSPARC (v3.2, v4.0 & v4.1)	Structura Biotechnology	https://cryosparc.com/updates
CTFFIND-4.1	The Grigorieff Lab	https://grigoriefflab.umassmed.edu/ctffind4
AlphaFold (v2.0)	DeepMind	https://www.deepmind.com/open-source/alphafold
AlphaFold-multimer (v2.0)	DeepMind	https://github.com/deepmind/alphafold
ColabFold (v1.5.2)	Ovchinnikov & Steinegger Labs	https://github.com/sokrypton/ColabFold
Epson Scan 3.9.3.0EN	Seiko Epson Corporation	https://www.epson.co.uk
Amersham Typhoon (1.1.0.7)	Cytiva	

**Other**		

Amicon Ultra Centrifugal Filter Units	Millipore	Cat# UFC901096
QUANTIFOIL Copper 400 mesh R2/2 holey carbon TEM grids	Electron Microscopy Sciences	Cat# Q450CR2
HiTrap Blue HP	GE Healthcare	Cat# 17-0412-01
HiTrap DEAE Fast Flow	GE Healthcare	Cat# 17-5055-01
HiTrap Heparin HP	GE Healthcare	Cat# 17-0406-01
HiTrap SP HP	GE Healthcare	Cat# 29-0513-24
HiTrap SP FF	GE Healthcare	Cat# 29-0513-24
IgG Sepharose Fast Flow	GE Healthcare	Cat# 17-0969-01
StrepTactin Superflow high-capacity resin	IBA life sciences	Cat# 2-1208-002
MonoQ PC 1.6/5	GE Healthcare	Cat# 17-0671-01
MonoS 5/50 GL	GE Healthcare	Cat# 17-5168-01
Ni-NTA Agarose	QIAGEN	Cat# 30210
Superdex 200 Increase 10/300 GL	GE Healthcare	Cat# 28-9909-44
Superose™ 6 Increase 10/300 GL	GE Healthcare	Cat# 29-0915-96
Sepharose 4B	Sigma	Cat# 4B200
Microspin G-50 columns	GE Healthcare	Cat# GE27-5330-02
Anti-FLAG M2 affinity gel	Sigma	Cat# A2220
Bio-Gel HT (Hydrated) Hydroxyapatite	Bio-Rad	Cat# 130-0150
Calmodulin-Sepharose 4B	GE Healthcare	Cat# 17-0529-01
Criterion XT 4-12% Bis-Tris precast gels	BioRad	Cat# 3450124
NuPAGE™ 4-12% Bis-Tris precast gels	Thermo Fisher	Cat# NPO323box
Whatman 3 MM paper	Cytivia	Cat# 11895375
BAS-IP MS phosphor screen	Cytivia	Cat# 28956474
Amersham Hyperfilm MP	Cytivia	Cat# 28906842

### Resource availability

#### Lead contact

Further information and requests for resources and reagents should be directed to and will be fulfilled by the lead contact, Joseph Yeeles (jyeeles@mrc-lmb.cam.ac.uk).

#### Materials availability

Budding yeast strains and protein expression plasmids will be made available on request.

### Experimental model and study participant details

*S. cerevisiae* strains constructed for genetic experiments were based on the W303 genetic background. Comprehensive information regarding the genotypes of these *S. cerevisiae* strains can be found in Table S4.

### Method details

#### Protein expression and purification

Details of protein expression plasmids and strains made during this study can be found in Tables S1 and S2. An overview of the purification strategy for each protein is provided in Table S3. All wild type budding yeast proteins were expressed and purified as described previously.^30^^,^^37^^,^^38^^,^^42^ Cdt1-Mcm2-7 and Pol α-primase mutants / truncations were purified using the same procedure as for the wild type proteins. Human proteins were expressed and purified as described previously.^27^^,^^41^^,^^42^ Human Pol α-primase (Pri2-Δ2-7) was expressed and purified as described for the wild type protein.^27^ Human CMG (MCM3-4A) was expressed by coinfecting Hi5 cells at a density of 1 x 10^6^ cells/ml with four viruses (generated as previously described^42^) expressing: MCM2, MCM5, MCM3-4A (vVA62); MCM7, MCM4, MCM6 (YB_X3); Cdc45 (MT_O1)^41^; PSF1, PSF2, PSF3, SLD5 (YB_1).^41^ Cells were grown for 72 hours before harvest by centrifugation. Protein purification was performed as described previously for the wild type protein.^41^

#### TIMELESS-TIPIN purification

Cells from a 1L culture were resuspended in lysis buffer (25 mM HEPES-KOH pH 7.2, 150 mM KCl, 5% glycerol, 0.5 mM TCEP, 0.01% NP-40-S) + protease inhibitors (cOmplete, EDTA-free, one tablet per 50 ml buffer) and lysed by dounce homogenization. Insoluble material was cleared by centrifugation (235,000 *g*, 4°C, 45 min) and 0.5 ml Strep-Tactin XT superflow high capacity resin was added to the lysate. Following a 30 min incubation at 4°C the resin was collected in a 20-ml column and was washed with 50 ml lysis buffer. The resin was resuspended in ∼ 2 ml lysis buffer and TEV protease was added to 100 ug/ml. The sample was incubated at 4°C overnight with gentle rotation. The sample was collected and applied to a 1 ml HiTrap Q HP column (GE Healthcare) equilibrated in 25 mM HEPES-KOH pH 7.2, 150 mM KCl, 5% glycerol, 0.5 mM TCEP, 0.01% NP-40-S. Proteins were eluted with a 20 column volume gradient from 150 to 1,000 mM KCl and peak fractions containing TIMELESS-TIPIN were pooled, concentrated to ∼ 500 μl in an Amicon Ultra-15 30 kDa MWCO concentrator and applied to a Superdex 200 Increase 10/300 gel filtration column (GE Healthcare) equilibrated in 25 mM Tris–HCl pH 7.2, 5% glycerol, 0.01% NP-40-S, 1 mM DTT, 150 mM NaCl. Peak fractions were pooled, frozen in liquid nitrogen and stored at −80°C.

#### Preparation of fork DNA for cryo-EM

To prepare forked DNA for cryo-EM sample preparation, leading and lagging strand oligonucleotides (Integrated DNA Technologies) were mixed at equimolar ratios in annealing buffer (25 mM HEPES-NaOH, pH 7.5, 150 mM NaOAc, 0.5 mM TCEP, 2 mM Mg(OAc)_2_) and gradually cooled from 80°C to room temperature. Leading strand oligo: 5′-(Cy3)-TAGAGTAGGAAGTGAGGTAAGTGATT

AGAGAATTGGAGAGTGTG(T)_34_ T^∗^T^∗^T^∗^T^∗^T^∗^T – 3′

^∗^ Denotes a phosphorothioate backbone linkage. 15 Nucleotide 5′-flap lagging strand oligo:

5′-GGCAGGCAGGCAGGCACACACTCTCCAATTCTCTAATCACTTACCACACTTCCTACT

CTA – 3′. 60 nucleotide 5′-flap lagging strand sequence:

5′-TTTTTTTTTTTTTTTTTTTTTTTTTTTTTTTTTTTTTTTTTTTTTTTTTTTTTTTTTTTTAC

ACACTCTCCAATTCTCTAATCACTTACCATCACTTCCTACTCTA – 3′

#### Replisome assembly for cryo-EM

Reconstitution reactions were set up to yield a final volume of 300 μl, containing 150 nM CMG with a 1.5-fold molar excess of replisome proteins and fork DNA in reconstitution buffer (25 mM HEPES-NaOH pH 7.6, 150 mM NaOAc, 0.5 mM TCEP, 500 μM AMP-PNP, 10 mM Mg(OAc)_2_). Firstly, CMG was incubated with fork DNA for 10 min on ice in an 80 μl reaction. Next, the additional proteins were added in the following order: Ctf4/AND-1, Tof1-Csm3/TIMELESS-TIPIN, Mrc1/CLASPIN and Pol α-primase. The reaction volume was then adjusted to 300 μl using reconstitution buffer before being incubated for 20 min on ice. Following incubation, 132 μl of the reconstitution reaction was loaded separately onto two 10-30% glycerol gradients, each containing crosslinker. The remaining 36 μl of the reconstitution reaction was diluted in reconstitution buffer to 132 μl and this sample loaded onto a glycerol gradient prepared in the absence of crosslinker. Glycerol gradients were prepared as previously described^30^: Buffer A (40 mM HEPES-NaOH, pH 7.5, 150 mM NaOAc, 0.5 mM TCEP, 10% v/v glycerol, 0.5 mM AMP-PNP and 3 mM Mg(OAc)_2_) was layered on top an equal volume of Buffer B (Buffer A, except 30% v/v glycerol, 0.16% glutaraldehyde [Sigma] and 2mM bis(sulfosuccinimidyl)suberate (BS^3^, ThermoFisher Scientific)) in a 2.2 mL TLS-55 tube (Beranek Laborgerate) and gradients made using a gradient-making station (Biocomp Instruments, Ltd.) before cooling on ice. The sample was separated by centrifugation (200,000g, 4°C, 2 h) prior to manual fractionation. SDS-PAGE gel analysis was used to identify two peak fractions from each gradient containing crosslinker (total volume 368 μl) as previously described.^41^ These fractions were then buffer exchanged and concentrated prior to being immediately used for cryo-EM grid preparation as previously described.^41^

#### Cryo-EM data collection

##### Budding yeast replisome + Pol α-primase + 60 nucleotide 5′-flap DNA fork

A total of 12,819 raw movies were acquired using a 300 keV Titan Krios microscope (FEI) equipped with a K3 direct electron detector (Gatan) operated in electron counting mode using the EPU automated acquisition software (ThermoFisher) with “Faster Acquisition” mode (AFIS) enabled. A slit width of 20 eV was used for the BioQuantum energy filter. Data were collected in super-resolution mode bin 2 at an effective pixel size of 0.86 Å/pixel over a defocus range of -1.8 to -3.5 μm. Movies were dose-fractionated into 39 fractions over a 4 s exposure, resulting in a total dose of 39.2 e^-^/Å^2^.

##### Human replisome + Pol α-primase + 60 nucleotide 5′-flap DNA fork

A total of 7,355 raw movies were acquired using a 300 keV Titan Krios microscope (FEI) equipped with a K3 direct electron detector (Gatan) operated in electron counting mode using the EPU automated acquisition software (ThermoFisher) with “Faster Acquisition” mode (AFIS) enabled. A slit width of 20 eV was used for the BioQuantum energy filter. Data were collected at a pixel size of 0.86 Å/pixel using a defocus range of -2.1 to -3.5 μm. Movies were dose-fractionated into 39 fractions over a 4 s exposure resulting in a total dose of 47.4 e^-^/Å^2^.

##### Human replisome + Pol α-primase + 15 nucleotide 5′-flap DNA fork

A total of 6,718 raw movies were acquired using a 300 keV Titan Krios microscope (FEI) equipped with a Falcon III direct electron detector (Thermo) operated in linear mode using the EPU automated acquisition software (ThermoFisher). Data were collected at a pixel size of 1.07 Å/pixel using a defocus range of -0.9 to -3.5 μm. Movies were dose-fractionated into 39 fractions over a 1 s exposure resulting in a total dose of 88.5 e^-^/Å^2^.

#### Cryo-EM data processing

##### Budding yeast replisome + Pol α-primase + 60 nucleotide 5′-flap DNA fork

The data processing pipeline outlined here is schematised in Figure S2. Data were processed using either RELION-3^64^^,^^65^^,^^66^^,^^67^ (henceforth referred to as RELION) or cryoSPARC-3^68^ (henceforth referred to as cryoSPARC) unless otherwise stated. 12,819 39-fraction movies were aligned and dose-weighted (1.00513 e^-^/Å^2^/fraction, 5 x 5 patches, 150 Å^2^ B-factor) using RELION’s implementation of a MotionCor2-like program.^69^ CTF parameters were estimated using CTFFIND-4.1^70^ and 112 poor-quality micrographs excluded from future processing. Particles were picked using RELION’s Laplacian-of-Gaussian (LoG) function providing a minimum diameter of 200 Å and maximum of 350 Å. 2,003,322 picked particles were extracted using a box size of 430 Å. During extraction the data were down-sampled to a pixel size of 3.44 Å/pixel. One round of RELION 2D classification was carried out and 1,623,209 particles were selected for further classification. Four successive rounds of RELION 3D classification (regularisation parameter, T = 4), each generating 6 classes, were carried out using a previously obtained map of the budding yeast replisome as a reference (EMD-10227).^30^ Class selection was based upon the presence of secondary structure features within CMG. The first two round of 3D classification were performed with a 250 Å diameter circular mask to focus classification on the CMG, with the subsequent two rounds using a more dilated mask of 380 Å to incorporate signal from Pol α-primase.

202,655 particles containing poor density for dsDNA were selected for an additional two rounds of 3D classification in RELION. This resulted in the selection of 44,871 particles in classes displaying density for both Pol α-primase and CMG in the absence of dsDNA. These particles were refined using 3D auto-refinement in RELION generating a reconstruction, after post-processing, at 7.4 Å resolution. In order to enrich for replisomes stably bound by Pol α-primase in the absence of dsDNA, signal subtraction was carried out in RELION focussing on the interface between Pol α-primase and Mcm3. The refined reconstruction was low-pass filtered to 10 Å in UCSF Chimera^71^ and a soft mask was generated covering density for both Pol α-primase and the Mcm3 N-terminal helical domain. Subtracted particles were re-centred on the mask and sub-classified using 3D classification without alignment in RELION. 18,412 particles in classes containing strong density for both Pol α-primase and the Mcm3 N-terminal helical domain were selected and reverted to the original (non-subtracted) particles prior to refinement using 3D auto-refine in RELION. This generated a reconstruction, following postprocessing, in RELION at 6.8 Å resolution (Figure S3J).

Returning to the results of the fourth overall round of 3D classification, 884,301 particles were selected for further processing to enrich for replisome complexes bound by Pol α-primase engaged on DNA. These particles were re-extracted using an un-binned pixel size of 0.86 Å in a 450 Å box and submitted for refinement using RELION 3D auto-refine, yielding a reconstruction at 3.5 Å following postprocessing. These data were submitted for two rounds of iterative per-particle motion correction using dataset-trained particle polishing in RELION^72^ and RELION CTF-refinement^64^ (beamtilt and trefoil correction, anisotropic magnification correction, and per-particle defocus and astigmatism CTF correction). These data were refined to an improved resolution of 3.0 Å following postprocessing in RELION. At this stage of processing the density for CMG, Ctf4, Tof1-Csm3 and DNA was of high quality yet the Pol α-primase density was disordered and fragmented with only the Mcm5_ZnF_ contact preserved at appropriate map thresholds.

In order to identify classes in which Pol α-primase was stably engaged with the replisome, the strategy previously described to enrich for Pol α-primase stably engaged on replisomes in the absence of DNA was employed. Signal subtraction was carried out using a mask encompassing both Pol α-primase and the Mcm3 N-terminal helical domain. These subtracted particles were than sub-classified using 3D classification without alignment, resulting in 614,228 particles in classes with improved Pol α-primase density. These data were reverted to the original non-subtracted particles and refined using RELION 3D auto-refine. The subtraction and subclassification process was then iterated to select for 3D classes representing 588,597 particles with improved Pol α-primase density. These particles were imported into cryoSPARC and subsequently down sampled to a pixel size of 1.72 Å/pixel to boost the signal-to-noise for spatial frequencies describing secondary structure elements. These data were then classified in 3D via heterogeneous refinement using five different replisome reference maps (composition indicated in Figure S2). Classes in which Pol α-primase was only engaged at both the Mcm5_ZnF_ and Psf2 sites were selected representing 434,311 particles. These particles were refined using non-uniform refinement,^73^ with a pixel size of 0.86 Å, to 3.0 Å resolution (Figure S1N). To aid interpretation, all non-uniform and local refinements completed in cryoSPARC were subsequently locally filtered using their respective local resolution maps.

Returning to the previous heterogeneous refinement, classes with improved Pol α-primase density representing 140,426 particles were selected and further classified via heterogeneous refinement with six identical 3D references, the results of which are used as the input for processing strategies 1-4:

Strategy 1: Classes representing 84,628 particles were selected based on the presence of both strong Ctf4 and Pol α-primase density. A soft mask was generated in UCSF Chimera^71^ covering Pol α-primase and masked 3D classification without alignment carried out using five identical 3D references in cryoSPARC (target resolution 8 Å, initialisation mode PCA). Masks generated for use in cryoSPARC were binarised using the vop_threshold command in UCSF Chimera^71^ and a soft padding width applied using the Volume Tools in cryoSPARC (mask softness=5^∗^resolution(Å)/pixel-size(Å)). Classes representing 54,970 particles were selected based upon Pol α-primase Pri2_NTD_ engagement with the Mcm3 N-terminal helical domain. A consensus refinement for these unmasked particles was carried out using non-uniform refinement in cryoSPARC, with an binned pixel size, resulting in a reconstruction at 4.6 Å resolution in which the MCM C-tier adopts conformation II. This process was iterated to generate a reconstruction, with an un-binned pixel size of 0.86, at 3.5 Å resolution (Figure S1M). In this reconstruction Pol α-primase engages the replisome via contacts with Mcm5_ZnF_, Psf2, Mcm3 N-terminal helical domain, Mcm3 AAA^+^ domain and Ctf4. Reconstructions in which these sites are all engaged (+/- Ctf4) we define as the “fully engaged” complex. Soft masks were generated covering both the visible regions of Ctf4/Cdc45/GINS in the resulting map, and the remainder of the density. These maps were used to subtract the non Ctf4/Cdc45/GINS density from the particle images and subsequently carry out masked local refinement of the Ctf4/Cdc45/GINS region in cryoSPARC. Local refinement was carried out using the default parameters and a fulcrum point defined by the mask centre, resulting in a reconstruction at 3.3 Å resolution for Ctf4/Cdc45/GINS (Figure S1H).
Strategy 2: Classes representing 73,884 particles were selected following heterogeneous refinement displaying strong Pol α-primase density in the absence of Ctf4 with the Mcm2-7 C-tier in conformation II. Masked 3D classification without alignment using ten 3D references was carried out in cryoSPARC, as described in strategy 1, to enrich for classes where the Pol α-primase Pri2_NTD_ is engaged with the Mcm3 N-terminal helical domain. Classes representing 53,964 particles were selected for two non-uniform refinement jobs in cryoSPARC using either a pixel size of 0.86 Å or 2.27 Å, resulting reconstructions at both 3.5 Å and 4.6 Å resolution respectively for the fully engaged complex in the absence of Ctf4 (Figure S1E). Signal subtraction and masked local refinement was carried out in cryoSPARC using a pixel size of 0.86 Å, as described in Strategy 1, focussing on both the Pol12/Pol1_CTD_ (Figure S1I) and Pol12/Pol1_CTD_/Pri2_NTD_ (Figure S1J) regions of the complex, yielding reconstructions at 4.8 Å and 5.0 Å resolution respectively. In order to improve the noisy density adjacent to Pri1, a soft mask covering this region and Pri1 was generated in UCSF Chimera^71^ based upon a low-pass filtered map derived from the consensus non-uniform refinement at 3.5 Å resolution. Masked 3D classification of this region (10 classes, target resolution 12 Å) resulted in the selection of 39,555 particles that were unmasked and refined using non-uniform refinement, resulting in a reconstruction at 4.6 Å resolution at a pixel size of 2.27 Å (Figure S1K). The improved resolution in this region resulted in its assignment as the Pri2_CTD_.
Strategy 3: Classes representing 100,178 particles with strong density for both the Mcm2-7 C-tier in conformation II and Pol α-primase regardless of Ctf4 occupancy. These data were refined using non-uniform refinement in cryoSPARC, using a pixel size of 0.86 Å, to a resolution of 3.5 Å. Signal subtraction and masked local refinement was carried out in cryoSPARC using a pixel size of 0.86 Å, as described in Strategy 1, focussing on the Mcm2-7 C-tier yielding a reconstruction at 3.2 Å resolution (Figure S1F).
Strategy 4: Classes representing 84,350 particles with strong density for Tof1-Csm3/dsDNA and Pol α-primase regardless of Ctf4 occupancy. These data were refined using non-uniform refinement in cryoSPARC, using a pixel size of 0.86 Å, to a resolution of 3.4 Å. Signal subtraction and masked local refinement was carried out in cryoSPARC using a pixel size of 0.86 Å, as described in Strategy 1, focussing on Tof1-Csm3/DNA yielding a reconstruction at 3.9 Å resolution (Figure S1G).

In order to enrich for particles in which the Pri2_CTD_ was well resolved, the 588,597 particle subset initially imported into cryoSPARC following processing in RELION was re-processed using a 3D reference obtained from refinement of the 39,555 particle subset containing density for the Pri2_CTD_. Four rounds of iterative heterogeneous refinement were carried out using four 3D references containing Pri2_CTD_ domain density resulting in an 87,540 particle subset. The non-Pol α-primase density was subtracted from these particles and 3D classification without alignment was carried out within a mask encompassing all of the visible regions of Pol α-primase. Classes were selected based on clear Pri2_CTD_ domain density resulting in a 45,111 particle subset. The corresponding un-subtracted particles were then refined using non-uniform refinement to a resolution of 4.6 Å prior to being further classified using 3D variability analysis^49^ using 3 modes and a filter resolution of 9 Å. The results were displayed using clustering analysis using 6 clusters and a filter resolution of 9 Å. Inspection of the resulting reconstructions revealed the presence of additional density corresponding to the Pol1_exo/cat_ domain. An additional round of 3D variability analysis was carried out using the same parameters with a new mask additionally encompassing the Pol1_exo/cat_ domain density. This procedure identified a subset of 9,633 particles which were then refined to a resolution of 4.6 Å that displayed density for both the Pri2_CTD_ and the Pol1_exo/cat_ domains (Figures S3G and S3H).

##### Human replisome + Pol α-primase + 60 nucleotide 5′-flap DNA fork

The data processing pipeline is illustrated by the schematic in Figure S6C. 7,355 39-fraction movies were aligned and dose-weighted (1.22 e-/Å^2^/fraction, 5 x 5 patches, 150 Å^2^ B-factor) using RELION’s implementation of a MotionCor2-like program.^69^ CTF parameters were estimated using CTFFIND-4.1^70^ and 44 poor-quality micrographs excluded from future processing. Particles were picked using RELION’s Laplacian-of-Gaussian (LoG) function providing a minimum diameter of 180 Å and maximum of 330 Å. 1,535,548 particles were extracted using a box size of 380 Å. During extraction the data were down-sampled to a pixel size of 3.78 Å/pixel. Two successive rounds of RELION 3D classification (regularisation parameter, T = 4), using 6 classes, were carried out using a previously obtained map of the human core replisome as a 3D reference (EMD-13375).^41^ Class selection was based upon the presence of secondary structure features within CMG. 3D classification was performed with a 250 Å diameter circular mask to focus classification on CMG. A resulting 550,340 particles were subsequently refined using 3D auto-refinement in RELION generating a reconstruction at 7.6 Å resolution following postprocessing. These data were submitted for per-particle motion correction using dataset-trained particle polishing in RELION^72^ using a pixel size 0.86 Å in a 450 Å box. RELION CTF-refinement^64^ (beamtilt and trefoil correction, anisotropic magnification correction, and per-particle defocus and astigmatism CTF correction) was then carried out and the data refined to an improved resolution of 3.6 Å following postprocessing in RELION. A further round of 3D classification was carried out with a dilated circular mask of 380 Å and classes with significant Pol α-primase density representing 359,677 particles selected for subclassification. Signal subtraction and masked 3D classification without alignment was carried out in RELION as described in the budding yeast data processing methods to enrich for replisomes stably associated with Pol α-primase using a mask covering both Pol α-primase and the MCM3 N-terminal helical domain.

3D classes in which Pol α-primase adopted the previously reported autoinhibited primosome conformation (PDB:5EXR)^46^ were selected, representing 74,940 particles. Following reversion to the original non-subtracted particles.star file these data were imported into cryoSPARC and refined using non-uniform refinement^73^ to 3.6 Å resolution (Figure S7G). Density for DNA was not observed within this reconstruction.

Returning to the previous masked 3D classification without alignment in RELION, classes comprising 174,696 particles were selected in which Pol α-primase adopted a conformation distinct from that of the primosome. Following reversion to the original non-subtracted particles.star file these data were refined via 3D auto-refinement in RELION and postprocessed to a resolution of 4.1 Å. These data were then submitted to an additional round of particle polishing and CTF-refinement in RELION using the same parameters as the previous round. Particles were imported into cryoSPARC and a consensus refinement carried out using non-uniform refinement generating a reconstruction at a resolution of 3.4 Å (Figure S6E). Local refinements were carried out in cryoSPARC, as described in the budding yeast data processing methods, for regions encompassing both TIMELESS-TIPIN/DNA (Figure S6F) and the MCM2-7 C-tier (Figure S6H) resulting in reconstructions at 4.1 Å and 3.7 Å respectively.

In order to improve the quality of the AND-1 density, particle subtraction followed by masked 3D classification without alignment was carried out as described in the budding yeast data processing methods. 3D classification was carried out on the total dataset imported into cryoSPARC, in a conformation distinct from the primosome, focussing on the AND-1/CDC45/GINS region of the map. 3D classes were selected, consisting of 63,393 particles, based on the presence of continuous strong AND-1 density. These data were then locally refined to generate a reconstruction at 3.3 Å resolution (Figure S6G).

In order to improve the quality of the Pol α-primase density, particle subtraction followed by masked 3D classification without alignment was carried out using a mask covering the POLA1_CTD_/POLA2/PRIM1/PRIM2_NTD_ region of the map. 3D classes were selected, consisting of 148,103 particles which were locally refined to generate a reconstruction at 4.4 Å resolution (Figure S6I). A single 3D class was selected from this procedure, containing 23,758 particles, with particularly strong PRIM1 density. The non-subtracted particles comprising this class were subjected to consensus non-uniform refinement generating a reconstruction at 4.3 Å resolution.

#### Human replisome + Pol α-primase + 15 nucleotide 5′-flap DNA fork

The data processing pipeline is illustrated by the schematic in Figure S8D. 6,718 39-fraction movies were aligned and dose-weighted (2.27 e-/Å^2^/fraction, 5 x 5 patches, 150 Å^2^ B-factor) using RELION’s implementation of a MotionCor2-like program.^69^ CTF parameters were estimated using CTFFIND-4.1^70^ and 109 poor-quality micrographs excluded from future processing. Particles were picked using Gautomatch v0.56 (https://www2.mrc-lmb.cam.ac.uk/research/locally-developed-software/zhang-software/#gauto) leading to extraction of 724,557 particles using a box size of 380 Å and pixel size of 4.28 Å/pixel (raw pixel size 1.07 Å/pixel). Two successive rounds of RELION 3D classification (regularisation parameter, T = 4), using 6 classes, were carried out using a previously obtained map of the human core replisome as a 3D reference (EMD-13375).^41^ Class selection was based upon the presence of secondary structure features within CMG. 3D classification was performed with a 250 Å diameter circular mask to focus classification on CMG. A resulting 584,362 particles were subsequently refined using 3D auto-refinement in RELION generating a reconstruction at 8.8 Å resolution. These data were submitted for per-particle motion correction using dataset-trained particle polishing in RELION^72^ using a pixel size 1.02 Å in a 450 Å box. RELION CTF-refinement^64^ (beamtilt and trefoil correction, anisotropic magnification correction, and per-particle defocus and astigmatism CTF correction) was then carried out and the data refined to an improved resolution of 3.4 Å following postprocessing in RELION. A further round of 3D classification was carried out with a dilated circular mask of 380 Å. 28,202 particles in 3D classes lacking DNA were imported into cryoSPARC and refined using non-uniform refinement to 3.6 Å resolution. 492,011 particles in 3D classes with significant Pol α-primase and DNA density were selected for further subclassification. Signal subtraction and masked 3D classification without alignment was carried out in RELION as described in the budding yeast data processing methods to enrich for replisomes stably associated with Pol α-primase using a mask covering both Pol α-primase and the MCM3 N-terminal helical domain. The signal subtraction followed by 3D classification without alignment procedure was iterated resulting in the selection of 258,339 particles in 3D classes with strong Pol α-primase density. These data were imported into cryoSPARC and refined using non-uniform refinement to 3.3 Å resolution.

#### Cryo-EM model building

##### Budding yeast replisome + Pol α-primase + 60 nucleotide 5′-flap DNA fork

To begin model building, structures of the budding yeast core replisome (PDB:6SKL)^30^ with the MCM C-tier removed and the MCM C-tier in conformation II (PDB:6SKO),^30^ were rigid body docked into the cryo-EM map of the budding yeast replisome fully engaged by Pol α-primase in the absence of Ctf4 at 4.6 Å resolution (binned pixel size of 2.27 Å) using ChimeraX.^74^ The atomic model for Ctf4 contained within 6SKO was manually removed at this stage. The structure of the human primosome (PDB:5EXR)^46^ was then rigid body docked into the region of the cryo-EM map that remained unassigned, guided by the presence of secondary structure features within density for the Pri2_NTD_. Inspection of the fit-to-density following 5EXR docking revealed a lack of strong cryo-EM density corresponding to both the POLA1 exonuclease and catalytic domains and the PRIM2_CTD_, therefore these were subsequently removed from the model. The quality of the fit-to-density for the remaining human Pol α-primase model was then improved via manual manipulation followed by automated docking for both the PRIM1 and PRIM2_NTD_ domains and the POLA1_CTD_/POLA2 module respectively. The positions of these human Pol α-primase subunits provided a reference to which the AlphaFold^50^ models for budding yeast Pri2_NTD_ (residues S44-T299), PRIM1 (residues S12-D402) and the crystal structure the Pol1_CTD_/Pol12 dimer (PDB:3FLO) were aligned prior to being rigid body fit into the density. An AlphaFold multimer^75^ model for the Pol12 (residues 203-705) / Pol1_CTD_ (residues 1260-1468) complex was aligned to the Pol12 subunit of 3FLO, rigid-body fit into the cryo-EM density and the most N-terminal residue of Pol1 trimmed to I1271. The Pol1 C-terminus was remodelled based on an AlphaFold-Multimer^75^ result indicating complex formation between the Pol1 C-terminus and Pri2_NTD_, in an analogous fashion to the POLA1_C-term_ in the primosome structure 5EXR.

The quality of the fit for the model into the fully engaged cryo-EM map lacking Ctf4 at 4.6 Å resolution was optimised via an all-atom simulation in ISOLDE,^27^ using adaptive distance restraints for the dsDNA model (kappa=100). The resulting model was further refined using Phenix^76^ real-space-refine, utilising the input model as a reference to generate restraints with sigma=0.1 and global minimisation with nonbonded_weight=2000 and weight=0.5. Regions of the model that fit poorly to the density were the manually refined in Coot^77^ using the local refinement and regularisation tools incorporating stereochemical restraints. The model for the Mcm2-ZnF (residues 338–378) was truncated due to the absence of well resolved density in this region of the map.

At this stage in the modelling process, regions of cryo-EM density that remained unmodelled were identified for further analysis to determine their identity. Density for a small four-helical bundle bound to the Mcm3 AAA^+^ domain was assigned as the flexibly tethered Pol12_NTD_ (residues M1-I79). An AlphaFold-Multimer prediction for the Pol12_NTD_ interacting with the AAA^+^ domain of Mcm3 was used to dock the Pol12_NTD_ into the cryo-EM density.

Two regions of disconnected helical density were identified in analogous positions to CLASPIN in the core human replisome (PDB:7PFO).^41^ This led us to speculate that these represented regions of the budding yeast homologue of CLASPIN, Mrc1. Furthermore, AlphaFold-Multimer modelling predicted multiple high-confidence interactions between Mrc1 and the replisome. Predictions were validated by the presence of corresponding side-chain density for Mrc1, present in the un-binned cryo-EM reconstruction of the fully engaged Pol α-primase complex at 3.5 Å resolution. Mrc1 residues S339-K323 interact with the α-solenoid of Tof1, whilst D468-Q483 contacts Mcm2 in the N-tier and the Tof1 N-terminus. An additional region of disconnected density, on the same side of the replisome to the two modelled regions of Mrc1, was also assigned to Mrc1 based on AlphaFold-Multimer prediction and the presence of clear side-chain density. Alphafold-Multimer predicts an interaction between Mrc1 residues L815-E858 spanning both Cdc45 and the Mcm2 N-tier, for which there is cryo-EM density present in the fully engaged reconstruction at 4.6 Å resolution. However, there is only cryo-EM density of sufficient resolution to enable unambiguous assignment, in the 3.5 Å resolution fully engaged map, for Mrc1 residues N842-E858, therefore these are the only residues deposited in the final model for this particular Mrc1 interface.

A region of unmodelled density bound to the GINS subunit Psf2 was ascribed to Pri2_Nterm_ (residues M1-S5). Assignment was based upon the close proximity of the otherwise most N-terminal modelled residue of Pri2 (S44) and the presence of continuous density between S5-S44 present at low map thresholds. Clear side chain density was present for residues M1-Q4. As the second residue in Pri2 is a large phenylalanine residue the first methionine will not be removed^78^ and is likely to be acetylated.^79^

At this stage the model was inspected residue-by-residue in Coot,^77^ docked into the highest resolution cryo-EM map (consensus and focused refinements) for the corresponding region of the model. Focussed refinements were rigid-body docked into the consensus and resampled onto the same origin. The model was manually refined against the map in Coot^77^ and both backbone Ramachandran and rotamer outliers corrected. A final global run in ISOLDE^80^ was carried out to minimise the clash-score using distance and torsion restraints prior to Phenix^76^ real-space-refinement using the same restraints as described above. Model validation was carried out using the Molprobity server,^81^ Phenix^76^ validation and the wwPDB OneDep validation server.

In order to model the budding yeast replisome fully engaged by Pol α-primase in the presence of Ctf4, the structure of trimeric Ctf4 (PDB:6SKL)^30^ was rigid body docked into the corresponding density. The fit-to-density was subsequently refined via local ISOLDE^80^ simulation followed by Phenix^76^ real-space-refinement and manual adjustment in Coot.^77^ Inspection of the locally refined Ctf4/GINS/Cdc45 cryo-EM map revealed the presence of unmodelled density bound to the helical bundles of two Ctf4 monomers in the correct position to accommodate the Pol1 CIP-box (a.a. F140-S149).^29^ The Pol1 CIP box was subsequently modelled into each discrete density based on the crystal structure of the Ctf4_CTD_-Pol1 CIP-box (PDB: 4C93).^29^ It was not possible to sub-classify the Ctf4 density to generate reconstructions with only one-site occupied at any time, therefore two models were deposited to the PDB with the Pol1 CIP-box interacting with a different monomer of Ctf4 in each.

##### Human replisome + Pol α-primase + 60 nucleotide 5′-flap DNA fork

A previously determined structure of the core human replisome (PDB: 7PFO)^41^ was rigid body docked into the locally filtered cryo-EM map of the human replisome fully engaged by Pol α-primase on a DNA fork containing a 60 nucleotide 5ʹ-flap at 3.4 Å resolution using ChimeraX.^74^ The atomic model for Pol ε contained within 7PFO^41^ was manually removed at this stage, as were MCM2 residues 324-368 which comprise its zinc-finger motif due to the lack of corresponding density. Using the same strategy employed for the modelling of the budding yeast Pol α-primase-replisome structure, a previously determined model for the human primosome (PDB:5EXR) was rigid body docked into the remaining unassigned density. The 5EXR model was then manually edited to remove both the POLA1 exonuclease and catalytic domains and the PRIM2_CTD_ due to a lack of corresponding cryo-EM density. The fit-to-density for the Pol α-primase subunits was improved by docking each module: PRIM1, PRIM2 and the POLA1_CTD_/POLA2 dimer, individually as a rigid body. AlphaFold multimer^75^ structure predications for the PRIM1- PRIM2_NTD_ complex and the POLA2-POLA1_CTD_ complex were aligned to the corresponding subunits derived from 5EXR, replacing the crystal structure subunits. Using this strategy atomic models were generated for the following regions of sequence: PRIM2 residues Q17-H252, PRIM1 residues M9-T349 and T386-G408, POLA1 residues Q1279-G1445 and E1448-C1458 and POLA2 residues I96-T114 and V170-I598.

The quality of the fit-to-density was optimised via an all-atom simulation in ISOLDE,^80^ using adaptive distance restraints for the dsDNA model (kappa=100). The resulting model was further refined using Phenix^76^ real-space-refine, utilising the input model as a reference to generate restraints with sigma=0.1 and global minimisation with nonbonded_weight=2000 and weight=0.5. Regions of the model that fit poorly to the density were then manually refined in Coot^77^ using the local refinement and regularisation tools incorporating stereochemical restraints.

Inspection of the cryo-EM density following the modelling procedure outlined revealed a short region of unmodelled helical density adjacent to the PSF1 subunit. AlphaFold-Multimer analysis predicted a helix within the flexible N-terminus of POLA2, residues I96-T114, to bind at the location of the unmodelled density. Furthermore, clear side chain density for POLA2 Y113 and L109 corroborated the prediction in addition to stereo-chemically favourable contacts formed. This region of POLA2 was subsequently incorporated into the final model.

A region of unmodelled density bound to the GINS subunit PSF2 was assigned to the PRIM2_Nterm_, residues M1-G5, and incorporated into the final model. Assignment was based upon the close proximity of the otherwise most N-terminal modelled residue of Pri2, Q17, and the presence of continuous density between G5-Q17 present at low map thresholds. Clear side chain density was present for residues M1-S4. Furthermore, the PRIM2_Nterm_ – PSF2 contact was predicted by AlphaFold-Multimer at high confidence.

At this stage the model was inspected residue-by-residue in Coot,^77^ docked into the highest resolution cryo-EM map (consensus and focused refinements) for the corresponding region of the model. The model was manually refined against the map in Coot^77^ and both backbone Ramachandran and rotamer outliers corrected, followed by real-space refinement in both ISOLDE^80^ and Phenix.^76^ Each focussed refinement was rigid-body docked into the consensus refinement (EMD-15341) and resampled onto the same map origin. Only density at the AND-1 – CDC45/GINS interface was used to align the CDC45/GINS/AND-1 local refinement (EMD-15342) to the consensus (EMD-15341). However, due to subtle differences between the density at this interface between the local and consensus refinements it was not possible to build a model that perfectly satisfied both maps. A final global run in ISOLDE^80^ including distance and torsion restraints was carried out to minimise the clash-score prior to Phenix^76^ real-space-refinement using the same restraints as described above into the consensus refinement with additional reference model restraints. Model validation was carried out using the Molprobity server,^81^ Phenix^76^ validation and the wwPDB OneDep validation server. Following this procedure, the model for CLASPIN residues E299-E310 was removed due to the lack of corresponding cryo-EM density.

#### Multiple sequence alignments

Amino acid sequences were retrieved from UniProt and protein sequence alignments carried out using Clustal Omega.^82^ Alignments were rendered using ESPript3.0 (http://espript.ibcp.fr).^83^

#### AlphaFold and AlphaFold-Multimer

AlphaFold models were obtained from the AlphaFold Protein Structure Database^50^ (https://alphafold.ebi.ac.uk). AlphaFold-Multimer structure predictions were determined using the Colabfold^84^ implementation (version 1.2.0) using the following parameters: num_models=5, num_recycles=3, use_amber=false, use_templates=false. Colabfold sequence alignments were performed using Mmseqs2. Below is a description of the AlphaFold-Multimer input sequences, including protein names and the residue ranges included as input: Pri2_NTD_ (44-299) / Pol1_C-term_ (1140-1468); Pol12_N-term_ (1-74) / Mcm3 AAA^+^ domain (337-750); Pol12 (203-705) / Pol1_CTD_ (1260-1468); Mrc1_site #1_ (300-350) / Tof1 (1-779); Mrc1_site #2_ (420-550) / Tof1 (1-300) / Mcm2 (150-458); Mrc1_site #3_ (600-1000) / Mcm2 (150-458) / Cdc45 (1-468); Pri2_Nterm_ (1-30) / Psf2 (1-213); PRIM2_Nterm_ (1-14) / PSF2 (1-185); POLA2 (1-200) / PSF1 (1-196) / SLD5 (1-223); POLA2 (100-598) / POLA1_CTD_ (1260-1458); PRIM1 (1-420) / Pri2 (1-509).

#### Budding yeast primase-polymerase assay on M13mp18 ssDNA

Reactions (10 μl) were performed at 30^o^C in a buffer containing: 25 mM HEPES-KOH (pH 7.6); 100 mM potassium glutamate; 10 mM Mg(OAc)_2_; 1 mM DTT; 0.01 % NP-40 substitute (NP-40-S) (Roche #11754599001), 0.1 mg/ml bovine serum albumin (BSA); 0.5 nM M13mp18 ssDNA (NEB #N4040S); 30 μM dC, dG, dT, dATP; 200 μM G, C, UTP; 33 nM α-[^32^P]-dCTP (Hartmann Analytic #SCP-205); 3 mM ATP; 40 nM RPA; 20 nM Pol α-primase. RPA was prebound to the ssDNA template for 10 min and then reactions were started by addition of Pol α-primase. Reactions were quenched with 10 μl 100 mM EDTA and unincorporated radiolabelled nucleotide was removed using a Microspin G-50 column (Cytiva). 1/10 volume loading buffer (10% w/v sucrose; 500 mM NaOH; ∼0.25% w/v xylene cyanol) was added to the sample before analysis on 0.7% alkaline agarose gels run in 30 mM NaOH, 2 mM EDTA for 16 hours at 25 volts. After electrophoresis gels were incubated at 4^o^C in 5% trichloroacetic acid for 30 min with one buffer change after 15 min. The gel was then incubated in 500 mM Tris-Cl (pH 8) for 15 mins before being dried at 75^o^C under vacuum onto Whatman 3 MM paper (Cytiva). The dried gel was imaged using a BAS-IP MS phosphor screen (Cytiva) and an Amersham Typhoon phosphorimager and on Amersham Hyperfilm MP (Cytiva).

#### Origin-dependent budding yeast DNA replication assay

Origin-dependent budding yeast DNA replication reactions were based on a previously established protocol.^1^^,^^7^^,^^37^^,^^38^ First, Mcm2-7 double hexamers were loaded onto the DNA template in an MCM loading reaction (10-40 μl) containing: 25 mM HEPES-KOH (pH 7.6); 100 mM potassium glutamate; 10 mM Mg(OAc)_2_; 1 mM DTT; 0.01 % NP-40-S, 0.1 mg/ml bovine serum albumin (BSA); 40 mM KCl, 3 mM ATP, 3 nM SphI-linearised vVA20^1^; 75 nM Cdt1-Mcm2-7, 40 nM Cdc6; 20 nM ORC; 25 nM DDK. The reaction was incubated at 24^o^C for 10 mins, at which point S-CDK was added to a final concentration of 80 nM and incubation continued at 24^o^C for a further 5 min. The MCM loading reaction was diluted 4-fold into a replication buffer to give a final replication reaction buffer consisting of: 25 mM HEPES-KOH (pH 7.6); 250 mM potassium glutamate; 1 mM DTT; 0.01 % NP-40-S, 0.1 mg/ml BSA; 10 mM KCl, 3 mM ATP, 30 μM dC, dG, dT, dATP; 200 μM G, C, UTP, 33 nM α-[32P]-dCTP (Hartmann Analytic #SCP-205); 0.75 nM SphI-linearised vVA20; 18.75 nM Cdt1-Mcm2-7, 10 nM Cdc6; 5 nM ORC; 6.25 nM DDK; 20 nM S-CDK. Pol α-primase was added to a final concentration of 10 nM and reactions (10 μl) were equilibrated at 30^o^C. DNA replication was initiated by addition of replication proteins to final concentrations of: 30 nM Dpb11; 100 nM GINS; 30 nM Cdc45; 10 nM Mcm10; 20 nM Pol ε; 20 nM Ctf4; 100 nM RPA; 20 nM RFC; 20 nM Tof1-Csm3; 20 nM PCNA; 10 nM Pol δ; 12.5 nM Sld3/7; 20 nM Sld2; 10 nM Mrc1. Reactions were incubated at 30^o^C for 20 mins before quenching, processing and analysis as described for budding yeast primase-polymerase assays on M13mp18 ssDNA.

#### Budding yeast tetrad dissection

Details of yeast strains can be found in Table S4. Diploid yeast cells were patched onto sporulation plates (0.25% yeast extract; 0.1% glucose; 1.5% potassium acetate; 2% agar; 5 μg/ml Arginine, 10 μg/ml Adenine, 10 μg/ml Uracil; 5 μg/ml Histidine; 5 μg/ml Leucine; 5 μg/ml Lysine; 5 μg/ml Tryptophan, 2 μg/ml Tyrosine; 25 μg/ml Phenylalanine; 5 μg/ml Methionine; 1 μg/ml Proline) and incubated for 3-5 days at 30^o^C. Sporulated cells were picked using sterile toothpicks and resuspended in 75 μl sterile Milli-Q water and 5 μl β-Glucuronidase (Sigma #G7017). Following incubation at room temperature for 15-20 min, 150 μl sterile Milli-Q water was added and 10 μl of this mix was streaked onto YPD plates (1.1% yeast extract; 2.2% peptone; 2% glucose; 55 μg/ml Adenine; 2.5% agar). Tetrads were dissected using a micromanipulator (Singer Instruments) and the resulting cells were grown for 3 days at 25^o^C. Plates were imaged on an Epson Perfection V850 Pro scanner.

Cells were genotyped by analysing growth on appropriate selective plates or, when two alleles were associated with the same auxotrophic marker, by PCR from purified genomic DNA. For Pri2, primers JY105 and JY609 were used to generate a 608 bp PCR product. Products were digested with BsaHI, which digests the *Pri2-AAA* allele but not the wild type. For Mcm3, primers JY604 and VA212 were used to generate a 728 bp PCR product. Products were digested with FspI, which digests the wild type allele but not *Mcm3-CR*. Alternatively, primers JY370 / JY491 were used to amplify a region of the Mcm3 allele linked to the Ura3 marker. Primers JY370 / JY104 were used to amplify a region of the Pri2 allele linked to the Ura3 marker.

#### Human primase-polymerase assay on M13mp18 ssDNA

Reactions were performed at 37°C in a buffer containing 25 mM HEPES-KOH (pH 7.6), 100 mM potassium glutamate, 0.01% NP-40-S, 1 mM DTT, 10 mM Mg(OAc)_2_, 0.1 mg/ml BSA, 5 mM ATP, 200 μM CTP, GTP, UTP, 30 μM dATP, dCTP, dGTP, dTTP, and 33 nM α-[^32^P]-dCTP. 0.5 nM M13mp18 single-stranded DNA (New England Biolabs) was pre-incubated with 40 nM RPA for 10 min. Reactions were initiated by the addition of 20 nM Pol α-primase. After 20 min, reactions were quenched by addition of EDTA to 50 mM. Post reaction processing and analysis were performed as described for budding yeast primase-polymerase assays on M13mp18 ssDNA.

#### Human DNA replication assays

Replication reactions were performed on a 9.7 kbp forked linear DNA template (made from SapI-linearized ZN3 plasmid) as previously described^27^ with minor modifications. Reactions were conducted at 37°C in a replication buffer consisting of 25 mM HEPES-KOH (pH 7.6), 0.01% NP-40-S, 100 mM potassium glutamate, 1 mM DTT, 10 mM Mg(OAc)_2_ and 0.1 mg/ml BSA. The final concentration of potassium glutamate in reactions was increased to 230 mM when replication was initiated. Protein and nucleotide concentrations in the final reactions were: 25 nM CMG, 15 nM Pol ε, 20 nM RFC, 7 nM Pol δ, 20 nM PCNA, 20 nM AND-1, 10 nM Pol α-primase, 100 nM RPA, 20 nM CLASPIN, 20 nM TIMELESS–TIPIN, 20 nM CTF18–RFC, 4 mM ATP, 30 μM dC/dT/dG/dATP, 200 μM C/G/UTP and 33 nM α-[^32^P]-dCTP. Reactions were set up as follows: 2 nM linear forked DNA template was pre-incubated with 50 nM CMG for 10 min in replication buffer. The reaction was diluted twofold by the addition of replication buffer containing 60 μM dA/dCTP, PCNA, Pol ε, Pol α-primase, Pol δ, CLASPIN, TIMELESS–TIPIN, AND-1 and CTF18–RFC. Replication was initiated by addition of a 10X solution containing ATP, RFC, dTTP, dGTP, GTP, CTP, UTP, α-[^32^P]-dCTP and RPA. Reactions were stopped by addition of 50 mM EDTA and unincorporated α-[^32^P]-dCTP was removed with illustra MicroSpin G-50 columns (GE Healthcare). Reactions were run on 0.7% alkaline agarose gels in 2 mM EDTA and 30 mM NaOH and for 16 h at 26 V. Post reaction processing and analysis were performed as described for budding yeast primase-polymerase assays on M13mp18 ssDNA.

### Quantification and statistical analysis

No quantification or statistical analysis were performed in this manuscript.

## Data Availability

• Cryo-EM density maps used in model building have been deposited in the Electron Microscopy Data Bank (EMDB) https://www.ebi.ac.uk/pdbe/emdb, under the following accession numbers: ***Budding yeast.*** EMD-16320 (binned), EMD-16322 (un-binned), Pol α-primase associated replisome consensus refinement in the absence of Ctf4, 60 nucleotide 5ʹ-flap DNA fork. EMD-15304, Tof1-Csm3 local refinement. EMD-15305, Mcm2-7 C-tier local refinement, conformation II. EMD-15306, Pol12, Pol1_CTD_, Pri2_NTD_ local refinement. EMD-16885, Pol12, Pol1CTD local refinement. EMD-15309 (binned), EMD-15902 (un-binned), Pol α-primase associated replisome consensus refinement in the presence of Ctf4, 60 nucleotide 5ʹ-flap DNA fork. EMD-15310, Ctf4 local refinement. EMD-16247, Pri1, Pri2_CTD_ local refinement. EMD-16248, Pol α-primase associated replisome consensus refinement including density for the Pol1_exo/cat_ and Pri2_CTD_ domains. EMD-15924 (binned), EMD-15303 (un-binned), Pol α-primase associated replisome consensus refinement with continuous density for the lagging strand DNA template extending towards the Pri1 active site and density for the Pri1_CTD_, 60 nucleotide 5ʹ-flap DNA fork. EMD-16323, consensus refinement of Pol α-primase associated with the replisome only via the Pri2:Mcm5_ZnF_ and Pri2_Nterm_:Psf2 interfaces, 60 nucleotide 5ʹ-flap DNA fork. ***Human.*** EMD-15341, Pol α-primase associated replisome un-binned consensus refinement, 60 nucleotide 5ʹ-flap DNA fork. EMD-15342, AND-1 local refinement. EMD-15340, MCM2-7 C-tier local refinement. EMD-15351, PRIM1, POLA2, PolA1_CTD_, Pri2_NTD_ local refinement. EMD-15356, TIMELESS-TIPIN local refinement. EMD-15904. EMD-15349, Pol α-primase associated replisome binned consensus refinement, strong PRIM1 density. composite map assembled from EMD-15342: 15341:15340:15349:15351:15356. EMD-15918, Pol α-primase associated replisome consensus refinement, not engaged on DNA derived from dataset including a 15 nucleotide 5ʹ-flap DNA fork. EMD-15923, Pol α-primase associated replisome consensus refinement, not engaged on DNA derived from dataset including a 60 nucleotide 5ʹ-flap DNA fork. EMD-15922, Pol α-primase associated replisome consensus refinement, engaged on a 15 nucleotide 5ʹ-flap DNA fork. Atomic coordinates have been deposited in the Protein Data Bank (PDB), http://www.pdb.org, with the following accession numbers: **Budding yeast.** 8B9C - Pol α-primase associated replisome in the absence of Ctf4. 8B9A - Pol α-primase associated replisome in the presence of Ctf4, CIP box site #1. 8B9B - Pol α-primase associated replisome in the presence of Ctf4, CIP box site #2. ***Human.*** 8B9D – Pol α-primase associated replisome. Unprocessed images of the data featured in this manuscript have been deposited at Mendeley Data and are publicly available as of the date of publication (https://doi.org/10.17632/n2wm36mrmw.1). • This study does not report any original code. • Any additional information required to reanalyse the data reported in this paper is available from the lead contact upon request.

## Supplemental information

Document S1. Figures S1–S9, Tables S1–S4, and supplemental references

Document S2. Article plus supplemental information
