# Supplementary material for: How Pol α-primase is targeted to replisomes to prime eukaryotic DNA replication
Source: Mol Cell. 2023 Aug 17;83(16):2911–2924.e16. doi: 10.1016/j.molcel.2023.06.035 (PMC10501992; doi:10.1016/j.molcel.2023.06.035)
Supplement: Document S1. Figures S1–S9, Tables S1–S4, and supplemental references [file mmc1.pdf]

Molecular Cell, Volume *83*

Supplemental information

How Pol α-primase is targeted to replisomes to prime eukaryotic DNA replication

Morgan L. Jones, Valentina Aria, Yasemin Baris, and Joseph T.P. Yeeles

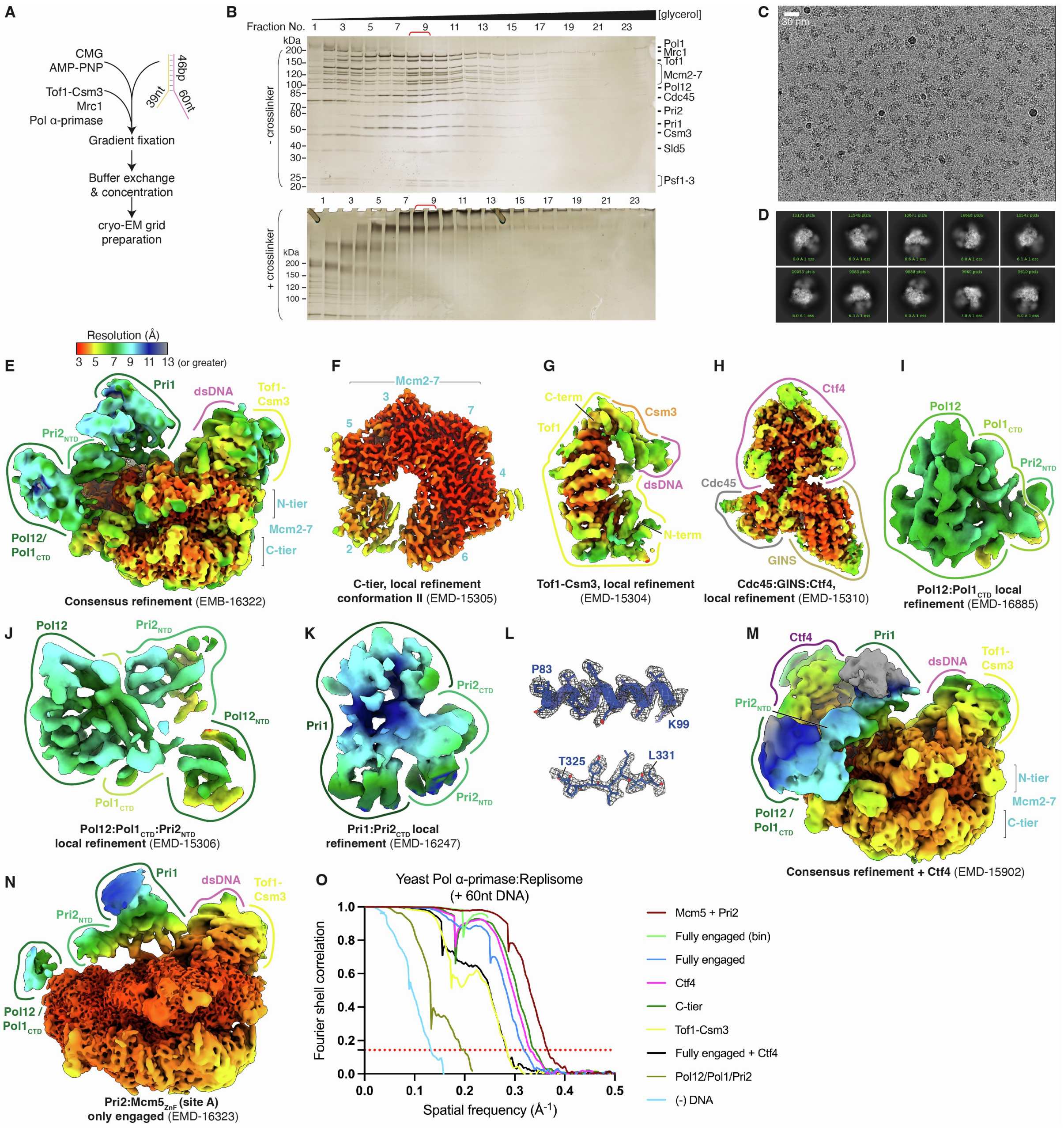

Figure S1. Cryo-EM analysis of a budding yeast replisome containing Pol α-primase assembled on forked DNA with a 60 nt 5'-flap, related to Figure 1.

(A) Schematic outlining the *in vitro* reconstitution method used to generate complexes for cryo-EM. Cartoon of the forked DNA is shown with the leading-strand template in orange and the lagging-strand template in pink.

(B) Silver-stained SDS-PAGE gels analysing 100 µl fractions taken across 10-30% glycerol gradients, either in the absence (top) or presence (bottom) of crosslinking agents. Fractions 8-9 that were used for cryo-EM sample preparation are indicated with red brackets.

(C) Representative cryo-EM micrograph, 30 nm scale bar inset, obtained using a K3 direct electron detector (Gatan) at a nominal pixel size of 0.86 Å/pixel.

(D) Representative 2D class averages with corresponding particle numbers, mask diameter 360 Å, obtained using cryoSPARC-3 2D classification.

(E-K) Cryo-EM density maps for reconstructions relevant to model building in the absence of Ctf4, coloured by local resolution according to inset key in **(E)**. Local resolution was calculated using RELION for the reconstruction in panel **(E)**, and ResMap[S1] for panels **(F-K)**.

(L) Representative cryo-EM density (mesh) for an α-helix (top) at a local resolution of 3.1 Å and a β-strand at a local resolution of 2.9 Å (bottom) in Mcm5 (blue ribbon model).

(M) Cryo-EM reconstruction, obtained via consensus refinement, for particles containing Ctf4, coloured by local resolution, calculated using RELION, according to inset key in panel **(E)**.

(N) Cryo-EM reconstruction obtained via consensus refinement in which only the $Pri2_{NTD}$:Mcm5 (site a) and $Pri2_{Nterm}$:Psf2 (site d) interfaces are simultaneously occupied, coloured by local resolution, calculated using RELION, according to inset key in **(E).**

(O) Fourier shell correlation (FSC) graph describing the maps used in model building. Resolution was calculated using the FSC=0.143 cut-off with values reported in Figure S2.

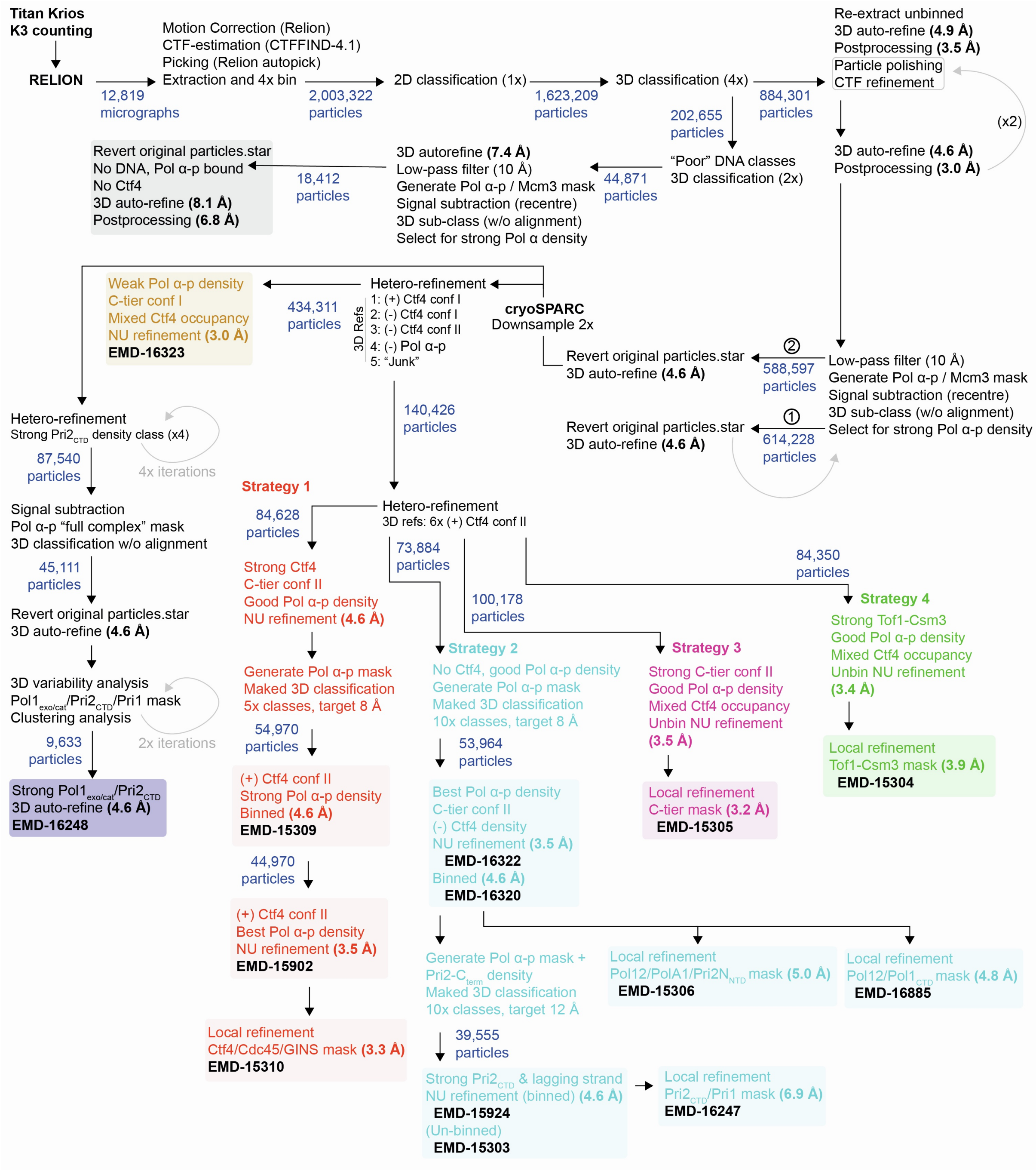

Figure S2. Cryo-EM data processing pipeline for the budding yeast replisome assembled on forked DNA with a 60 nt 5'-flap, related to Figure 1.

Abbreviations: NU, non-uniform; w/o, without; Pol α-p, Pol α-primase. Shaded text denotes cryo-EM maps deposited in the EMDB.

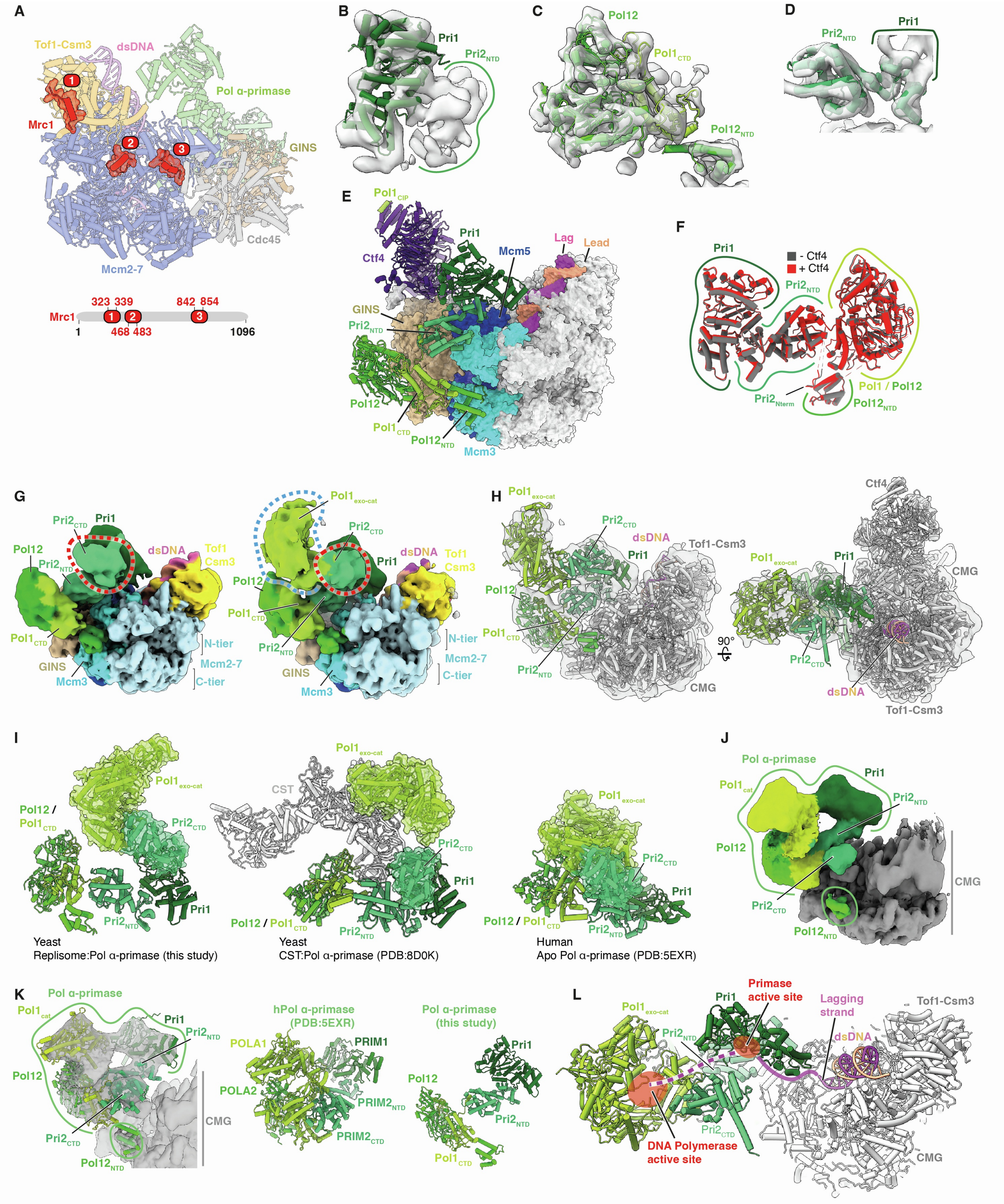

Figure S3. Structure of a budding yeast replisome containing Pol α-primase, related to Figure 1.

(A) Model highlighting the regions of Mrc1 (coloured red with surface rendering) for which an atomic model could be built. Modelled regions are numbered 1-3 (indicated by red circles) and their associated sequence coverage denoted by the sequence diagram.

(B-D) Atomic models displayed with cryo-EM density for Pri1 (**B**), Pol12-Pol1$_{CTD}$-Pol12$_{NTD}$ (**C**) and Pri2$_{NTD}$ (**D**). Cryo-EM density corresponds to local refinements shown in Figures S1I-S1K.

(E) Atomic model of the budding yeast Pol α-primase associated replisome containing Ctf4. Regions of CMG that physically interact with Pol α-primase are coloured.

(F) Comparison between the structures of Pol α-primase in replisomes lacking (grey) or containing (red) Ctf4, aligned on $Pri2_{NTD}$.

(G) Cryo-EM reconstructions, obtained using a binned pixel size of 2.3 Å, containing additional density of the appropriate shape and volume to accommodate $Pri2_{CTD}$ and $Pol1_{exo\text{-}cat}$. (Left) Reconstruction containing density for $Pri2_{CTD}$ (circled red). (Right) Reconstruction containing density for both $Pri2_{CTD}$ (circled red) and $Pol1_{exo\text{-}cat}$ (circled blue).

(H) Model for the budding yeast replisome containing Pol α-primase and including $Pri2_{CTD}$ and $Pol1_{exo\text{-}cat}$, fit into the cryo-EM reconstruction in panel **(G)** (right). To fit $Pri2_{CTD}$, the primer DNA strand was removed from the crystal structure of human $PRIM2_{CTD}$ containing a primer/template junction (PDB: 5F0Q)[S2] before being rigid body fit into the $Pri2_{CTD}$ density. The model was initially placed in such an orientation as to maintain the correct lagging-strand template DNA polarity assuming the shortest path after emergence from the Pri1 active site. The AlphaFold[S3] model for yeast $Pri2_{CTD}$ was then aligned to 5F0Q. A crystal structure of the $Pol1_{exo\text{-}cat}$ domain bound to a DNA/RNA duplex and dGTP (PDB: 4FYD)[S4] was placed into the $Pol1_{exo\text{-}cat}$ density such that lagging-strand template DNA was oriented as to be continuous with the previously docked 5F0Q model. The fit-to-density was then optimised via rigid body docking in ChimeraX. DNA was removed from the model for visualisation.

(I) Comparison between the conformations of yeast Pol α-primase from this study (left), human Pol α-primase bound to CST and telomeric DNA (PDB: 8D0K)[S5] (middle) and human apo Pol α-primase (PDB: 5EXR)[S2] (right). Models were aligned on their $Pri2/PRIM2_{NTD}$. The $Pri2/PRIM2_{CTD}$ and $Pol1/POLA1_{exo\text{-}cat}$ domains are visualised using transparent surface rendering to highlight their relative positioning between the three models.

(J) Cryo-EM reconstruction of a budding yeast replisome containing Pol α-primase but lacking DNA. Regions of the map corresponding to Pol α-primase are coloured according to subunit occupancy.

(K) (Left) Atomic model for replisome associated Pol α-primase in the absence of DNA with the corresponding cryo-EM density from panel **(J)** overlaid. (Middle) Atomic model for the human apo Pol α-primase complex (PDB: 5EXR)[S2]. (Right) Atomic model for replisome associated yeast Pol α-primase complex engaged on DNA from this study. Models were aligned on $Pri2/PRIM2_{NTD}$.

(L) Model highlighting the arrangement of the primase and DNA polymerase catalytic centres, derived from the cryo-EM reconstruction containing density for both $Pri2_{CTD}$ and $Pol1_{exo\text{-}cat}$. The approximate location of the Pri1 and Pol1 active sites are indicated by red circles. The continuous purple line extending from the last modelled residue of the lagging-strand template is illustrative of the path of DNA described in Figure 1F. The dashed purple line indicates the proposed path of the lagging-strand template extending from the Pri1 active site towards the Pol1 active site.

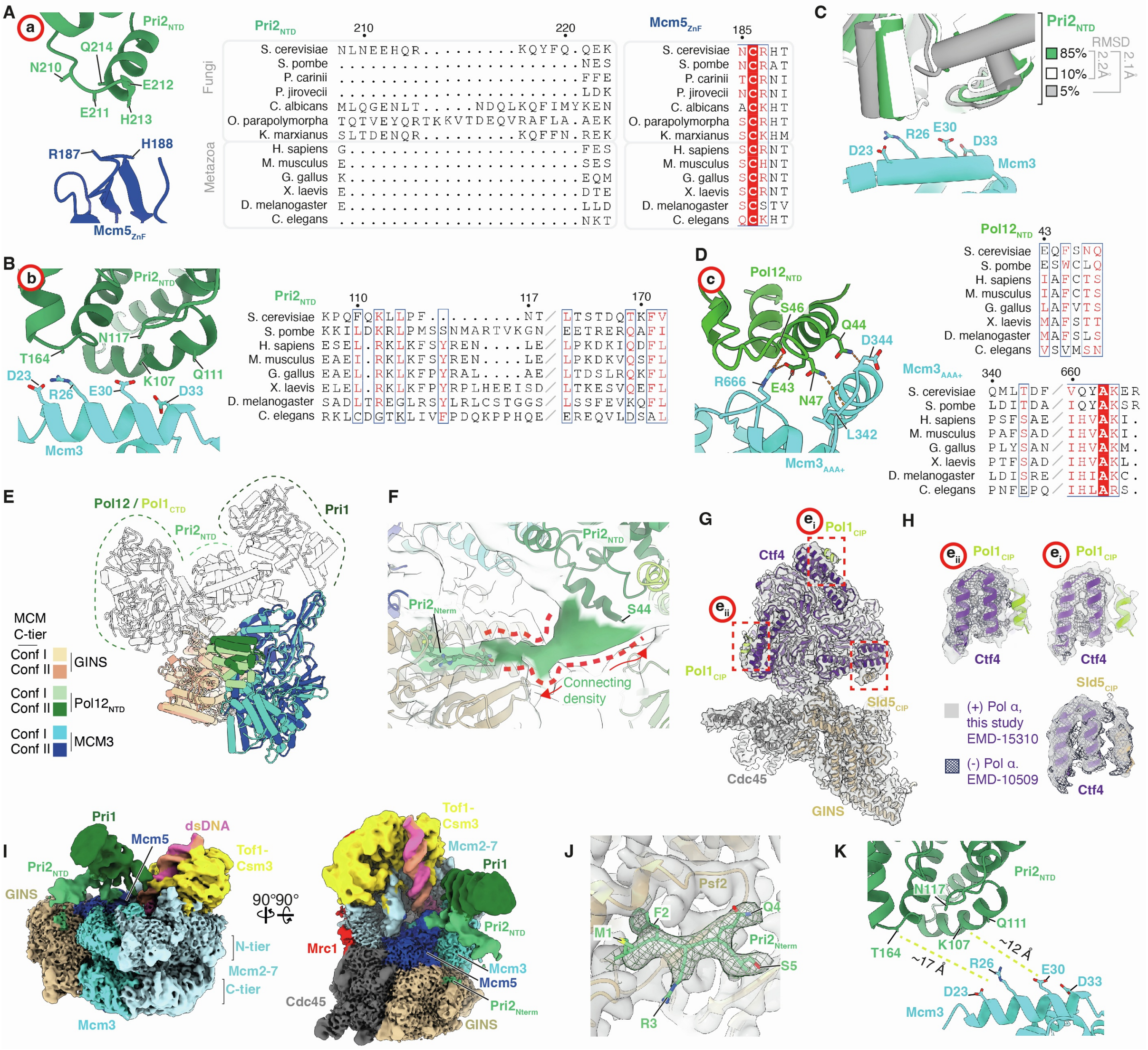

Figure S4. Details of budding yeast Pol α-primase replisome interactions, related to Figure 2.

(A) (Left) Focused view of the atomic model for the interface between Pri2$_{NTD}$ (green) and the Mcm5 ZnF domain (blue) (site a). Residues positioned to form inter-protein contacts are labelled. Residue sidechains are displayed as truncated stubs as the corresponding cryo-EM density is of insufficient resolution to determine their conformation. (Right) Multiple sequence alignments for the regions of Pri2/PRIM2 and Mcm5 involved in this interface, grouped by Fungal and Metazoan species and coloured according to conservation.

(B) Focused view of the atomic model for the electrostatic interface between Pri2$_{NTD}$ (green) and Mcm3 helix α1 of the N-terminal helical domain (cyan) (site b). Residue sidechains are only displayed when the corresponding cryo-EM density is of sufficiently high-resolution to determine their conformation, otherwise sidechains are displayed as truncated stubs. (Right) Multiple sequence alignment for the region of Pri2 involved in this interface coloured according to conservation. The corresponding sequence alignment for the Mcm3 residues involved in this interface are shown in Figure 2C.

(C) Atomic model highlighting the flexible nature of the interface between the Mcm3 N-terminal helical domain and Pri2$_{NTD}$ (site b). 3D variability analysis was carried out in cryoSPARC to understand the extent of the positional variance at this interface. Two reconstructions displaying the greatest divergence in Pri2$_{NTD}$ positioning with

respect to the Mcm3 were obtained. Models for both Mcm3 and Pri2 were rigid-body fit into each reconstruction, then each model aligned to the Mcm3 N-terminal helical domain. Visualised here is the overlay of the two resulting models (grey and white) alongside the dominant conformation deposited in the PDB (green). Included are the percentages of particles occupying each discrete class and their relative RMSD values.

(D) (Left) Focused view of the atomic model of the interface between $Pol12_{NTD}$ (green) and the Mcm3 AAA+ domain (cyan) (site c). Selected sidechains positioned to form inter-protein contacts are displayed, with predicted hydrogen bonds indicated by dashed orange lines. (Right) Multiple sequence alignments for the regions of $Pol12_{NTD}$ (top) and Mcm3 (bottom) involved in this interface, coloured according to conservation.

(E) Model illustrating the position of $Pol12_{NTD}$ bound to Mcm3 in the C-tier in conformations I and II[S6]. To generate the model for this interface in MCM C-tier conformation I, an existing model for Mcm3 and GINS in conformation I (PDB: 6SKL)[S6] was aligned to the N-tier of Mcm3 from this study (conformation II). The structure of the $Pol12_{NTD}$ in complex with the Mcm3 AAA+ domain from this study was then aligned to the Mcm3 AAA+ domain in conformation I from 6SKL. The interface can be maintained in both C-tier conformations without clashes.

(F) Model for the Pol α-primase associated replisome docked into a cryo-EM map (transparent grey surface) visualised using a low threshold. Continuous density is visible extending from the most C-terminal residue of $Pri2_{Nterm}$ visualised (S5) to the next visualised residue (S44). This connecting density was manually coloured green using ChimeraX.

(G) Atomic model for Cdc45, GINS and Ctf4 in complex with the Sld5 CIP-box and two copies of the Pol1 CIP-box docked into a locally refined cryo-EM density map (transparent grey surface).

(H) Focused view of the cryo-EM density at the CIP-box interaction sites on the helical bundle of each Ctf4 monomer. The crystal structures of the Pol1 CIP-box in complex with Ctf4 (PDB:4C93)[S7] and the Sld5 CIP box (PDB:4C95)[S8] were rigid-body docked into the cryo-EM density. Models were overlaid with cryo-EM density both from this study, and a yeast replisome reconstruction in the absence of Pol α-primase (EMD-10509)[S6].

(I) Alternative views of a cryo-EM reconstruction of a Pol α-primase associated replisome engaged only via the $Pri2_{NTD}$:$Mcm5_{ZnF}$ (site a) and $Pri2_{Nterm}$:Psf2 (site d) interfaces. The density is coloured according to chain occupancy as indicated.

(J) Model for the $Pri2_{Nterm}$:Psf2 interface (site d) built into the cryo-EM map described in **(I).** Cryo-EM density (transparent grey) displayed with the $Pri2_{Nterm}$ density highlighted using green mesh.

(K) Model focused on the regions of $Pri2_{NTD}$ and Mcm3 N-terminal helical domain (site b) involved in complex formation built into cryo-EM density for the map described in **I**. Residues seen to be interacting in site b are now too far away to make contacts. Distances between sidechains are indicated using dashed yellow lines.

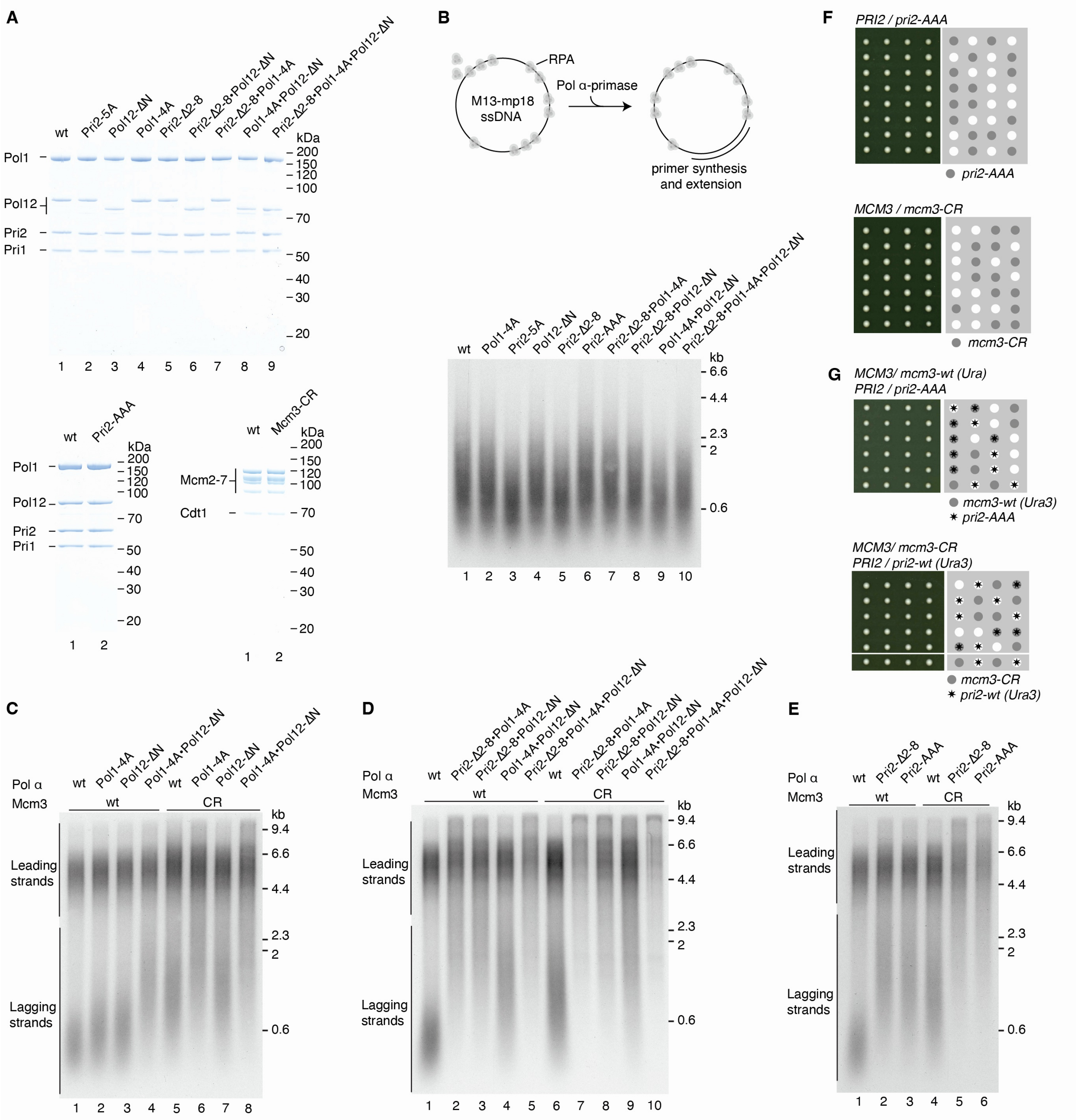

Figure S5. Analysis of budding yeast Pol α-primase interaction site mutants *in vitro* and *in vivo*, related to Figure 3.

(A) Coomassie-stained 4-12% SDS-PAGE analysis of purified Pol α-primase and Cdt1-Mcm2-7 mutant/truncated complexes. Pri2-5A, $Pri2^{N210A,\ E211A,\ E212A,\ H213A,\ Q214A}$; Pri2-AAA, $Pri2^{F2A,\ R3A,\ Q4A}$; Pol1-4A, $Pol1^{D141A,\ D142A,\ L144A,\ F147A}$; Pol12-ΔN, $Pol12^{\Delta 2\text{-}81}$, Mcm3-CR, $Mcm3^{D23R,\ R26E,\ E30R,\ D33R}$.

(B) Top, Schematic of the primase-polymerase assay on M13mp18 ssDNA in the presence of sub-saturating RPA. Bottom, alkaline agarose gel analysis of an assay performed as illustrated (top) with the indicated proteins for 20 min. **(C-E)** Denaturing agarose gel analysis of origin-dependent DNA replication reactions performed as illustrated in Figure 3B with the indicated proteins for 20 min.

(F and G) Diploid budding yeast cells of the indicated genotype were sporulated and the resulting tetrads were dissected and grown on YPD medium for 3 days at 25°C. Dissections that displayed abnormal segregation patterns were cropped from plate images.

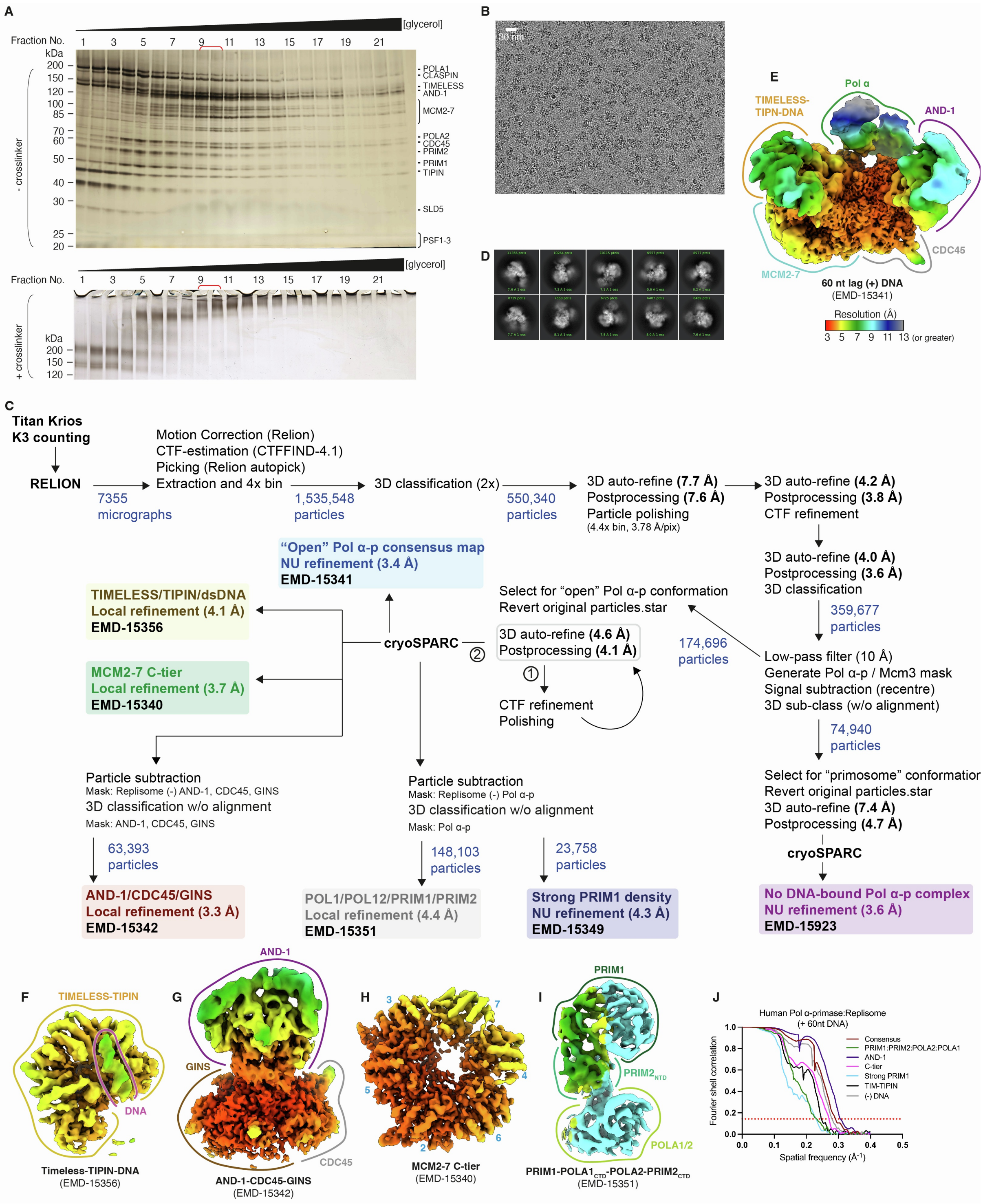

Figure S6. Cryo-EM analysis of a human replisome containing Pol α-primase assembled on fork DNA with 60 nt 5′-flap, related to Figure 4.

(A) Silver-stained SDS-PAGE gels of 100 µl fractions taken across 10-30% glycerol gradients, either in the absence (top) or presence (bottom) of crosslinking agents. Fractions 9-10 were used for cryo-EM sample preparation (red brackets). **(B)** Representative cryo-EM micrograph obtained using a K3 direct electron detector (Gatan) at a nominal pixel size of 1.23 Å/pixel. 30 nm scale bar inset.

(C) Cryo-EM data processing pipeline for the human replisome assembled on forked DNA with a 60 nt 5′-flap. “open” Pol α-primase conformation refers to the DNA engaged state of the complex that differs from the Pol α-primase apo structure (PDB:5EXR). Abbreviations: NU, non-uniform; w/o, without; Pol α-p, Pol α-primase. Shaded text denotes cryo-EM maps deposited in the EMDB.

(D) Representative 2D class averages using a mask boundary of 360 Å with corresponding particle numbers labelled, obtained using cryoSPARC-3.

(E-I) Cryo-EM reconstructions used in model building coloured by local resolution according to inset key in **(E)**. Local resolution was calculated using RELION for the reconstruction in panel **(E)**, and ResMap[S1] for panels **(F-I)**.

(J) Fourier shell correlation (FSC) graph describing the resolution of cryo-EM reconstructions used in model building. Resolution was calculated using the FSC=0.143 cut-off with values reported in **(C)**.

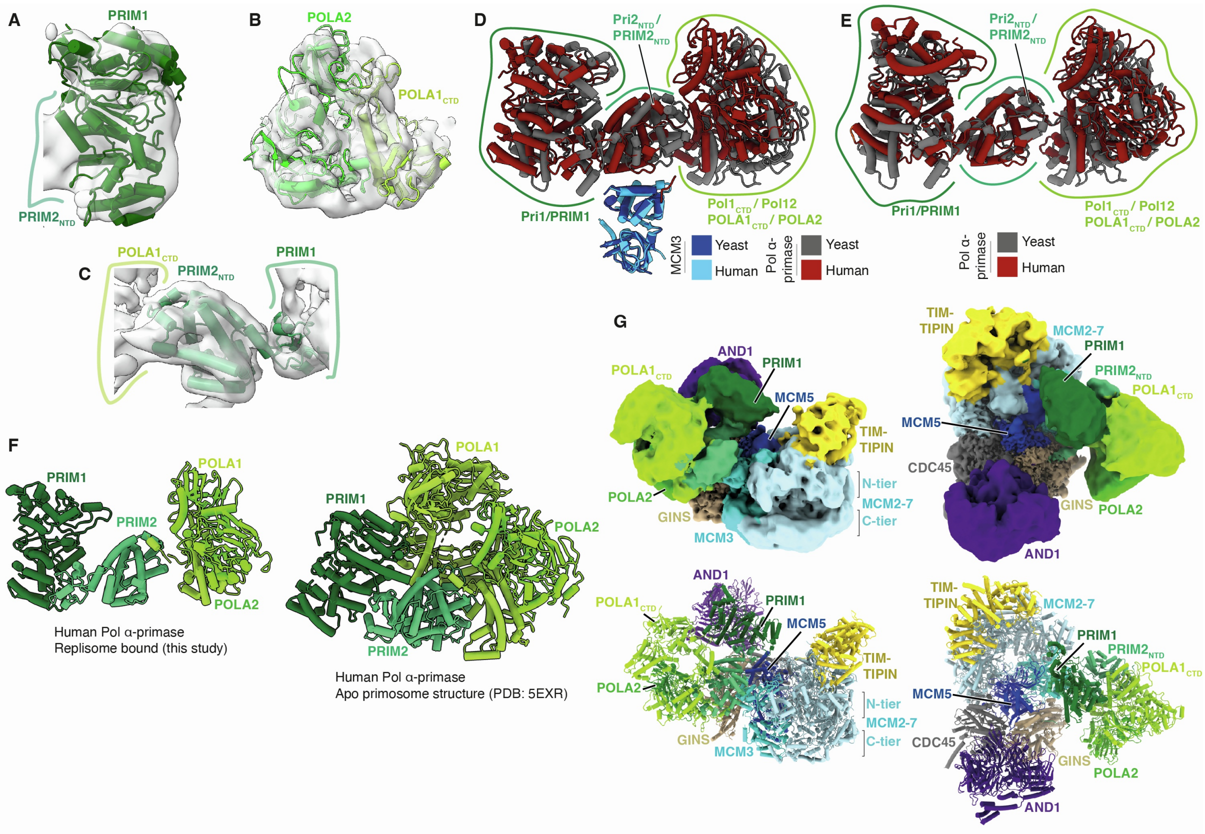

Figure S7. Structural analysis of Pol α-primase in the human replisome, related to Figure 4.

(A-C) Atomic models displayed with cryo-EM density for PRIM1 (**A**), POLA2 (**B**) and $PRIM2_{NTD}$ (**C**). Cryo-EM density corresponds to the local refinement shown in Figure S6I.

(D) Comparison between the atomic models of yeast (grey) and human (red) replisome associated Pol α-primase aligned on the Mcm3 N-terminal helical domain (blue).

(E) Comparison between the atomic models of yeast (grey) and human (red) replisome associated Pol α-primase aligned on the $Pri2_{NTD}$.

(F) Comparison between the structures of human Pol α-primase associated with DNA-engaged replisomes from this study (left) and the crystal structure of human apo Pol α-primase (right) (PDB: 5EXR)[S2]. Models were aligned on the $Pri2_{NTD}$.

(G) Alternative views of a cryo-EM reconstruction (top) and the corresponding model (bottom) for the human Pol α-primase associated replisome not bound to fork DNA. Maps were coloured according to domain occupancy.

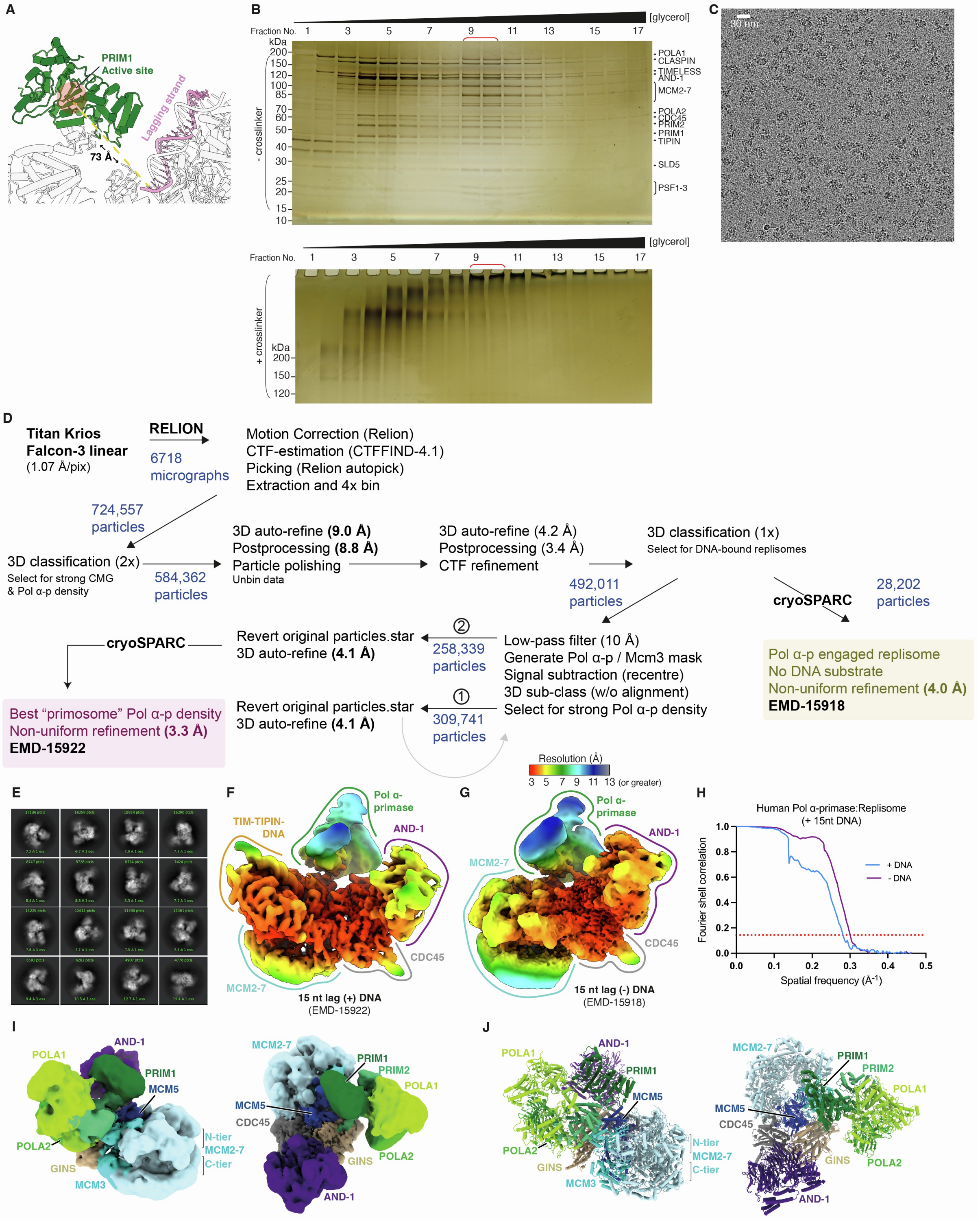

Figure S8. Cryo-EM structure and analysis of a human Pol α-primase associated replisome assembled on fork DNA with a 15 nt 5′-flap, related to Figure 5.

(A) Structural model indicating the shortest path and distance between the last modelled nucleotide of the lagging-strand template and the active site of PRIM1 (circled red), indicated using a dashed yellow line.

(B) Silver-stained SDS-PAGE gels of 100 µl fractions taken across 10-30% glycerol gradients in the absence (top) or presence (bottom) of crosslinking agents. Fractions 9-10 were used for cryo-EM sample preparation (red brackets).

(C) Representative cryo-EM micrograph obtained using a Falcon III direct electron detector (Thermo) at a nominal pixel size of 1.07 Å/pixel. 30 nm scale bar inset.

(D) Cryo-EM data processing pipeline for the human replisome assembled on forked DNA with a 15 nt 5′-flap. Abbreviations: w/o, without; Pol α-p, Pol α-primase. Shaded text denotes cryo-EM maps deposited in the EMDB.

(E) Representative 2D class averages using a mask boundary of 360 Å with corresponding particle numbers indicated obtained using cryoSPARC-3.

(F and G) Cryo-EM reconstructions obtained via consensus refinement for complexes engaged with forked DNA containing a 15 nt 5′-flap (left) and lacking DNA engagement (right). Maps coloured by local resolution, calculated using RELION, according to inset key in **(G)**.

(H) Fourier shell correlation (FSC) graph indicating the resolution for the cryo-EM reconstructions described in panels **(G)** and **(F)**. Resolution was calculated using the FSC=0.143 cut-off with values reported in (**D**).

(I and J) Alternate views of a cryo-EM reconstruction (**I**) and corresponding atomic model (**J**) for the Pol α-primase associated human replisome lacking DNA engagement obtained from the cryo-EM data collection of a human replisome assembled on a DNA fork with a 15 nt 5′-flap. Maps coloured according to subunit occupancy.

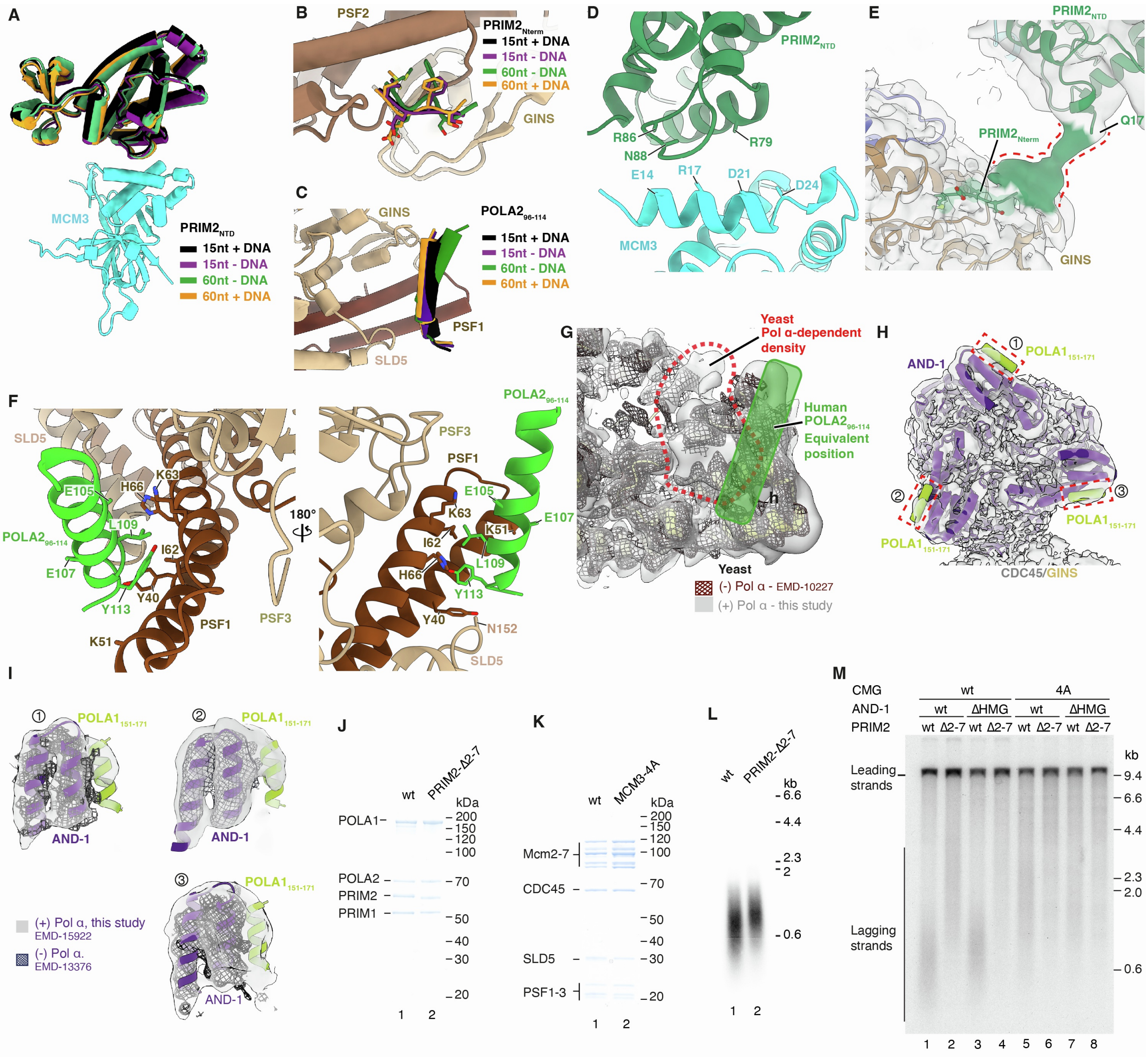

Figure S9. Analysis of Pol α-primase binding sites in the human replisome, Related to Figure 6.

(A) Focused view of the interface between $PRIM2_{NTD}$ and the MCM3 N-terminal helical domain (site b). Models overlaid were derived from reconstructions on fork DNA with either a 15 nt or 60 nt 5′-flap, in addition to models derived from these respective datasets where DNA engagement was not observed. Models were aligned to the MCM3 N-terminal helical domain**.**

(B) Focused view of the interface between $PRIM2_{Nterm}$ and PSF2. Models overlaid were derived from reconstructions on fork DNA with either a 15 nt or 60 nt 5′-flap, in addition to models derived from these respective datasets where DNA engagement was not observed. Models were aligned on PSF2.

(C) Focused view of the interface between $POLA2_{96-114}$ and GINS subunits PSF1 and SLD5. Models overlaid were derived from reconstructions on fork DNA with either a 15 nt or 60 nt 5′-flap, in addition to models derived from these respective datasets where DNA engagement was not observed. Models were aligned on PSF1.

(D) Detailed view of the atomic model for the interface between the $PRIM2_{NTD}$ (green) and the MCM3 N-terminal helical domain (cyan) (site b). Selected residue sidechains positioned to form inter-protein contacts are labelled.

Residue sidechains are displayed as truncated stubs as the corresponding cryo-EM density is of insufficient resolution to determine their conformation.

(E) Pol α-primase associated replisome model docked into a cryo-EM map in which continuous low-resolution density is visualised between the most C-terminal modelled residue of the $PRIM2_{Nterm}$ (G5) and the next modelled residue (Q17). The connecting density was manually coloured green using ChimeraX.

(F) Alternative views of the atomic model for the interface between the POLA2 N-terminal helix (residues 96-114) (green) and GINS subunits PSF1, PSF3 and SLD5 (brown). Residue sidechains positioned to form inter-protein contacts are labelled. Residue sidechains are only displayed when the corresponding cryo-EM density is of sufficiently high-resolution to determine their conformation, otherwise sidechains are displayed as truncated stubs.

(G) Overlay of cryo-EM density for reconstructions of budding yeast replisomes containing (transparent grey, this study) and lacking (black mesh, EMD-10227) Pol α-primase[S6]. The associated atomic model is docked into the density (yellow). The approximate position of the human POLA2 helix (residues 96-114) bound to PSF1 and SLD5 is overlaid in green and unmodelled density in the vicinity in yeast reconstructions is circled in red.

(H) Model for AND-1 in complex with three copies of the POLA1 AND-1 interaction motif (residues 151-171), docked into a locally refined reconstruction.

(I) Focused views of the cryo-EM density for the helical bundle of each AND-1 monomer bound by POLA1 (transparent grey) overlaid with cryo-EM density for a reconstruction of the human replisome in the absence of Pol α-primase (EMDB-13376)[S9] (black mesh). An AlphaFold-Multimer model for the interface between $POLA1_{151\text{-}171}$ and the helical bundle of AND-1 was aligned to each AND-1 monomer and the fit optimised using rigid-body docking into the cryo-EM map.

(J) Coomassie-stained SDS-PAGE gel of purified wild-type human Pol α-primase (lane 1) and mutant Pol α-primase (lane 2) containing a truncation of residues 2-7 in PRIM2 designed to disrupt the interface with PSF2.

(K) Coomassie-stained SDS-PAGE gel of purified human CMG (lane 1) and a mutant CMG complex (lane 2) in which four conserved residues on helix α1 of MCM3 were mutated to alanine (MCM3-4A: E14A, R17A, D21A and D24A) to disrupt the interface with PRIM2.

(L) Denaturing agarose gel analysis of a primase-polymerase assay performed as illustrated in Figure S5B for 20 min with the indicated proteins.

(M) *In vitro* DNA replication reaction with purified human proteins performed as in Figure 6F and 6G.

SUPPLEMENTARY TABLES (Tables S1-S3)

Plasmid name	Proteins expressed	Plasmid details
vVA52	*S. cerevisiae* Mcm2, Mcm3-CR	pRS306-CBP-TEV-Mcm3$^{D23R, R26E, E30R, D33R}$-Gal1-10-Mcm2
vVA58	*S. cerevisiae* Pol1-4A, Pol12	pRS304-Pol1$^{D141A, D142A, L144A, F147A}$-Gal1-10-Pol12
vJY186	*S. cerevisiae* Pol1, Pol12-ΔN	pRS304-Pol1 -Gal1-10-Pol12$^{\Delta 2\text{-}81}$
vJY187	*S. cerevisiae* Pol1-4A, Pol12-ΔN	pRS304-Pol1$^{D141A, D142A, L144A, F147A}$-Gal1-10-Pol12$^{\Delta 2\text{-}81}$
vJY196	*S. cerevisiae* Pri1, Pri2-Δ2-8	pRS306-CBP-TEV-Pri1-Gal1-10-Pri2$^{\Delta 2\text{-}8}$
vJY183	*S. cerevisiae* Pri1, Pri2-5A	pRS306-CBP-TEV-Pri1-Gal1-10-Pri2$^{N210A, E211A, E212A, H213A, Q214A}$
vJY199	*S. cerevisiae* Pri1, Pri2-AAA	pRS306-CBP-TEV-Pri1-Gal1-10-Pri2$^{F2A, R3A, Q4A}$
vMJ9	*H. sapiens* PRIM2-Δ2-7	pACEBac1-PRIM2$^{\Delta 2\text{-}7}$
YB_X2	*H. sapiens* MCM2, MCM5, MCM3	pBIG1a-MCM2, MCM5, MCM3
vVA62	*H. sapiens* MCM2, MCM5, MCM3-4A	pBIG1a-MCM2, MCM5, MCM3$^{E14A, R17A, D21A, D24A}$
YB_X3	*H. sapiens* MCM4, MCM6, MCM7	pBIG1b-MCM7, MCM4, MCM6
vJY177	N/A	Yeast Mcm3 ORF + 935 bp upstream DNA cloned into pFA6-URA at SalI (5′) and AscI (3′) restriction sites. Mcm3-CR mutations were introduced by site-directed mutagenesis. The sequence of the Mcm3 ORF was confirmed by Sanger sequencing.
vJY206	N/A	Yeast Pri2 ORF +705 bp upstream and 156 bp downstream DNA cloned into pFA6-URA at SalI (5′) and AscI (3′) restriction sites. Pri2-AAA mutations were introduced by site-directed mutagenesis. The sequence of the Pri2 ORF was confirmed by Sanger sequencing.

Table S1. Plasmids constructed for this study, related to METHODS.

**Strain Name**	**Protein expressed**	**Genotype**
yVA87	Cdt1-Mcm2-7 (Mcm3-CR)	*MATa ade2-1 ura3-1 his3-11,15 trp1-1 leu2-3,112 can1-100*<br>*bar1::Hyg*<br>*pep4::KanMX*<br>*his3::HIS3pRS303-Cdt1-Gal1-10-Gal4*<br>*trp1::TRP1pRS304-Mcm4-Gal1-10-Mcm5*<br>*leu2::LEU2pRS305-Mcm6-Gal1-10-Mcm7*<br>*ura3::URA3pRS306-CBP-TEV-Mcm3-CR (vVA52)-Gal- Mcm2*
yJY239	Pol α-primase (Pol12-ΔN)	*MATa ade2-1 ura3-1 his3-11,15 trp1-1 leu2-3,112 can1-100*<br>*bar1::Hyg*<br>*pep4::KanMX*<br>*trp1::TRP1-pRS304-Pol1- Gal1-10-Pol12-ΔN (vJY186)*<br>*ura3::URA3pRS306-CBP-TEV-Pri1-Gal1-10-Pri2*<br>*pol1-3XFLAG (NatNT2)*
yJY381	Pol α-primase (Pol1-4A•Pol12-ΔN)	*MATa ade2-1 ura3-1 his3-11,15 trp1-1 leu2-3,112 can1-100*<br>*bar1::Hyg*<br>*pep4::KanMX*<br>*trp1::TRP1pRS304-Pol1-4A-Gal1-10-Pol12-ΔN (vJY187)*<br>*ura3::URA3pRS306-CBP-TEV-Pri1-Gal1-10-Pri2*<br>*pol1-3XFLAG (NatNT2)*
yVA96	Pol α-primase (Pol1-4A)	*MATa ade2-1 ura3-1 his3-11,15 trp1-1 leu2-3,112 can1-100*<br>*bar1::Hyg*<br>*pep4::KanMX*<br>*ura::URA3pRS306-CBP-TEV-Pri1-Gal1-10-Pri2*<br>*trp::TRP1pRS304-Pol1-4A-Gal1-10-Pol12 (vVA58)*<br>*pol1-3XFLAG (NatNT2)*
yJY232	Pol α-primase (Pri2-5A)	*MATa ade2-1 ura3-1 his3-11,15 trp1-1 leu2-3,112 can1-100*<br>*bar1::Hyg*<br>*pep4::KanMX*<br>*trp1::TRP1-pRS304-Pol1-Gal1-10-Pol12*<br>*ura3::URA3pRS306-CBP-TEV-Pri1-Gal1-10-Pri2-5A (vJY183)*
yJY241	Pol α-primase (Pri2-Δ2-8)	*MATa ade2-1 ura3-1 his3-11,15 trp1-1 leu2-3,112 can1-100*<br>*bar1::Hyg*<br>*pep4::KanMX*<br>*trp1::TRP1pRS304-Pol1-Gal1-10-Pol12*<br>*ura3::URA3pRS306-CBP-TEV-Pri1-Gal1-10-Pri2Δ2-8 (vJY196)*
yJY242	Pol α-primase (Pri2-AAA)	*MATa ade2-1 ura3-1 his3-11,15 trp1-1 leu2-3,112 can1-100*<br>*bar1::Hyg*<br>*pep4::KanMX*

		trp1::TRP1pRS304-Pol1-Gal1-10-Pol12<br>*ura3::URA3pRS306-CBP-TEV-Pri1-Gal1-10-Pri2-AAA (vJY199)*
yMJ12	Pol α-primase (Pol1-4A•Pri2-Δ2-8)	*MATa ade2-1 ura3-1 his3-11,15 trp1-1 leu2-3,112 can1-100*<br>*bar1::Hyg*<br>*pep4::KanMX*<br>*trp1::TRP1pRS304-Pol1-4A-Gal1-10-Pol12 (vVA58)*<br>*ura3::URA3pRS306-CBP-TEV-Pri1-Gal1-10-Pri2-Δ2-8 (vJY196)*
yMJ13	Pol α-primase (Pol1-4A•Pol12-ΔN•Pri2-Δ2-8)	*MATa ade2-1 ura3-1 his3-11,15 trp1-1 leu2-3,112 can1-100*<br>*bar1::Hyg*<br>*pep4::KanMX*<br>*trp1::TRP1pRS304-Pol1-4A-Gal1-10-Pol12-ΔN (vJY187)*<br>*ura3::URA3pRS306-CBP-TEV-Pri1-Gal1-10-Pri2-Δ2-8 (vJY196)*<br>*pol1-3XFLAG (NatNT2)*
yMJ18	Pol α-primase (Pol12-ΔN•Pri2-Δ2-8)	*MATa ade2-1 ura3-1 his3-11,15 trp1-1 leu2-3,112 can1-100*<br>*bar1::Hyg*<br>*pep4::KanMX*<br>*trp1::TRP1pRS304-Pol1-Gal1-10-Pol12-ΔN (vJY186)*<br>*ura3::URA3pRS306-CBP-TEV-Pri1-Gal1-10-Pri2-Δ2-8 (vJY196)*<br>*pol1-3XFLAG (NatNT2)*

Table S2. ***S. cerevisiae*** **protein expression strains constructed for this study, related to METHODS.**

Protein	Expression strain / plasmid	Affinity tag	Purification steps	Reference where Purification method described
Cdc45	yJY13	Internal 2xFLAG tag	Anti-FLAG M2 Agarose<br>Bio-Gel HT Hydroxyapatite	[S10]
Cdc6	Plasmid pAM3 (*E. coli* expression)	N-terminal GST cleavable tag	Glutathione Sepharose 4B<br>Bio-Gel HT Hydroxyapatite	[S11]
Cdt1-Mcm2-7	yAM33	N-terminal CBP cleavable tag on Mcm3	Calmodulin-Sepharose 4B<br>Superdex 200	[S11]
Tof1-Csm3	yAE48	N-terminal CBP cleavable tag on Csm3	Calmodulin-Sepharose 4B<br>MonoQ<br>Superdex 200	[S6,12]
Ctf4	yAE40	N-terminal CBP tag	Calmodulin-Sepharose 4B<br>MonoQ<br>Superdex 200	[S10]
DDK	ySDK8	CBP tag on Dbf4	Calmodulin-Sepharose 4B<br>Lambda phosphatase dephosphorylation<br>Superdex 200	[S13]
Dpb11	yJY26	C-terminal 3xFLAG tag	Anti-FLAG M2 Agarose<br>MonoS	[S10]
GINS	Plasmid pJFDJ5 (*E. coli* expression)	N-terminal His tag on Psf3	Ni-NTA Agarose<br>MonoQ<br>Superdex 200	[S10]
Mcm10	pET28a-Mcm10 (*E. coli* expression)	N-terminal His tag	Ni-NTA Agarose<br>MonoS (twice)	[S10]
Mrc1	yJY32	C-terminal 2xFLAG tag	Anti-FLAG M2 Agarose<br>Superose 6	[S6,12]
ORC	ySD-ORC	CBP-cleavable tag on Orc1	Calmodulin-Sepharose 4B<br>Superdex 200	[S14]
Yeast PCNA	vJY19 (*E. coli* expression)	Untagged	Nucleic acid precipitation with Polymin P<br>Ammonium sulfate precipitation<br>HiTrap SP HP (flow through)<br>HiTrap Heparin HP (flow through)<br>HiTrap DEAE Fast Flow<br>MonoQ	[S12]
Yeast Pol α-primase	yAE95	N-terminal CBP tag on Pri1	Calmodulin-Sepharose 4B<br>MonoQ<br>Superdex 200	[S12]
Yeast Pol δ	yAE34	C-terminal CBP tag on Pol32	Calmodulin-Sepharose 4B<br>HiTrap Heparin HP<br>Superdex 200	[S12]
Yeast Pol ε	yAJ2	C-terminal CBP tag on Dpb4	Calmodulin-Sepharose 4B<br>HiTrap Heparin HP<br>Superdex 200	[S10]
Yeast RFC	yAE41	N-terminal CBP tag on Rfc3	Calmodulin-Sepharose 4B<br>MonoS<br>Superdex 200	[S12]
Yeast RPA	yJY106	Untagged	Nucleic acid precipitation with Polymin P<br>Ammonium sulfate precipitation<br>HiTrap Blue HP<br>ssDNA Cellulose<br>MonoQ	[S6]
Sld2	yTD8	C-terminal 3x FLAG	Ammonium sulfate precipitation<br>Anti-FLAG M2 Agarose<br>HiTrap SP HP	[S10]
Sld3/7	yTD6	C-terminal cleavable TCP tag	IgG Sepharose Fast Flow<br>TEV removal with Ni-NTA Agarose<br>Superdex 200	[S10]
Yeast CMG	yJY197	Internal 2xFLAG tag on Cdc45<br>N-terminal CBP cleavable tag on Mcm3<br>N-terminal His tag on Psf3	Anti-FLAG M2 Agarose<br>Calmodulin-Sepharose 4B<br>MonoQ	[S6,15]
TIMELESS-TIPIN	MT_DF1, MT_BD1	N-terminal Twin-Strep TEV on TIMELESS	Strep-Tactin XT superflow high capacity<br>HiTrap Q HP	[S9] and this study

			Superdex 200i	
AND-1	MT_BF1	N-terminal 3X FLAG TEV	Anti-FLAG M2 Agarose<br>MonoQ<br>Superdex 200i	[S9]
AND-1-Δ1017	YB_8	N-terminal 3X FLAG TEV	Anti-FLAG M2 Agarose<br>MonoQ<br>Superdex 200i	[S16]
Human Pol α-primase	MT_BC3, MT_AE1, MT_AF1, MT_AG1	N-terminal Twin-Strep 3C on POLA1	Strep-Tactin XT superflow high capacity<br>MonoQ<br>Superdex 200i	[S16]
CLASPIN	MT_DB1	C-terminal HRV 3C 3X FLAG	Anti-FLAG M2 Agarose<br>Superose 6	[S9]
Human PCNA	MT_EB1	Untagged	Nucleic acid precipitation with Polymin P<br>Ammonium sulfate precipitation<br>Tandem HiTrap SP FF, HiTrap heparin<br>DEAE column<br>MonoQ<br>Superdex 200i	[S9]
Human Pol δ	MT_CF1, MT_CH1, YB_3, MT_FC1	N-terminal Twin-Strep-TEV on POLD4	Strep-Tactin XT superflow high capacity<br>MonoQ<br>Superdex 200i	[S16]
Human Pol ε	MT_U2, MT_L1, MT_M1, MT_N1	C-terminal TEV CBP on POLE3 (not used for purification)	Nucleic acid precipitation with Polymin P<br>Ammonium sulfate precipitation<br>HiTrap SP FF<br>HiTrap Q FF<br>HiTrap heparin<br>MonoQ<br>Superdex 200i	[S16]
Human RFC	MT_BH1, MT_BJ1, MT_BK1, MT_BL1, MT_BI1		Strep-Tactin XT superflow high capacity<br>HiTrap Heparin<br>MonoQ	[S9]
Human RPA	YB_X1	Untagged	HiTrap Blue<br>Bio-Gel HT hydroxyapatite<br>MonoQ	[S9]
CTF18-RFC	YB_7, YB_5, YB_6, YB_4	N-terminal Twin-Strep-TEV on CTF18	Strep-Tactin XT superflow high capacity<br>MonoQ<br>Superdex 200i	[S16]
Human CMG	YB_2, YB_1, MT_01	N-terminal Twin-Strep HRV 3C on SLD5, Internal FLAG in CDC45	Anti-FLAG M2 Agarose<br>Strep-Tactin XT superflow high capacity<br>MonoQ	[S9]

Table S3. Details of protein purification strategy, related to METHODS.

**Strain name**	**Genotype**
yJY244	*MATa ade2-1 ura3-1 his3-11,15 trp1-1 leu2-3,112 can1-100 / MATα ade2-1 ura3-1 his3-11,15 trp1-1 leu2-3,112 can1-100*<br>*PRI2 / PRI2 (URA3)*
yJY297	*MATa ade2-1 ura3-1 his3-11,15 trp1-1 leu2-3,112 can1-100 / MATα ade2-1 ura3-1 his3-11,15 trp1-1 leu2-3,112 can1-100*<br>*MCM3 / MCM3 (Ura3)*
yJY321	MATa ade2-1 ura3-1 his3-11,15 trp1-1 leu2-3,112 can1-100 / MATα ade2-1 ura3-1 his3-11,15 trp1-1 leu2-3,112 can1-100<br>*PRI2 / pri2-AAA (URA3)*
yJY300	*MATa ade2-1 ura3-1 his3-11,15 trp1-1 leu2-3,112 can1-100 / MATα ade2-1 ura3-1 his3-11,15 trp1-1 leu2-3,112 can1-100*<br>*MCM3 / mcm3-CR (Ura3)*
yJY301	*MATa ade2-1 ura3-1 his3-11,15 trp1-1 leu2-3,112 can1-100 / MATα ade2-1 ura3-1 his3-11,15 trp1-1 leu2-3,112 can1-100*<br>*MCM3 / mcm3-CR (Ura3)*
yJY365	*MATa ade2-1 ura3-1 his3-11,15 trp1-1 leu2-3,112 can1-100 / MATα ade2-1 ura3-1 his3-11,15 trp1-1 leu2-3,112 can1-100*<br>*MCM3 / mcm3-CR (Ura3)*<br>*PRI2 / PRI2 (URA3)*
yJY367	*MATa ade2-1 ura3-1 his3-11,15 trp1-1 leu2-3,112 can1-100 / MATα ade2-1 ura3-1 his3-11,15 trp1-1 leu2-3,112 can1-100*<br>*MCM3 / MCM3 (Ura3)*<br>*PRI2 / pri2-AAA (URA3)*
yJY345	*MATa ade2-1 ura3-1 his3-11,15 trp1-1 leu2-3,112 can1-100 / MATα ade2-1 ura3-1 his3-11,15 trp1-1 leu2-3,112 can1-100*<br>*MCM3 / mcm3-CR (Ura3)*<br>*PRI2 / pri2-AAA (URA3)*
yJY350	*MATa ade2-1 ura3-1 his3-11,15 trp1-1 leu2-3,112 can1-100 / MATα ade2-1 ura3-1 his3-11,15 trp1-1 leu2-3,112 can1-100*<br>*MCM3 / mcm3-CR (Ura3)*<br>*SML1 / sml1Δ::HIS3*
yJY351	*MATa ade2-1 ura3-1 his3-11,15 trp1-1 leu2-3,112 can1-100 / MATα ade2-1 ura3-1 his3-11,15 trp1-1 leu2-3,112 can1-100*<br>*PRI2 / pri2-AAA (URA3)*<br>*SML1 / sml1Δ::HIS3*
yJY356	*MATa ade2-1 ura3-1 his3-11,15 trp1-1 leu2-3,112 can1-100 / MATα ade2-1 ura3-1 his3-11,15 trp1-1 leu2-3,112 can1-100*<br>*MCM3 / mcm3-CR (Ura3)*

	sml1Δ::HIS3 / sml1Δ::HIS3<br>*MEC1 / mec1Δ::ADE2*
yJY357	*MATa ade2-1 ura3-1 his3-11,15 trp1-1 leu2-3,112 can1-100 / MATα ade2-1 ura3-1 his3-11,15 trp1-1 leu2-3,112 can1-100*<br>*PRI2 / pri2-AAA (URA3)*<br>*sml1Δ::HIS3 / sml1Δ::HIS3*<br>*MEC1 / mec1Δ::ADE2*
yJY255	*MATa ade2-1 ura3-1 his3-11,15 trp1-1 leu2-3,112 can1-100*<br>*PRI2 (URA3)*
yJY302	*MATa ade2-1 ura3-1 his3-11,15 trp1-1 leu2-3,112 can1-100*<br>*MCM3 (Ura3)*
yJY326	*MATa ade2-1 ura3-1 his3-11,15 trp1-1 leu2-3,112 can1-100*<br>*pri2-AAA (URA3)*
yJY328	*MATα ade2-1 ura3-1 his3-11,15 trp1-1 leu2-3,112 can1-100*<br>*pri2-AAA (URA3)*
yJY313	*MATa ade2-1 ura3-1 his3-11,15 trp1-1 leu2-3,112 can1-100*<br>*mcm3-CR (Ura3)*
yJY315	*MATα ade2-1 ura3-1 his3-11,15 trp1-1 leu2-3,112 can1-100*<br>*mcm3-CR (Ura3)*
yJY317	*MATa ade2-1 ura3-1 his3-11,15 trp1-1 leu2-3,112 can1-100*<br>*mcm3-CR (Ura3)*
yJY352	*MATa ade2-1 ura3-1 his3-11,15 trp1-1 leu2-3,112 can1-100*<br>*mcm3-CR (Ura3)*<br>*sml1Δ::HIS3*
yJY354	*MATa ade2-1 ura3-1 his3-11,15 trp1-1 leu2-3,112 can1-100*<br>*pri2-AAA (URA3)*<br>*sml1Δ::HIS3*

Table S4. *S. cerevisiae* strains constructed in this study for genetics experiments. All strains are based on the W303 background, related to METHODS.

Supplemental References

S1. Kucukelbir, A., Sigworth, F.J., and Tagare, H.D. (2014). Quantifying the local resolution of cryo-EM density maps. Nat Methods *11*, 63-65. 10.1038/nmeth.2727.

S2. Baranovskiy, A.G., Babayeva, N.D., Zhang, Y., Gu, J., Suwa, Y., Pavlov, Y.I., and Tahirov, T.H. (2016). Mechanism of Concerted RNA-DNA Primer Synthesis by the Human Primosome*. Journal of Biological Chemistry *291*, 10006-10020. https://doi.org/10.1074/jbc.M116.717405.

S3. Jumper, J., Evans, R., Pritzel, A., Green, T., Figurnov, M., Ronneberger, O., Tunyasuvunakool, K., Bates, R., Žídek, A., Potapenko, A., et al. (2021). Highly accurate protein structure prediction with AlphaFold. Nature. 10.1038/s41586-021-03819-2.

S4. Perera, R.L., Torella, R., Klinge, S., Kilkenny, M.L., Maman, J.D., and Pellegrini, L. (2013). Mechanism for priming DNA synthesis by yeast DNA polymerase alpha. Elife *2*, e00482. 10.7554/eLife.00482.

S5. He, Q., Lin, X., Chavez, B.L., Agrawal, S., Lusk, B.L., and Lim, C.J. (2022). Structures of the human CST-Polα–primase complex bound to telomere templates. Nature *608*, 826-832. 10.1038/s41586-022-05040-1.

S6. Baretić, D., Jenkyn-Bedford, M., Aria, V., Cannone, G., Skehel, M., and Yeeles, J.T.P. (2020). Cryo-EM Structure of the Fork Protection Complex Bound to CMG at a Replication Fork. Molecular Cell *78*, 926-940.e913. https://doi.org/10.1016/j.molcel.2020.04.012.

S7. Simon, A.C., Zhou, J.C., Perera, R.L., van Deursen, F., Evrin, C., Ivanova, M.E., Kilkenny, M.L., Renault, L., Kjaer, S., Matak-Vinkovic, D., et al. (2014). A Ctf4 trimer couples the CMG helicase to DNA polymerase alpha in the eukaryotic replisome. Nature *510*, 293-297. 10.1038/nature13234.

S8. Bellelli, R., and Boulton, S.J. (2021). Spotlight on the Replisome: Aetiology of DNA Replication-Associated Genetic Diseases. Trends Genet *37*, 317-336. 10.1016/j.tig.2020.09.008.

S9. Jones, M.L., Baris, Y., Taylor, M.R.G., and Yeeles, J.T.P. (2021). Structure of a human replisome shows the organisation and interactions of a DNA replication machine. The EMBO Journal *n/a*, e108819. https://doi.org/10.15252/embj.2021108819.

S10. Yeeles, J.T., Deegan, T.D., Janska, A., Early, A., and Diffley, J.F. (2015). Regulated eukaryotic DNA replication origin firing with purified proteins. Nature *519*, 431-435. 10.1038/nature14285.

S11. Coster, G., Frigola, J., Beuron, F., Morris, E.P., and Diffley, J.F. (2014). Origin licensing requires ATP binding and hydrolysis by the MCM replicative helicase. Mol Cell *55*, 666-677. 10.1016/j.molcel.2014.06.034.

S12. Yeeles, J.T.P., Janska, A., Early, A., and Diffley, J.F.X. (2017). How the Eukaryotic Replisome Achieves Rapid and Efficient DNA Replication. Mol Cell *65*, 105-116. 10.1016/j.molcel.2016.11.017.

S13. On, K.F., Beuron, F., Frith, D., Snijders, A.P., Morris, E.P., and Diffley, J.F. (2014). Prereplicative complexes assembled in vitro support origin-dependent and independent DNA replication. EMBO J *33*, 605-620. 10.1002/embj.201387369.

S14. Frigola, J., Remus, D., Mehanna, A., and Diffley, J.F. (2013). ATPase-dependent quality control of DNA replication origin licensing. Nature *495*, 339-343. 10.1038/nature11920.

S15. Jenkyn-Bedford, M., Jones, M.L., Baris, Y., Labib, K.P.M., Cannone, G., Yeeles, J.T.P., and Deegan, T.D. (2021). A Conserved Mechanism for Regulating Replisome Disassembly in Eukaryotes. Nature. 10.1038/s41586-021-04145-3.

S16. Baris, Y., Taylor, M.R.G., Aria, V., and Yeeles, J.T.P. (2022). Fast and efficient DNA replication with purified human proteins. Nature *606*, 204-210. 10.1038/s41586-022-04759-1.
