# Supplementary material for: How Pol α-primase is targeted to replisomes to prime eukaryotic DNA replication
Source: Mol Cell. 2023 Aug 17;83(16):2911–2924.e16. doi: 10.1016/j.molcel.2023.06.035 (PMC10501992; doi:10.1016/j.molcel.2023.06.035)
Supplement: Document S2. Article plus supplemental information [file mmc4.pdf]

Molecular Cell

Article

# How Pol α-primase is targeted to replisomes to prime eukaryotic DNA replication

## Graphical abstract

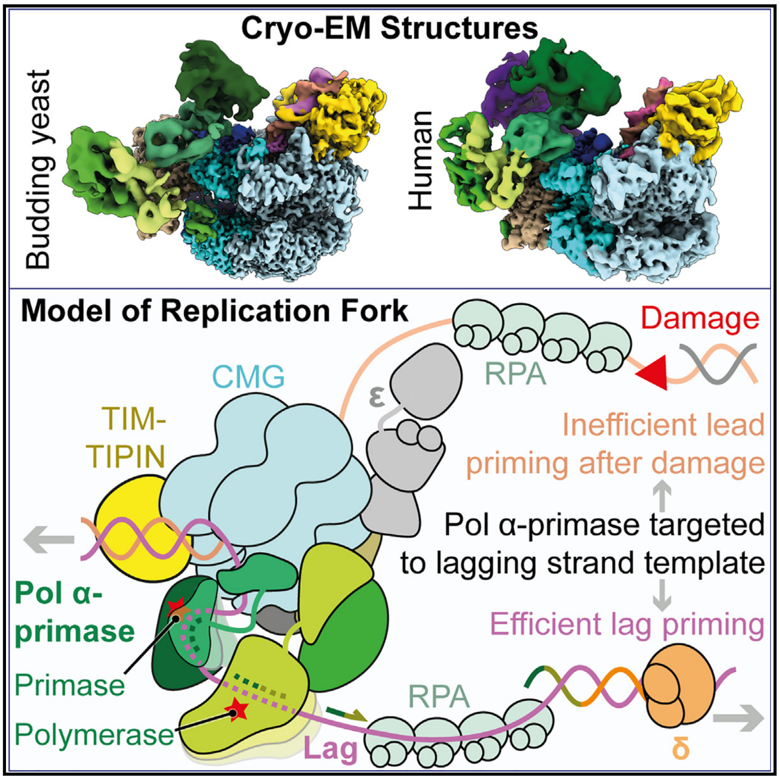


## Highlights

- Structures of DNA engaged yeast and human replisomes containing Pol α-primase
- A conserved mechanism targets Pol α-primase to the replisome for priming
- A multisite interaction network targets primase to the lagging-strand template
- Direct interactions between CMG and Pol α-primase are critical for priming

## Authors

Morgan L. Jones, Valentina Aria, Yasemin Baris, Joseph T.P. Yeeles

## Correspondence

jyeeles@mrc-lmb.cam.ac.uk

## In brief

By determining cryo-EM structures of budding yeast and human replisomes containing the Pol α-primase complex, Jones et al. reveal a conserved mechanism for the coordination of nascent-strand priming in the eukaryotic replisome. The mechanism explains why priming by Pol α-primase is highly efficient on the lagging-strand but not leading-strand template.

Jones et al., 2023, Molecular Cell 83, 2911–2924
August 17, 2023 © 2023 MRC Laboratory of Molecular Biology.
Published by Elsevier Inc.
https://doi.org/10.1016/j.molcel.2023.06.035

Molecular Cell

CellPress
OPEN ACCESS

Article

# How Pol α-primase is targeted to replisomes to prime eukaryotic DNA replication

Morgan L. Jones,[1] Valentina Aria,[1] Yasemin Baris,[1] and Joseph T.P. Yeeles[1,2,*]
[1]MRC Laboratory of Molecular Biology, Cambridge CB2 0QH, UK
[2]Lead contact
*Correspondence: jyeeles@mrc-lmb.cam.ac.uk
https://doi.org/10.1016/j.molcel.2023.06.035

## SUMMARY

During eukaryotic DNA replication, Pol α-primase generates primers at replication origins to start leading-strand synthesis and every few hundred nucleotides during discontinuous lagging-strand replication. How Pol α-primase is targeted to replication forks to prime DNA synthesis is not fully understood. Here, by determining cryoelectron microscopy (cryo-EM) structures of budding yeast and human replisomes containing Pol α-primase, we reveal a conserved mechanism for the coordination of priming by the replisome. Pol α-primase binds directly to the leading edge of the CMG (CDC45-MCM-GINS) replicative helicase via a complex interaction network. The non-catalytic PRIM2/Pri2 subunit forms two interfaces with CMG that are critical for *in vitro* DNA replication and yeast cell growth. These interactions position the primase catalytic subunit PRIM1/Pri1 directly above the exit channel for lagging-strand template single-stranded DNA (ssDNA), revealing why priming occurs efficiently only on the lagging-strand template and elucidating a mechanism for Pol α-primase to overcome competition from RPA to initiate primer synthesis.

## INTRODUCTION

Following replisome assembly and template unwinding at bi-directional origins of DNA replication, Pol α-primase is recruited to the two advancing replisomes where it primes the lagging-strand template.[1] To start coupled leading-strand replication, these primers are extended across the origin by the main lagging-strand polymerase, Pol δ,[2] before a polymerase switch transfers the nascent strand to the principal leading-strand polymerase, Pol ε.[1,3–6] As replication forks progress primers are synthesized every few hundred nucleotides to support discontinuous lagging-strand replication. If leading-strand synthesis is interrupted due to DNA damage, biochemical reconstitution experiments have demonstrated that *S. cerevisiae* (budding yeast) replisomes continue lagging-strand replication but do not frequently reinitiate leading-strand replication due to a failure to support efficient primer synthesis on this strand.[7–10] Collectively, these observations indicate that the replisome efficiently targets Pol α-primase to the lagging-strand template but not the leading-strand template, which likely explains why some eukaryotes, including humans, encode a second primase-polymerase, PRIMPOL, to restart leading-strand replication.[11–14] Currently the mechanistic basis underlying the preference of Pol α-primase for lagging- rather than leading-strand priming is unknown.

Pol α-primase is a constitutive heterotetramer composed of a dimeric primase (PRIM1 and PRIM2 in *H. sapiens* [human], Pri1 and Pri2 in budding yeast) and a dimeric DNA polymerase (POLA1 and POLA2 in human, Pol1 and Pol12 in budding yeast) (Figure 1A). Primase synthesizes 8–10 nucleotides (nt) of RNA that are transferred to the Pol α DNA polymerase for limited extension to a total primer length about 20–35 nt.[15–18] *In vitro*, the ability of budding yeast[7] and human[19–21] Pol α-primase to initiate primer synthesis on single-stranded DNA (ssDNA) templates is blocked when the template is saturated with RPA, indicating that a mechanism exists to target primase to ssDNA at the eukaryotic replication fork. Consistent with this idea, Pol α-primase interacts with several core components of the replisome including AND-1 (Ctf4 in budding yeast), MCM10, GINS, and CMG.[22–27]

In budding yeast, Pol α-primase is tethered to replisome progression complexes (RPCs) via interaction between its Pol1 subunit and Ctf4, a trimeric scaffold protein that binds directly to CMG.[28–31] Similarly, human Pol α-primase associates with AND-1 but does so primarily via the N-terminal domain (NTD) of POLA2, which binds a C-terminal HMG-box in AND-1.[32,33] However, considerable evidence indicates that AND-1/Ctf4 does not provide a pivotal link between Pol α-primase and the replisome to support priming: Ctf4 is a non-essential protein in budding yeast; disruption of the Pol1:Ctf4 interaction does not result in obvious DNA replication defects *in vivo*[34]; Pol α-primase localizes to replisomes in yeast cells lacking Ctf4[35]; depletion of AND-1 in DT40 cells does not prevent the completion of bulk replication[36]; AND-1 and Ctf4 are dispensable for lagging-strand synthesis in DNA replication reactions reconstituted with purified proteins.[27,37–39] Similarly, reconstituted budding yeast and


This is an open access article under the CC BY license (http://creativecommons.org/licenses/by/4.0/).

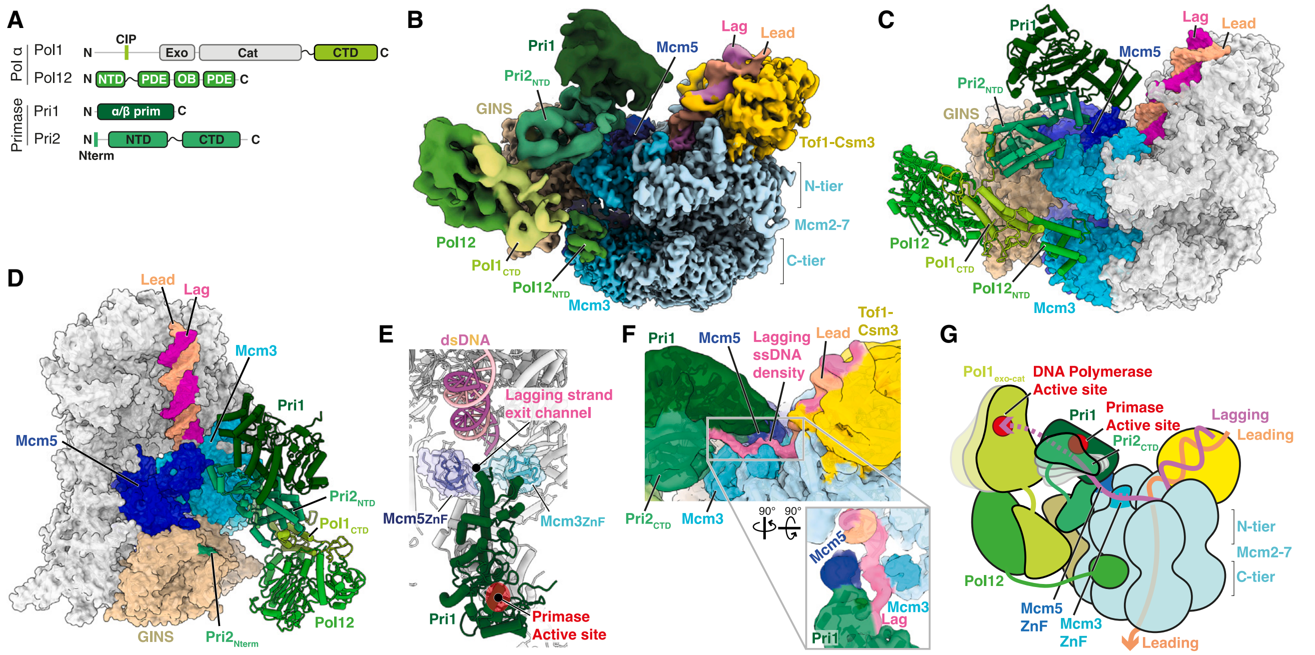


**Figure 1. Structure of Pol α-primase in the budding yeast replisome**

(A) Domain architecture of yeast Pol α-primase. exo, exonuclease domain; cat, catalytic domain; CIP, Ctf4-interacting peptide; NTD, N-terminal domain; CTD, C-terminal domain; PDE, phosphodiesterase domain; OB, oligonucleotide/oligosaccharide-binding domain.

(B) Composite cryo-EM map of the budding yeast Pol α-primase associated replisome bound to replication fork DNA containing a 60 nucleotide 5′ flap. Density for Ctf4 is not observed in this map. The map was derived from combining individual focused refinements and is colored according to chain occupancy.

(C and D) Atomic model of the budding yeast Pol α-primase associated replisome lacking Ctf4 derived from cryo-EM data displayed in (B). Regions of CMG that physically interact with Pol α-primase are colored.

(E) Focused view of the Pri1 catalytic subunit of primase, showing how it is positioned above the exit channel for lagging-strand template ssDNA.

(F) Cryo-EM reconstruction displaying continuous density for lagging-strand template ssDNA extending from the point of dsDNA strand separation toward the active site region of Pri1. Map colored by chain occupancy with the density assigned to the lagging-strand template post-strand separation colored manually.

(G) Schematic illustrating the organization of Pol α-primase in the budding yeast replisome. The path of lagging-strand template ssDNA visualized in the structure immediately following strand separation is illustrated (solid pink line). The putative path of the lagging-strand template between the Pri1 and Pol1 active sites is also illustrated (dashed pink line).

human replisomes perform lagging-strand replication in the absence of MCM10.[27,39,40]

Accumulating evidence suggests that Pol α-primase is recruited to the replisome for priming via direct interactions with CMG. Yeast Pol α-primase can execute lagging-strand replication when functioning only with CMG and RPA.[39] We recently found that minimal human replisomes consisting of CMG, Pol α-primase, Pol ε, CTF18-RFC, PCNA, and RPA support lagging-strand replication and that human Pol α-primase comigrates with CMG in glycerol gradient sedimentation experiments.[27] However, because there are no structures of Pol α-primase in the eukaryotic replisome, we do not know how Pol α-primase binds to CMG, how these putative interactions contribute to lagging-strand DNA replication, how Pol α-primase might overcome competition with RPA for exposed ssDNA, where in the replisome Pol α-primase is positioned, and why Pol α-primase efficiently primes the lagging-strand but not leading-strand template. To address these questions, we have determined cryoelectron microscopy (cryo-EM) structures of budding yeast and human replisomes bound to Pol α-primase and replication fork DNA.

## RESULTS

### Yeast Pol α-primase replisome structure

To assemble budding yeast replisomes associated with Pol α-primase for cryo-EM analysis, CMG was bound to a model replication fork containing a 39 nt 3′ flap, onto which CMG loads, and a 60 nt 5′ ssDNA flap to mimic the unwound lagging-strand template (Figure S1A). Fork-bound CMG was incubated with Pol α-primase and the replisome accessory factors Mrc1 and Tof1-Csm3. Tof1-Csm3 binds to the leading edge of CMG where it engages and stabilizes the parental DNA duplex and fork junction,[30] which we reasoned might be important to aid the visualization of template DNA in cryo-EM reconstructions. After glycerol gradient sedimentation, complexes were isolated containing all replisome and Pol α-primase subunits and used to prepare grids for cryo-EM data collection and analysis (Figures S1 and S2; Table 1).

Three-dimensional (3D) reconstructions revealed well resolved cryo-EM density for CMG and Tof1-Csm3. Multiple additional densities were also apparent extending from the N-tier face of CMG atop Mcm3 and Mcm5 toward the MCM

**Table 1. Cryo-EM statistics**

| | Budding yeast | Budding yeast | Budding yeast | Human | Human |
|---|---|---|---|---|---|
| | replisome:Pol α-primase | replisome:Pol α-primase | replisome:Pol α-primase | replisome:Pol α-primase | replisome:Pol α-primase |
| | 60 nt 5′ flap (–) Ctf4 | 60 nt 5′ flap (+) Ctf4. CIP #1 | 60 nt 5′ flap (+) Ctf4. CIP #2 | 60 nt 5′ flap | 15 nt 5′ flap |
| | (EMDB-16322) | (EMDB-15902) | (EMDB-15902) | (EMDB-15341) | (EMD-15922) |
| | (PDB: 8B9C) | (PDB: 8B9A) | (PDB: 8B9B) | (PDB: 8B9D) | – |
| Data collection and processing | | | | | |
| Magnification | 81,000 × | 81,000× | 81,000× | 81,000× | 81,000× |
| Voltage (kV) | 300 | 300 | 300 | 300 | 300 |
| Electron exposure ($e^{-}$/Å$^2$) | 40.184 | 40.184 | 40.184 | 37.8 | 88.5 |
| Defocus range (μm) | 1.5–3 | 1.5–3 | 1.5–3 | 1.5–3 | 3.5–0.9 |
| Pixel size (Å) (super resoution) | 0.86 | 0.86 | 0.86 | 1.23 | 1.07 |
| Symetry imposed | none | none | none | none | none |
| Movies collected | 12,819 | 12,819 | 12,819 | 7,355 | 6,718 |
| Initial particle images (no.) | 2,003,322 | 2,003,322 | 2,003,322 | 1,535,548 | 724,557 |
| Final particle images (no.) | 100,179 | 54,970 | 44,970 | 174,696 | 258,339 |
| Map resolution (Å) (0.143 FSC threshold) | 3.34 | 3.5 | 3.5 | 3.4 | 3.3 |
| Map resolution range (Å) | 2.8–12 | 2.9–12 | 2.9–12 | 2.8–12 | 3.0–12 |
| Refinement | | | | | |
| Initial model used (PDB code) | PDB: 6SKL | PDB: 6SKL | PDB: 6SKL | PDB: 7PFO | – |
| Model resolution (Å) (0.5 FSC threshold) | 4.1 | 4.2 | 4.2 | 4.1 | – |
| Map sharpening *B* factor (Å$^2$) | −20 to −50 | −20 to −50 | −20 to −50 | −20 to −50 | – |
| Model composition | | | | | |
| Non-hydrogen atoms | 59,119 | 69,886 | 69,866 | 66,207 | – |
| Protein residues | 7,193 | 8,528 | 8,528 | 8,108 | – |
| Ligands | 4 AMP-PNP | 4 AMP-PNP | 4 AMP-PNP | 3 AMP-PNP | – |
| | 4 $Mg^{2+}$, 4 $Zn^{2+}$ | 4 $Mg^{2+}$, 4 $Zn^{2+}$ | 4 $Mg^{2+}$, 4 $Zn^{2+}$ | 3 $Mg^{2+}$, 4 $Zn^{2+}$ | – |
| RMSDs | | | | | |
| Bond lengths (Å) | 0.023 | 0.011 | 0.01 | 0.027 | – |
| Bond angles (°) | 1.913 | 1.396 | 1.101 | 2.66 | – |
| Validation | | | | | |
| MolProbity score | 0.73 | 0.75 | 0.77 | 0.78 | – |
| Clashscore | 0.32 | 0.62 | 0.69 | 0.32 | – |
| Poor rotamers (%) | 0.29 | 0.5 | 0.53 | 0.66 | – |
| Ramachandran plot | | | | | |
| Favored (%) | 97.42 | 97.81 | 97.81 | 97.12 | – |
| Allowed (%) | 2.58 | 2.19 | 2.19 | 2.88 | – |
| Disallowed (%) | 0 | 0 | 0 | 0 | – |

C-tier beside Mcm3 and GINS (Figure 1B). The resolution of these densities was typically lower than for CMG and displayed considerable variability (Figure S1E), indicating large conformational flexibility. Nonetheless, following extensive focused classification and refinement (see Figure S2), these densities could be unambiguously attributed to Pol α-primase (Figures 1B, S1E–S1O, and S3B–S3D), enabling us to construct a model of a DNA engaged yeast replisome encompassing CMG, Tof1-Csm3, several small sections of Mrc1 (Figure S3A) and regions of all four Pol α-primase subunits (Figures 1C and 1D; Video S1). The Pol α-primase model comprises the primase catalytic subunit Pri1 aside from its flexible N and C termini, the N terminus (residues 1–5) and NTD (residues 44–177 and 181–299) of the primase accessory subunit Pri2, the C-terminal domain (CTD) of the Pol α catalytic subunit Pol1 ($Pol1_{CTD}$) (residues 1,271–1,468) and the majority of the Pol α accessory subunit Pol12 (residues 1–79, 203–582, and 604–705) (Figures 1C, 1D, and S3B–S3D).

Pri1 is positioned close to the incoming parental double-stranded DNA (dsDNA) above a channel between the Mcm3 and Mcm5 zinc-finger (ZnF) domains, through which lagging-strand template ssDNA is extruded after strand separation (Figure 1E).[41–44] It is localized to the replisome through its interaction with the Pri2 NTD ($Pri2_{NTD}$), which sits on the periphery of the MCM N-tier straddling Mcm3 and Mcm5 (Figures 1C and 1D). Pol α ($Pol1_{CTD}$ and Pol12) is situated between the CMG N- and C-tiers, close to Mcm3 and GINS (Figures 1C and 1D) and is coupled to primase via an interaction between $Pol1_{CTD}$ and $Pri2_{NTD}$.[45] Pol12 interacts extensively with $Pol1_{CTD}$ and is anchored to the MCM C-tier through an interface involving its flexibly tethered NTD and Mcm3 (Figure 1C). Although Ctf4 was not included in replisome reconstitutions, we identified a 3D class containing both Pol α-primase and Ctf4 (Figure S3E). The presence of Ctf4 likely resulted from endogenous Ctf4 copurifying with CMG due to the extensive interface between the two complexes.[30,31] Comparison of the structures with and without Ctf4 revealed no substantial changes in the conformation of Pol α-primase (Figures S3E and S3F).

Clear densities for the Pol1 exonuclease-catalytic (exo-cat) domain ($Pol1_{exo\text{-}cat}$) and Pri2 CTD ($Pri2_{CTD}$) were not observed in our consensus refinement (Figures 1B and S1E), indicating that neither domain adopts a single stable conformation when Pol α-primase is bound to the replisome. This behavior contrasts with the crystal structure of human apo Pol α-primase, where both domains were well ordered.[46] However, we recovered several rare 3D classes with low-resolution densities of the appropriate shape and volume to accommodate $Pol1_{exo\text{-}cat}$ and $Pri2_{CTD}$, although the precise orientation of each domain could not be assigned (Figures S3G and S3H). In these reconstructions, $Pri2_{CTD}$ is adjacent to Pri1 close to the primase active site, while $Pol1_{exo\text{-}cat}$ sits above $Pri2_{NTD}$ on the periphery of the replisome adjacent to the $Pri2_{CTD}$. This configuration more closely resembles the architecture of human Pol α-primase bound to CST (CTC1-STN1-TEN1) and telomeric ssDNA[47] (Figure S3I), and a very recent structure of a human Pol α-primase elongation complex,[48] than the human Pol α-primase apo structure.[46] This led us to consider that Pol α-primase conformation in the yeast replisome might be modulated by protein-protein interactions and/or DNA engagement. Because DNA binding was heterogeneous across the dataset, we obtained a 3D replisome reconstruction lacking DNA (Figure S3J). Here, the positioning of $Pol1_{exo\text{-}cat}$ and $Pri2_{CTD}$ resembled the human apo crystal structure[46] (Figure S3K), indicating that Pol α-primase undergoes DNA-dependent conformational changes when associated with CMG in the budding yeast replisome.

To further explore the putative DNA engagement state of Pol α-primase, we performed additional classification focusing on regions of primase close to the replication fork junction (Figure S2). Strikingly, this strategy revealed a 3D class with continuous density extending from the parental DNA duplex at the point of strand separation, through the channel between the Mcm3 and Mcm5 ZnF domains and alongside Pri1 in the direction of the primase active site (Figure 1F). The density between the Mcm3 and Mcm5 ZnF domains is in an equivalent position to the previously identified path of the lagging-strand template following strand separation in the human replisome,[41,42] strongly suggesting that it corresponds to lagging-strand template ssDNA. Moreover, the close proximity of the density to Pri1 and its continuation beyond the Mcm3-Mcm5 ZnF channel—which has not been observed in prior human and yeast replisome structures lacking Pol α-primase—indicate that Pri1 engages lagging-strand template ssDNA in the yeast replisome structure. We hypothesize that this configuration functions to ensure a minimal length of ssDNA is required for the lagging-strand template to reach the primase active site, thereby enabling primase to outcompete RPA for access to the template to initiate primer synthesis. Moreover, the positioning of Pri1 and $Pol1_{exo\text{-}cat}$ arranges the primase and DNA polymerase catalytic centers in synthesis order along the template (Figures 1G and S3L), suggesting a possible mechanism for transfer of the RNA primer to the Pol α DNA polymerase as the replisome advances, similar to the mechanism proposed for human Pol α-primase during telomere C strand fill-in.[47]

## Pol α-primase replisome interactions

Four small interaction sites, labeled sites a–d in Figure 2A, tether Pol α-primase directly to CMG and position primase to engage lagging-strand template ssDNA (Video S1). $Pri2_{NTD}$ forms electrostatic interfaces with both the Mcm5 ZnF domain (site a) and the Mcm3 N-terminal helical domain (site b) (Figure 2B). The interface with the Mcm5 ZnF is mediated by a small insertion in $Pri2_{NTD}$ that appears confined to a subset of fungal species (Figure S4A), while the interface with Mcm3 involves three flexible loops within the $Pri2_{NTD}$ (between helices α3-4, α4-5, an α6-7) that are positioned to interact with conserved surface-exposed charged residues on the first alpha helix (α1) of Mcm3 (Figures 2B, 2C, and S4B). 3D variability analysis[49] shows that $Pri2_{NTD}$ adopts a continuum of rotational states with respect to Mcm3 while remaining engaged, likely due to the electrostatic nature of the interface (Figure S4C).

The remaining two interfaces between Pol α-primase and CMG (sites c and d) involve regions of the Pri2 and Pol12 subunits situated at the ends of regions of polypeptide predicted to be unstructured,[50] indicating that they form flexible tethering points (Figures 1A, 1D, 2A, and 2D–2F). The 79 amino acid (aa) Pol12 NTD ($Pol12_{NTD}$) adopts a compact helical fold connected

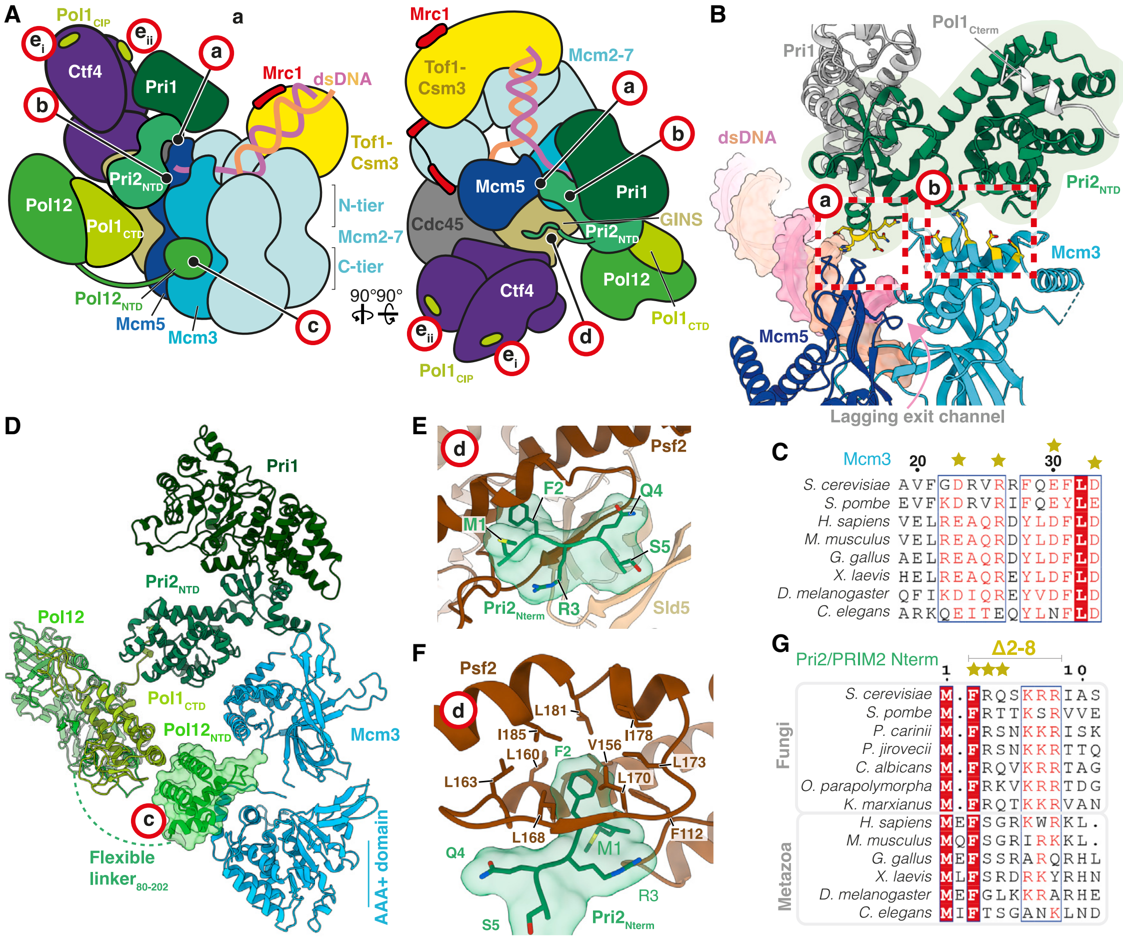


**Figure 2. The structural basis for Pol α-primase recruitment to the budding yeast replisome**

(A) Schematic of the budding yeast replisome highlighting Pol α-primase-binding sites (red circles labeled a–e).

(B) Atomic model highlighting the interfaces between $Pri2_{NTD}$ (green) and the Mcm5 (blue) zinc finger (site a) and Mcm3 (cyan) N-terminal helical domain (site b). Residues colored yellow with side chains displayed represent those targeted for mutational analysis.

(C) Multiple sequence alignment indicating the conservation of Mcm3 residues contacting $Pri2_{NTD}$ (site b), colored according to conservation. Stars correspond to the Mcm3 residues colored yellow in (B) that were mutated.

(D) Atomic model highlighting the interface between the $Pol12_{NTD}$ (green) and the Mcm3 (cyan) AAA+ domain in the MCM C-tier (site c).

(E) Atomic model highlighting the interface between the $Pri2_{Nterm}$ (green) and the Psf2 subunit of GINS (brown) (site d).

(F) Atomic model showing how Pri2-F2 projects into a hydrophobic pocket on Psf2, colored as in (E).

(G) Multiple sequence alignment of $Pri2_{Nterm}$ residues contacting Psf2. The alignment is grouped into fungal and metazoan sequences and colored according to conservation. Stars indicate residues mutated to alanine in the Pri2-AAA mutant.

to the phosphodiesterase (PDE) domain via a 130 aa unstructured linker region. $Pol12_{NTD}$ binds the Mcm3 AAA+ domain in the MCM C-tier (site c), where helices α8 and α18 of Mcm3 form an electrostatic cradle into which α4 of the $Pol12_{NTD}$ docks (Figures 2D and S4D). During ATP-dependent DNA translocation the MCM C-tier adopts multiple conformational states dependent on nucleotide occupancy and DNA engagement.[30,44] While we observe only one C-tier conformation when $Pol12_{NTD}$ is bound to Mcm3, the $Pol12_{NTD}$ can be docked without clashes onto Mcm3 via the same interface through a range of C-tier configurations, indicating it might remain associated with Mcm3 throughout active replication (Figure S4E). Site d involves the N-terminal 5 amino acids of Pri2 ($Pri2_{Nterm}$), where Pri2-F2—invariant in fungal species—docks into a surface-exposed hydrophobic pocket on the GINS subunit Psf2 (Figures 2E–2G). $Pri2_{Nterm}$ is connected to $Pri2_{NTD}$ via a 40 aa linker and, although this linker is predicted to be unstructured, at low map thresholds, continuous density is visible between the last modeled residue of $Pri2_{Nterm}$ (S5) and the first modeled residue of the $Pri2_{NTD}$ (S44), indicating that a section of the linker might adopt a structured conformation (Figure S4F).

In reconstructions containing Ctf4, local refinement revealed the presence of Pol α-primase-dependent density on the surface of the C-terminal α-helical domains of Ctf4 at the previously identified Pol1 binding site,[29] into which the Pol1 Ctf4-interacting peptide (CIP box) ($Pol1_{CIP}$) can be docked (PDB: 4C93)

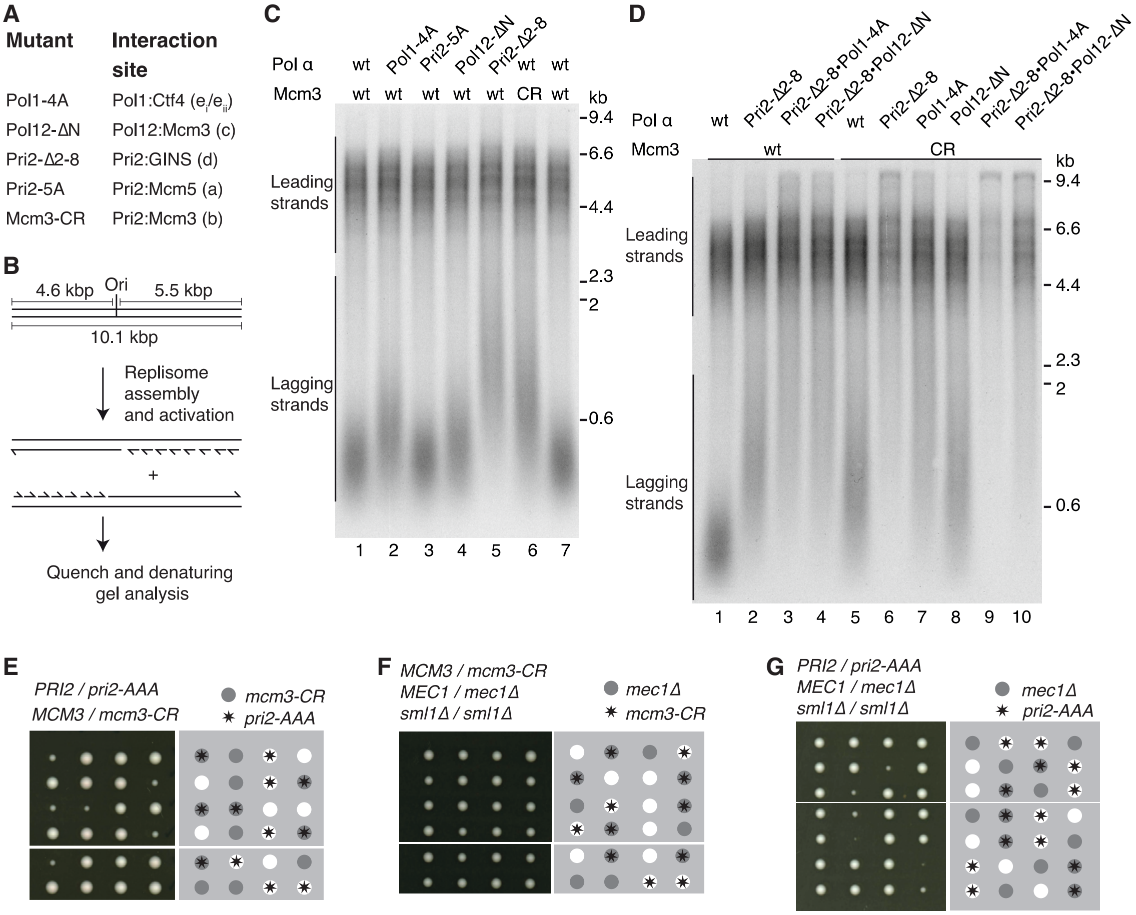


**Figure 3. Pol α-primase CMG binding sites are critical for DNA replication**

(A) Summary of Pol α-primase and Cdt1-Mcm2-7 mutants and the interaction sites that are targeted. CR, charge reversal.

(B) Schematic of the DNA template and anticipated products for origin-dependent budding yeast *in vitro* DNA replication reactions.

(C and D) Denaturing agarose gel analysis of origin-dependent DNA replication reactions performed as illustrated in (B) for 20 min.

(E–G) Diploid budding yeast cells of the indicated genotype were sporulated and the resulting tetrads were dissected and grown on YPD medium for 3 days at 25°C. Dissections that displayed abnormal segregation patterns were cropped from plate images.

(Figures 2A, S4G, and S4H). Although we see no evidence for the presence of multiple copies of Pol α-primase in our dataset, density for $Pol1_{CIP}$ was observed on two Ctf4 monomers (the third monomer is bound to the Sld5 CIP box[30,31]), suggesting mixed occupancy within our reconstructions. Thus, $Pol1_{CIP}$ can bind to either Ctf4 monomer while Pol α-primase is associated with CMG (labeled sites $e_i$ and $e_{ii}$ in Figure 2A), presumably because the CIP box is linked to $Pol1_{exo}$ via ~200 aa of largely unstructured polypeptide.

Although we obtained 3D reconstructions where Pol α-primase was bound to CMG at all 4 sites, a substantial fraction of the dataset lacking Ctf4 displayed binding at just the Mcm5 ZnF and GINS interfaces (sites a and d), demonstrating that only a subset of binding sites are necessary to anchor Pol α-primase to CMG (Figures S4I–S4K). Inspection of the cryo-EM density in reconstructions where only sites a and d were engaged reveals that $Pri2_{NTD}$ and $Pol1_{CTD}$-Pol12 are less well resolved, indicating that these regions are stabilized by the binding of $Pri2_{NTD}$ and $Pol12_{NTD}$ to Mcm3 (sites b and c, respectively). These data indicate that Pol α-primase can utilize only a subset of interaction sites for replisome association, which might be important to permit conformational changes during the priming cycle.[18,46]

## Pol α-primase interaction mutants

To examine the contributions of the Pol α-primase:CMG interfaces during DNA replication, we purified Pol α-primase mutants and truncations designed to disrupt the Pri2:Mcm5 (Pri2-5A), Pol12:Mcm3 (Pol12-ΔN) and Pri2:GINS (Pri2-Δ2-8) interfaces (sites a, c, and d, respectively) and a Cdt1-Mcm2-7 charge reversal (CR) mutant (Mcm3-CR) designed to disrupt the $Pri2_{NTD}$:Mcm3 binding site (site b) (Figures 3A and S5A). We also purified a Pol α-primase complex in which the Pol1 CIP box was mutated to abrogate its interaction with Ctf4[29] (Pol1-4A) (targeting sites $e_i$ and $e_{ii}$) (Figures 3A and S5A). Figure S5B shows that all Pol α-primase mutants displayed similar priming and DNA synthesis activities to the wild-type protein on ssDNA templates. Origin-dependent DNA replication reactions that

CellPress
OPEN ACCESS

generate leading- and lagging-strand products were reconstituted with purified budding yeast proteins on a 10.1 kbp linear DNA template with the origin positioned roughly at its center (Figure 3B).[1,37,38] In reconstituted replication reactions in which the lagging-strand maturation machinery is omitted, the length distribution of lagging-strand products is dependent on Pol α-primase concentration, with less frequent priming resulting in the synthesis of longer lagging strands.[37,39,51]

Replication with wild-type proteins produced a population of ~4.5–6.5 kb leading strands and lagging strands of less than 0.6 kb (Figure 3C, lanes 1 and 7). Figure 3C shows that all mutant proteins with a single binding site targeted were competent for leading and lagging-strand DNA replication, demonstrating that no single interface is essential for Pol α-primase function. However, the distribution of lagging-strand products varied considerably among mutants, indicating that each interface does not contribute equally to productive primer synthesis. Surprisingly, mutations designed to target the Pri2:Mcm5 interface (Pri2-5A, site a) did not affect the length of lagging-strand products (Figure 3C, lane 3). Disruption of the Pol12:Mcm3 (Pol12-ΔN, site c) and Pol1:Ctf4 (Pol1-4A, sites $e_i/e_{ii}$) binding sites resulted in slightly longer lagging-strand products than reactions containing wild-type Pol α-primase under these conditions (Figures 3C, lanes 1, 2, and 4 and S5C), indicating that these interfaces make relatively minor contributions to primer synthesis in the replisome. Strikingly, there was a marked lengthening of lagging-strand products when the Pri2:Mcm3 (Mcm3-CR, site b) and Pri2:GINS (Pri2-Δ2-8, site d) interfaces were perturbed (Figure 3C, lanes 5 and 6). Loss of the Pri2:GINS interaction had the most pronounced effect, with lagging-strand products displaying a broad length distribution of between 0.6 and 2 kb (Figure 3C, lane 5). These data indicate that the Pri2:GINS (site d) and Pri2:Mcm3 (site b) interfaces are the most important interaction sites for priming in the budding yeast replisome.

To gain further insight into the hierarchy of Pol α-primase:replisome interactions during DNA replication, we purified additional complexes harboring combinations of mutations/truncations (Figures S5A and S5B). Figures 3D, S5C, and S5D show that, in almost all cases, disrupting multiple Pol α-primase binding sites resulted in further increases in the length of lagging-strand products. Notably, lagging-strand synthesis was all but abolished in a reaction where both the Pri2:Mcm3 and Pri2:GINS interfaces (sites b and d) were disrupted, and there was a reduction in intensity and subtle lengthening of leading-strand products (Figure 3D, lane 6), which is indicative of delayed synthesis of the primers used to start leading-strand replication.[1] Leading-strand replication was further compromised in reactions where the Pri2:Mcm3, Pri2:GINS, and Pol1:Ctf4 interfaces (sites b, d, and $e_i/e_{ii}$) were targeted simultaneously (Figures 3D, lane 9 and S5D, lane 7). Similar defects were observed when the Pri2:GINS interface was disrupted together with both Pol12:Mcm3 and Pol1:Ctf4 (Figure S5D, lane 5) and when the four sites that individually contribute to DNA replication were simultaneously disrupted (Figure S5D, lane 10). In contrast, we observed robust leading-strand replication and some long lagging-strand products when the Pri2:Mcm3, Pol12:Mcm3, and Pol1:Ctf4 interfaces were simultaneously targeted (Figures S5C, lane 8 and S5D, lane 9), indicating that the interaction between $Pri2_{Nterm}$ and GINS is sufficient to support the necessary priming to start leading-strand replication. These data demonstrate that four distinct interfaces between Pol α-primase and the replisome contribute to nascent-strand priming and that collectively they are essential for efficient *in vitro* DNA replication. Importantly, the data also indicate that the contribution of each interface is not equal: disruption of the interface between $Pri2_{Nterm}$ and GINS (site d) is most deleterious for lagging-strand replication followed by the interface between $Pri2_{NTD}$ and Mcm3 (site b), whereas the interactions between Pol1 and Ctf4 (sites $e_i/e_{ii}$) and $Pol12_{NTD}$ and Mcm3 (site c) make more minor contributions.

## Pol α-primase mutants *in vivo*

Priming at replication forks is an essential function of Pol α-primase and therefore the key interactions we have identified should be critical for cell growth. To test this, we generated budding yeast strains with mutations targeting the Pri2:GINS (Pri2-AAA) and Pri2:Mcm3 (Mcm3-CR) interfaces (Figure 2A, sites b and d). In the Pri2-AAA allele, amino acids F2, R3, and Q4 are substituted to alanine. Figure S5E shows that Pol α-primase complexes containing Pri2-AAA and Pri2-Δ2-8 displayed almost indistinguishable behavior in *in vitro* replication assays. Colony growth of both *pri2-AAA* and *mcm3-CR* cells was comparable to control cells, indicating that priming was occurring at sufficient levels to permit relatively normal DNA replication (Figure S5F). We therefore combined the *pri2-AAA* and *mcm-CR* mutations. This resulted in a profound reduction in colony size relative to control cells, consistent with these cells having DNA replication defects (Figures 3E and S5G), which is concordant with the near absence of lagging-strand products in *in vitro* reactions when these interfaces are disrupted (Figures 3D, lane 6 and S5E, lanes 5 and 6).

Although the lack of obvious growth defects for *pri2-AAA* and *mcm3-CR* cells was somewhat surprising, previous work has shown that budding yeast are reasonably tolerant of reduced Pol α-primase levels.[52] Moreover, colony growth of *pol1-F1463A* cells in which the interaction between primase and the Pol1 C terminus is disrupted, was comparable to control cells.[45] However, *pol1-F1463A* is synthetic lethal with deletion of the gene encoding the apical checkpoint kinase Mec1, the ortholog of ATR, indicating that these cells do in fact have DNA replication defects.[45] We therefore wondered if *pri2-AAA and mcm3-CR* might have subtle DNA replication defects that render cells dependent on checkpoint activation. Figure 3F shows that deletion of *MEC1* in combination with *mcm3-CR* had minimal effect on colony growth. In contrast there was a notable reduction in colony size when *mec1Δ* was combined with *pri2-AAA* (Figure 3G), revealing that tethering of Pol α-primase to CMG via the Pri2:GINS interface is essential for unperturbed DNA replication in budding yeast.

## Human Pol α-primase replisome structure

Because priming is fundamental for genome duplication, we considered it likely that key features of the mechanism targeting Pol α-primase to prime DNA synthesis were conserved. To examine this directly we determined the cryo-EM structure of a

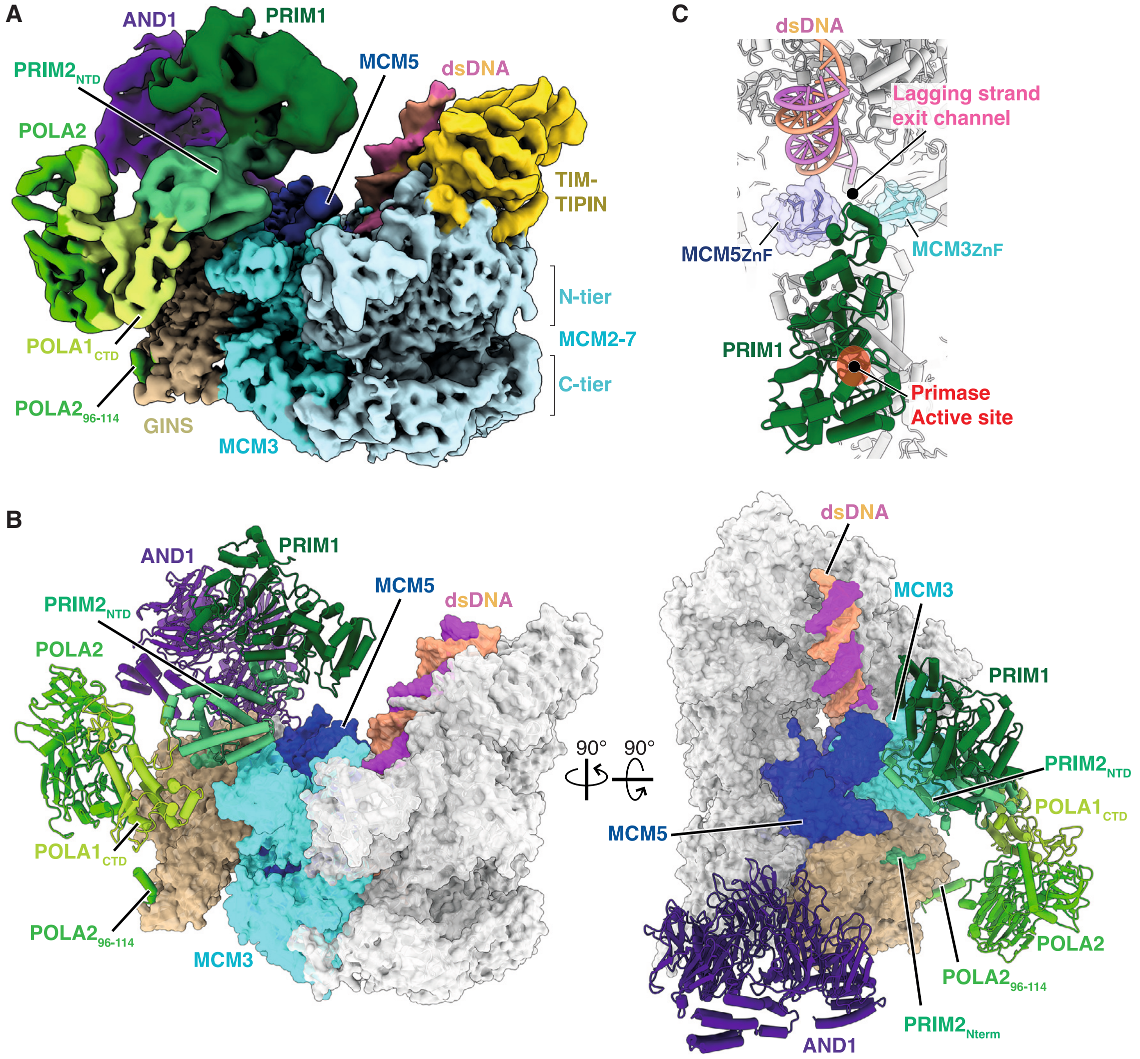


**Figure 4. Structure of Pol α-primase in a human replisome assembled on fork DNA with a 60-nt 5′ flap**

(A) Composite cryo-EM map of the human replisome containing Pol α-primase, assembled on forked DNA containing a 60 nucleotide 5′ flap (Figure S1A). The map was derived from combining individual focused refinements and is colored according to chain occupancy.

(B) Atomic model for the human Pol α-primase associated replisome, derived from cryo-EM data displayed in (A). Regions of CMG that interact directly with Pol α-primase are colored.

(C) Focused view of PRIM1 showing its position at the mouth of the exit channel for lagging-strand ssDNA.

human replisome containing CMG, TIMELESS-TIPIN, AND-1, CLASPIN, Pol α-primase and a DNA replication fork with a 60 nt 5′ ssDNA flap (Figures S6A–S6D; Table 1). Similar to the yeast replisome, in addition to well resolved cryo-EM density for CMG, TIMELESS-TIPIN, and AND-1, poorly resolved density extended from the N-tier face of CMG atop MCM3 (Figures S6E–S6J). Following focused classification and refinement (Figure S6C) this density could be unambiguously assigned to Pol α-primase enabling assignment of PRIM1 (residues 9–349 and 386–408), the N terminus (residues 1–5) and NTD (residues 17–252) of PRIM2, the CTD of POLA1 (residues 1,279–1,445 and 1,448–1,458) and the majority POLA2 (residues 96–114 and 170–598) (Figures 4A, 4B, and S7A–S7C; Video S2).

The conformation of human Pol α-primase and its positioning in the replisome are remarkably similar to yeast (Figures S7D and S7E), with the catalytic PRIM1 subunit again positioned above the mouth of the lagging-strand template exit channel (Figures 4A–4C). Similar to yeast, $POLA1_{exo\text{-}cat}$ is invisible when CMG is bound to replication fork DNA but is visualized stably engaging Pol α-primase in reconstructions lacking DNA, where it adopts the conformation observed for apo human Pol α-primase[46] (Figures S7F and S7G). This suggested that the conformation of Pol α-primase in the human replisome might represent a DNA engaged state. To investigate this further, we repeated our human replisome sample preparation and cryo-EM analysis as before, but with a replication fork containing a

CellPress
OPEN ACCESS

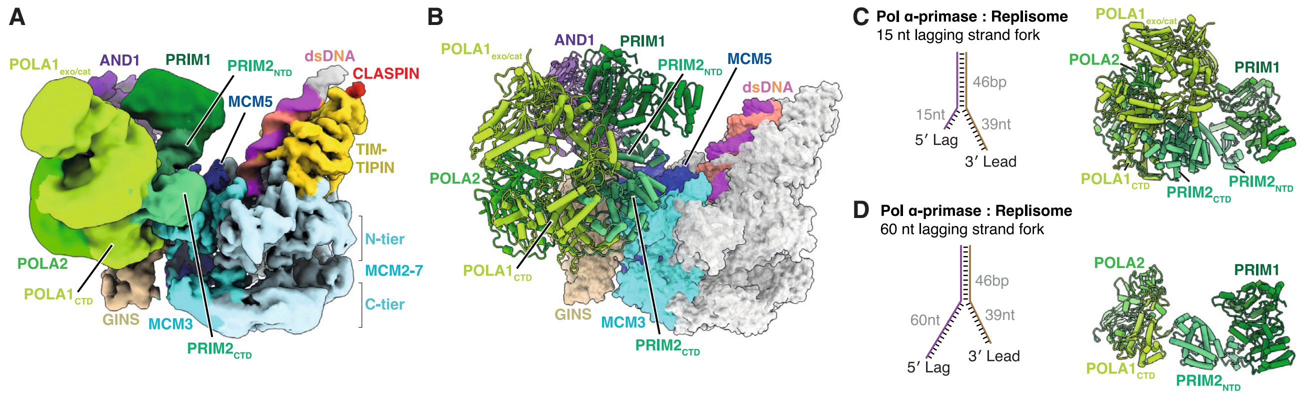


**Figure 5. Structure of Pol α-primase in a human replisome assembled on fork DNA with a 15-nt 5′ flap**

(A) Cryo-EM reconstruction of the Pol α-primase associated human replisome engaged on a DNA fork containing a 15 nucleotide 5′ flap. Map colored according to subunit occupancy.

(B) Atomic model for the human Pol α-primase associated replisome, derived from cryo-EM data displayed in (A). Regions of CMG that interact directly with Pol α-primase are colored.

(C and D) Comparison of Pol α-primase from human replisomes bound to forked DNA with a 15 nt 5′ flap (C) and 60 nt 5′ flap (D) as illustrated.

15 nt 5′ ssDNA flap that we reasoned would be too short to fully engage Pol α-primase (Figures S8A–S8H; Table 1). In the resulting 3D reconstructions, clear density is observed for the $\text{POLA1}_{\text{exo-cat}}$ domain, both when CMG is bound to the replication fork and when CMG is not engaging DNA (Figures 5, S8I, and S8J). In both situations, Pol α-primase adopts the same conformation as observed for the human apo structure,[46] strongly suggesting that Pol α-primase is bound to lagging-strand template ssDNA when the human replisome is assembled on a replication fork with a 60 nt 5′ flap.

## Conservation of Pol α-primase tethering

Human Pol α-primase is tethered directly to CMG via three small interfaces that are all occupied independently of DNA engagement state (Figures 6A–6D and S9A–S9C; Video S2). PRIM2 binds to MCM3 (site b) and GINS (site d) in an analogous manner to Pri2 in the budding yeast replisome (Figures 6B–6D, S9D, and S9E), consistent with these binding sites being the most important for priming in yeast (Figures 3C and 3D). The PRIM2:MCM3 interface involves charged residues on α4 and the α3-4 linker of the $\text{PRIM2}_{\text{NTD}}$ that form electrostatic contacts with four conserved residues on α1 of MCM3 (Figures 2C, 6B, and S9D). Binding of PRIM2 to GINS is mediated by the N terminus of PRIM2, with amino acids M1 and F3—invariant in Metazoa—projecting into a surface-exposed hydrophobic pocket on PSF2 in a comparable manner to Pri2-F2 in yeast (Figure 6D). Continuous cryo-EM density links the last modeled residue of $\text{PRIM2}_{\text{Nterm}}$ (G5) and the first modeled residue of $\text{PRIM2}_{\text{NTD}}$ (Q17) indicating this interface spatially constrains the position of the $\text{PRIM2}_{\text{NTD}}$ and PRIM1 (Figure S9E). In addition to sites b and d, a flexibly tethered helix in POLA2 (residues 96–114) interacts with PSF1 and SLD5 (site g, Figures 6A, 6C, and S9F). Although this helix is predicted to be absent from Pol12, we note the presence of low-resolution Pol α-primase-dependent density on the surface of Psf1 in our budding yeast replisome maps, suggesting a similar binding site could be present in the yeast replisome (Figure S9G). We also note that binding of the POLA2 helix to PSF1 and SLD5 will localize the POLA2 NTD—that binds to the C-terminal AND-1 HMG-box[32] (labeled site f in Figure 6A)—to this region of the replisome because the POLA2 helix and NTD are separated by a short (12 aa) linker. In contrast to the budding yeast replisome, we find no evidence of $\text{PRIM2}_{\text{NTD}}$ binding to the MCM5 ZnF, indicating it is not a conserved mode of interaction and perhaps explaining why the yeast Pri2-5A mutant did not display a lagging-strand replication defect (Figure 3C). Finally, consistent with reports that AND-1 binds the unstructured POLA1 N terminus (residues 151–171),[32,33] we observe a small region of Pol α-primase dependent density on the C-terminal α-helical domain of each AND-1 monomer (labeled sites $e_{\text{i–iii}}$, Figures S9H and S9I), indicating that Pol1 can access all available binding sites on the AND-1 trimer.

The conservation of the PRIM2:GINS and PRIM2:MCM3 interfaces (Figure 6E) suggested they would be important for priming in the human replisome. To test this directly we purified a Pol α-primase complex lacking the PRIM2 N terminus (PRIM2-Δ2-7) and a CMG complex where four conserved residues on helix α1 of MCM3 were mutated to alanine (MCM3-4A) (Figures S9J–S9L) and analyzed them in an *in vitro* human DNA replication system that we recently developed (Figure 6F).[27] Here, replisomes assembled around purified CMG at model replication forks perform leading and lagging-strand DNA replication at rates comparable to those measured in cultured human cells. Figures 6G and S9M show that lagging-strand products distributed around ~0.6 kb in length were synthesized with wild-type proteins. These products were substantially longer when the PRIM2:GINS interface (PRIM2-Δ2-7) was disrupted (Figure 6G, lanes 2 and 6). While disruption of the PRIM2:MCM3 (MCM3-4A) interface was less severe, there was still a notable increase in the length of lagging-strand products, which were longer compared with when the $\text{AND-1}_{\text{HMG}}$:POLA2 interface was abolished (AND-1-ΔHMG) (Figures 6G and S9M). Disruption of multiple interfaces in the same reaction further compromised lagging-strand replication

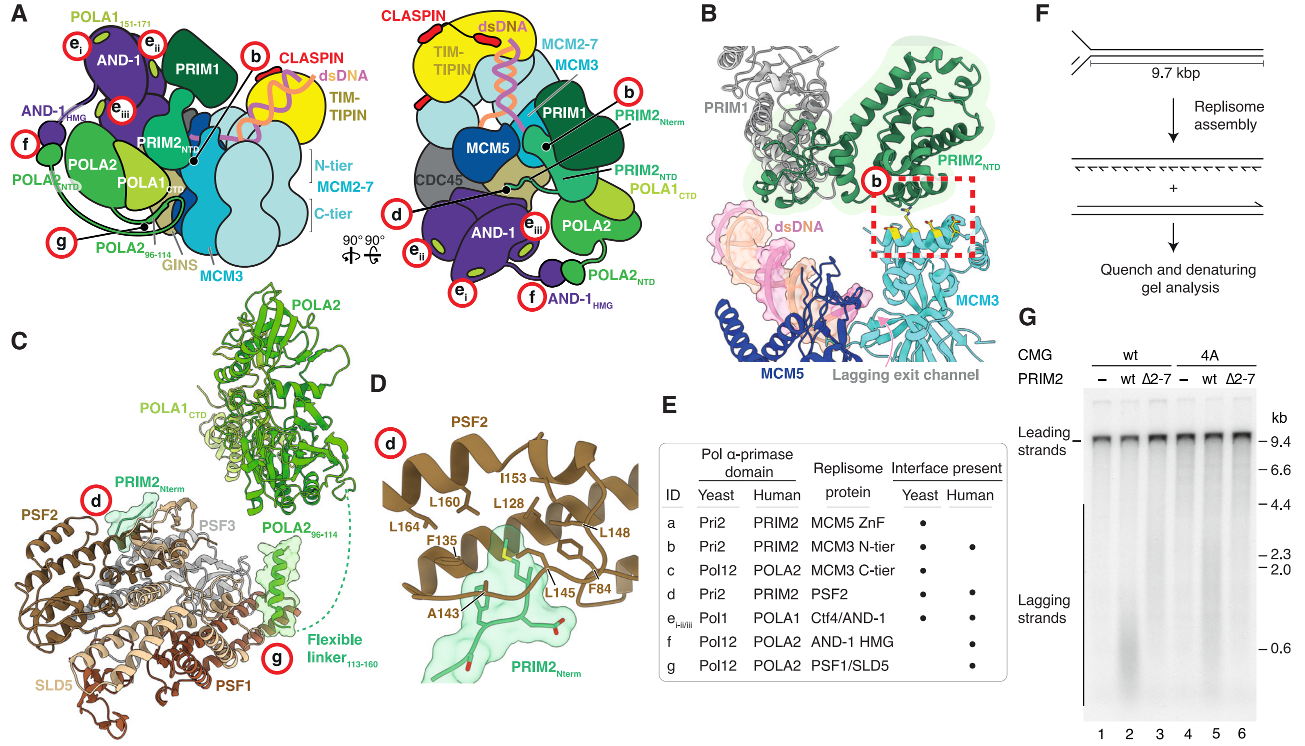


**Figure 6. Structural basis for Pol α-primase recruitment to the human replisome for priming**

(A) Schematic of the human replisome engaged by Pol α-primase. Red circled labels indicate protein-protein interaction sites between Pol α-primase and the replisome.

(B) Atomic model highlighting the interface between $PRIM2_{NTD}$ (green) and the MCM3 (cyan) N-terminal helical domain (site b). Residues colored yellow with side chains displayed are those targeted for mutational analysis.

(C) Atomic model highlighting the interfaces between $PRIM2_{Nterm}$ and the PSF2 subunit of GINS (site d) and the POLA2 N-terminal helix (residues 96–114) and both PSF1 and SLD5 (site g).

(D) Zoomed in view of the $PRIM2_{Nterm}$:PSF2 interface (site d).

(E) Table summarizing the protein-protein interfaces between Pol α-primase and the replisome in both budding yeast and human. Each discrete site is assigned a letter identifier corresponding to the labeling in (A) and Figure 2A.

(F) Schematic of the forked DNA template and anticipated products of *in vitro* DNA replication with purified human proteins.[27]

(G) Denaturing agarose gel analysis of an *in vitro* DNA replication reaction performed as in (A) with the indicated proteins for 20 min.

compared with single site mutants (Figure S9M). Collectively, these data indicate that key anchor points for attaching Pol α-primase to CMG to facilitate primer synthesis at replication forks are structurally and functionally conserved between yeast and human.

## DISCUSSION

By determining cryo-EM structures of budding yeast and human replisomes that are poised to initiate primer synthesis, we have elucidated a conserved mechanism for targeting Pol α-primase to replication forks for priming. The positioning of the catalytic Pri1/PRIM1 subunit at the mouth of the exit channel for lagging-strand template ssDNA explains how Pol α-primase functions so efficiently on this template strand and reveals a mechanism for primase to overcome competition with RPA for access to the DNA template. By contrast, the unwound leading-strand template exits CMG ~150 Å away from Pri1/PRIM1 on the opposite side of the replisome, which is presumably not conducive for leading-strand priming, thus explaining why the core yeast replisome cannot efficiently restart leading-strand replication by repriming downstream of DNA damage[7,8] or secondary structures[10] and why lagging-strand primers are used to start leading-strand replication.[1,3,4]

Pol α-primase is targeted to the lagging-strand template for priming via a complex multisite interaction network involving several direct interactions with CMG. These interactions explain why Pol α-primase tethering by Ctf4/AND-1 is dispensable for DNA replication.[27,34,36,37] The primary function of Ctf4/AND-1-dependent tethering of Pol α-primase is to facilitate the transfer of parental histones to the lagging strand.[34,53] It will be interesting to discover why Ctf4/AND-1-dependent tethering is required for this activity and why the interactions between Pol α-primase and CMG that we have identified cannot fulfill this role. In both the yeast and human replisomes, the majority of Pol α-primase docking sites—including the crucial interaction between Pri2/PRIM2 and GINS—are mediated by regions of Pol α-primase situated at the end of, or within, unstructured

linker regions, thereby providing flexible tethering points. Of the interactions that contribute to priming, only binding of the Pri2/PRIM2 NTD to MCM3 involves the association of two large rigid bodies. However, this interface is frequently disengaged and, due to its electrostatic nature, permits considerable motion between the two domains. This suggests that, although the positioning of Pol α-primase at the mouth of the lagging-strand template exit channel is crucial for priming, it is also important that primase is not rigidly fixed in this location. We hypothesize that flexible tethering of Pol α-primase in the replisome is required to allow other proteins access to key binding sites on CMG. For example, the E3 ubiquitin ligase that regulates replisome disassembly ($Cul2^{LRR1}$ in human and $SCF^{Dia2}$ in budding yeast) binds across the lagging-strand template exit channel[42] and this binding site is inaccessible when Pri2/PRIM2 is bound to MCM3. Flexible tethering may also function to enable Pol α-primase to remain associated with the replisome while it undergoes conformational changes during the primer synthesis reaction.

Our structures indicate that incorporation of Pol α-primase into the replisome does not induce conformational changes in CMG that are likely to modulate helicase activity. Consequently, concomitant primer synthesis and template unwinding will result in increasing lengths of ssDNA being formed between the primase/DNA polymerase active sites and the point of template unwinding, thereby generating what has been termed a "priming loop."[54,55] Currently, we do not know whether Pol α-primase remains fully engaged with CMG throughout the priming reaction, or whether the multiple docking sites are utilized dynamically. We consider it likely that Pol α-primase remains associated with CMG via at least one docking site for the entirety of the priming cycle given the prolonged replisome association kinetics that have been observed in single molecule experiments.[35,56] The conformational dynamics of Pol α-primase and its utilization of docking sites during primer synthesis are interesting subjects for future investigation that we anticipate will also influence the disposition of priming loops in the replisome.

Considerable recent progress has been made in delineating the mechanisms of primer synthesis including the molecular basis for DNA primer initiation,[57] how Pol α-primase activity is coordinated by the CST complex during telomeric C strand fill-in.[47,58,59] Our work represents another important advance by revealing a conserved mechanism for targeting Pol α-primase to replisomes to prime eukaryotic DNA replication and also provides a platform to visualize additional key intermediates during this fundamental process.

### Limitations of the study

Our structures of budding yeast and human Pol α-primase bound to the replisome likely only represent a small subset of conformations that Pol α-primase adopts during the priming cycle. Moreover, while our data strongly support the conclusion that Pol α-primase is engaging ssDNA in both the yeast and human replisomes, it is not possible to determine precisely which step of the priming cycle the structures represent. Although the structures provide important insights into how Pol α-primase is targeted to the replisome for priming, including identifying key protein:protein interaction sites, additional proteins that were not included in our replisome preparations might also modulate Pol α-primase activity at replication forks. For example, subunits of Pol α-primase have been reported to interact directly with Mcm10, RPA, and Pol δ. Therefore, an important future goal will be to determine structures of more complete replisomes performing lagging-strand replication to visualize intermediates along the primer synthesis pathway, the handoff of primers from Pol α-primase to Pol δ, and gain insights into how proteins such as RPA modulate the activity of Pol α-primase in the context of the replisome.

## STAR★METHODS

Detailed methods are provided in the online version of this paper and include the following:

- KEY RESOURCES TABLE
- RESOURCE AVAILABILITY
  - Lead contact
  - Materials availability
  - Data and code availability
- EXPERIMENTAL MODEL AND STUDY PARTICIPANT DETAILS
- METHOD DETAILS
  - Protein expression and purification
  - TIMELESS-TIPIN purification
  - Preparation of fork DNA for cryo-EM
  - Replisome assembly for cryo-EM
  - Cryo-EM data collection
  - Cryo-EM data processing
  - Human replisome + Pol α-primase + 15 nucleotide 5′-flap DNA fork
  - Cryo-EM model building
  - Multiple sequence alignments
  - AlphaFold and AlphaFold-Multimer
  - Budding yeast primase-polymerase assay on M13mp18 ssDNA
  - Origin-dependent budding yeast DNA replication assay
  - Budding yeast tetrad dissection
  - Human primase-polymerase assay on M13mp18 ssDNA
  - Human DNA replication assays
- QUANTIFICATION AND STATISTICAL ANALYSIS

## SUPPLEMENTAL INFORMATION

Supplemental information can be found online at https://doi.org/10.1016/j.molcel.2023.06.035.

## ACKNOWLEDGMENTS

We thank K. Labib for sharing budding yeast strains. We thank J. Shi for operation of the LMB baculovirus facility; LMB media preparation for budding yeast plates; S. Chen, G. Sharov, G. Cannone, A. Yeates, and B. Ahsan for smooth running of the MRC LMB EM facility; and J. Grimmett, T. Darling, and I. Clayson for maintenance of scientific computing facilities. We are grateful to F. Abid-Ali and members of the Yeeles lab for comments on the manuscript. This work was supported by the MRC as part of UK Research and Innovation (MRC grant MC_UP_1201/12 to J.T.P.Y.).

## AUTHOR CONTRIBUTIONS

Investigation—performed all cryo-EM experiments, data analysis, and model building, methodology—purified proteins, writing – original draft, review & editing, M.L.J.; investigation—performed preliminary DNA replication assays and Pol α-primase:CMG interaction studies, methodology—generated expression vectors and yeast strains and purified proteins, V.A.; investigation—performed human DNA replication assays, methodology—purified proteins, Y.B.; conceptualization, supervision, funding acquisition, investigation—performed biochemistry and genetic experiments, methodology—DNA template preparation, protein purification, yeast strains, writing – original draft, review & editing, J.T.P.Y.

## DECLARATION OF INTERESTS

The authors declare no competing interests.

Received: February 7, 2023
Revised: June 16, 2023
Accepted: June 28, 2023
Published: July 27, 2023

## REFERENCES

1. Aria, V., and Yeeles, J.T.P. (2019). Mechanism of bidirectional leading-strand synthesis establishment at eukaryotic DNA replication origins. Mol. Cell *73*, 199–211.e110. https://doi.org/10.1016/j.molcel.2018.10.019.
2. Nick McElhinny, S.A., Gordenin, D.A., Stith, C.M., Burgers, P.M., and Kunkel, T.A. (2008). Division of labor at the eukaryotic replication fork. Mol. Cell *30*, 137–144. https://doi.org/10.1016/j.molcel.2008.02.022.
3. Garbacz, M.A., Lujan, S.A., Burkholder, A.B., Cox, P.B., Wu, Q., Zhou, Z.X., Haber, J.E., and Kunkel, T.A. (2018). Evidence that DNA polymerase delta contributes to initiating leading strand DNA replication in Saccharomyces cerevisiae. Nat. Commun. *9*, 858. https://doi.org/10.1038/s41467-018-03270-4.
4. Zhou, Z.-X., Lujan, S.A., Burkholder, A.B., Garbacz, M.A., and Kunkel, T.A. (2019). Roles for DNA polymerase δ in initiating and terminating leading strand DNA replication. Nat. Commun. *10*, 3992. https://doi.org/10.1038/s41467-019-11995-z.
5. Daigaku, Y., Keszthelyi, A., Müller, C.A., Miyabe, I., Brooks, T., Retkute, R., Hubank, M., Nieduszynski, C.A., and Carr, A.M. (2015). A global profile of replicative polymerase usage. Nat. Struct. Mol. Biol. *22*, 192–198. https://doi.org/10.1038/nsmb.2962.
6. Pursell, Z.F., Isoz, I., Lundström, E.B., Johansson, E., and Kunkel, T.A. (2007). Yeast DNA polymerase epsilon participates in leading-strand DNA replication. Science *317*, 127–130. https://doi.org/10.1126/science.1144067.
7. Taylor, M.R.G., and Yeeles, J.T.P. (2018). The initial response of a eukaryotic replisome to DNA damage. Mol. Cell *70*, 1067–1080.e12. https://doi.org/10.1016/j.molcel.2018.04.022.
8. Taylor, M.R.G., and Yeeles, J.T.P. (2019). Dynamics of replication fork progression following helicase–polymerase uncoupling in eukaryotes. J. Mol. Biol. *431*, 2040–2049. https://doi.org/10.1016/j.jmb.2019.03.011.
9. Guilliam, T.A., and Yeeles, J.T.P. (2020). Reconstitution of translesion synthesis reveals a mechanism of eukaryotic DNA replication restart. Nat. Struct. Mol. Biol. *27*, 450–460. https://doi.org/10.1038/s41594-020-0418-4.
10. Casas-Delucchi, C.S., Daza-Martin, M., Williams, S.L., and Coster, G. (2022). The mechanism of replication stalling and recovery within repetitive DNA. Nat. Commun. *13*, 3953. https://doi.org/10.1038/s41467-022-31657-x.
11. Guilliam, T.A., Brissett, N.C., Ehlinger, A., Keen, B.A., Kolesar, P., Taylor, E.M., Bailey, L.J., Lindsay, H.D., Chazin, W.J., and Doherty, A.J. (2017). Molecular basis for PrimPol recruitment to replication forks by RPA. Nat. Commun. *8*, 15222. https://doi.org/10.1038/ncomms15222.
12. Schiavone, D., Jozwiakowski, S.K., Romanello, M., Guilbaud, G., Guilliam, T.A., Bailey, L.J., Sale, J.E., and Doherty, A.J. (2016). PrimPol is required for replicative tolerance of G quadruplexes in vertebrate cells. Mol. Cell *61*, 161–169. https://doi.org/10.1016/j.molcel.2015.10.038.
13. García-Gómez, S., Reyes, A., Martínez-Jiménez, M.I., Chocrón, E.S., Mourón, S., Terrados, G., Powell, C., Salido, E., Méndez, J., Holt, I.J., and Blanco, L. (2013). PrimPol, an archaic primase/polymerase operating in human cells. Mol. Cell *52*, 541–553. https://doi.org/10.1016/j.molcel.2013.09.025.
14. Wan, L., Lou, J., Xia, Y., Su, B., Liu, T., Cui, J., Sun, Y., Lou, H., and Huang, J. (2013). hPrimpol1/CCDC111 is a human DNA primase-polymerase required for the maintenance of genome integrity. EMBO Rep. *14*, 1104–1112. https://doi.org/10.1038/embor.2013.159.
15. Pellegrini, L. (2012). The Pol alpha-primase complex. Subcell. Biochem. *62*, 157–169. https://doi.org/10.1007/978-94-007-4572-8_9.
16. Perera, R.L., Torella, R., Klinge, S., Kilkenny, M.L., Maman, J.D., and Pellegrini, L. (2013). Mechanism for priming DNA synthesis by yeast DNA polymerase alpha. eLife *2*, e00482. https://doi.org/10.7554/eLife.00482.
17. Garg, P., and Burgers, P.M.J. (2005). DNA polymerases that propagate the eukaryotic DNA replication fork. Crit. Rev. Biochem. Mol. Biol. *40*, 115–128. https://doi.org/10.1080/10409230590935433.
18. Baranovskiy, A.G., Lisova, A.E., Morstadt, L.M., Babayeva, N.D., and Tahirov, T.H. (2022). Insight into RNA–DNA primer length counting by human primosome. Nucleic Acids Res. *50*, 6264–6270. https://doi.org/10.1093/nar/gkac492.
19. Collins, K.L., and Kelly, T.J. (1991). Effects of T antigen and replication protein A on the initiation of DNA synthesis by DNA polymerase alpha-primase. Mol. Cell. Biol. *11*, 2108–2115.
20. Huang, H., Weiner, B.E., Zhang, H., Fuller, B.E., Gao, Y., Wile, B.M., Zhao, K., Arnett, D.R., Chazin, W.J., and Fanning, E. (2010). Structure of a DNA polymerase alpha-primase domain that docks on the SV40 helicase and activates the viral primosome. J. Biol. Chem. *285*, 17112–17122. https://doi.org/10.1074/jbc.M110.116830.
21. Weisshart, K., Förster, H., Kremmer, E., Schlott, B., Grosse, F., and Nasheuer, H.P. (2000). Protein-protein interactions of the primase subunits p58 and p48 with simian virus 40 T antigen are required for efficient primer synthesis in a cell-free system. J. Biol. Chem. *275*, 17328–17337. https://doi.org/10.1074/jbc.M000717200.
22. Zhu, W., Ukomadu, C., Jha, S., Senga, T., Dhar, S.K., Wohlschlegel, J.A., Nutt, L.K., Kornbluth, S., and Dutta, A. (2007). Mcm10 and And-1/CTF4 recruit DNA polymerase alpha to chromatin for initiation of DNA replication. Genes Dev. *21*, 2288–2299. https://doi.org/10.1101/gad.1585607.
23. Miles, J., and Formosa, T. (1992). Protein affinity chromatography with purified yeast DNA polymerase alpha detects proteins that bind to DNA polymerase. Proc. Natl. Acad. Sci. USA *89*, 1276–1280. https://doi.org/10.1073/pnas.89.4.1276.
24. De Falco, M., Ferrari, E., De Felice, M., Rossi, M., Hübscher, U., and Pisani, F.M. (2007). The human GINS complex binds to and specifically stimulates human DNA polymerase α-primase. EMBO Rep. *8*, 99–103. https://doi.org/10.1038/sj.embor.7400870.
25. Ricke, R.M., and Bielinsky, A.-K. (2004). Mcm10 regulates the stability and chromatin association of DNA polymerase-α. Mol. Cell *16*, 173–185. https://doi.org/10.1016/j.molcel.2004.09.017.
26. Chattopadhyay, S., and Bielinsky, A.K. (2007). Human Mcm10 regulates the catalytic subunit of DNA polymerase-alpha and prevents DNA damage during replication. Mol. Biol. Cell *18*, 4085–4095. https://doi.org/10.1091/mbc.e06-12-1148.
27. Baris, Y., Taylor, M.R.G., Aria, V., and Yeeles, J.T.P. (2022). Fast and efficient DNA replication with purified human proteins. Nature *606*, 204–210. https://doi.org/10.1038/s41586-022-04759-1.
28. Gambus, A., van Deursen, F., Polychronopoulos, D., Foltman, M., Jones, R.C., Edmondson, R.D., Calzada, A., and Labib, K. (2009). A key role for Ctf4 in coupling the MCM2-7 helicase to DNA polymerase alpha within the eukaryotic replisome. EMBO J. *28*, 2992–3004. https://doi.org/10.1038/emboj.2009.226.

29. Simon, A.C., Zhou, J.C., Perera, R.L., van Deursen, F., Evrin, C., Ivanova, M.E., Kilkenny, M.L., Renault, L., Kjaer, S., Matak-Vinković, D., et al. (2014). A Ctf4 trimer couples the CMG helicase to DNA polymerase alpha in the eukaryotic replisome. Nature *510*, 293–297. https://doi.org/10.1038/nature13234.

30. Baretić, D., Jenkyn-Bedford, M., Aria, V., Cannone, G., Skehel, M., and Yeeles, J.T.P. (2020). Cryo-EM structure of the fork protection complex bound to CMG at a replication fork. Mol. Cell *78*, 926–940.e13. https://doi.org/10.1016/j.molcel.2020.04.012.

31. Yuan, Z., Georgescu, R., Santos, R.L.A., Zhang, D., Bai, L., Yao, N.Y., Zhao, G., O'Donnell, M.E., and Li, H. (2019). Ctf4 organizes sister replisomes and Pol alpha into a replication factory. eLife *8*, e47405. https://doi.org/10.7554/eLife.47405.

32. Kilkenny, M.L., Simon, A.C., Mainwaring, J., Wirthensohn, D., Holzer, S., and Pellegrini, L. (2017). The human CTF4-orthologue AND-1 interacts with DNA polymerase alpha/primase via its unique C-terminal HMG box. Open Biol. *7*, 170217. https://doi.org/10.1098/rsob.170217.

33. Guan, C., Li, J., Sun, D., Liu, Y., and Liang, H. (2017). The structure and polymerase-recognition mechanism of the crucial adaptor protein AND-1 in the human replisome. J. Biol. Chem. *292*, 9627–9636. https://doi.org/10.1074/jbc.M116.758524.

34. Evrin, C., Maman, J.D., Diamante, A., Pellegrini, L., and Labib, K. (2018). Histone H2A-H2B binding by Pol $\alpha$ in the eukaryotic replisome contributes to the maintenance of repressive chromatin. EMBO J. *37*. H2A–H2B. https://doi.org/10.15252/embj.201899021.

35. Kapadia, N., El-Hajj, Z.W., Zheng, H., Beattie, T.R., Yu, A., and Reyes-Lamothe, R. (2020). Processive activity of replicative DNA polymerases in the replisome of live eukaryotic cells. Mol. Cell *80*, 114–126.e8. https://doi.org/10.1016/j.molcel.2020.08.014.

36. Abe, T., Kawasumi, R., Giannattasio, M., Dusi, S., Yoshimoto, Y., Miyata, K., Umemura, K., Hirota, K., and Branzei, D. (2018). AND-1 fork protection function prevents fork resection and is essential for proliferation. Nat. Commun. *9*, 3091. https://doi.org/10.1038/s41467-018-05586-7.

37. Yeeles, J.T.P., Janska, A., Early, A., and Diffley, J.F.X. (2017). How the eukaryotic replisome achieves rapid and efficient DNA replication. Mol. Cell *65*, 105–116. https://doi.org/10.1016/j.molcel.2016.11.017.

38. Yeeles, J.T., Deegan, T.D., Janska, A., Early, A., and Diffley, J.F. (2015). Regulated eukaryotic DNA replication origin firing with purified proteins. Nature *519*, 431–435. https://doi.org/10.1038/nature14285.

39. Georgescu, R.E., Schauer, G.D., Yao, N.Y., Langston, L.D., Yurieva, O., Zhang, D., Finkelstein, J., and O'Donnell, M.E. (2015). Reconstitution of a eukaryotic replisome reveals suppression mechanisms that define leading/lagging strand operation. eLife *4*, e04988. https://doi.org/10.7554/eLife.04988.

40. Lõoke, M., Maloney, M.F., and Bell, S.P. (2017). Mcm10 regulates DNA replication elongation by stimulating the CMG replicative helicase. Genes Dev. *31*, 291–305. https://doi.org/10.1101/gad.291336.116.

41. Jones, M.L., Baris, Y., Taylor, M.R.G., and Yeeles, J.T.P. (2021). Structure of a human replisome shows the organisation and interactions of a DNA replication machine. EMBO J. *40*, e108819. https://doi.org/10.15252/embj.2021108819.

42. Jenkyn-Bedford, M., Jones, M.L., Baris, Y., Labib, K.P.M., Cannone, G., Yeeles, J.T.P., and Deegan, T.D. (2021). A conserved mechanism for regulating replisome disassembly in eukaryotes. Nature *600*, 743–747. https://doi.org/10.1038/s41586-021-04145-3.

43. Yuan, Z., Georgescu, R., Bai, L., Zhang, D., Li, H., and O'Donnell, M.E. (2020). DNA unwinding mechanism of a eukaryotic replicative CMG helicase. Nat. Commun. *11*, 688. https://doi.org/10.1038/s41467-020-14577-6.

44. Eickhoff, P., Kose, H.B., Martino, F., Petojevic, T., Ali, F.A., Locke, J., Tamberg, N., Nans, A., Berger, J.M., Botchan, M.R., et al. (2019). Molecular basis for ATP-hydrolysis-driven DNA translocation by the CMG helicase of the eukaryotic replisome. Cell Rep. *28*, 2673–2688.e8. https://doi.org/10.1016/j.celrep.2019.07.104.

45. Kilkenny, M.L., De Piccoli, G., Perera, R.L., Labib, K., and Pellegrini, L. (2012). A conserved motif in the C-terminal tail of DNA polymerase alpha tethers primase to the eukaryotic replisome. J. Biol. Chem. *287*, 23740–23747. https://doi.org/10.1074/jbc.M112.368951.

46. Baranovskiy, A.G., Babayeva, N.D., Zhang, Y., Gu, J., Suwa, Y., Pavlov, Y.I., and Tahirov, T.H. (2016). Mechanism of concerted RNA-DNA primer synthesis by the human primosome. J. Biol. Chem. *291*, 10006–10020. https://doi.org/10.1074/jbc.M116.717405.

47. He, Q., Lin, X., Chavez, B.L., Agrawal, S., Lusk, B.L., and Lim, C.J. (2022). Structures of the human CST-Pol$\alpha$–primase complex bound to telomere templates. Nature *608*, 826–832. https://doi.org/10.1038/s41586-022-05040-1.

48. He, Q., Baranovskiy, A.G., Morstadt, L.M., Lisova, A.E., Babayeva, N.D., Lusk, B.L., Lim, C.J., and Tahirov, T.H. (2023). Structures of human primosome elongation complexes. Nat. Struct. Mol. Biol. *30*, 579–583. https://doi.org/10.1038/s41594-023-00971-3.

49. Punjani, A., and Fleet, D.J. (2021). 3D variability analysis: resolving continuous flexibility and discrete heterogeneity from single particle cryo-EM. J. Struct. Biol. *213*, 107702. https://doi.org/10.1016/j.jsb.2021.107702.

50. Jumper, J., Evans, R., Pritzel, A., Green, T., Figurnov, M., Ronneberger, O., Tunyasuvunakool, K., Bates, R., Žídek, A., Potapenko, A., et al. (2021). Highly accurate protein structure prediction with AlphaFold. Nature *596*, 583–589. https://doi.org/10.1038/s41586-021-03819-2.

51. Kurat, C.F., Yeeles, J.T.P., Patel, H., Early, A., and Diffley, J.F.X. (2017). Chromatin controls DNA replication origin selection, lagging-strand synthesis, and replication fork rates. Mol. Cell *65*, 117–130. https://doi.org/10.1016/j.molcel.2016.11.016.

52. Porcella, S.Y., Koussa, N.C., Tang, C.P., Kramer, D.N., Srivastava, P., and Smith, D.J. (2020). Separable, Ctf4-mediated recruitment of DNA polymerase $\alpha$ for initiation of DNA synthesis at replication origins and lagging-strand priming during replication elongation. PLoS Genet. *16*, e1008755. https://doi.org/10.1371/journal.pgen.1008755.

53. Gan, H., Serra-Cardona, A., Hua, X., Zhou, H., Labib, K., Yu, C., and Zhang, Z. (2018). The Mcm2-Ctf4-Polalpha axis facilitates parental histone H3-H4 transfer to lagging strands. Mol. Cell *72*, 140–151.e3. https://doi.org/10.1016/j.molcel.2018.09.001.

54. Nelson, S.W., Kumar, R., and Benkovic, S.J. (2008). RNA primer handoff in bacteriophage T4 DNA replication: the ROLE OF SINGLE-STRANDED DNA-BINDING PROTEIN AND polymerase ACCESSORY PROTEINS. J. Biol. Chem. *283*, 22838–22846. https://doi.org/10.1074/jbc.M802762200.

55. Pandey, M., Syed, S., Donmez, I., Patel, G., Ha, T., and Patel, S.S. (2009). Coordinating DNA replication by means of priming loop and differential synthesis rate. Nature *462*, 940–943. https://doi.org/10.1038/nature08611.

56. Lewis, J.S., Spenkelink, L.M., Schauer, G.D., Yurieva, O., Mueller, S.H., Natarajan, V., Kaur, G., Maher, C., Kay, C., O'Donnell, M.E., and van Oijen, A.M. (2020). Tunability of DNA polymerase stability during eukaryotic DNA replication. Mol. Cell *77*, 17–25.e5. https://doi.org/10.1016/j.molcel.2019.10.005.

57. Li, A.W.H., Zabrady, K., Bainbridge, L.J., Zabrady, M., Naseem-Khan, S., Berger, M.B., Kolesar, P., Cisneros, G.A., and Doherty, A.J. (2022). Molecular basis for the initiation of DNA primer synthesis. Nature *605*, 767–773. https://doi.org/10.1038/s41586-022-04695-0.

58. He, Y., Song, H., Chan, H., Liu, B., Wang, Y., Sušac, L., Zhou, Z.H., and Feigon, J. (2022). Structure of Tetrahymena telomerase-bound CST with polymerase $\alpha$-primase. Nature *608*, 813–818. https://doi.org/10.1038/s41586-022-04931-7.

59. Cai, S.W., Zinder, J.C., Svetlov, V., Bush, M.W., Nudler, E., Walz, T., and de Lange, T. (2022). Cryo-EM structure of the human CST-Pol$\alpha$/primase complex in a recruitment state. Nat. Struct. Mol. Biol. *29*, 813–819. https://doi.org/10.1038/s41594-022-00766-y.

60. Coster, G., Frigola, J., Beuron, F., Morris, E.P., and Diffley, J.F. (2014). Origin licensing requires ATP binding and hydrolysis by the MCM

replicative helicase. Mol Cell *55*, 666–677. https://doi.org/10.1016/j.molcel.2014.06.034.

61. Frigola, J., Remus, D., Mehanna, A., and Diffley, J.F. (2013). ATPase-dependent quality control of DNA replication origin licensing. Nature *495*, 339–343. https://doi.org/10.1038/nature11920.
62. On, K.F., Beuron, F., Frith, D., Snijders, A.P., Morris, E.P., and Diffley, J.F. (2014). Prereplicative complexes assembled in vitro support origin-dependent and independent DNA replication. EMBO J *33*, 605–620. https://doi.org/10.1002/embj.201387369.
63. Jake, H., Patrik, E., Lucy, S.D., Alessandro, C., and John, F.X.D. (2020). The eukaryotic replisome requires an additional helicase to disarm dormant replication origins. bioRxiv. 2020.2009.2017.301366. https://doi.org/10.1101/2020.09.17.301366.
64. Zivanov, J., Nakane, T., Forsberg, B.O., Kimanius, D., Hagen, W.J., Lindahl, E., and Scheres, S.H. (2018). New tools for automated high-resolution cryo-EM structure determination in RELION-3. eLife *7*, e42166. https://doi.org/10.7554/eLife.42166.
65. Scheres, S.H. (2016). Processing of structurally heterogeneous cryo-EM data in RELION. Methods Enzymol. *579*, 125–157. https://doi.org/10.1016/bs.mie.2016.04.012.
66. Scheres, S.H. (2012). RELION: implementation of a Bayesian approach to cryo-EM structure determination. J. Struct. Biol. *180*, 519–530. https://doi.org/10.1016/j.jsb.2012.09.006.
67. Scheres, S.H. (2012). A Bayesian view on cryo-EM structure determination. J. Mol. Biol. *415*, 406–418. https://doi.org/10.1016/j.jmb.2011.11.010.
68. Punjani, A., Rubinstein, J.L., Fleet, D.J., and Brubaker, M.A. (2017). cryoSPARC: algorithms for rapid unsupervised cryo-EM structure determination. Nat. Methods *14*, 290–296. https://doi.org/10.1038/nmeth.4169.
69. Zheng, S.Q., Palovcak, E., Armache, J.P., Verba, K.A., Cheng, Y., and Agard, D.A. (2017). MotionCor2: anisotropic correction of beam-induced motion for improved cryo-electron microscopy. Nat. Methods *14*, 331–332. https://doi.org/10.1038/nmeth.4193.
70. Rohou, A., and Grigorieff, N. (2015). CTFFIND4: fast and accurate defocus estimation from electron micrographs. J. Struct. Biol. *192*, 216–221. https://doi.org/10.1016/j.jsb.2015.08.008.
71. Pettersen, E.F., Goddard, T.D., Huang, C.C., Couch, G.S., Greenblatt, D.M., Meng, E.C., and Ferrin, T.E. (2004). UCSF Chimera–a visualization system for exploratory research and analysis. J. Comput. Chem. *25*, 1605–1612. https://doi.org/10.1002/jcc.20084.
72. Zivanov, J., Nakane, T., and Scheres, S.H.W. (2019). A Bayesian approach to beam-induced motion correction in cryo-EM single-particle analysis. IUCrJ *6*, 5–17. https://doi.org/10.1107/S205225251801463X.
73. Punjani, A., Zhang, H., and Fleet, D.J. (2020). Non-uniform refinement: adaptive regularization improves single-particle cryo-EM reconstruction. Nat. Methods *17*, 1214–1221. https://doi.org/10.1038/s41592-020-00990-8.
74. Pettersen, E.F., Goddard, T.D., Huang, C.C., Meng, E.C., Couch, G.S., Croll, T.I., Morris, J.H., and Ferrin, T.E. (2021). UCSF ChimeraX: structure visualization for researchers, educators, and developers. Protein Sci. *30*, 70–82. https://doi.org/10.1002/pro.3943.
75. Evans, R., O'Neill, M., Pritzel, A., Antropova, N., Senior, A., Green, T., Žídek, A., Bates, R., Blackwell, S., Yim, J., et al. (2022). Protein complex prediction with AlphaFold-Multimer. https://doi.org/10.1101/2021.10.04.463034.
76. Liebschner, D., Afonine, P.V., Baker, M.L., Bunkóczi, G., Chen, V.B., Croll, T.I., Hintze, B., Hung, L.W., Jain, S., McCoy, A.J., et al. (2019). Macromolecular structure determination using X-rays, neutrons and electrons: recent developments in Phenix. Acta Crystallogr. D Struct. Biol. *75*, 861–877. https://doi.org/10.1107/S2059798319011471.
77. Emsley, P., Lohkamp, B., Scott, W.G., and Cowtan, K. (2010). Features and development of coot. Acta Crystallogr. D Biol. Crystallogr. *66*, 486–501. https://doi.org/10.1107/S0907444910007493.
78. Wingfield, P.T. (2017). N-terminal methionine processing. Curr. Protoc. Protein Sci. *88*, 6.14.1–6.14.3. https://doi.org/10.1002/cpps.29.
79. Ree, R., Varland, S., and Arnesen, T. (2018). Spotlight on protein N-terminal acetylation. Exp. Mol. Med. *50*, 1–13. https://doi.org/10.1038/s12276-018-0116-z.
80. Croll, T.I. (2018). Isolde: a physically realistic environment for model building into low-resolution electron-density maps. Acta Crystallogr. D Struct. Biol. *74*, 519–530. https://doi.org/10.1107/S2059798318002425.
81. Chen, V.B., Arendall, W.B., 3rd, Headd, J.J., Keedy, D.A., Immormino, R.M., Kapral, G.J., Murray, L.W., Richardson, J.S., and Richardson, D.C. (2010). MolProbity: all-atom structure validation for macromolecular crystallography. Acta Crystallogr. D Biol. Crystallogr. *66*, 12–21. https://doi.org/10.1107/S0907444909042073.
82. Sievers, F., and Higgins, D.G. (2014). Clustal Omega, accurate alignment of very large numbers of sequences. In Multiple Sequence Alignment Methods, D.J. Russell, ed. (Humana Press), pp. 105–116. https://doi.org/10.1007/978-1-62703-646-7_6.
83. Robert, X., and Gouet, P. (2014). Deciphering key features in protein structures with the new ENDscript server. Nucleic Acids Res. *42*, W320–W324. https://doi.org/10.1093/nar/gku316.
84. Mirdita, M., Schütze, K., Moriwaki, Y., Heo, L., Ovchinnikov, S., and Steinegger, M. (2022). ColabFold: making protein folding accessible to all. Nat. Methods *19*, 679–682. https://doi.org/10.1038/s41592-022-01488-1.

CellPress
OPEN ACCESS

## STAR★METHODS

### KEY RESOURCES TABLE

| REAGENT or RESOURCE | SOURCE | IDENTIFIER |
|---|---|---|
| **Bacterial and virus strains** | | |
| *E. coli* 5-alpha Competent (High Efficiency) | New England Biolabs | Cat# C2987H |
| *E. coli*: Rosetta™ 2(DE3) strain: $F^-$ *ompT* *hsdS*$_B$($r_B^-$ $m_B^-$) *gal dcm* (DE3) pRARE2 ($Cam^R$) | Novagen / Merck Millipore | Cat# 71400 |
| *E. coli* DH10 EMBacY | Geneva Biotech | https://geneva-biotech.com/product_category/insect-cell-expression/multibac/ |
| **Chemicals, peptides, and recombinant proteins** | | |
| 3X FLAG peptide | Sigma | Cat# F4799 |
| Adenosine 5'-(β,γ-imido)triphosphate lithium salt hydrate (AMP-PNP) | Sigma | Cat# A2647 |
| dNTP set | Invitrogen | Cat# 10297018 |
| NTP set | Invitrogen | Cat# R0481 |
| [alpha-P32]dCTP | Hatmann analytic | Cat# SRP-205 |
| Glutaraldehyde | Sigma | Cat# G5882 |
| Nonidet P-40 substitute (NP-40-S) | Roche | Cat# 11754599001 |
| Glutathione Sepharose 4B | GE Healthcare | Cat# 17-0756-01 |
| Suberic acid bis(3-sulfo-N-hydroxysuccinimide ester) sodium salt ($BS^3$) | Sigma | Cat# S5799 |
| TWEEN® 20 | Sigma | Cat# P8341 |
| Biotin | Sigma | Cat# B4501 |
| cOmplete™, Mini, EDTA-free protease inhibitor cocktail | Sigma | Cat# 11873580001 |
| FuGENE® HD | Promega | Cat# E2311 |
| Insect-XPRESS protein-free insect cell media with L-glutamine | Lonza | Cat# BELN12-730Q |
| Sf-900™ II serum-free media | GIBCO | Cat# 10902-088 |
| SilverQuest™ staining kit | Invitrogen | Cat# LC6070 |
| Bovine Serum Albumin | Invitrogen | Cat# AM2616 |
| Phusion® High-Fidelity DNA Polymerase | New England Biolabs | Cat# E0553 |
| SphI | New England Biolabs | Cat# R0182 |
| SapI | New England Biolabs | Cat# R0569 |
| FspI | New England Biolabs | Cat# R0135 |
| BsaHI | New England Biolabs | Cat# R0556 |
| β-Glucuronidase | Sigma | Cat# G7017 |
| TEV protease | Nagai laboratory | N/A |
| Proteinase K | New England Biolabs | P8107 |
| **Recombinant proteins** | | |
| (see also Table S2) | | |
| Budding yeast (*S. cerevisiae*) | | |
| Cdt1-Mcm2-7 | Coster et al.[60] | N/A |
| ORC | Frigola et al.[61] | N/A |
| Cdc6 | Frigola et al.[61] | N/A |
| DDK | On et al.[62] | N/A |
| Sld3/7 | Yeeles et al.[38] | N/A |

(*Continued on next page*)

| *Continued* | | |
|---|---|---|
| REAGENT or RESOURCE | SOURCE | IDENTIFIER |
| Cdc45 | Yeeles et al.[38] | N/A |
| Dpb11 | Yeeles et al.[38] | N/A |
| Sld2 | Yeeles et al.[38] | N/A |
| GINS | Yeeles et al.[38] | N/A |
| Pol ε | Yeeles et al.[38] | N/A |
| S-CDK | Yeeles et al.[38] | N/A |
| Mcm10 | Yeeles et al.[38] | N/A |
| Pol α | Yeeles et al.[38] | N/A |
| Ctf4 | Yeeles et al.[38] | N/A |
| RPA | Baretić et al.[30] | N/A |
| Mrc1 | Baretić et al.[30] | N/A |
| Tof1-Csm3 | Baretić et al.[30] | N/A |
| RFC | Yeeles et al.[37] | N/A |
| PCNA | Yeeles et al.[37] | N/A |
| Pol δ | Yeeles et al.[37] | N/A |
| CMG | Baretić et al.[30] | N/A |
| Cdt1-Mcm2-7: Mcm3-CR | This study | N/A |
| Pol α-primase: Pol1-4A | This study | N/A |
| Pol α-primase: Pol12ΔN | This study | N/A |
| Pol α-primase: Pol1-4A•Pol12ΔN | This study | N/A |
| Pol α-primase: Pri2-Δ2-8 | This study | N/A |
| Pol α-primase: Pri2-5A | This study | N/A |
| Pol α-primase: Pri2-AAA | This study | N/A |
| Pol α-primase: Pol1-4A•Pri2-Δ2-8 | This study | N/A |
| Pol α-primase: Pol1-4A•Pol12-ΔN•Pri2-Δ2-8 | This study | N/A |
| Pol α-primase: Pol12-ΔN•Pri2-Δ2-8 | This study | N/A |
| Human (*H. sapiens*) | | |
| CMG | Jones et al.[41] | N/A |
| CLASPIN | Jones et al.[41] | N/A |
| TIMELESS-TIPIN | Jones et al.[41] | N/A |
| AND-1 | Jones et al.[41] | N/A |
| Pol ε | Jones et al.[41] | N/A |
| Pol α-primase | Baris et al.[27] | N/A |
| PCNA | Jones et al.[41] | N/A |
| RPA | Jones et al.[41] | N/A |
| Ctf18-RFC | Baris et al.[27] | N/A |
| Pol δ | Baris et al.[27] | N/A |
| Pol α-primase: PRIM2-Δ2-7 | This study | N/A |
| CMG: MCM3-4A | This study | N/A |
| AND1: ΔHMG (Δ1017) | Baris et al.[27] | N/A |
| Deposited data | | |
| Budding yeast (*S. cerevisiae*) | | |
| Co-ordinate file for the Pol α-primase associated replisome in the absence of Ctf4 | This study | 8B9C |
| Co-ordinate file for the Pol α-primase associated replisome in the presence of Ctf4, CIP box site #1 | This study | 8B9A |

(*Continued on next page*)

CellPress
OPEN ACCESS

*Continued*

| REAGENT or RESOURCE | SOURCE | IDENTIFIER |
|---|---|---|
| Co-ordinate file for the Pol α-primase associated replisome in the presence of Ctf4, CIP box site #2 | This study | 8B9B |
| Pol α-primase associated replisome consensus refinement in the absence of Ctf4 (binned) | This study | EMD-16320 |
| Pol α-primase associated replisome consensus refinement in the absence of Ctf4 (un-binned) | This study | EMD-16322 |
| Tof1-Csm3 local refinement | This study | EMD-15304 |
| Mcm2-7 C-tier local refinement | This study | EMD-15305 |
| Pol12, Pol1CTD, Pri2NTD local refinement | This study | EMD-15306 |
| Pol12, Pol1CTD local refinement | This study | EMD-16885 |
| Pol α-primase associated replisome consensus refinement in the presence of Ctf4 (binned) | This study | EMD-15309 |
| Pol α-primase associated replisome consensus refinement in the presence of Ctf4 (un-binned) | This study | EMD-15902 |
| Ctf4 local refinement | This study | EMD-15310 |
| Pri1, Pri2CTD local refinement | This study | EMD-16247 |
| Pol α-primase associated replisome consensus refinement containing density for the Pol1exo/cat and Pri2CTD domains | This study | EMD-16248 |
| Pol α-primase associated replisome consensus refinement containing density for the lagging strand DNA template and the Pri1CTD (binned) | This study | EMD-15924 |
| Pol α-primase associated replisome consensus refinement containing density for the lagging strand DNA template and the Pri1CTD (un-binned) | This study | EMD-15303 |
| Pol α-primase associated replisome consensus refinement where only the Pri2:Mcm5ZnF interface is engaged | This study | EMD-16323 |
| Human (*H. sapiens*) | | |
| Co-ordinate file for the Pol α-primase associated replisome | This study | 8B9D |
| Pol α-primase associated replisome consensus refinement (un-binned) | This study | EMD-15341 |
| MCM2-7 C-tier local refinement | This study | EMD-15340 |
| AND-1 local refinement | This study | EMD-15342 |
| Pol α-primase associated replisome consensus refinement containing strong PRIM1 density (binned) | This study | EMD-15349 |
| PRIM1, POLA2, PolA1CTD, Pri2NTD local refinement | This study | EMD-15351 |
| TIMELESS-TIPIN local refinement | This study | EMD-15356 |
| Composite map assembled from EMD-15342:15341:15340:15349: 15351:15356 | This study | EMD-15904 |
| Pol α-primase associated replisome consensus refinement, not engaged on DNA derived from dataset including a 15 nucleotide 5'-flap DNA fork | This study | EMD-15918 |

(*Continued on next page*)

*Continued*

| REAGENT or RESOURCE | SOURCE | IDENTIFIER |
|---|---|---|
| Pol α-primase associated replisome consensus refinement, not engaged on DNA derived from dataset including a 60 nucleotide 5'-flap DNA fork | This study | EMD-15923 |
| Pol α-primase associated replisome consensus refinement, engaged on a 15 nucleotide 5'-flap DNA fork | This study | EMD-15922 |
| Experimental Models: Cell Lines | | |
| Hi5 | Thermo Fisher | B85502 |
| Experimental models: Organisms/strains | | |
| *S. cerevisiae* strains (See also Table S3 for additional details of strains constructed as part of this study) | | |
| *yAM33 (Cdt1-Mcm2-7 purification)* | Coster et al.[60] | N/A |
| *ySDORC (ORC purification)* | Frigola et al.[61] | N/A |
| *ySDK8 (DDK purification)* | On et al.[62] | N/A |
| *yTD6 (Sld3/7 purification)* | Yeeles et al.[38] | N/A |
| *yTD8 (Sld2 purification)* | Yeeles et al.[38] | N/A |
| *yJY13 (Cdc45 purification)* | Yeeles et al.[38] | N/A |
| *yJY26 (Dpb11 purification)* | Yeeles et al.[38] | N/A |
| *yAJ2 (Pol epsilon purification)* | Yeeles et al.[38] | N/A |
| *yAE88 (S-CDK purification)* | Jake et al.[63] | N/A |
| *yAE95 (Pol alpha purification)* | Jake et al.[63] | N/A |
| *yAE40 (Ctf4 purification)* | Yeeles et al.[38] | N/A |
| *yJY106 (RPA purification)* | Baretić et al.[30] | N/A |
| *yJY32 (Mrc1 purification)* | Yeeles et al.[37] | N/A |
| *yAE48 (Tof1-Csm3 purification)* | Yeeles et al.[37] | N/A |
| *yAE41 (RFC purification)* | Yeeles et al.[37] | N/A |
| *yAE34 (Pol delta purification)* | Yeeles et al.[37] | N/A |
| *yJY197 (CMG purification)* | Jenkyn-Bedford et al.[42] | N/A |
| *yVA87 (Cdt1-Mcm2-7: Mcm3-CR)* | This study | N/A |
| *yVA96 (Pol α-primase: Pol1-4A)* | This study | N/A |
| *yJY239 (Pol α-primase: Pol12-ΔN)* | This study | N/A |
| *yJY232 (Pol α-primase: Pri2-5A)* | This study | N/A |
| *yJY241 (Pol α-primase: Pri2-Δ2-8)* | This study | N/A |
| *yJY242 (Pol α-primase: Pri2-AAA)* | This study | N/A |
| *yJY381 (Pol α-primase: Pol1-4A•Pol12ΔN)* | This study | N/A |
| *yMJ12 (Pol α-primase*: Pol1-4A•Pri2-Δ2-8) | This study | N/A |
| *yMJ13 (Pol α-primase*: Pol1-4A•Pol12-ΔN•Pri2-Δ2-8) | This study | N/A |
| *yMJ18 (Pol α-primase*: Pol12-ΔN•Pri2-Δ2-8) | This study | N/A |
| yJY244 | This study | N/A |
| yJY297 | This study | N/A |
| yJY321 | This study | N/A |
| yJY300 | This study | N/A |
| yJY301 | This study | N/A |
| yJY365 | This study | N/A |
| yJY367 | This study | N/A |
| yJY345 | This study | N/A |
| yJY350 | This study | N/A |
| yJY351 | This study | N/A |

(*Continued on next page*)

CellPress
OPEN ACCESS

***Continued***

| REAGENT or RESOURCE | SOURCE | IDENTIFIER |
|---|---|---|
| yJY356 | This study | N/A |
| yJY357 | This study | N/A |
| yJY255 | This study | N/A |
| yJY302 | This study | N/A |
| yJY326 | This study | N/A |
| yJY328 | This study | N/A |
| yJY313 | This study | N/A |
| yJY315 | This study | N/A |
| yJY317 | This study | N/A |
| yJY352 | This study | N/A |
| yJY354 | This study | N/A |
| Oligonucleotides | | |
| Leading strand: 5'-(Cy3)-TAGAGTAGGAAG TGAGGTAAGTGATTAGAGAATTGGAGAGT GTG(T)34T*T*T*T*T*T – 3' (* - phosphorothioate) | Integrated DNA Technologies (IDT) | N/A |
| 15 Nucleotide 5'-flap lagging strand: 5'-GGCAGG CAGGCAGGCACACACTCTCCAATTCTCTAATCA CTTACCACACTTCCTACTCTA – 3' | Integrated DNA Technologies (IDT) | N/A |
| 60 nucleotide 5'-flap lagging strand: $(T)_{60}$ACACAC TCTCCAATTCTCTAATCACTTACCATCACTTCCT ACTCTA – 3' | Integrated DNA Technologies (IDT) | N/A |
| MT096: 5′phos/GCTATGTGGTAGGA AGTGAGAATTGGAGAGTGTGTTTTT TTTTTTTTTTTTTTTTTTTTTTTTTTTT TTTTTTTGAGGAAAGAATGTTGGTG AGGGTTGGGAAGTGGAAGGATGG GCTCGAGAGGTTTTTTTTTTTTTTTT TTTTTTTTTTTTTTTTTT | Integrated DNA Technologies (IDT) | N/A |
| JY197: 5′- TTTTTTTTTTTTTTTTTTTTTCACA CTCTCCAATTCTCACTTCCTACCACAT | Integrated DNA Technologies (IDT) | N/A |
| JY195: 5′ - CCTCTCGAGCCCATC CTTCCACTTCCCAACCCTCACC | Integrated DNA Technologies (IDT) | N/A |
| JY104: 5′ - GAATTGCGCTCTATGAAGTTGAC | Merck | N/A |
| JY105: 5′ - GAACTGCGGCTTGATAATGG | Merck | N/A |
| JY370: 5′ - GGACTAGGATGAGTAGCAGC | Merck | N/A |
| JY491: 5′ - GAGTCAGACAACCAGCAAGC | Merck | N/A |
| JY604: 5′ - GGTTGAAGAGCAGGCCAAGG | Merck | N/A |
| JY609: 5′ - TCAGGCCAAAGGTGATACGAC | Merck | N/A |
| VA212: 5′ - GACCTGTCGAATTCTCTCAA | Merck | N/A |
| Recombinant DNA | | |
| vVA20 | Aria and Yeeles[1] | N/A |
| M13mp18 ssDNA | New England Biolabs | Cat# N4040S |
| ZN3 | Taylor and Yeeles[7] | N/A |
| pJFDJ5 (yeast GINS purification) | Yeeles et al.[38] | N/A |
| vJY19 (yeast PCNA purification) | Yeeles et al.[37] | N/A |
| pAM3 (yeast Cdc6 purification) | Coster et al.[60] | N/A |
| pET28a-Mcm10 (yeast Mcm10 purification) | Yeeles et al.[38] | N/A |
| YB_X1 (human RPA purification) | This study | N/A |
| MT_EB1 (human PCNA purification) | Jones et al.[41] | N/A |
| YB_2 (human CMG purification) | Jones et al.[41] | N/A |
| YB_1 (human CMG purification) | Jones et al.[41] | N/A |

(*Continued on next page*)

*Continued*

| REAGENT or RESOURCE | SOURCE | IDENTIFIER |
|---|---|---|
| MT_01 (human CMG purification) | Jones et al.[41] | N/A |
| MT_BF1 (AND-1 purification) | Jones et al.[41] | N/A |
| MT_DB1 (CLASPIN purification) | Jones et al.[41] | N/A |
| MT_DF1 (TIMELESS-TIPIN purification) | Jones et al.[41] | N/A |
| MT_BD1 (TIMELESS-TIPIN purification) | Jones et al.[41] | N/A |
| MT_BH1 (human RFC purification) | Jones et al.[41] | N/A |
| MT_BJ1 (human RFC purification) | Jones et al.[41] | N/A |
| MT_BK1 (human RFC purification) | Jones et al.[41] | N/A |
| MT_BL1 (human RFC purification) | Jones et al.[41] | N/A |
| MT_BI1 (human RFC purification) | Jones et al.[41] | N/A |
| YB_7 (CTF18-RFC purification) | Baris et al.[27] | N/A |
| YB_5 (CTF18-RFC purification) | Baris et al.[27] | N/A |
| YB_6 (CTF18-RFC purification) | Baris et al.[27] | N/A |
| YB_4 (CTF18-RFC purification) | Baris et al.[27] | N/A |
| MT_CF1 (human Pol delta purification) | Baris et al.[27] | N/A |
| MT_CH1 (human Pol delta purification) | Baris et al.[27] | N/A |
| YB_3 (human Pol delta purification) | Baris et al.[27] | N/A |
| MT_FC1 (human Pol delta purification) | Baris et al.[27] | N/A |
| MT_BC3 (human Pol alpha-primase purification) | Baris et al.[27] | N/A |
| MT_AE1 (human Pol alpha-primase purification) | Baris et al.[27] | N/A |
| MT_AF1 (human Pol alpha-primase purification) | Baris et al.[27] | N/A |
| MT_AG1 (human Pol alpha-primase purification) | Baris et al.[27] | N/A |
| MT_U2 (human Pol epsilon purification) | Baris et al.[27] | N/A |
| MT_L1 (human Pol epsilon purification) | Baris et al.[27] | N/A |
| MT_M1 (human Pol epsilon purification) | Baris et al.[27] | N/A |
| MT_N1 (human Pol epsilon purification) | Baris et al.[27] | N/A |
| YB_8 (AND-1-ΔHMG purification) | Baris et al.[27] | N/A |
| YB_X2 (MCM3-4A construction) | This study | N/A |
| YB_X3 (CMG: MCM3-4A purification) | This study | N/A |
| vVA62 (CMG: MCM3-4A purification) | This study | N/A |
| vMJ9 (human Pol alpha-primase: PRIM2-Δ2-7) | This study | N/A |
| vVA52 (Cdt1-Mcm2-7: Mcm3-CR strain construction) | This study | N/A |
| vVA58 (yeast Pol alpha-primase: Pol1-4A strain construction) | This study | N/A |
| vJY186 (yeast Pol alpha-primase: Pol12-ΔN) strain construction) | This study | N/A |
| vJY187 (yeast Pol alpha-primase: Pol1-4A•Pol12-ΔN) strain construction) | This study | N/A |
| vJY196 (yeast Pol alpha-primase: Pri2-Δ2-8) strain construction) | This study | N/A |
| vJY183 (yeast Pol alpha-primase: Pri2-5A) strain construction) | This study | N/A |
| vJY199 (yeast Pol alpha-primase: Pri2-AAA strain construction) | This study | N/A |
| vJY177 (construction of Mcm3-CR (Ura3) strain) | This study | N/A |
| vJY206 (construction of Pri2-AAA (Ura3) strain) | This study | N/A |
| **Software and algorithms** | | |
| Chimera (v1.13) | UCSF Resource for Biocomputing, Visualization, and Informatics | https://www.cgl.ucsf.edu/chimera/ |

(*Continued on next page*)

*Continued*

| REAGENT or RESOURCE | SOURCE | IDENTIFIER |
|---|---|---|
| ChimeraX (v1.52) | UCSF Resource for Biocomputing, Visualization, and Informatics | https://www.cgl.ucsf.edu/chimerax/ |
| Coot (v1.0) | Paul Emsley (Medical Research Council Laboratory of Molecular Biology) | https://www2.mrc-lmb.cam.ac.uk/personal/pemsley/coot/ |
| EPU (v2.0) | ThermoFisher Scientific (FEI) | https://www.fei.com/software/epu-automated-single-particles-software-for-life-sciences |
| ESPript (v3.0.7) | Patrice Gouet (Lyon University); Xavier Robert (Centre national de la recherche scientifique) | http://espript.ibcp.fr/ESPript/ESPript/ |
| FIJI (v1.0) | National Institute of Health | https://imagej.net/Fiji/Downloads |
| Gautomatch (v0.53) | Kai Zhang (Medical Research Council Laboratory of Molecular Biology) | https://www.mrc-lmb.cam.ac.uk/kzhang/Gautomatch/ |
| ImageJ (v1.50i) | National Institute of Health | https://imagej.nih.gov/ij/ |
| ISOLDE (v1.4) | Tristan Croll (Cambridge Institute for Medical Research) | https://isolde.cimr.cam.ac.uk/ |
| Phenix (v1.20-4459) | Cambridge University; Duke University; Lawrence Berkeley National Laboratory; Los Alamos National Laboratory | https://www.phenix-online.org/ |
| Photoshop 2020 | Adobe | https://www.adobe.com/uk/products/photoshop.html |
| Prism (v9.0.0) | GraphPad | https://www.graphpad.com/scientific-software/prism/ |
| RELION (v2.1 & v3.1) | Sjors Scheres (Medical Research Council Laboratory of Molecular Biology) | https://www3.mrc-lmb.cam.ac.uk/relion/ |
| MUSCLE | European Molecular Biology Laboratory - European Bioinformatics Institute (EMBL-EBI) | https://www.ebi.ac.uk/Tools/msa/muscle/ |
| cryoSPARC (v3.2, v4.0 & v4.1) | Structura Biotechnology | https://cryosparc.com/updates |
| CTFFIND-4.1 | The Grigorieff Lab | https://grigoriefflab.umassmed.edu/ctffind4 |
| AlphaFold (v2.0) | DeepMind | https://www.deepmind.com/open-source/alphafold |
| AlphaFold-multimer (v2.0) | DeepMind | https://github.com/deepmind/alphafold |
| ColabFold (v1.5.2) | Ovchinnikov & Steinegger Labs | https://github.com/sokrypton/ColabFold |
| Epson Scan 3.9.3.0EN | Seiko Epson Corporation | https://www.epson.co.uk |
| Amersham Typhoon (1.1.0.7) | Cytiva | |
| Other | | |
| Amicon Ultra Centrifugal Filter Units | Millipore | Cat# UFC901096 |
| QUANTIFOIL Copper 400 mesh R2/2 holey carbon TEM grids | Electron Microscopy Sciences | Cat# Q450CR2 |
| HiTrap Blue HP | GE Healthcare | Cat# 17-0412-01 |
| HiTrap DEAE Fast Flow | GE Healthcare | Cat# 17-5055-01 |
| HiTrap Heparin HP | GE Healthcare | Cat# 17-0406-01 |
| HiTrap SP HP | GE Healthcare | Cat# 29-0513-24 |
| HiTrap SP FF | GE Healthcare | Cat# 29-0513-24 |
| IgG Sepharose Fast Flow | GE Healthcare | Cat# 17-0969-01 |
| StrepTactin Superflow high-capacity resin | IBA life sciences | Cat# 2-1208-002 |
| MonoQ PC 1.6/5 | GE Healthcare | Cat# 17-0671-01 |
| MonoS 5/50 GL | GE Healthcare | Cat# 17-5168-01 |

(*Continued on next page*)

| Continued | | |
|---|---|---|
| REAGENT or RESOURCE | SOURCE | IDENTIFIER |
| Ni-NTA Agarose | QIAGEN | Cat# 30210 |
| Superdex 200 Increase 10/300 GL | GE Healthcare | Cat# 28-9909-44 |
| Superose™ 6 Increase 10/300 GL | GE Healthcare | Cat# 29-0915-96 |
| Sepharose 4B | Sigma | Cat# 4B200 |
| Microspin G-50 columns | GE Healthcare | Cat# GE27-5330-02 |
| Anti-FLAG M2 affinity gel | Sigma | Cat# A2220 |
| Bio-Gel HT (Hydrated) Hydroxyapatite | Bio-Rad | Cat# 130-0150 |
| Calmodulin-Sepharose 4B | GE Healthcare | Cat# 17-0529-01 |
| Criterion XT 4-12% Bis-Tris precast gels | BioRad | Cat# 3450124 |
| NuPAGE™ 4-12% Bis-Tris precast gels | Thermo Fisher | Cat# NPO323box |
| Whatman 3 MM paper | Cytivia | Cat# 11895375 |
| BAS-IP MS phosphor screen | Cytivia | Cat# 28956474 |
| Amersham Hyperfilm MP | Cytivia | Cat# 28906842 |

## RESOURCE AVAILABILITY

### Lead contact

Further information and requests for resources and reagents should be directed to and will be fulfilled by the lead contact, Joseph Yeeles (jyeeles@mrc-lmb.cam.ac.uk).

### Materials availability

Budding yeast strains and protein expression plasmids will be made available on request.

### Data and code availability

- Cryo-EM density maps used in model building have been deposited in the Electron Microscopy Data Bank (EMDB) https://www.ebi.ac.uk/pdbe/emdb, under the following accession numbers:

  ***Budding yeast.*** EMD-16320 (binned), EMD-16322 (un-binned), Pol α-primase associated replisome consensus refinement in the absence of Ctf4, 60 nucleotide 5'-flap DNA fork. EMD-15304, Tof1-Csm3 local refinement. EMD-15305, Mcm2-7 C-tier local refinement, conformation II. EMD-15306, Pol12, Pol1$_{CTD}$, Pri2$_{NTD}$ local refinement. EMD-16885, Pol12, Pol1CTD local refinement. EMD-15309 (binned), EMD-15902 (un-binned), Pol α-primase associated replisome consensus refinement in the presence of Ctf4, 60 nucleotide 5'-flap DNA fork. EMD-15310, Ctf4 local refinement. EMD-16247, Pri1, Pri2$_{CTD}$ local refinement. EMD-16248, Pol α-primase associated replisome consensus refinement including density for the Pol1$_{exo/cat}$ and Pri2$_{CTD}$ domains. EMD-15924 (binned), EMD-15303 (un-binned), Pol α-primase associated replisome consensus refinement with continuous density for the lagging strand DNA template extending towards the Pri1 active site and density for the Pri1$_{CTD}$, 60 nucleotide 5'-flap DNA fork. EMD-16323, consensus refinement of Pol α-primase associated with the replisome only via the Pri2:Mcm5$_{ZnF}$ and Pri2$_{Nterm}$:Psf2 interfaces, 60 nucleotide 5'-flap DNA fork.
  ***Human.*** EMD-15341, Pol α-primase associated replisome un-binned consensus refinement, 60 nucleotide 5'-flap DNA fork. EMD-15342, AND-1 local refinement. EMD-15340, MCM2-7 C-tier local refinement. EMD-15351, PRIM1, POLA2, PolA1$_{CTD}$, Pri2$_{NTD}$ local refinement. EMD-15356, TIMELESS-TIPIN local refinement. EMD-15904. EMD-15349, Pol α-primase associated replisome binned consensus refinement, strong PRIM1 density. composite map assembled from EMD-15342: 15341:15340:15349:15351:15356. EMD-15918, Pol α-primase associated replisome consensus refinement, not engaged on DNA derived from dataset including a 15 nucleotide 5'-flap DNA fork. EMD-15923, Pol α-primase associated replisome consensus refinement, not engaged on DNA derived from dataset including a 60 nucleotide 5'-flap DNA fork. EMD-15922, Pol α-primase associated replisome consensus refinement, engaged on a 15 nucleotide 5'-flap DNA fork.
  Atomic coordinates have been deposited in the Protein Data Bank (PDB), http://www.pdb.org, with the following accession numbers:
  **Budding yeast.** 8B9C - Pol α-primase associated replisome in the absence of Ctf4. 8B9A - Pol α-primase associated replisome in the presence of Ctf4, CIP box site #1. 8B9B - Pol α-primase associated replisome in the presence of Ctf4, CIP box site #2.
  ***Human.*** 8B9D – Pol α-primase associated replisome.
  Unprocessed images of the data featured in this manuscript have been deposited at Mendeley Data and are publicly available as of the date of publication (https://doi.org/10.17632/n2wm36mrmw.1).

- This study does not report any original code.
- Any additional information required to reanalyse the data reported in this paper is available from the lead contact upon request.

CellPress
OPEN ACCESS

## EXPERIMENTAL MODEL AND STUDY PARTICIPANT DETAILS

*S. cerevisiae* strains constructed for genetic experiments were based on the W303 genetic background. Comprehensive information regarding the genotypes of these *S. cerevisiae* strains can be found in Table S4.

## METHOD DETAILS

### Protein expression and purification

Details of protein expression plasmids and strains made during this study can be found in Tables S1 and S2. An overview of the purification strategy for each protein is provided in Table S3. All wild type budding yeast proteins were expressed and purified as described previously.[30,37,38,42] Cdt1-Mcm2-7 and Pol α-primase mutants / truncations were purified using the same procedure as for the wild type proteins. Human proteins were expressed and purified as described previously.[27,41,42] Human Pol α-primase (Pri2-Δ2-7) was expressed and purified as described for the wild type protein.[27] Human CMG (MCM3-4A) was expressed by coinfecting Hi5 cells at a density of 1 x $10^6$ cells/ml with four viruses (generated as previously described[42]) expressing: MCM2, MCM5, MCM3-4A (vVA62); MCM7, MCM4, MCM6 (YB_X3); Cdc45 (MT_O1)[41]; PSF1, PSF2, PSF3, SLD5 (YB_1).[41] Cells were grown for 72 hours before harvest by centrifugation. Protein purification was performed as described previously for the wild type protein.[41]

### TIMELESS-TIPIN purification

Cells from a 1L culture were resuspended in lysis buffer (25 mM HEPES-KOH pH 7.2, 150 mM KCl, 5% glycerol, 0.5 mM TCEP, 0.01% NP-40-S) + protease inhibitors (cOmplete, EDTA-free, one tablet per 50 ml buffer) and lysed by dounce homogenization. Insoluble material was cleared by centrifugation (235,000 *g*, 4°C, 45 min) and 0.5 ml Strep-Tactin XT superflow high capacity resin was added to the lysate. Following a 30 min incubation at 4°C the resin was collected in a 20-ml column and was washed with 50 ml lysis buffer. The resin was resuspended in ~2 ml lysis buffer and TEV protease was added to 100 ug/ml. The sample was incubated at 4°C overnight with gentle rotation. The sample was collected and applied to a 1 ml HiTrap Q HP column (GE Healthcare) equilibrated in 25 mM HEPES-KOH pH 7.2, 150 mM KCl, 5% glycerol, 0.5 mM TCEP, 0.01% NP-40-S. Proteins were eluted with a 20 column volume gradient from 150 to 1,000 mM KCl and peak fractions containing TIMELESS-TIPIN were pooled, concentrated to ~500 µl in an Amicon Ultra-15 30 kDa MWCO concentrator and applied to a Superdex 200 Increase 10/300 gel filtration column (GE Healthcare) equilibrated in 25 mM Tris–HCl pH 7.2, 5% glycerol, 0.01% NP-40-S, 1 mM DTT, 150 mM NaCl. Peak fractions were pooled, frozen in liquid nitrogen and stored at −80°C.

### Preparation of fork DNA for cryo-EM

To prepare forked DNA for cryo-EM sample preparation, leading and lagging strand oligonucleotides (Integrated DNA Technologies) were mixed at equimolar ratios in annealing buffer (25 mM HEPES-NaOH, pH 7.5, 150 mM NaOAc, 0.5 mM TCEP, 2 mM $Mg(OAc)_2$) and gradually cooled from 80°C to room temperature. Leading strand oligo: 5′-(Cy3)-TAGAGTAGGAAGTGAGGTAAGTGATT

AGAGAATTGGAGAGTGTG$(T)_{34}$ T*T*T*T*T*T – 3′

* Denotes a phosphorothioate backbone linkage. 15 Nucleotide 5′-flap lagging strand oligo:

5′-GGCAGGCAGGCAGGCACACACTCTCCAATTCTCTAATCACTTACCACACTTCCTACT

CTA – 3′. 60 nucleotide 5′-flap lagging strand sequence:

5′-TTTTTTTTTTTTTTTTTTTTTTTTTTTTTTTTTTTTTTTTTTTTTTTTTTTTTTTTTTTTAC

ACACTCTCCAATTCTCTAATCACTTACCATCACTTCCTACTCTA – 3′

### Replisome assembly for cryo-EM

Reconstitution reactions were set up to yield a final volume of 300 µl, containing 150 nM CMG with a 1.5-fold molar excess of replisome proteins and fork DNA in reconstitution buffer (25 mM HEPES-NaOH pH 7.6, 150 mM NaOAc, 0.5 mM TCEP, 500 µM AMP-PNP, 10 mM $Mg(OAc)_2$). Firstly, CMG was incubated with fork DNA for 10 min on ice in an 80 µl reaction. Next, the additional proteins were added in the following order: Ctf4/AND-1, Tof1-Csm3/TIMELESS-TIPIN, Mrc1/CLASPIN and Pol α-primase. The reaction volume was then adjusted to 300 µl using reconstitution buffer before being incubated for 20 min on ice. Following incubation, 132 µl of the reconstitution reaction was loaded separately onto two 10-30% glycerol gradients, each containing crosslinker. The remaining 36 µl of the reconstitution reaction was diluted in reconstitution buffer to 132 µl and this sample loaded onto a glycerol gradient prepared in the absence of crosslinker. Glycerol gradients were prepared as previously described[30]: Buffer A (40 mM HEPES-NaOH, pH 7.5, 150 mM NaOAc, 0.5 mM TCEP, 10% v/v glycerol, 0.5 mM AMP-PNP and 3 mM $Mg(OAc)_2$) was layered on top an equal volume of Buffer B (Buffer A, except 30% v/v glycerol, 0.16% glutaraldehyde [Sigma] and 2mM bis(sulfosuccinimidyl)suberate ($BS^3$, ThermoFisher Scientific)) in a 2.2 mL TLS-55 tube (Beranek Laborgerate) and gradients made using a gradient-making station (Biocomp Instruments, Ltd.) before cooling on ice. The sample was separated by centrifugation (200,000g, 4°C, 2 h) prior to manual fractionation. SDS-PAGE gel analysis was used to identify two peak fractions from each gradient containing crosslinker (total volume 368 µl) as previously described.[41] These fractions were then buffer exchanged and concentrated prior to being immediately used for cryo-EM grid preparation as previously described.[41]

## Cryo-EM data collection

### *Budding yeast replisome + Pol α-primase + 60 nucleotide 5′-flap DNA fork*

A total of 12,819 raw movies were acquired using a 300 keV Titan Krios microscope (FEI) equipped with a K3 direct electron detector (Gatan) operated in electron counting mode using the EPU automated acquisition software (ThermoFisher) with "Faster Acquisition" mode (AFIS) enabled. A slit width of 20 eV was used for the BioQuantum energy filter. Data were collected in super-resolution mode bin 2 at an effective pixel size of 0.86 Å/pixel over a defocus range of -1.8 to -3.5 μm. Movies were dose-fractionated into 39 fractions over a 4 s exposure, resulting in a total dose of 39.2 $e^{-}$/Å$^2$.

### *Human replisome + Pol α-primase + 60 nucleotide 5′-flap DNA fork*

A total of 7,355 raw movies were acquired using a 300 keV Titan Krios microscope (FEI) equipped with a K3 direct electron detector (Gatan) operated in electron counting mode using the EPU automated acquisition software (ThermoFisher) with "Faster Acquisition" mode (AFIS) enabled. A slit width of 20 eV was used for the BioQuantum energy filter. Data were collected at a pixel size of 0.86 Å/pixel using a defocus range of -2.1 to -3.5 μm. Movies were dose-fractionated into 39 fractions over a 4 s exposure resulting in a total dose of 47.4 $e^{-}$/Å$^2$.

### *Human replisome + Pol α-primase + 15 nucleotide 5′-flap DNA fork*

A total of 6,718 raw movies were acquired using a 300 keV Titan Krios microscope (FEI) equipped with a Falcon III direct electron detector (Thermo) operated in linear mode using the EPU automated acquisition software (ThermoFisher). Data were collected at a pixel size of 1.07 Å/pixel using a defocus range of -0.9 to -3.5 μm. Movies were dose-fractionated into 39 fractions over a 1 s exposure resulting in a total dose of 88.5 $e^{-}$/Å$^2$.

## Cryo-EM data processing

### *Budding yeast replisome + Pol α-primase + 60 nucleotide 5′-flap DNA fork*

The data processing pipeline outlined here is schematised in Figure S2. Data were processed using either RELION-3[64–67] (henceforth referred to as RELION) or cryoSPARC-3[68] (henceforth referred to as cryoSPARC) unless otherwise stated. 12,819 39-fraction movies were aligned and dose-weighted (1.00513 $e^{-}$/Å$^2$/fraction, 5 x 5 patches, 150 Å$^2$ B-factor) using RELION's implementation of a MotionCor2-like program.[69] CTF parameters were estimated using CTFFIND-4.1[70] and 112 poor-quality micrographs excluded from future processing. Particles were picked using RELION's Laplacian-of-Gaussian (LoG) function providing a minimum diameter of 200 Å and maximum of 350 Å. 2,003,322 picked particles were extracted using a box size of 430 Å. During extraction the data were down-sampled to a pixel size of 3.44 Å/pixel. One round of RELION 2D classification was carried out and 1,623,209 particles were selected for further classification. Four successive rounds of RELION 3D classification (regularisation parameter, T = 4), each generating 6 classes, were carried out using a previously obtained map of the budding yeast replisome as a reference (EMD-10227).[30] Class selection was based upon the presence of secondary structure features within CMG. The first two round of 3D classification were performed with a 250 Å diameter circular mask to focus classification on the CMG, with the subsequent two rounds using a more dilated mask of 380 Å to incorporate signal from Pol α-primase.

202,655 particles containing poor density for dsDNA were selected for an additional two rounds of 3D classification in RELION. This resulted in the selection of 44,871 particles in classes displaying density for both Pol α-primase and CMG in the absence of dsDNA. These particles were refined using 3D auto-refinement in RELION generating a reconstruction, after post-processing, at 7.4 Å resolution. In order to enrich for replisomes stably bound by Pol α-primase in the absence of dsDNA, signal subtraction was carried out in RELION focussing on the interface between Pol α-primase and Mcm3. The refined reconstruction was low-pass filtered to 10 Å in UCSF Chimera[71] and a soft mask was generated covering density for both Pol α-primase and the Mcm3 N-terminal helical domain. Subtracted particles were re-centred on the mask and sub-classified using 3D classification without alignment in RELION. 18,412 particles in classes containing strong density for both Pol α-primase and the Mcm3 N-terminal helical domain were selected and reverted to the original (non-subtracted) particles prior to refinement using 3D auto-refine in RELION. This generated a reconstruction, following postprocessing, in RELION at 6.8 Å resolution (Figure S3J).

Returning to the results of the fourth overall round of 3D classification, 884,301 particles were selected for further processing to enrich for replisome complexes bound by Pol α-primase engaged on DNA. These particles were re-extracted using an un-binned pixel size of 0.86 Å in a 450 Å box and submitted for refinement using RELION 3D auto-refine, yielding a reconstruction at 3.5 Å following postprocessing. These data were submitted for two rounds of iterative per-particle motion correction using dataset-trained particle polishing in RELION[72] and RELION CTF-refinement[64] (beamtilt and trefoil correction, anisotropic magnification correction, and per-particle defocus and astigmatism CTF correction). These data were refined to an improved resolution of 3.0 Å following post-processing in RELION. At this stage of processing the density for CMG, Ctf4, Tof1-Csm3 and DNA was of high quality yet the Pol α-primase density was disordered and fragmented with only the $Mcm5_{ZnF}$ contact preserved at appropriate map thresholds.

In order to identify classes in which Pol α-primase was stably engaged with the replisome, the strategy previously described to enrich for Pol α-primase stably engaged on replisomes in the absence of DNA was employed. Signal subtraction was carried out using a mask encompassing both Pol α-primase and the Mcm3 N-terminal helical domain. These subtracted particles were than sub-classified using 3D classification without alignment, resulting in 614,228 particles in classes with improved Pol α-primase density. These data were reverted to the original non-subtracted particles and refined using RELION 3D auto-refine. The subtraction and subclassification process was then iterated to select for 3D classes representing 588,597 particles with improved Pol α-primase density. These particles were imported into cryoSPARC and subsequently down sampled to a pixel size of 1.72 Å/pixel to boost the

signal-to-noise for spatial frequencies describing secondary structure elements. These data were then classified in 3D via heterogeneous refinement using five different replisome reference maps (composition indicated in Figure S2). Classes in which Pol α-primase was only engaged at both the $Mcm5_{ZnF}$ and Psf2 sites were selected representing 434,311 particles. These particles were refined using non-uniform refinement,[73] with a pixel size of 0.86 Å, to 3.0 Å resolution (Figure S1N). To aid interpretation, all non-uniform and local refinements completed in cryoSPARC were subsequently locally filtered using their respective local resolution maps.

Returning to the previous heterogeneous refinement, classes with improved Pol α-primase density representing 140,426 particles were selected and further classified via heterogeneous refinement with six identical 3D references, the results of which are used as the input for processing strategies 1-4:

Strategy 1: Classes representing 84,628 particles were selected based on the presence of both strong Ctf4 and Pol α-primase density. A soft mask was generated in UCSF Chimera[71] covering Pol α-primase and masked 3D classification without alignment carried out using five identical 3D references in cryoSPARC (target resolution 8 Å, initialisation mode PCA). Masks generated for use in cryoSPARC were binarised using the vop_threshold command in UCSF Chimera[71] and a soft padding width applied using the Volume Tools in cryoSPARC (mask softness=5*resolution(Å)/pixel-size(Å)). Classes representing 54,970 particles were selected based upon Pol α-primase $Pri2_{NTD}$ engagement with the Mcm3 N-terminal helical domain. A consensus refinement for these unmasked particles was carried out using non-uniform refinement in cryoSPARC, with an binned pixel size, resulting in a reconstruction at 4.6 Å resolution in which the MCM C-tier adopts conformation II. This process was iterated to generate a reconstruction, with an un-binned pixel size of 0.86, at 3.5 Å resolution (Figure S1M). In this reconstruction Pol α-primase engages the replisome via contacts with $Mcm5_{ZnF}$, Psf2, Mcm3 N-terminal helical domain, Mcm3 $AAA^+$ domain and Ctf4. Reconstructions in which these sites are all engaged (+/- Ctf4) we define as the "fully engaged" complex. Soft masks were generated covering both the visible regions of Ctf4/Cdc45/GINS in the resulting map, and the remainder of the density. These maps were used to subtract the non Ctf4/Cdc45/GINS density from the particle images and subsequently carry out masked local refinement of the Ctf4/Cdc45/GINS region in cryoSPARC. Local refinement was carried out using the default parameters and a fulcrum point defined by the mask centre, resulting in a reconstruction at 3.3 Å resolution for Ctf4/Cdc45/GINS (Figure S1H).

Strategy 2: Classes representing 73,884 particles were selected following heterogeneous refinement displaying strong Pol α-primase density in the absence of Ctf4 with the Mcm2-7 C-tier in conformation II. Masked 3D classification without alignment using ten 3D references was carried out in cryoSPARC, as described in strategy 1, to enrich for classes where the Pol α-primase $Pri2_{NTD}$ is engaged with the Mcm3 N-terminal helical domain. Classes representing 53,964 particles were selected for two non-uniform refinement jobs in cryoSPARC using either a pixel size of 0.86 Å or 2.27 Å, resulting reconstructions at both 3.5 Å and 4.6 Å resolution respectively for the fully engaged complex in the absence of Ctf4 (Figure S1E). Signal subtraction and masked local refinement was carried out in cryoSPARC using a pixel size of 0.86 Å, as described in Strategy 1, focussing on both the Pol12/$Pol1_{CTD}$ (Figure S1I) and Pol12/$Pol1_{CTD}$/$Pri2_{NTD}$ (Figure S1J) regions of the complex, yielding reconstructions at 4.8 Å and 5.0 Å resolution respectively. In order to improve the noisy density adjacent to Pri1, a soft mask covering this region and Pri1 was generated in UCSF Chimera[71] based upon a low-pass filtered map derived from the consensus non-uniform refinement at 3.5 Å resolution. Masked 3D classification of this region (10 classes, target resolution 12 Å) resulted in the selection of 39,555 particles that were unmasked and refined using non-uniform refinement, resulting in a reconstruction at 4.6 Å resolution at a pixel size of 2.27 Å (Figure S1K). The improved resolution in this region resulted in its assignment as the $Pri2_{CTD}$.

Strategy 3: Classes representing 100,178 particles with strong density for both the Mcm2-7 C-tier in conformation II and Pol α-primase regardless of Ctf4 occupancy. These data were refined using non-uniform refinement in cryoSPARC, using a pixel size of 0.86 Å, to a resolution of 3.5 Å. Signal subtraction and masked local refinement was carried out in cryoSPARC using a pixel size of 0.86 Å, as described in Strategy 1, focussing on the Mcm2-7 C-tier yielding a reconstruction at 3.2 Å resolution (Figure S1F).

Strategy 4: Classes representing 84,350 particles with strong density for Tof1-Csm3/dsDNA and Pol α-primase regardless of Ctf4 occupancy. These data were refined using non-uniform refinement in cryoSPARC, using a pixel size of 0.86 Å, to a resolution of 3.4 Å. Signal subtraction and masked local refinement was carried out in cryoSPARC using a pixel size of 0.86 Å, as described in Strategy 1, focussing on Tof1-Csm3/DNA yielding a reconstruction at 3.9 Å resolution (Figure S1G).

In order to enrich for particles in which the $Pri2_{CTD}$ was well resolved, the 588,597 particle subset initially imported into cryoSPARC following processing in RELION was re-processed using a 3D reference obtained from refinement of the 39,555 particle subset containing density for the $Pri2_{CTD}$. Four rounds of iterative heterogeneous refinement were carried out using four 3D references containing $Pri2_{CTD}$ domain density resulting in an 87,540 particle subset. The non-Pol α-primase density was subtracted from these particles and 3D classification without alignment was carried out within a mask encompassing all of the visible regions of Pol α-primase. Classes were selected based on clear $Pri2_{CTD}$ domain density resulting in a 45,111 particle subset. The corresponding un-subtracted particles were then refined using non-uniform refinement to a resolution of 4.6 Å prior to being further classified using 3D variability analysis[49] using 3 modes and a filter resolution of 9 Å. The results were displayed using clustering analysis using 6 clusters and a filter resolution of 9 Å. Inspection of the resulting reconstructions revealed the presence of additional density corresponding to the $Pol1_{exo/cat}$ domain. An additional round of 3D variability analysis was carried out using the same parameters with a new mask additionally encompassing the $Pol1_{exo/cat}$ domain density. This procedure identified a subset of 9,633 particles which were then refined to a resolution of 4.6 Å that displayed density for both the $Pri2_{CTD}$ and the $Pol1_{exo/cat}$ domains (Figures S3G and S3H).

### *Human replisome + Pol α-primase + 60 nucleotide 5′-flap DNA fork*

The data processing pipeline is illustrated by the schematic in Figure S6C. 7,355 39-fraction movies were aligned and dose-weighted (1.22 e-/Å$^2$/fraction, 5 x 5 patches, 150 Å$^2$ B-factor) using RELION's implementation of a MotionCor2-like program.[69] CTF parameters were estimated using CTFFIND-4.1[70] and 44 poor-quality micrographs excluded from future processing. Particles were picked using RELION's Laplacian-of-Gaussian (LoG) function providing a minimum diameter of 180 Å and maximum of 330 Å. 1,535,548 particles were extracted using a box size of 380 Å. During extraction the data were down-sampled to a pixel size of 3.78 Å/pixel. Two successive rounds of RELION 3D classification (regularisation parameter, T = 4), using 6 classes, were carried out using a previously obtained map of the human core replisome as a 3D reference (EMD-13375).[41] Class selection was based upon the presence of secondary structure features within CMG. 3D classification was performed with a 250 Å diameter circular mask to focus classification on CMG. A resulting 550,340 particles were subsequently refined using 3D auto-refinement in RELION generating a reconstruction at 7.6 Å resolution following postprocessing. These data were submitted for per-particle motion correction using dataset-trained particle polishing in RELION[72] using a pixel size 0.86 Å in a 450 Å box. RELION CTF-refinement[64] (beamtilt and trefoil correction, anisotropic magnification correction, and per-particle defocus and astigmatism CTF correction) was then carried out and the data refined to an improved resolution of 3.6 Å following postprocessing in RELION. A further round of 3D classification was carried out with a dilated circular mask of 380 Å and classes with significant Pol α-primase density representing 359,677 particles selected for subclassification. Signal subtraction and masked 3D classification without alignment was carried out in RELION as described in the budding yeast data processing methods to enrich for replisomes stably associated with Pol α-primase using a mask covering both Pol α-primase and the MCM3 N-terminal helical domain.

3D classes in which Pol α-primase adopted the previously reported autoinhibited primosome conformation (PDB:5EXR)[46] were selected, representing 74,940 particles. Following reversion to the original non-subtracted particles.star file these data were imported into cryoSPARC and refined using non-uniform refinement[73] to 3.6 Å resolution (Figure S7G). Density for DNA was not observed within this reconstruction.

Returning to the previous masked 3D classification without alignment in RELION, classes comprising 174,696 particles were selected in which Pol α-primase adopted a conformation distinct from that of the primosome. Following reversion to the original non-subtracted particles.star file these data were refined via 3D auto-refinement in RELION and postprocessed to a resolution of 4.1 Å. These data were then submitted to an additional round of particle polishing and CTF-refinement in RELION using the same parameters as the previous round. Particles were imported into cryoSPARC and a consensus refinement carried out using non-uniform refinement generating a reconstruction at a resolution of 3.4 Å (Figure S6E). Local refinements were carried out in cryoSPARC, as described in the budding yeast data processing methods, for regions encompassing both TIMELESS-TIPIN/DNA (Figure S6F) and the MCM2-7 C-tier (Figure S6H) resulting in reconstructions at 4.1 Å and 3.7 Å respectively.

In order to improve the quality of the AND-1 density, particle subtraction followed by masked 3D classification without alignment was carried out as described in the budding yeast data processing methods. 3D classification was carried out on the total dataset imported into cryoSPARC, in a conformation distinct from the primosome, focussing on the AND-1/CDC45/GINS region of the map. 3D classes were selected, consisting of 63,393 particles, based on the presence of continuous strong AND-1 density. These data were then locally refined to generate a reconstruction at 3.3 Å resolution (Figure S6G).

In order to improve the quality of the Pol α-primase density, particle subtraction followed by masked 3D classification without alignment was carried out using a mask covering the POLA1$_{CTD}$/POLA2/PRIM1/PRIM2$_{NTD}$ region of the map. 3D classes were selected, consisting of 148,103 particles which were locally refined to generate a reconstruction at 4.4 Å resolution (Figure S6I). A single 3D class was selected from this procedure, containing 23,758 particles, with particularly strong PRIM1 density. The non-subtracted particles comprising this class were subjected to consensus non-uniform refinement generating a reconstruction at 4.3 Å resolution.

### Human replisome + Pol α-primase + 15 nucleotide 5′-flap DNA fork

The data processing pipeline is illustrated by the schematic in Figure S8D. 6,718 39-fraction movies were aligned and dose-weighted (2.27 e-/Å$^2$/fraction, 5 x 5 patches, 150 Å$^2$ B-factor) using RELION's implementation of a MotionCor2-like program.[69] CTF parameters were estimated using CTFFIND-4.1[70] and 109 poor-quality micrographs excluded from future processing. Particles were picked using Gautomatch v0.56 (https://www2.mrc-lmb.cam.ac.uk/research/locally-developed-software/zhang-software/#gauto) leading to extraction of 724,557 particles using a box size of 380 Å and pixel size of 4.28 Å/pixel (raw pixel size 1.07 Å/pixel). Two successive rounds of RELION 3D classification (regularisation parameter, T = 4), using 6 classes, were carried out using a previously obtained map of the human core replisome as a 3D reference (EMD-13375).[41] Class selection was based upon the presence of secondary structure features within CMG. 3D classification was performed with a 250 Å diameter circular mask to focus classification on CMG. A resulting 584,362 particles were subsequently refined using 3D auto-refinement in RELION generating a reconstruction at 8.8 Å resolution. These data were submitted for per-particle motion correction using dataset-trained particle polishing in RELION[72] using a pixel size 1.02 Å in a 450 Å box. RELION CTF-refinement[64] (beamtilt and trefoil correction, anisotropic magnification correction, and per-particle defocus and astigmatism CTF correction) was then carried out and the data refined to an improved resolution of 3.4 Å following postprocessing in RELION. A further round of 3D classification was carried out with a dilated circular mask of 380 Å. 28,202 particles in 3D classes lacking DNA were imported into cryoSPARC and refined using non-uniform refinement to 3.6 Å resolution. 492,011 particles in 3D classes with significant Pol α-primase and DNA density were selected for further subclassification. Signal subtraction and masked 3D classification without alignment was carried out in RELION as described in the budding

yeast data processing methods to enrich for replisomes stably associated with Pol α-primase using a mask covering both Pol α-primase and the MCM3 N-terminal helical domain. The signal subtraction followed by 3D classification without alignment procedure was iterated resulting in the selection of 258,339 particles in 3D classes with strong Pol α-primase density. These data were imported into cryoSPARC and refined using non-uniform refinement to 3.3 Å resolution.

## Cryo-EM model building

### *Budding yeast replisome + Pol α-primase + 60 nucleotide 5′-flap DNA fork*

To begin model building, structures of the budding yeast core replisome (PDB:6SKL)[30] with the MCM C-tier removed and the MCM C-tier in conformation II (PDB:6SKO),[30] were rigid body docked into the cryo-EM map of the budding yeast replisome fully engaged by Pol α-primase in the absence of Ctf4 at 4.6 Å resolution (binned pixel size of 2.27 Å) using ChimeraX.[74] The atomic model for Ctf4 contained within 6SKO was manually removed at this stage. The structure of the human primosome (PDB:5EXR)[46] was then rigid body docked into the region of the cryo-EM map that remained unassigned, guided by the presence of secondary structure features within density for the $Pri2_{NTD}$. Inspection of the fit-to-density following 5EXR docking revealed a lack of strong cryo-EM density corresponding to both the POLA1 exonuclease and catalytic domains and the $PRIM2_{CTD}$, therefore these were subsequently removed from the model. The quality of the fit-to-density for the remaining human Pol α-primase model was then improved via manual manipulation followed by automated docking for both the PRIM1 and $PRIM2_{NTD}$ domains and the $POLA1_{CTD}$/POLA2 module respectively. The positions of these human Pol α-primase subunits provided a reference to which the AlphaFold[50] models for budding yeast $Pri2_{NTD}$ (residues S44-T299), PRIM1 (residues S12-D402) and the crystal structure the $Pol1_{CTD}$/Pol12 dimer (PDB:3FLO) were aligned prior to being rigid body fit into the density. An AlphaFold multimer[75] model for the Pol12 (residues 203-705) / $Pol1_{CTD}$ (residues 1260-1468) complex was aligned to the Pol12 subunit of 3FLO, rigid-body fit into the cryo-EM density and the most N-terminal residue of Pol1 trimmed to I1271. The Pol1 C-terminus was remodelled based on an AlphaFold-Multimer[75] result indicating complex formation between the Pol1 C-terminus and $Pri2_{NTD}$, in an analogous fashion to the $POLA1_{C\text{-}term}$ in the primosome structure 5EXR.

The quality of the fit for the model into the fully engaged cryo-EM map lacking Ctf4 at 4.6 Å resolution was optimised via an all-atom simulation in ISOLDE,[27] using adaptive distance restraints for the dsDNA model (kappa=100). The resulting model was further refined using Phenix[76] real-space-refine, utilising the input model as a reference to generate restraints with sigma=0.1 and global minimisation with nonbonded_weight=2000 and weight=0.5. Regions of the model that fit poorly to the density were the manually refined in Coot[77] using the local refinement and regularisation tools incorporating stereochemical restraints. The model for the Mcm2-ZnF (residues 338–378) was truncated due to the absence of well resolved density in this region of the map.

At this stage in the modelling process, regions of cryo-EM density that remained unmodelled were identified for further analysis to determine their identity. Density for a small four-helical bundle bound to the Mcm3 $AAA^+$ domain was assigned as the flexibly tethered $Pol12_{NTD}$ (residues M1-I79). An AlphaFold-Multimer prediction for the $Pol12_{NTD}$ interacting with the $AAA^+$ domain of Mcm3 was used to dock the $Pol12_{NTD}$ into the cryo-EM density.

Two regions of disconnected helical density were identified in analogous positions to CLASPIN in the core human replisome (PDB:7PFO).[41] This led us to speculate that these represented regions of the budding yeast homologue of CLASPIN, Mrc1. Furthermore, AlphaFold-Multimer modelling predicted multiple high-confidence interactions between Mrc1 and the replisome. Predictions were validated by the presence of corresponding side-chain density for Mrc1, present in the un-binned cryo-EM reconstruction of the fully engaged Pol α-primase complex at 3.5 Å resolution. Mrc1 residues S339-K323 interact with the α-solenoid of Tof1, whilst D468-Q483 contacts Mcm2 in the N-tier and the Tof1 N-terminus. An additional region of disconnected density, on the same side of the replisome to the two modelled regions of Mrc1, was also assigned to Mrc1 based on AlphaFold-Multimer prediction and the presence of clear side-chain density. Alphafold-Multimer predicts an interaction between Mrc1 residues L815-E858 spanning both Cdc45 and the Mcm2 N-tier, for which there is cryo-EM density present in the fully engaged reconstruction at 4.6 Å resolution. However, there is only cryo-EM density of sufficient resolution to enable unambiguous assignment, in the 3.5 Å resolution fully engaged map, for Mrc1 residues N842-E858, therefore these are the only residues deposited in the final model for this particular Mrc1 interface.

A region of unmodelled density bound to the GINS subunit Psf2 was ascribed to $Pri2_{Nterm}$ (residues M1-S5). Assignment was based upon the close proximity of the otherwise most N-terminal modelled residue of Pri2 (S44) and the presence of continuous density between S5-S44 present at low map thresholds. Clear side chain density was present for residues M1-Q4. As the second residue in Pri2 is a large phenylalanine residue the first methionine will not be removed[78] and is likely to be acetylated.[79]

At this stage the model was inspected residue-by-residue in Coot,[77] docked into the highest resolution cryo-EM map (consensus and focused refinements) for the corresponding region of the model. Focussed refinements were rigid-body docked into the consensus and resampled onto the same origin. The model was manually refined against the map in Coot[77] and both backbone Ramachandran and rotamer outliers corrected. A final global run in ISOLDE[80] was carried out to minimise the clash-score using distance and torsion restraints prior to Phenix[76] real-space-refinement using the same restraints as described above. Model validation was carried out using the Molprobity server,[81] Phenix[76] validation and the wwPDB OneDep validation server.

In order to model the budding yeast replisome fully engaged by Pol α-primase in the presence of Ctf4, the structure of trimeric Ctf4 (PDB:6SKL)[30] was rigid body docked into the corresponding density. The fit-to-density was subsequently refined via local ISOLDE[80] simulation followed by Phenix[76] real-space-refinement and manual adjustment in Coot.[77] Inspection of the locally refined Ctf4/GINS/Cdc45 cryo-EM map revealed the presence of unmodelled density bound to the helical bundles of two Ctf4 monomers in the correct position to accommodate the Pol1 CIP-box (a.a. F140-S149).[29] The Pol1 CIP box was subsequently modelled into each discrete

density based on the crystal structure of the $Ctf4_{CTD}$-Pol1 CIP-box (PDB: 4C93).[29] It was not possible to sub-classify the Ctf4 density to generate reconstructions with only one-site occupied at any time, therefore two models were deposited to the PDB with the Pol1 CIP-box interacting with a different monomer of Ctf4 in each.

***Human replisome + Pol α-primase + 60 nucleotide 5′-flap DNA fork***

A previously determined structure of the core human replisome (PDB: 7PFO)[41] was rigid body docked into the locally filtered cryo-EM map of the human replisome fully engaged by Pol α-primase on a DNA fork containing a 60 nucleotide 5'-flap at 3.4 Å resolution using ChimeraX.[74] The atomic model for Pol ε contained within 7PFO[41] was manually removed at this stage, as were MCM2 residues 324-368 which comprise its zinc-finger motif due to the lack of corresponding density. Using the same strategy employed for the modelling of the budding yeast Pol α-primase-replisome structure, a previously determined model for the human primosome (PDB:5EXR) was rigid body docked into the remaining unassigned density. The 5EXR model was then manually edited to remove both the POLA1 exonuclease and catalytic domains and the $PRIM2_{CTD}$ due to a lack of corresponding cryo-EM density. The fit-to-density for the Pol α-primase subunits was improved by docking each module: PRIM1, PRIM2 and the $POLA1_{CTD}$/POLA2 dimer, individually as a rigid body. AlphaFold multimer[75] structure predications for the PRIM1- $PRIM2_{NTD}$ complex and the POLA2-$POLA1_{CTD}$ complex were aligned to the corresponding subunits derived from 5EXR, replacing the crystal structure subunits. Using this strategy atomic models were generated for the following regions of sequence: PRIM2 residues Q17-H252, PRIM1 residues M9-T349 and T386-G408, POLA1 residues Q1279-G1445 and E1448-C1458 and POLA2 residues I96-T114 and V170-I598.

The quality of the fit-to-density was optimised via an all-atom simulation in ISOLDE,[80] using adaptive distance restraints for the dsDNA model (kappa=100). The resulting model was further refined using Phenix[76] real-space-refine, utilising the input model as a reference to generate restraints with sigma=0.1 and global minimisation with nonbonded_weight=2000 and weight=0.5. Regions of the model that fit poorly to the density were then manually refined in Coot[77] using the local refinement and regularisation tools incorporating stereochemical restraints.

Inspection of the cryo-EM density following the modelling procedure outlined revealed a short region of unmodelled helical density adjacent to the PSF1 subunit. AlphaFold-Multimer analysis predicted a helix within the flexible N-terminus of POLA2, residues I96-T114, to bind at the location of the unmodelled density. Furthermore, clear side chain density for POLA2 Y113 and L109 corroborated the prediction in addition to stereo-chemically favourable contacts formed. This region of POLA2 was subsequently incorporated into the final model.

A region of unmodelled density bound to the GINS subunit PSF2 was assigned to the $PRIM2_{Nterm}$, residues M1-G5, and incorporated into the final model. Assignment was based upon the close proximity of the otherwise most N-terminal modelled residue of Pri2, Q17, and the presence of continuous density between G5-Q17 present at low map thresholds. Clear side chain density was present for residues M1-S4. Furthermore, the $PRIM2_{Nterm}$ – PSF2 contact was predicted by AlphaFold-Multimer at high confidence.

At this stage the model was inspected residue-by-residue in Coot,[77] docked into the highest resolution cryo-EM map (consensus and focused refinements) for the corresponding region of the model. The model was manually refined against the map in Coot[77] and both backbone Ramachandran and rotamer outliers corrected, followed by real-space refinement in both ISOLDE[80] and Phenix.[76] Each focussed refinement was rigid-body docked into the consensus refinement (EMD-15341) and resampled onto the same map origin. Only density at the AND-1 – CDC45/GINS interface was used to align the CDC45/GINS/AND-1 local refinement (EMD-15342) to the consensus (EMD-15341). However, due to subtle differences between the density at this interface between the local and consensus refinements it was not possible to build a model that perfectly satisfied both maps. A final global run in ISOLDE[80] including distance and torsion restraints was carried out to minimise the clash-score prior to Phenix[76] real-space-refinement using the same restraints as described above into the consensus refinement with additional reference model restraints. Model validation was carried out using the Molprobity server,[81] Phenix[76] validation and the wwPDB OneDep validation server. Following this procedure, the model for CLASPIN residues E299-E310 was removed due to the lack of corresponding cryo-EM density.

## Multiple sequence alignments

Amino acid sequences were retrieved from UniProt and protein sequence alignments carried out using Clustal Omega.[82] Alignments were rendered using ESPript3.0 (http://espript.ibcp.fr).[83]

## AlphaFold and AlphaFold-Multimer

AlphaFold models were obtained from the AlphaFold Protein Structure Database[50] (https://alphafold.ebi.ac.uk). AlphaFold-Multimer structure predictions were determined using the Colabfold[84] implementation (version 1.2.0) using the following parameters: num_models=5, num_recycles=3, use_amber=false, use_templates=false. Colabfold sequence alignments were performed using Mmseqs2. Below is a description of the AlphaFold-Multimer input sequences, including protein names and the residue ranges included as input: $Pri2_{NTD}$ (44-299) / $Pol1_{C\text{-}term}$ (1140-1468); $Pol12_{N\text{-}term}$ (1-74) / Mcm3 $AAA^{+}$ domain (337-750); Pol12 (203-705) / $Pol1_{CTD}$ (1260-1468); $Mrc1_{site\ \#1}$ (300-350) / Tof1 (1-779); $Mrc1_{site\ \#2}$ (420-550) / Tof1 (1-300) / Mcm2 (150-458); $Mrc1_{site\ \#3}$ (600-1000) / Mcm2 (150-458) / Cdc45 (1-468); $Pri2_{Nterm}$ (1-30) / Psf2 (1-213); $PRIM2_{Nterm}$ (1-14) / PSF2 (1-185); POLA2 (1-200) / PSF1 (1-196) / SLD5 (1-223); POLA2 (100-598) / $POLA1_{CTD}$ (1260-1458); PRIM1 (1-420) / Pri2 (1-509).

### Budding yeast primase-polymerase assay on M13mp18 ssDNA

Reactions (10 μl) were performed at 30°C in a buffer containing: 25 mM HEPES-KOH (pH 7.6); 100 mM potassium glutamate; 10 mM $Mg(OAc)_2$; 1 mM DTT; 0.01 % NP-40 substitute (NP-40-S) (Roche #11754599001), 0.1 mg/ml bovine serum albumin (BSA); 0.5 nM M13mp18 ssDNA (NEB #N4040S); 30 μM dC, dG, dT, dATP; 200 μM G, C, UTP; 33 nM α-[$^{32}$P]-dCTP (Hartmann Analytic #SCP-205); 3 mM ATP; 40 nM RPA; 20 nM Pol α-primase. RPA was prebound to the ssDNA template for 10 min and then reactions were started by addition of Pol α-primase. Reactions were quenched with 10 μl 100 mM EDTA and unincorporated radiolabelled nucleotide was removed using a Microspin G-50 column (Cytiva). 1/10 volume loading buffer (10% w/v sucrose; 500 mM NaOH; ~0.25% w/v xylene cyanol) was added to the sample before analysis on 0.7% alkaline agarose gels run in 30 mM NaOH, 2 mM EDTA for 16 hours at 25 volts. After electrophoresis gels were incubated at 4°C in 5% trichloroacetic acid for 30 min with one buffer change after 15 min. The gel was then incubated in 500 mM Tris-Cl (pH 8) for 15 mins before being dried at 75°C under vacuum onto Whatman 3 MM paper (Cytiva). The dried gel was imaged using a BAS-IP MS phosphor screen (Cytiva) and an Amersham Typhoon phosphorimager and on Amersham Hyperfilm MP (Cytiva).

### Origin-dependent budding yeast DNA replication assay

Origin-dependent budding yeast DNA replication reactions were based on a previously established protocol.[1,7,37,38] First, Mcm2-7 double hexamers were loaded onto the DNA template in an MCM loading reaction (10-40 μl) containing: 25 mM HEPES-KOH (pH 7.6); 100 mM potassium glutamate; 10 mM $Mg(OAc)_2$; 1 mM DTT; 0.01 % NP-40-S, 0.1 mg/ml bovine serum albumin (BSA); 40 mM KCl, 3 mM ATP, 3 nM SphI-linearised vVA20[1]; 75 nM Cdt1-Mcm2-7, 40 nM Cdc6; 20 nM ORC; 25 nM DDK. The reaction was incubated at 24°C for 10 mins, at which point S-CDK was added to a final concentration of 80 nM and incubation continued at 24°C for a further 5 min. The MCM loading reaction was diluted 4-fold into a replication buffer to give a final replication reaction buffer consisting of: 25 mM HEPES-KOH (pH 7.6); 250 mM potassium glutamate; 1 mM DTT; 0.01 % NP-40-S, 0.1 mg/ml BSA; 10 mM KCl, 3 mM ATP, 30 μM dC, dG, dT, dATP; 200 μM G, C, UTP, 33 nM α-[32P]-dCTP (Hartmann Analytic #SCP-205); 0.75 nM SphI-linearised vVA20; 18.75 nM Cdt1-Mcm2-7, 10 nM Cdc6; 5 nM ORC; 6.25 nM DDK; 20 nM S-CDK. Pol α-primase was added to a final concentration of 10 nM and reactions (10 μl) were equilibrated at 30°C. DNA replication was initiated by addition of replication proteins to final concentrations of: 30 nM Dpb11; 100 nM GINS; 30 nM Cdc45; 10 nM Mcm10; 20 nM Pol ε; 20 nM Ctf4; 100 nM RPA; 20 nM RFC; 20 nM Tof1-Csm3; 20 nM PCNA; 10 nM Pol δ; 12.5 nM Sld3/7; 20 nM Sld2; 10 nM Mrc1. Reactions were incubated at 30°C for 20 mins before quenching, processing and analysis as described for budding yeast primase-polymerase assays on M13mp18 ssDNA.

### Budding yeast tetrad dissection

Details of yeast strains can be found in Table S4. Diploid yeast cells were patched onto sporulation plates (0.25% yeast extract; 0.1% glucose; 1.5% potassium acetate; 2% agar; 5 μg/ml Arginine, 10 μg/ml Adenine, 10 μg/ml Uracil; 5 μg/ml Histidine; 5 μg/ml Leucine; 5 μg/ml Lysine; 5 μg/ml Tryptophan, 2 μg/ml Tyrosine; 25 μg/ml Phenylalanine; 5 μg/ml Methionine; 1 μg/ml Proline) and incubated for 3-5 days at 30°C. Sporulated cells were picked using sterile toothpicks and resuspended in 75 μl sterile Milli-Q water and 5 μl β-Glucuronidase (Sigma #G7017). Following incubation at room temperature for 15-20 min, 150 μl sterile Milli-Q water was added and 10 μl of this mix was streaked onto YPD plates (1.1% yeast extract; 2.2% peptone; 2% glucose; 55 μg/ml Adenine; 2.5% agar). Tetrads were dissected using a micromanipulator (Singer Instruments) and the resulting cells were grown for 3 days at 25°C. Plates were imaged on an Epson Perfection V850 Pro scanner.

Cells were genotyped by analysing growth on appropriate selective plates or, when two alleles were associated with the same auxotrophic marker, by PCR from purified genomic DNA. For Pri2, primers JY105 and JY609 were used to generate a 608 bp PCR product. Products were digested with BsaHI, which digests the *Pri2-AAA* allele but not the wild type. For Mcm3, primers JY604 and VA212 were used to generate a 728 bp PCR product. Products were digested with FspI, which digests the wild type allele but not *Mcm3-CR*. Alternatively, primers JY370 / JY491 were used to amplify a region of the Mcm3 allele linked to the Ura3 marker. Primers JY370 / JY104 were used to amplify a region of the Pri2 allele linked to the Ura3 marker.

### Human primase-polymerase assay on M13mp18 ssDNA

Reactions were performed at 37°C in a buffer containing 25 mM HEPES-KOH (pH 7.6), 100 mM potassium glutamate, 0.01% NP-40-S, 1 mM DTT, 10 mM $Mg(OAc)_2$, 0.1 mg/ml BSA, 5 mM ATP, 200 μM CTP, GTP, UTP, 30 μM dATP, dCTP, dGTP, dTTP, and 33 nM α-[$^{32}$P]-dCTP. 0.5 nM M13mp18 single-stranded DNA (New England Biolabs) was pre-incubated with 40 nM RPA for 10 min. Reactions were initiated by the addition of 20 nM Pol α-primase. After 20 min, reactions were quenched by addition of EDTA to 50 mM. Post reaction processing and analysis were performed as described for budding yeast primase-polymerase assays on M13mp18 ssDNA.

### Human DNA replication assays

Replication reactions were performed on a 9.7 kbp forked linear DNA template (made from SapI-linearized ZN3 plasmid) as previously described[27] with minor modifications. Reactions were conducted at 37°C in a replication buffer consisting of 25 mM HEPES-KOH (pH 7.6), 0.01% NP-40-S, 100 mM potassium glutamate, 1 mM DTT, 10 mM $Mg(OAc)_2$ and 0.1 mg/ml BSA. The final concentration of potassium glutamate in reactions was increased to 230 mM when replication was initiated. Protein and nucleotide concentrations in the final reactions were: 25 nM CMG, 15 nM Pol ε, 20 nM RFC, 7 nM Pol δ, 20 nM PCNA, 20 nM AND-1, 10 nM Pol

α-primase, 100 nM RPA, 20 nM CLASPIN, 20 nM TIMELESS–TIPIN, 20 nM CTF18–RFC, 4 mM ATP, 30 μM dC/dT/dG/dATP, 200 μM C/G/UTP and 33 nM α-[$^{32}$P]-dCTP. Reactions were set up as follows: 2 nM linear forked DNA template was pre-incubated with 50 nM CMG for 10 min in replication buffer. The reaction was diluted twofold by the addition of replication buffer containing 60 μM dA/dCTP, PCNA, Pol ε, Pol α-primase, Pol δ, CLASPIN, TIMELESS–TIPIN, AND-1 and CTF18–RFC. Replication was initiated by addition of a 10X solution containing ATP, RFC, dTTP, dGTP, GTP, CTP, UTP, α-[$^{32}$P]-dCTP and RPA. Reactions were stopped by addition of 50 mM EDTA and unincorporated α-[$^{32}$P]-dCTP was removed with illustra MicroSpin G-50 columns (GE Healthcare). Reactions were run on 0.7% alkaline agarose gels in 2 mM EDTA and 30 mM NaOH and for 16 h at 26 V. Post reaction processing and analysis were performed as described for budding yeast primase-polymerase assays on M13mp18 ssDNA.

## QUANTIFICATION AND STATISTICAL ANALYSIS

No quantification or statistical analysis were performed in this manuscript.

**Molecular Cell, Volume *83***

## Supplemental information

# How Pol $\alpha$-primase is targeted to replisomes to prime eukaryotic DNA replication

**Morgan L. Jones, Valentina Aria, Yasemin Baris, and Joseph T.P. Yeeles**

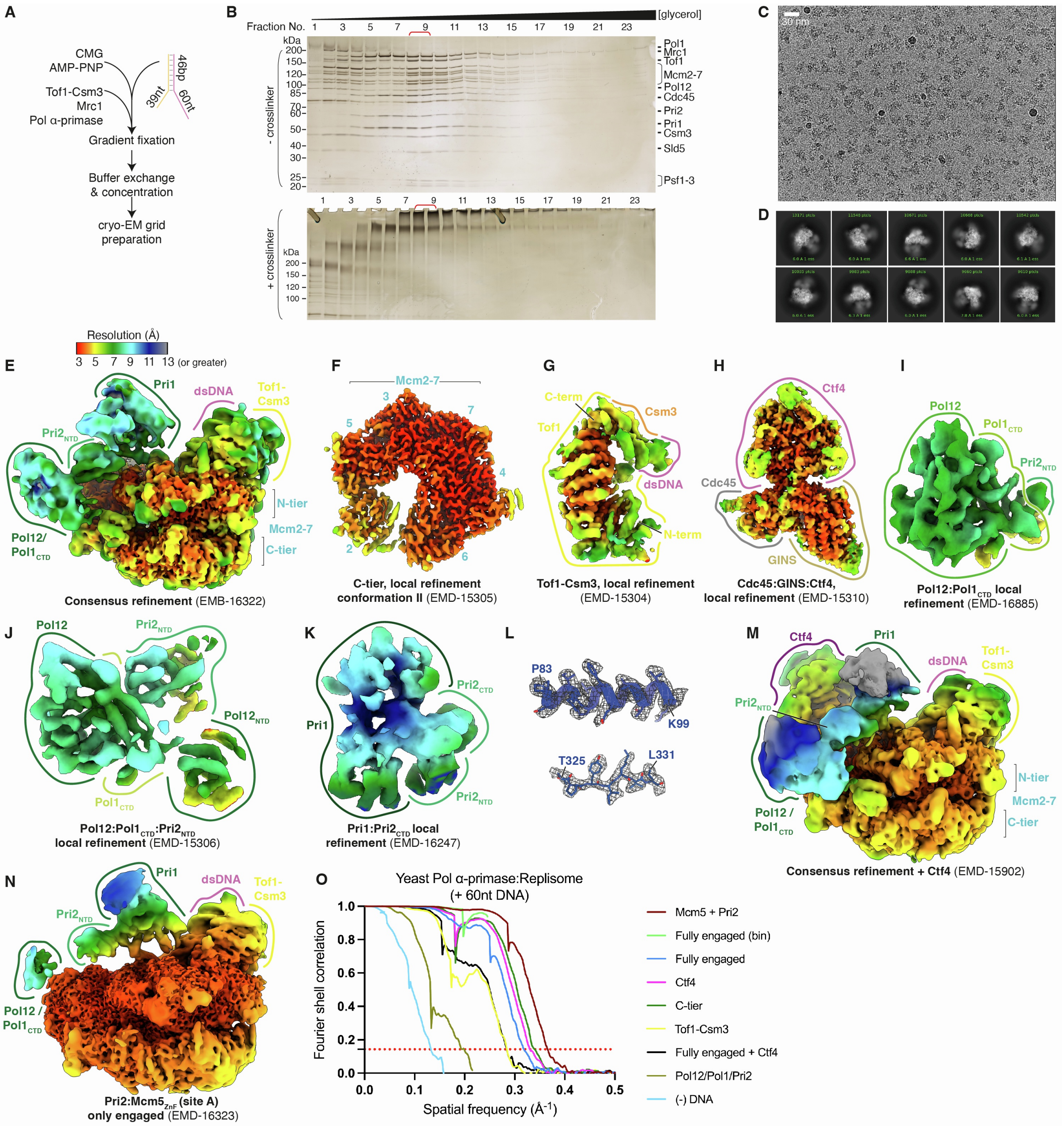


**Figure S1. Cryo-EM analysis of a budding yeast replisome containing Pol α-primase assembled on forked DNA with a 60 nt 5'-flap, related to Figure 1.**

**(A)** Schematic outlining the *in vitro* reconstitution method used to generate complexes for cryo-EM. Cartoon of the forked DNA is shown with the leading-strand template in orange and the lagging-strand template in pink.

**(B)** Silver-stained SDS-PAGE gels analysing 100 µl fractions taken across 10-30% glycerol gradients, either in the absence (top) or presence (bottom) of crosslinking agents. Fractions 8-9 that were used for cryo-EM sample preparation are indicated with red brackets.

**(C)** Representative cryo-EM micrograph, 30 nm scale bar inset, obtained using a K3 direct electron detector (Gatan) at a nominal pixel size of 0.86 Å/pixel.

**(D)** Representative 2D class averages with corresponding particle numbers, mask diameter 360 Å, obtained using cryoSPARC-3 2D classification.

**(E-K)** Cryo-EM density maps for reconstructions relevant to model building in the absence of Ctf4, coloured by local resolution according to inset key in **(E)**. Local resolution was calculated using RELION for the reconstruction in panel **(E)**, and ResMap[S1] for panels **(F-K)**.

**(L)** Representative cryo-EM density (mesh) for an α-helix (top) at a local resolution of 3.1 Å and a β-strand at a local resolution of 2.9 Å (bottom) in Mcm5 (blue ribbon model).

**(M)** Cryo-EM reconstruction, obtained via consensus refinement, for particles containing Ctf4, coloured by local resolution, calculated using RELION, according to inset key in panel **(E)**.

**(N)** Cryo-EM reconstruction obtained via consensus refinement in which only the $Pri2_{NTD}$:Mcm5 (site a) and $Pri2_{Nterm}$:Psf2 (site d) interfaces are simultaneously occupied, coloured by local resolution, calculated using RELION, according to inset key in **(E).**

**(O)** Fourier shell correlation (FSC) graph describing the maps used in model building. Resolution was calculated using the FSC=0.143 cut-off with values reported in Figure S2.

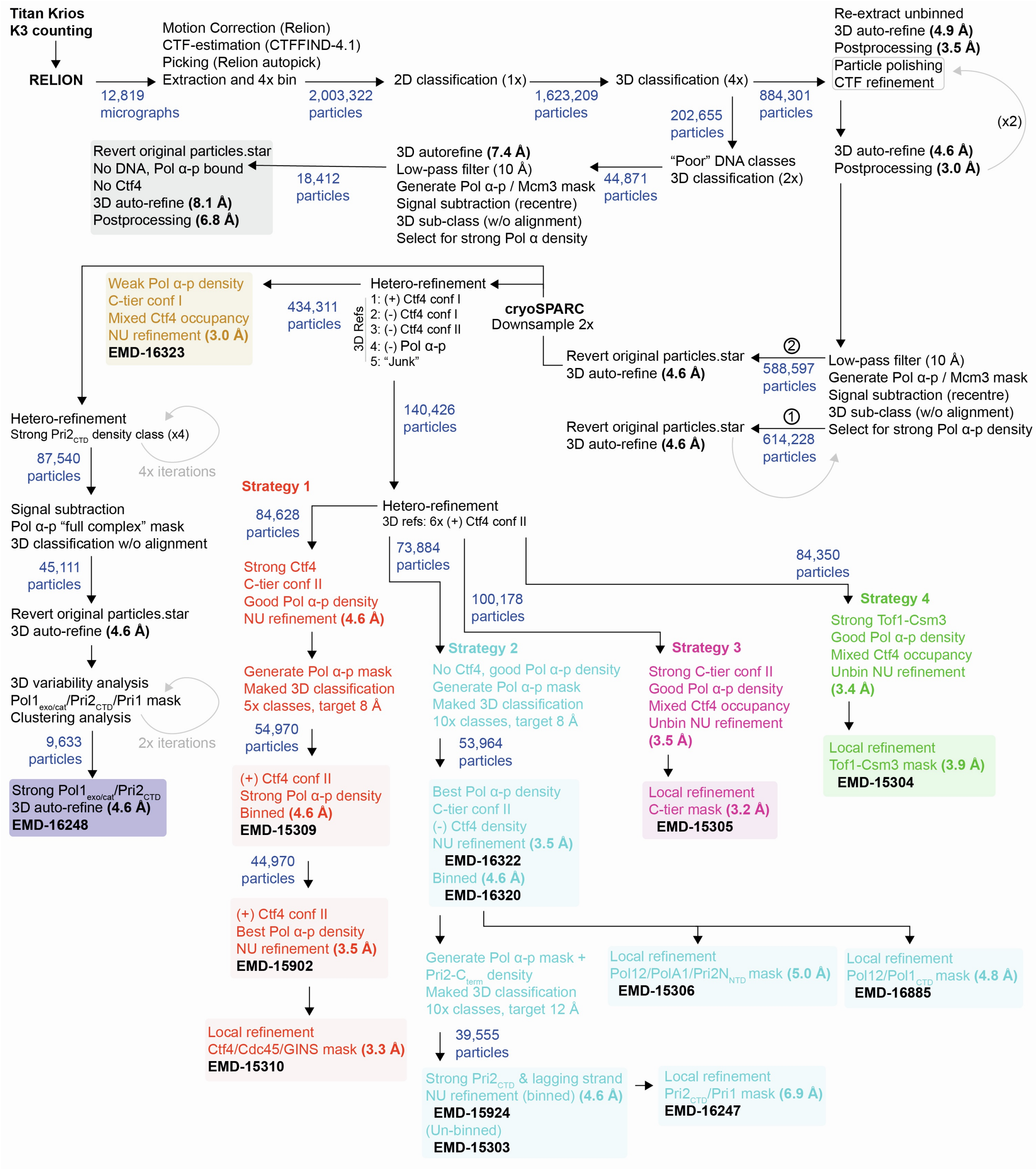


**Figure S2. Cryo-EM data processing pipeline for the budding yeast replisome assembled on forked DNA with a 60 nt 5'-flap, related to Figure 1.**

Abbreviations: NU, non-uniform; w/o, without; Pol α-p, Pol α-primase. Shaded text denotes cryo-EM maps deposited in the EMDB.

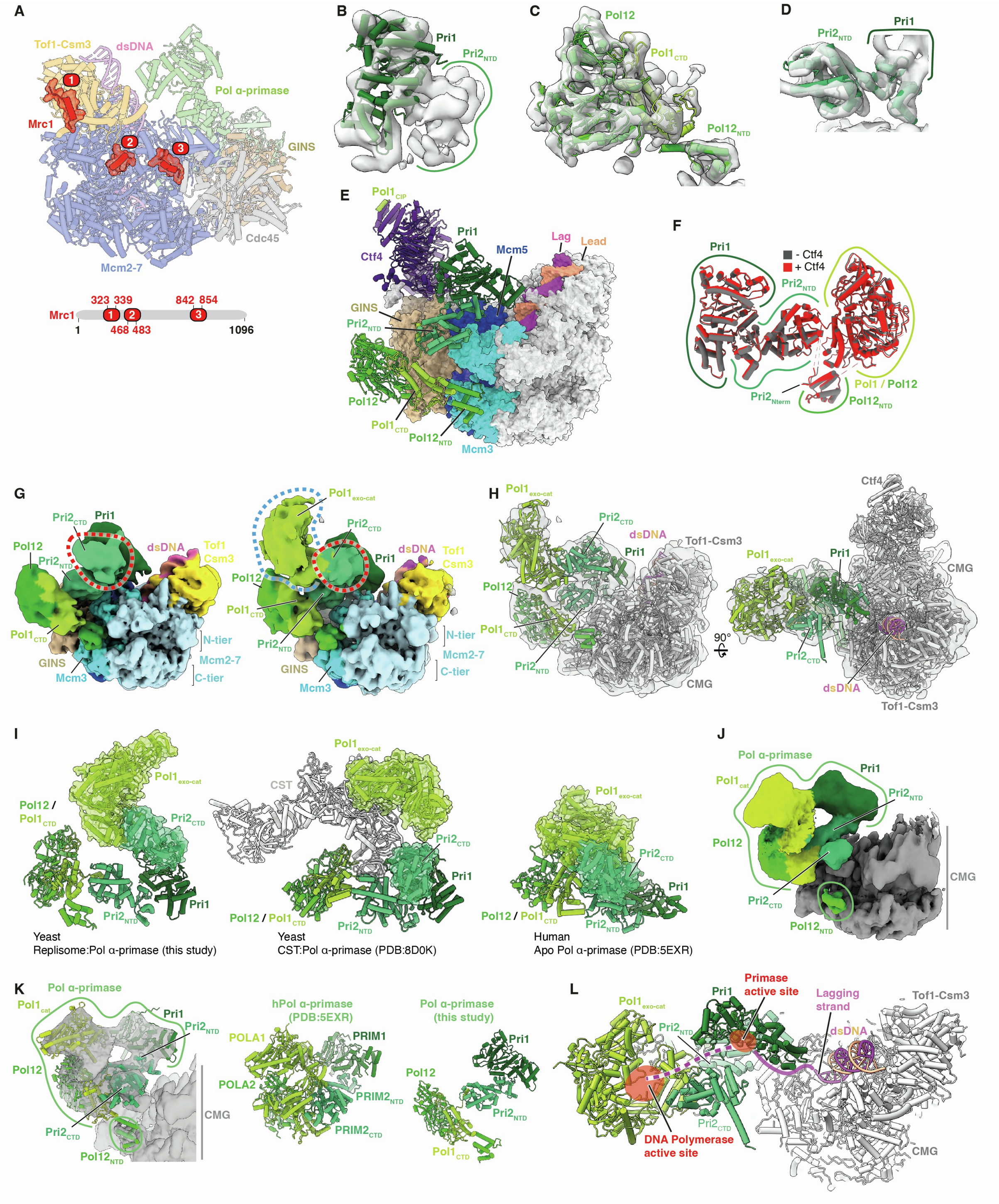

**Figure S3. Structure of a budding yeast replisome containing Pol α-primase, related to Figure 1.**

**(A)** Model highlighting the regions of Mrc1 (coloured red with surface rendering) for which an atomic model could be built. Modelled regions are numbered 1-3 (indicated by red circles) and their associated sequence coverage denoted by the sequence diagram.

**(B-D)** Atomic models displayed with cryo-EM density for Pri1 (**B**), Pol12-Pol1$_{CTD}$-Pol12$_{NTD}$ (**C**) and Pri2$_{NTD}$ (**D**). Cryo-EM density corresponds to local refinements shown in Figures S1I-S1K.

**(E)** Atomic model of the budding yeast Pol α-primase associated replisome containing Ctf4. Regions of CMG that physically interact with Pol α-primase are coloured.

**(F)** Comparison between the structures of Pol α-primase in replisomes lacking (grey) or containing (red) Ctf4, aligned on $Pri2_{NTD}$.

**(G)** Cryo-EM reconstructions, obtained using a binned pixel size of 2.3 Å, containing additional density of the appropriate shape and volume to accommodate $Pri2_{CTD}$ and $Pol1_{exo\text{-}cat}$. (Left) Reconstruction containing density for $Pri2_{CTD}$ (circled red). (Right) Reconstruction containing density for both $Pri2_{CTD}$ (circled red) and $Pol1_{exo\text{-}cat}$ (circled blue).

**(H)** Model for the budding yeast replisome containing Pol α-primase and including $Pri2_{CTD}$ and $Pol1_{exo\text{-}cat}$, fit into the cryo-EM reconstruction in panel **(G)** (right). To fit $Pri2_{CTD}$, the primer DNA strand was removed from the crystal structure of human $PRIM2_{CTD}$ containing a primer/template junction (PDB: 5F0Q)[S2] before being rigid body fit into the $Pri2_{CTD}$ density. The model was initially placed in such an orientation as to maintain the correct lagging-strand template DNA polarity assuming the shortest path after emergence from the Pri1 active site. The AlphaFold[S3] model for yeast $Pri2_{CTD}$ was then aligned to 5F0Q. A crystal structure of the $Pol1_{exo\text{-}cat}$ domain bound to a DNA/RNA duplex and dGTP (PDB: 4FYD)[S4] was placed into the $Pol1_{exo\text{-}cat}$ density such that lagging-strand template DNA was oriented as to be continuous with the previously docked 5F0Q model. The fit-to-density was then optimised via rigid body docking in ChimeraX. DNA was removed from the model for visualisation.

**(I)** Comparison between the conformations of yeast Pol α-primase from this study (left), human Pol α-primase bound to CST and telomeric DNA (PDB: 8D0K)[S5] (middle) and human apo Pol α-primase (PDB: 5EXR)[S2] (right). Models were aligned on their Pri2/$PRIM2_{NTD}$. The Pri2/$PRIM2_{CTD}$ and Pol1/$POLA1_{exo\text{-}cat}$ domains are visualised using transparent surface rendering to highlight their relative positioning between the three models.

**(J)** Cryo-EM reconstruction of a budding yeast replisome containing Pol α-primase but lacking DNA. Regions of the map corresponding to Pol α-primase are coloured according to subunit occupancy.

**(K)** (Left) Atomic model for replisome associated Pol α-primase in the absence of DNA with the corresponding cryo-EM density from panel **(J)** overlaid. (Middle) Atomic model for the human apo Pol α-primase complex (PDB: 5EXR)[S2]. (Right) Atomic model for replisome associated yeast Pol α-primase complex engaged on DNA from this study. Models were aligned on Pri2/$PRIM2_{NTD}$.

**(L)** Model highlighting the arrangement of the primase and DNA polymerase catalytic centres, derived from the cryo-EM reconstruction containing density for both $Pri2_{CTD}$ and $Pol1_{exo\text{-}cat}$. The approximate location of the Pri1 and Pol1 active sites are indicated by red circles. The continuous purple line extending from the last modelled residue of the lagging-strand template is illustrative of the path of DNA described in Figure 1F. The dashed purple line indicates the proposed path of the lagging-strand template extending from the Pri1 active site towards the Pol1 active site.

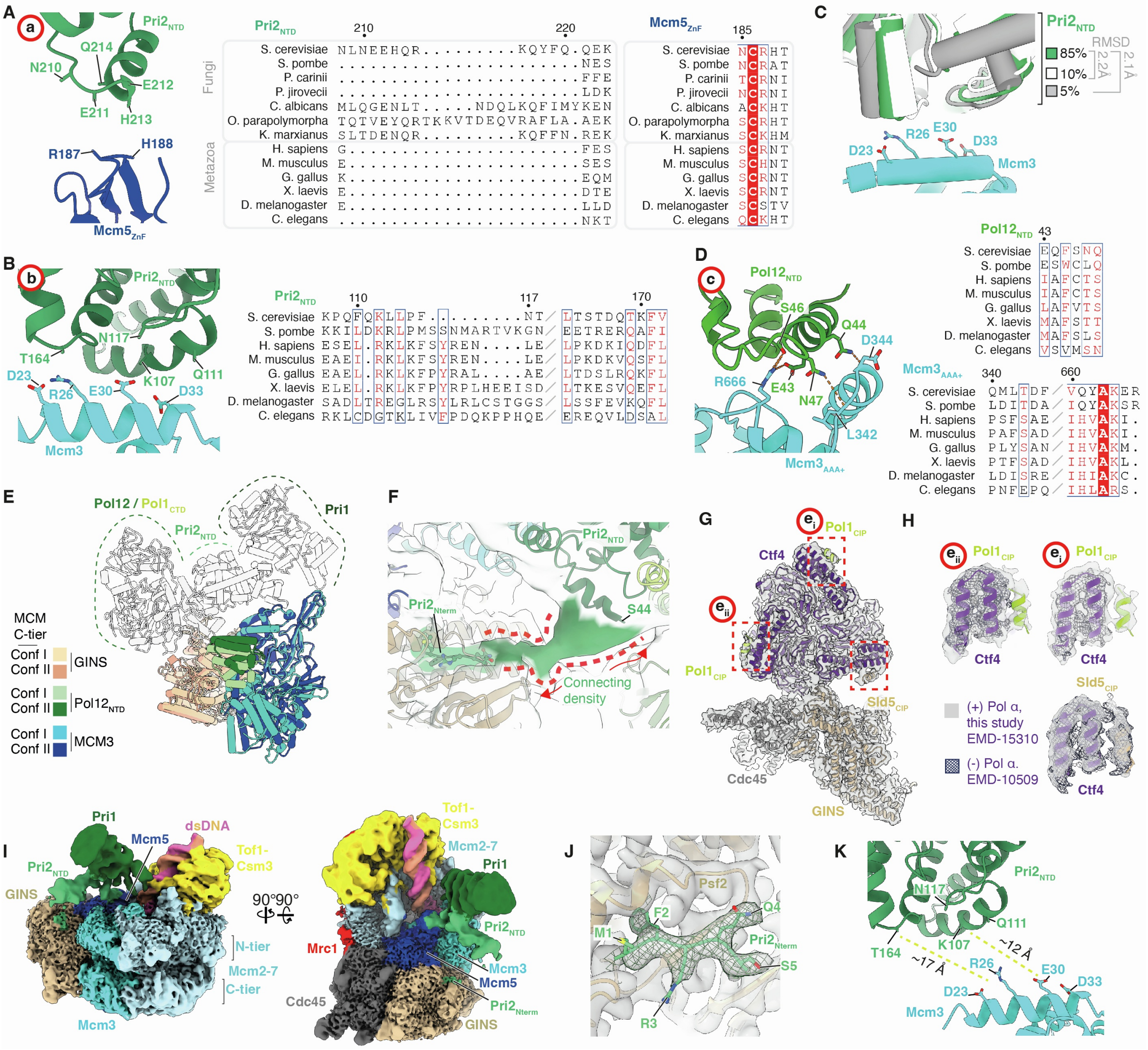


**Figure S4. Details of budding yeast Pol α-primase replisome interactions, related to Figure 2.**

**(A)** (Left) Focused view of the atomic model for the interface between $Pri2_{NTD}$ (green) and the Mcm5 ZnF domain (blue) (site a). Residues positioned to form inter-protein contacts are labelled. Residue sidechains are displayed as truncated stubs as the corresponding cryo-EM density is of insufficient resolution to determine their conformation. (Right) Multiple sequence alignments for the regions of Pri2/PRIM2 and Mcm5 involved in this interface, grouped by Fungal and Metazoan species and coloured according to conservation.

**(B)** Focused view of the atomic model for the electrostatic interface between $Pri2_{NTD}$ (green) and Mcm3 helix α1 of the N-terminal helical domain (cyan) (site b). Residue sidechains are only displayed when the corresponding cryo-EM density is of sufficiently high-resolution to determine their conformation, otherwise sidechains are displayed as truncated stubs. (Right) Multiple sequence alignment for the region of Pri2 involved in this interface coloured according to conservation. The corresponding sequence alignment for the Mcm3 residues involved in this interface are shown in Figure 2C.

**(C)** Atomic model highlighting the flexible nature of the interface between the Mcm3 N-terminal helical domain and $Pri2_{NTD}$ (site b). 3D variability analysis was carried out in cryoSPARC to understand the extent of the positional variance at this interface. Two reconstructions displaying the greatest divergence in $Pri2_{NTD}$ positioning with

respect to the Mcm3 were obtained. Models for both Mcm3 and Pri2 were rigid-body fit into each reconstruction, then each model aligned to the Mcm3 N-terminal helical domain. Visualised here is the overlay of the two resulting models (grey and white) alongside the dominant conformation deposited in the PDB (green). Included are the percentages of particles occupying each discrete class and their relative RMSD values.

**(D)** (Left) Focused view of the atomic model of the interface between $Pol12_{NTD}$ (green) and the Mcm3 AAA+ domain (cyan) (site c). Selected sidechains positioned to form inter-protein contacts are displayed, with predicted hydrogen bonds indicated by dashed orange lines. (Right) Multiple sequence alignments for the regions of $Pol12_{NTD}$ (top) and Mcm3 (bottom) involved in this interface, coloured according to conservation.

**(E)** Model illustrating the position of $Pol12_{NTD}$ bound to Mcm3 in the C-tier in conformations I and II[S6]. To generate the model for this interface in MCM C-tier conformation I, an existing model for Mcm3 and GINS in conformation I (PDB: 6SKL)[S6] was aligned to the N-tier of Mcm3 from this study (conformation II). The structure of the $Pol12_{NTD}$ in complex with the Mcm3 AAA+ domain from this study was then aligned to the Mcm3 AAA+ domain in conformation I from 6SKL. The interface can be maintained in both C-tier conformations without clashes.

**(F)** Model for the Pol α-primase associated replisome docked into a cryo-EM map (transparent grey surface) visualised using a low threshold. Continuous density is visible extending from the most C-terminal residue of $Pri2_{Nterm}$ visualised (S5) to the next visualised residue (S44). This connecting density was manually coloured green using ChimeraX.

**(G)** Atomic model for Cdc45, GINS and Ctf4 in complex with the Sld5 CIP-box and two copies of the Pol1 CIP-box docked into a locally refined cryo-EM density map (transparent grey surface).

**(H)** Focused view of the cryo-EM density at the CIP-box interaction sites on the helical bundle of each Ctf4 monomer. The crystal structures of the Pol1 CIP-box in complex with Ctf4 (PDB:4C93)[S7] and the Sld5 CIP box (PDB:4C95)[S8] were rigid-body docked into the cryo-EM density. Models were overlaid with cryo-EM density both from this study, and a yeast replisome reconstruction in the absence of Pol α-primase (EMD-10509)[S6].

**(I)** Alternative views of a cryo-EM reconstruction of a Pol α-primase associated replisome engaged only via the $Pri2_{NTD}$:$Mcm5_{ZnF}$ (site a) and $Pri2_{Nterm}$:Psf2 (site d) interfaces. The density is coloured according to chain occupancy as indicated.

**(J)** Model for the $Pri2_{Nterm}$:Psf2 interface (site d) built into the cryo-EM map described in **(I).** Cryo-EM density (transparent grey) displayed with the $Pri2_{Nterm}$ density highlighted using green mesh.

**(K)** Model focused on the regions of $Pri2_{NTD}$ and Mcm3 N-terminal helical domain (site b) involved in complex formation built into cryo-EM density for the map described in **I**. Residues seen to be interacting in site b are now too far away to make contacts. Distances between sidechains are indicated using dashed yellow lines.

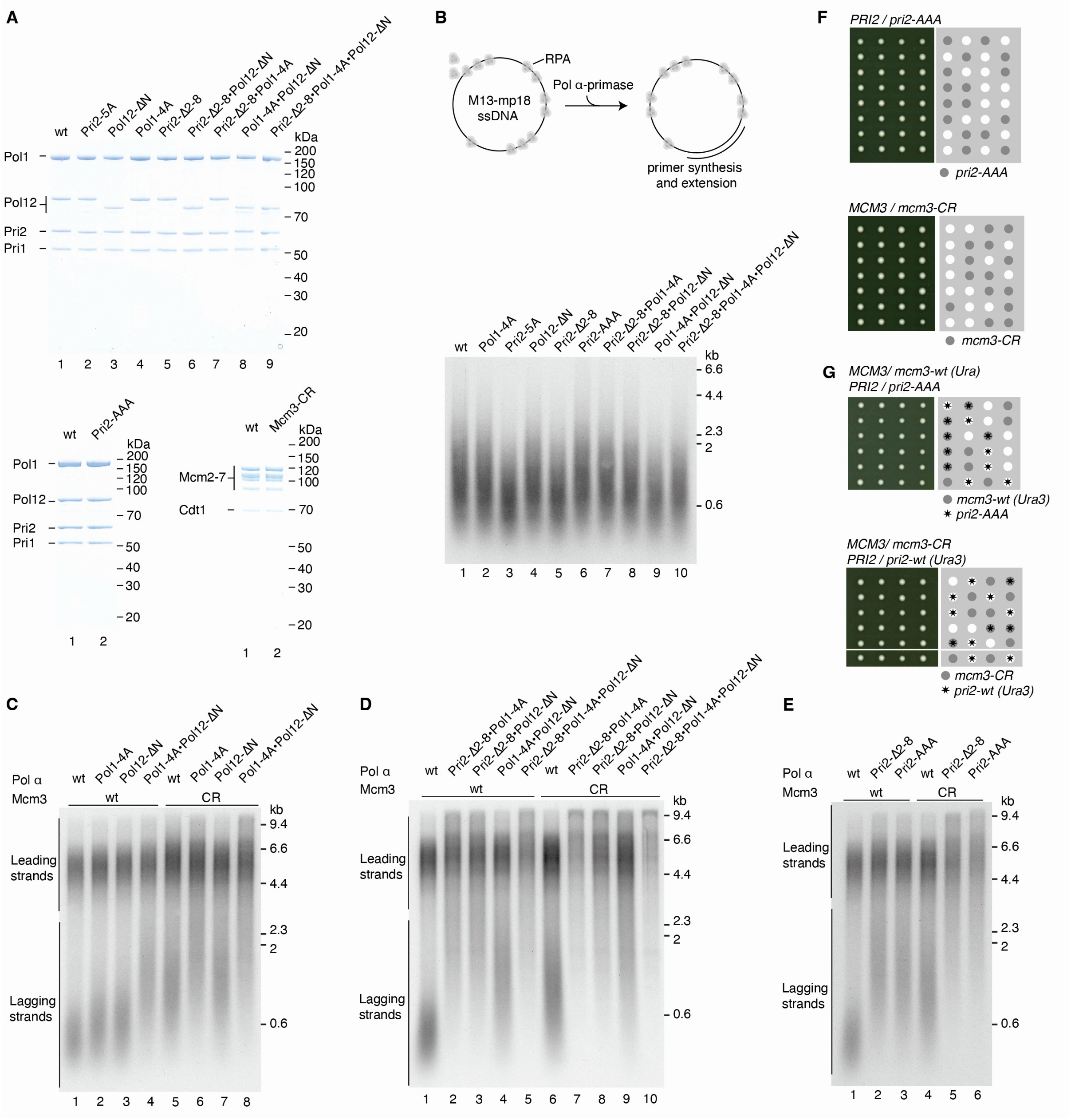


**Figure S5. Analysis of budding yeast Pol α-primase interaction site mutants *in vitro* and *in vivo*, related to Figure 3.**

**(A)** Coomassie-stained 4-12% SDS-PAGE analysis of purified Pol α-primase and Cdt1-Mcm2-7 mutant/truncated complexes. Pri2-5A, $Pri2^{N210A,\ E211A,\ E212A,\ H213A,\ Q214A}$; Pri2-AAA, $Pri2^{F2A,\ R3A,\ Q4A}$; Pol1-4A, $Pol1^{D141A,\ D142A,\ L144A,\ F147A}$; Pol12-ΔN, $Pol12^{\Delta 2\text{-}81}$, Mcm3-CR, $Mcm3^{D23R,\ R26E,\ E30R,\ D33R}$.

**(B)** Top, Schematic of the primase-polymerase assay on M13mp18 ssDNA in the presence of sub-saturating RPA. Bottom, alkaline agarose gel analysis of an assay performed as illustrated (top) with the indicated proteins for 20 min. **(C-E)** Denaturing agarose gel analysis of origin-dependent DNA replication reactions performed as illustrated in Figure 3B with the indicated proteins for 20 min.

**(F and G)** Diploid budding yeast cells of the indicated genotype were sporulated and the resulting tetrads were dissected and grown on YPD medium for 3 days at 25°C. Dissections that displayed abnormal segregation patterns were cropped from plate images.

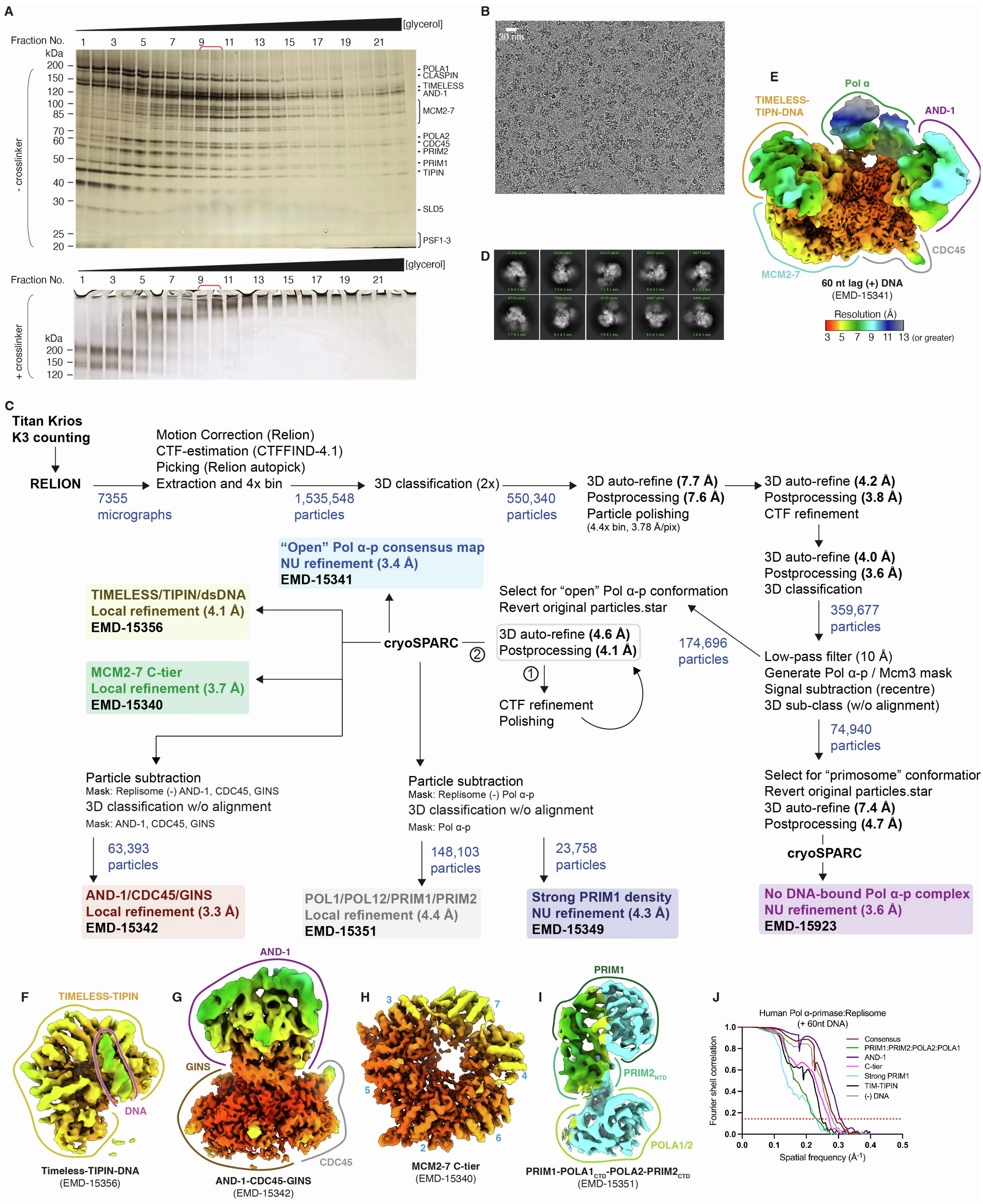


**Figure S6. Cryo-EM analysis of a human replisome containing Pol α-primase assembled on fork DNA with 60 nt 5′-flap, related to Figure 4.**

**(A)** Silver-stained SDS-PAGE gels of 100 µl fractions taken across 10-30% glycerol gradients, either in the absence (top) or presence (bottom) of crosslinking agents. Fractions 9-10 were used for cryo-EM sample preparation (red brackets). **(B)** Representative cryo-EM micrograph obtained using a K3 direct electron detector (Gatan) at a nominal pixel size of 1.23 Å/pixel. 30 nm scale bar inset.

**(C)** Cryo-EM data processing pipeline for the human replisome assembled on forked DNA with a 60 nt 5′-flap. "open" Pol α-primase conformation refers to the DNA engaged state of the complex that differs from the Pol α-primase apo structure (PDB:5EXR). Abbreviations: NU, non-uniform; w/o, without; Pol α-p, Pol α-primase. Shaded text denotes cryo-EM maps deposited in the EMDB.

**(D)** Representative 2D class averages using a mask boundary of 360 Å with corresponding particle numbers labelled, obtained using cryoSPARC-3.

**(E-I)** Cryo-EM reconstructions used in model building coloured by local resolution according to inset key in **(E)**. Local resolution was calculated using RELION for the reconstruction in panel **(E)**, and ResMap[S1] for panels **(F-I)**.

**(J)** Fourier shell correlation (FSC) graph describing the resolution of cryo-EM reconstructions used in model building. Resolution was calculated using the FSC=0.143 cut-off with values reported in **(C)**.

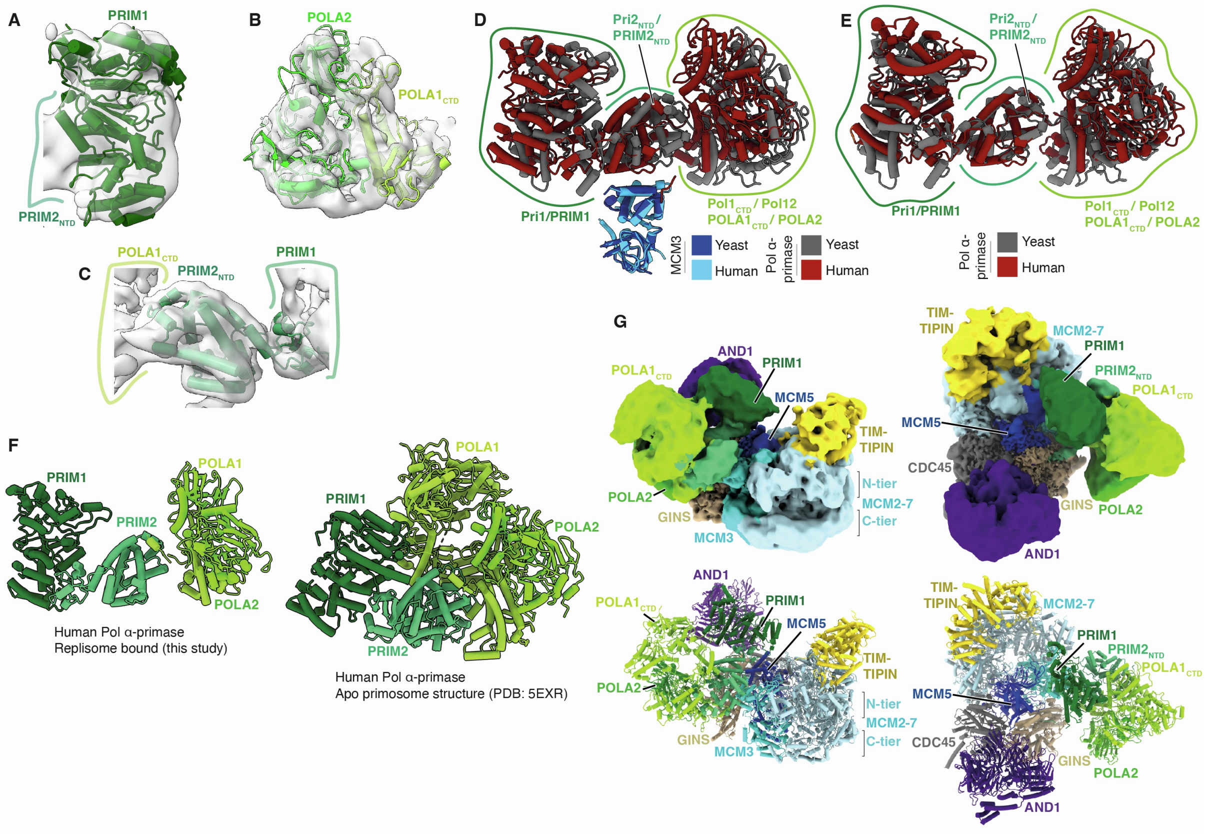


**Figure S7. Structural analysis of Pol α-primase in the human replisome, related to Figure 4.**

**(A-C)** Atomic models displayed with cryo-EM density for PRIM1 (**A**), POLA2 (**B**) and $PRIM2_{NTD}$ (**C**). Cryo-EM density corresponds to the local refinement shown in Figure S6I.

**(D)** Comparison between the atomic models of yeast (grey) and human (red) replisome associated Pol α-primase aligned on the Mcm3 N-terminal helical domain (blue).

**(E)** Comparison between the atomic models of yeast (grey) and human (red) replisome associated Pol α-primase aligned on the $Pri2_{NTD}$.

**(F)** Comparison between the structures of human Pol α-primase associated with DNA-engaged replisomes from this study (left) and the crystal structure of human apo Pol α-primase (right) (PDB: 5EXR)[S2]. Models were aligned on the $Pri2_{NTD}$.

**(G)** Alternative views of a cryo-EM reconstruction (top) and the corresponding model (bottom) for the human Pol α-primase associated replisome not bound to fork DNA. Maps were coloured according to domain occupancy.

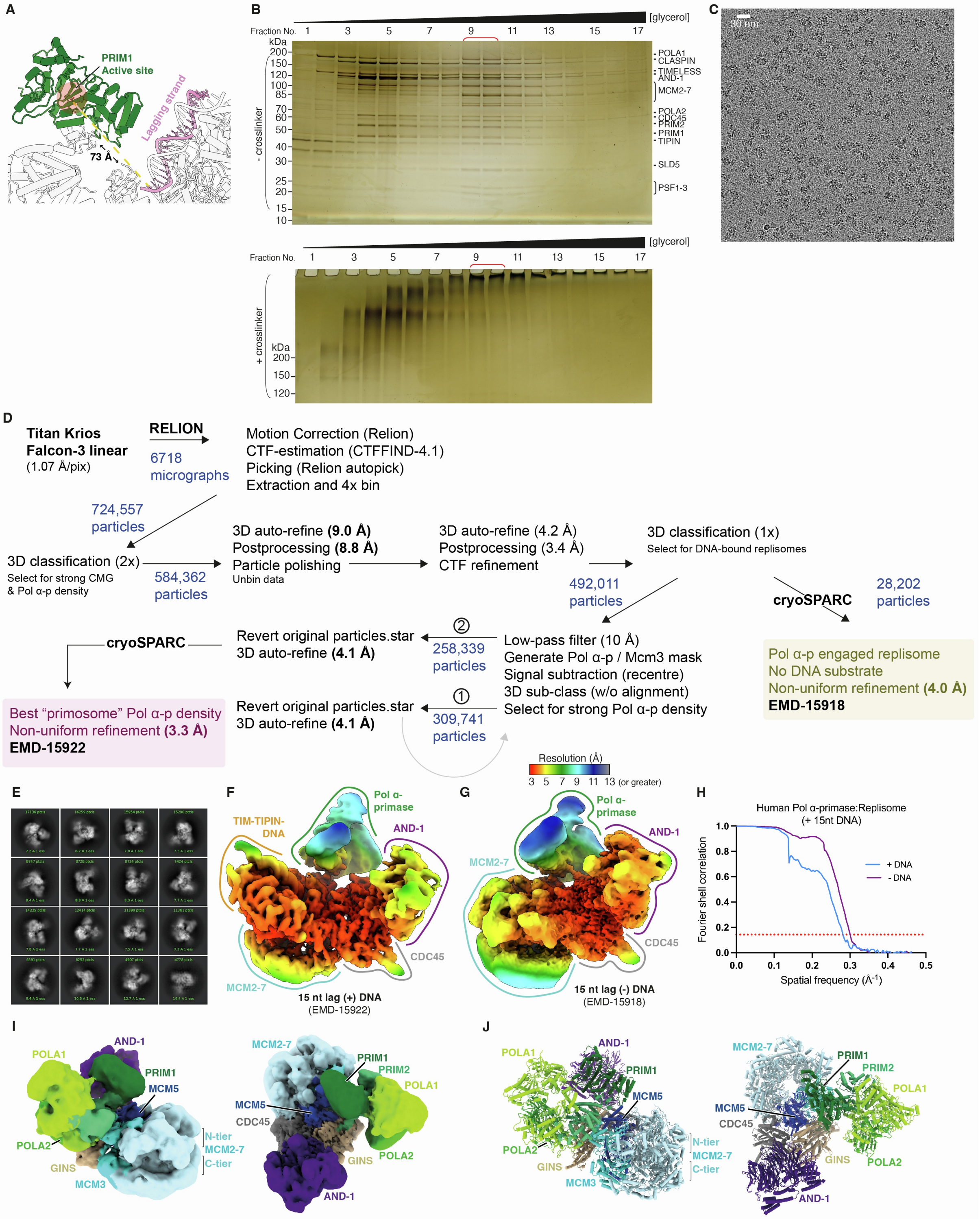


**Figure S8. Cryo-EM structure and analysis of a human Pol α-primase associated replisome assembled on fork DNA with a 15 nt 5′-flap, related to Figure 5.**

**(A)** Structural model indicating the shortest path and distance between the last modelled nucleotide of the lagging-strand template and the active site of PRIM1 (circled red), indicated using a dashed yellow line.

**(B)** Silver-stained SDS-PAGE gels of 100 µl fractions taken across 10-30% glycerol gradients in the absence (top) or presence (bottom) of crosslinking agents. Fractions 9-10 were used for cryo-EM sample preparation (red brackets).

**(C)** Representative cryo-EM micrograph obtained using a Falcon III direct electron detector (Thermo) at a nominal pixel size of 1.07 Å/pixel. 30 nm scale bar inset.

**(D)** Cryo-EM data processing pipeline for the human replisome assembled on forked DNA with a 15 nt 5′-flap. Abbreviations: w/o, without; Pol α-p, Pol α-primase. Shaded text denotes cryo-EM maps deposited in the EMDB.

**(E)** Representative 2D class averages using a mask boundary of 360 Å with corresponding particle numbers indicated obtained using cryoSPARC-3.

**(F and G)** Cryo-EM reconstructions obtained via consensus refinement for complexes engaged with forked DNA containing a 15 nt 5′-flap (left) and lacking DNA engagement (right). Maps coloured by local resolution, calculated using RELION, according to inset key in **(G)**.

**(H)** Fourier shell correlation (FSC) graph indicating the resolution for the cryo-EM reconstructions described in panels **(G)** and **(F)**. Resolution was calculated using the FSC=0.143 cut-off with values reported in (**D**).

**(I and J)** Alternate views of a cryo-EM reconstruction (**I**) and corresponding atomic model (**J**) for the Pol α-primase associated human replisome lacking DNA engagement obtained from the cryo-EM data collection of a human replisome assembled on a DNA fork with a 15 nt 5′-flap. Maps coloured according to subunit occupancy.

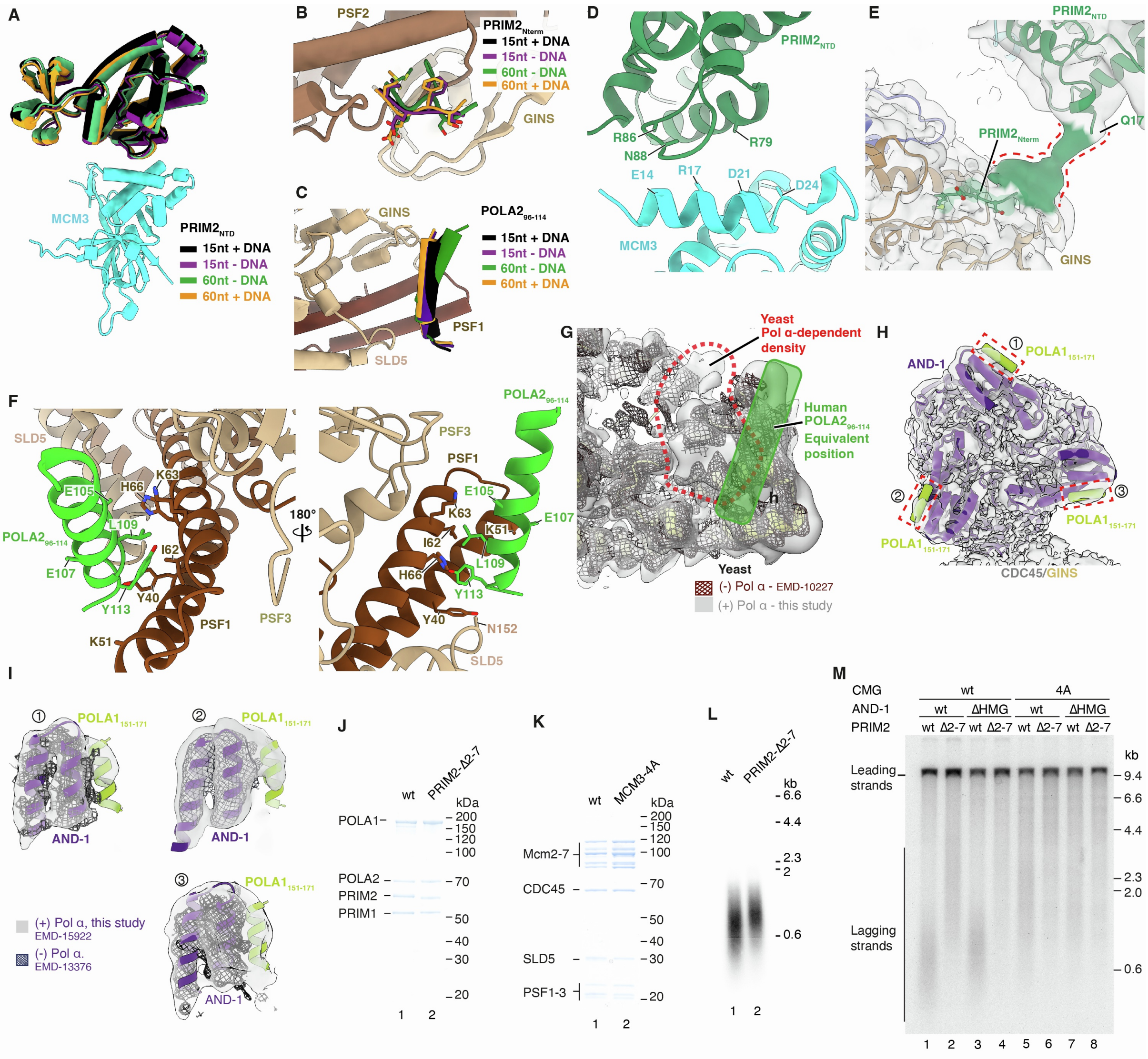


**Figure S9. Analysis of Pol α-primase binding sites in the human replisome, Related to Figure 6.**

**(A)** Focused view of the interface between PRIM2$_{NTD}$ and the MCM3 N-terminal helical domain (site b). Models overlaid were derived from reconstructions on fork DNA with either a 15 nt or 60 nt 5′-flap, in addition to models derived from these respective datasets where DNA engagement was not observed. Models were aligned to the MCM3 N-terminal helical domain**.**

**(B)** Focused view of the interface between PRIM2$_{Nterm}$ and PSF2. Models overlaid were derived from reconstructions on fork DNA with either a 15 nt or 60 nt 5′-flap, in addition to models derived from these respective datasets where DNA engagement was not observed. Models were aligned on PSF2.

**(C)** Focused view of the interface between POLA2$_{96-114}$ and GINS subunits PSF1 and SLD5. Models overlaid were derived from reconstructions on fork DNA with either a 15 nt or 60 nt 5′-flap, in addition to models derived from these respective datasets where DNA engagement was not observed. Models were aligned on PSF1.

**(D)** Detailed view of the atomic model for the interface between the PRIM2$_{NTD}$ (green) and the MCM3 N-terminal helical domain (cyan) (site b). Selected residue sidechains positioned to form inter-protein contacts are labelled.

Residue sidechains are displayed as truncated stubs as the corresponding cryo-EM density is of insufficient resolution to determine their conformation.

**(E)** Pol α-primase associated replisome model docked into a cryo-EM map in which continuous low-resolution density is visualised between the most C-terminal modelled residue of the $PRIM2_{Nterm}$ (G5) and the next modelled residue (Q17). The connecting density was manually coloured green using ChimeraX.

**(F)** Alternative views of the atomic model for the interface between the POLA2 N-terminal helix (residues 96-114) (green) and GINS subunits PSF1, PSF3 and SLD5 (brown). Residue sidechains positioned to form inter-protein contacts are labelled. Residue sidechains are only displayed when the corresponding cryo-EM density is of sufficiently high-resolution to determine their conformation, otherwise sidechains are displayed as truncated stubs.

**(G)** Overlay of cryo-EM density for reconstructions of budding yeast replisomes containing (transparent grey, this study) and lacking (black mesh, EMD-10227) Pol α-primase[S6]. The associated atomic model is docked into the density (yellow). The approximate position of the human POLA2 helix (residues 96-114) bound to PSF1 and SLD5 is overlaid in green and unmodelled density in the vicinity in yeast reconstructions is circled in red.

**(H)** Model for AND-1 in complex with three copies of the POLA1 AND-1 interaction motif (residues 151-171), docked into a locally refined reconstruction.

**(I)** Focused views of the cryo-EM density for the helical bundle of each AND-1 monomer bound by POLA1 (transparent grey) overlaid with cryo-EM density for a reconstruction of the human replisome in the absence of Pol α-primase (EMDB-13376)[S9] (black mesh). An AlphaFold-Multimer model for the interface between $POLA1_{151-171}$ and the helical bundle of AND-1 was aligned to each AND-1 monomer and the fit optimised using rigid-body docking into the cryo-EM map.

**(J)** Coomassie-stained SDS-PAGE gel of purified wild-type human Pol α-primase (lane 1) and mutant Pol α-primase (lane 2) containing a truncation of residues 2-7 in PRIM2 designed to disrupt the interface with PSF2.

**(K)** Coomassie-stained SDS-PAGE gel of purified human CMG (lane 1) and a mutant CMG complex (lane 2) in which four conserved residues on helix α1 of MCM3 were mutated to alanine (MCM3-4A: E14A, R17A, D21A and D24A) to disrupt the interface with PRIM2.

**(L)** Denaturing agarose gel analysis of a primase-polymerase assay performed as illustrated in Figure S5B for 20 min with the indicated proteins.

**(M)** *In vitro* DNA replication reaction with purified human proteins performed as in Figure 6F and 6G.

**SUPPLEMENTARY TABLES (Tables S1-S3)**

| Plasmid name | Proteins expressed | Plasmid details |
|---|---|---|
| vVA52 | *S. cerevisiae* Mcm2, Mcm3-CR | pRS306-CBP-TEV-Mcm3$^{D23R, R26E, E30R, D33R}$-Gal1-10-Mcm2 |
| vVA58 | *S. cerevisiae* Pol1-4A, Pol12 | pRS304-Pol1$^{D141A, D142A, L144A, F147A}$-Gal1-10-Pol12 |
| vJY186 | *S. cerevisiae* Pol1, Pol12-ΔN | pRS304-Pol1 -Gal1-10-Pol12$^{\Delta 2\text{-}81}$ |
| vJY187 | *S. cerevisiae* Pol1-4A, Pol12-ΔN | pRS304-Pol1$^{D141A, D142A, L144A, F147A}$-Gal1-10-Pol12$^{\Delta 2\text{-}81}$ |
| vJY196 | *S. cerevisiae* Pri1, Pri2-Δ2-8 | pRS306-CBP-TEV-Pri1-Gal1-10-Pri2$^{\Delta 2\text{-}8}$ |
| vJY183 | *S. cerevisiae* Pri1, Pri2-5A | pRS306-CBP-TEV-Pri1-Gal1-10-Pri2$^{N210A, E211A, E212A, H213A, Q214A}$ |
| vJY199 | *S. cerevisiae* Pri1, Pri2-AAA | pRS306-CBP-TEV-Pri1-Gal1-10-Pri2$^{F2A, R3A, Q4A}$ |
| vMJ9 | *H. sapiens* PRIM2-Δ2-7 | pACEBac1-PRIM2$^{\Delta 2\text{-}7}$ |
| YB_X2 | *H. sapiens* MCM2, MCM5, MCM3 | pBIG1a-MCM2, MCM5, MCM3 |
| vVA62 | *H. sapiens* MCM2, MCM5, MCM3-4A | pBIG1a-MCM2, MCM5, MCM3$^{E14A, R17A, D21A, D24A}$ |
| YB_X3 | *H. sapiens* MCM4, MCM6, MCM7 | pBIG1b-MCM7, MCM4, MCM6 |
| vJY177 | N/A | Yeast Mcm3 ORF + 935 bp upstream DNA cloned into pFA6-URA at SalI (5′) and AscI (3′) restriction sites. Mcm3-CR mutations were introduced by site-directed mutagenesis. The sequence of the Mcm3 ORF was confirmed by Sanger sequencing. |
| vJY206 | N/A | Yeast Pri2 ORF +705 bp upstream and 156 bp downstream DNA cloned into pFA6-URA at SalI (5′) and AscI (3′) restriction sites. Pri2-AAA mutations were introduced by site-directed mutagenesis. The sequence of the Pri2 ORF was confirmed by Sanger sequencing. |

**Table S1. Plasmids constructed for this study, related to METHODS.**

| Strain Name | Protein expressed | Genotype |
| --- | --- | --- |
| yVA87 | Cdt1-Mcm2-7 (Mcm3-CR) | *MATa ade2-1 ura3-1 his3-11,15 trp1-1 leu2-3,112 can1-100*<br>*bar1::Hyg*<br>*pep4::KanMX*<br>*his3::HIS3pRS303-Cdt1-Gal1-10-Gal4*<br>*trp1::TRP1pRS304-Mcm4-Gal1-10-Mcm5*<br>*leu2::LEU2pRS305-Mcm6-Gal1-10-Mcm7*<br>*ura3::URA3pRS306-CBP-TEV-Mcm3-CR (vVA52)-Gal- Mcm2* |
| yJY239 | Pol α-primase (Pol12-ΔN) | *MATa ade2-1 ura3-1 his3-11,15 trp1-1 leu2-3,112 can1-100*<br>*bar1::Hyg*<br>*pep4::KanMX*<br>*trp1::TRP1-pRS304-Pol1- Gal1-10-Pol12-ΔN (vJY186)*<br>*ura3::URA3pRS306-CBP-TEV-Pri1-Gal1-10-Pri2*<br>*pol1-3XFLAG (NatNT2)* |
| yJY381 | Pol α-primase (Pol1-4A•Pol12-ΔN) | *MATa ade2-1 ura3-1 his3-11,15 trp1-1 leu2-3,112 can1-100*<br>*bar1::Hyg*<br>*pep4::KanMX*<br>*trp1::TRP1pRS304-Pol1-4A-Gal1-10-Pol12-ΔN (vJY187)*<br>*ura3::URA3pRS306-CBP-TEV-Pri1-Gal1-10-Pri2*<br>*pol1-3XFLAG (NatNT2)* |
| yVA96 | Pol α-primase (Pol1-4A) | *MATa ade2-1 ura3-1 his3-11,15 trp1-1 leu2-3,112 can1-100*<br>*bar1::Hyg*<br>*pep4::KanMX*<br>*ura::URA3pRS306-CBP-TEV-Pri1-Gal1-10-Pri2*<br>*trp::TRP1pRS304-Pol1-4A-Gal1-10-Pol12 (vVA58)*<br>*pol1-3XFLAG (NatNT2)* |
| yJY232 | Pol α-primase (Pri2-5A) | *MATa ade2-1 ura3-1 his3-11,15 trp1-1 leu2-3,112 can1-100*<br>*bar1::Hyg*<br>*pep4::KanMX*<br>*trp1::TRP1-pRS304-Pol1-Gal1-10-Pol12*<br>*ura3::URA3pRS306-CBP-TEV-Pri1-Gal1-10-Pri2-5A (vJY183)* |
| yJY241 | Pol α-primase (Pri2-Δ2-8) | *MATa ade2-1 ura3-1 his3-11,15 trp1-1 leu2-3,112 can1-100*<br>*bar1::Hyg*<br>*pep4::KanMX*<br>*trp1::TRP1pRS304-Pol1-Gal1-10-Pol12*<br>*ura3::URA3pRS306-CBP-TEV-Pri1-Gal1-10-Pri2Δ2-8 (vJY196)* |
| yJY242 | Pol α-primase (Pri2-AAA) | *MATa ade2-1 ura3-1 his3-11,15 trp1-1 leu2-3,112 can1-100*<br>*bar1::Hyg*<br>*pep4::KanMX* |

| | | |
|---|---|---|
| | | *trp1::TRP1pRS304-Pol1-Gal1-10-Pol12*<br>*ura3::URA3pRS306-CBP-TEV-Pri1-Gal1-10-Pri2-AAA (vJY199)* |
| yMJ12 | Pol α-primase (Pol1-4A•Pri2-Δ2-8) | *MATa ade2-1 ura3-1 his3-11,15 trp1-1 leu2-3,112 can1-100*<br>*bar1::Hyg*<br>*pep4::KanMX*<br>*trp1::TRP1pRS304-Pol1-4A-Gal1-10-Pol12 (vVA58)*<br>*ura3::URA3pRS306-CBP-TEV-Pri1-Gal1-10-Pri2-Δ2-8 (vJY196)* |
| yMJ13 | Pol α-primase (Pol1-4A•Pol12-ΔN•Pri2-Δ2-8) | *MATa ade2-1 ura3-1 his3-11,15 trp1-1 leu2-3,112 can1-100*<br>*bar1::Hyg*<br>*pep4::KanMX*<br>*trp1::TRP1pRS304-Pol1-4A-Gal1-10-Pol12-ΔN (vJY187)*<br>*ura3::URA3pRS306-CBP-TEV-Pri1-Gal1-10-Pri2-Δ2-8 (vJY196)*<br>*pol1-3XFLAG (NatNT2)* |
| yMJ18 | Pol α-primase (Pol12-ΔN•Pri2-Δ2-8) | *MATa ade2-1 ura3-1 his3-11,15 trp1-1 leu2-3,112 can1-100*<br>*bar1::Hyg*<br>*pep4::KanMX*<br>*trp1::TRP1pRS304-Pol1-Gal1-10-Pol12-ΔN (vJY186)*<br>*ura3::URA3pRS306-CBP-TEV-Pri1-Gal1-10-Pri2-Δ2-8 (vJY196)*<br>*pol1-3XFLAG (NatNT2)* |

**Table S2.** ***S. cerevisiae*** **protein expression strains constructed for this study, related to METHODS.**

| Protein | Expression strain / plasmid | Affinity tag | Purification steps | Reference where Purification method described |
|---|---|---|---|---|
| Cdc45 | yJY13 | Internal 2xFLAG tag | Anti-FLAG M2 Agarose<br>Bio-Gel HT Hydroxyapatite | [S10] |
| Cdc6 | Plasmid pAM3 (*E. coli* expression) | N-terminal GST cleavable tag | Glutathione Sepharose 4B<br>Bio-Gel HT Hydroxyapatite | [S11] |
| Cdt1-Mcm2-7 | yAM33 | N-terminal CBP cleavable tag on Mcm3 | Calmodulin-Sepharose 4B<br>Superdex 200 | [S11] |
| Tof1-Csm3 | yAE48 | N-terminal CBP cleavable tag on Csm3 | Calmodulin-Sepharose 4B<br>MonoQ<br>Superdex 200 | [S6,12] |
| Ctf4 | yAE40 | N-terminal CBP tag | Calmodulin-Sepharose 4B<br>MonoQ<br>Superdex 200 | [S10] |
| DDK | ySDK8 | CBP tag on Dbf4 | Calmodulin-Sepharose 4B<br>Lambda phosphatase dephosphorylation<br>Superdex 200 | [S13] |
| Dpb11 | yJY26 | C-terminal 3xFLAG tag | Anti-FLAG M2 Agarose<br>MonoS | [S10] |
| GINS | Plasmid pJFDJ5 (*E. coli* expression) | N-terminal His tag on Psf3 | Ni-NTA Agarose<br>MonoQ<br>Superdex 200 | [S10] |
| Mcm10 | pET28a-Mcm10 (*E. coli* expression) | N-terminal His tag | Ni-NTA Agarose<br>MonoS (twice) | [S10] |
| Mrc1 | yJY32 | C-terminal 2xFLAG tag | Anti-FLAG M2 Agarose<br>Superose 6 | [S6,12] |
| ORC | ySD-ORC | CBP-cleavable tag on Orc1 | Calmodulin-Sepharose 4B<br>Superdex 200 | [S14] |
| Yeast PCNA | vJY19 (*E. coli* expression) | Untagged | Nucleic acid precipitation with Polymin P<br>Ammonium sulfate precipitation<br>HiTrap SP HP (flow through)<br>HiTrap Heparin HP (flow through)<br>HiTrap DEAE Fast Flow<br>MonoQ | [S12] |
| Yeast Pol α-primase | yAE95 | N-terminal CBP tag on Pri1 | Calmodulin-Sepharose 4B<br>MonoQ<br>Superdex 200 | [S12] |
| Yeast Pol δ | yAE34 | C-terminal CBP tag on Pol32 | Calmodulin-Sepharose 4B<br>HiTrap Heparin HP<br>Superdex 200 | [S12] |
| Yeast Pol ε | yAJ2 | C-terminal CBP tag on Dpb4 | Calmodulin-Sepharose 4B<br>HiTrap Heparin HP<br>Superdex 200 | [S10] |
| Yeast RFC | yAE41 | N-terminal CBP tag on Rfc3 | Calmodulin-Sepharose 4B<br>MonoS<br>Superdex 200 | [S12] |
| Yeast RPA | yJY106 | Untagged | Nucleic acid precipitation with Polymin P<br>Ammonium sulfate precipitation<br>HiTrap Blue HP<br>ssDNA Cellulose<br>MonoQ | [S6] |
| Sld2 | yTD8 | C-terminal 3x FLAG | Ammonium sulfate precipitation<br>Anti-FLAG M2 Agarose<br>HiTrap SP HP | [S10] |
| Sld3/7 | yTD6 | C-terminal cleavable TCP tag | IgG Sepharose Fast Flow<br>TEV removal with Ni-NTA Agarose<br>Superdex 200 | [S10] |
| Yeast CMG | yJY197 | Internal 2xFLAG tag on Cdc45<br>N-terminal CBP cleavable tag on Mcm3<br>N-terminal His tag on Psf3 | Anti-FLAG M2 Agarose<br>Calmodulin-Sepharose 4B<br>MonoQ | [S6,15] |
| TIMELESS-TIPIN | MT_DF1, MT_BD1 | N-terminal Twin-Strep TEV on TIMELESS | Strep-Tactin XT superflow high capacity<br>HiTrap Q HP | [S9] and this study |

| | | | | |
|---|---|---|---|---|
| | | | Superdex 200i | |
| AND-1 | MT_BF1 | N-terminal 3X FLAG TEV | Anti-FLAG M2 Agarose<br>MonoQ<br>Superdex 200i | [S9] |
| AND-1-Δ1017 | YB_8 | N-terminal 3X FLAG TEV | Anti-FLAG M2 Agarose<br>MonoQ<br>Superdex 200i | [S16] |
| Human Pol α-primase | MT_BC3, MT_AE1, MT_AF1, MT_AG1 | N-terminal Twin-Strep 3C on POLA1 | Strep-Tactin XT superflow high capacity<br>MonoQ<br>Superdex 200i | [S16] |
| CLASPIN | MT_DB1 | C-terminal HRV 3C 3X FLAG | Anti-FLAG M2 Agarose<br>Superose 6 | [S9] |
| Human PCNA | MT_EB1 | Untagged | Nucleic acid precipitation with Polymin P<br>Ammonium sulfate precipitation<br>Tandem HiTrap SP FF, HiTrap heparin<br>DEAE column<br>MonoQ<br>Superdex 200i | [S9] |
| Human Pol δ | MT_CF1, MT_CH1, YB_3, MT_FC1 | N-terminal Twin-Strep-TEV on POLD4 | Strep-Tactin XT superflow high capacity<br>MonoQ<br>Superdex 200i | [S16] |
| Human Pol ε | MT_U2, MT_L1, MT_M1, MT_N1 | C-terminal TEV CBP on POLE3 (not used for purification) | Nucleic acid precipitation with Polymin P<br>Ammonium sulfate precipitation<br>HiTrap SP FF<br>HiTrap Q FF<br>HiTrap heparin<br>MonoQ<br>Superdex 200i | [S16] |
| Human RFC | MT_BH1, MT_BJ1, MT_BK1, MT_BL1, MT_BI1 | | Strep-Tactin XT superflow high capacity<br>HiTrap Heparin<br>MonoQ | [S9] |
| Human RPA | YB_X1 | Untagged | HiTrap Blue<br>Bio-Gel HT hydroxyapatite<br>MonoQ | [S9] |
| CTF18-RFC | YB_7, YB_5, YB_6, YB_4 | N-terminal Twin-Strep-TEV on CTF18 | Strep-Tactin XT superflow high capacity<br>MonoQ<br>Superdex 200i | [S16] |
| Human CMG | YB_2, YB_1, MT_01 | N-terminal Twin-Strep HRV 3C on SLD5, Internal FLAG in CDC45 | Anti-FLAG M2 Agarose<br>Strep-Tactin XT superflow high capacity<br>MonoQ | [S9] |

**Table S3. Details of protein purification strategy, related to METHODS.**

| Strain name | Genotype |
|---|---|
| yJY244 | *MATa ade2-1 ura3-1 his3-11,15 trp1-1 leu2-3,112 can1-100 / MATα ade2-1 ura3-1 his3-11,15 trp1-1 leu2-3,112 can1-100*<br>*PRI2 / PRI2 (URA3)* |
| yJY297 | *MATa ade2-1 ura3-1 his3-11,15 trp1-1 leu2-3,112 can1-100 / MATα ade2-1 ura3-1 his3-11,15 trp1-1 leu2-3,112 can1-100*<br>*MCM3 / MCM3 (Ura3)* |
| yJY321 | MATa ade2-1 ura3-1 his3-11,15 trp1-1 leu2-3,112 can1-100 / MATα ade2-1 ura3-1 his3-11,15 trp1-1 leu2-3,112 can1-100<br>*PRI2 / pri2-AAA (URA3)* |
| yJY300 | *MATa ade2-1 ura3-1 his3-11,15 trp1-1 leu2-3,112 can1-100 / MATα ade2-1 ura3-1 his3-11,15 trp1-1 leu2-3,112 can1-100*<br>*MCM3 / mcm3-CR (Ura3)* |
| yJY301 | *MATa ade2-1 ura3-1 his3-11,15 trp1-1 leu2-3,112 can1-100 / MATα ade2-1 ura3-1 his3-11,15 trp1-1 leu2-3,112 can1-100*<br>*MCM3 / mcm3-CR (Ura3)* |
| yJY365 | *MATa ade2-1 ura3-1 his3-11,15 trp1-1 leu2-3,112 can1-100 / MATα ade2-1 ura3-1 his3-11,15 trp1-1 leu2-3,112 can1-100*<br>*MCM3 / mcm3-CR (Ura3)*<br>*PRI2 / PRI2 (URA3)* |
| yJY367 | *MATa ade2-1 ura3-1 his3-11,15 trp1-1 leu2-3,112 can1-100 / MATα ade2-1 ura3-1 his3-11,15 trp1-1 leu2-3,112 can1-100*<br>*MCM3 / MCM3 (Ura3)*<br>*PRI2 / pri2-AAA (URA3)* |
| yJY345 | *MATa ade2-1 ura3-1 his3-11,15 trp1-1 leu2-3,112 can1-100 / MATα ade2-1 ura3-1 his3-11,15 trp1-1 leu2-3,112 can1-100*<br>*MCM3 / mcm3-CR (Ura3)*<br>*PRI2 / pri2-AAA (URA3)* |
| yJY350 | *MATa ade2-1 ura3-1 his3-11,15 trp1-1 leu2-3,112 can1-100 / MATα ade2-1 ura3-1 his3-11,15 trp1-1 leu2-3,112 can1-100*<br>*MCM3 / mcm3-CR (Ura3)*<br>*SML1 / sml1Δ::HIS3* |
| yJY351 | *MATa ade2-1 ura3-1 his3-11,15 trp1-1 leu2-3,112 can1-100 / MATα ade2-1 ura3-1 his3-11,15 trp1-1 leu2-3,112 can1-100*<br>*PRI2 / pri2-AAA (URA3)*<br>*SML1 / sml1Δ::HIS3* |
| yJY356 | *MATa ade2-1 ura3-1 his3-11,15 trp1-1 leu2-3,112 can1-100 / MATα ade2-1 ura3-1 his3-11,15 trp1-1 leu2-3,112 can1-100*<br>*MCM3 / mcm3-CR (Ura3)* |

| | |
|---|---|
| | *sml1Δ::HIS3 / sml1Δ::HIS3*<br>*MEC1 / mec1Δ::ADE2* |
| yJY357 | *MATa ade2-1 ura3-1 his3-11,15 trp1-1 leu2-3,112 can1-100 / MATα ade2-1 ura3-1 his3-11,15 trp1-1 leu2-3,112 can1-100*<br>*PRI2 / pri2-AAA (URA3)*<br>*sml1Δ::HIS3 / sml1Δ::HIS3*<br>*MEC1 / mec1Δ::ADE2* |
| yJY255 | *MATa ade2-1 ura3-1 his3-11,15 trp1-1 leu2-3,112 can1-100*<br>*PRI2 (URA3)* |
| yJY302 | *MATa ade2-1 ura3-1 his3-11,15 trp1-1 leu2-3,112 can1-100*<br>*MCM3 (Ura3)* |
| yJY326 | *MATa ade2-1 ura3-1 his3-11,15 trp1-1 leu2-3,112 can1-100*<br>*pri2-AAA (URA3)* |
| yJY328 | *MATα ade2-1 ura3-1 his3-11,15 trp1-1 leu2-3,112 can1-100*<br>*pri2-AAA (URA3)* |
| yJY313 | *MATa ade2-1 ura3-1 his3-11,15 trp1-1 leu2-3,112 can1-100*<br>*mcm3-CR (Ura3)* |
| yJY315 | *MATα ade2-1 ura3-1 his3-11,15 trp1-1 leu2-3,112 can1-100*<br>*mcm3-CR (Ura3)* |
| yJY317 | *MATa ade2-1 ura3-1 his3-11,15 trp1-1 leu2-3,112 can1-100*<br>*mcm3-CR (Ura3)* |
| yJY352 | *MATa ade2-1 ura3-1 his3-11,15 trp1-1 leu2-3,112 can1-100*<br>*mcm3-CR (Ura3)*<br>*sml1Δ::HIS3* |
| yJY354 | *MATa ade2-1 ura3-1 his3-11,15 trp1-1 leu2-3,112 can1-100*<br>*pri2-AAA (URA3)*<br>*sml1Δ::HIS3* |

**Table S4. *S. cerevisiae* strains constructed in this study for genetics experiments. All strains are based on the W303 background, related to METHODS.**

## Supplemental References


S1. Kucukelbir, A., Sigworth, F.J., and Tagare, H.D. (2014). Quantifying the local resolution of cryo-EM density maps. Nat Methods *11*, 63-65. 10.1038/nmeth.2727.

S2. Baranovskiy, A.G., Babayeva, N.D., Zhang, Y., Gu, J., Suwa, Y., Pavlov, Y.I., and Tahirov, T.H. (2016). Mechanism of Concerted RNA-DNA Primer Synthesis by the Human Primosome*. Journal of Biological Chemistry *291*, 10006-10020. https://doi.org/10.1074/jbc.M116.717405.

S3. Jumper, J., Evans, R., Pritzel, A., Green, T., Figurnov, M., Ronneberger, O., Tunyasuvunakool, K., Bates, R., Žídek, A., Potapenko, A., et al. (2021). Highly accurate protein structure prediction with AlphaFold. Nature. 10.1038/s41586-021-03819-2.

S4. Perera, R.L., Torella, R., Klinge, S., Kilkenny, M.L., Maman, J.D., and Pellegrini, L. (2013). Mechanism for priming DNA synthesis by yeast DNA polymerase alpha. Elife *2*, e00482. 10.7554/eLife.00482.

S5. He, Q., Lin, X., Chavez, B.L., Agrawal, S., Lusk, B.L., and Lim, C.J. (2022). Structures of the human CST-Polα–primase complex bound to telomere templates. Nature *608*, 826-832. 10.1038/s41586-022-05040-1.

S6. Baretić, D., Jenkyn-Bedford, M., Aria, V., Cannone, G., Skehel, M., and Yeeles, J.T.P. (2020). Cryo-EM Structure of the Fork Protection Complex Bound to CMG at a Replication Fork. Molecular Cell *78*, 926-940.e913. https://doi.org/10.1016/j.molcel.2020.04.012.

S7. Simon, A.C., Zhou, J.C., Perera, R.L., van Deursen, F., Evrin, C., Ivanova, M.E., Kilkenny, M.L., Renault, L., Kjaer, S., Matak-Vinkovic, D., et al. (2014). A Ctf4 trimer couples the CMG helicase to DNA polymerase alpha in the eukaryotic replisome. Nature *510*, 293-297. 10.1038/nature13234.

S8. Bellelli, R., and Boulton, S.J. (2021). Spotlight on the Replisome: Aetiology of DNA Replication-Associated Genetic Diseases. Trends Genet *37*, 317-336. 10.1016/j.tig.2020.09.008.

S9. Jones, M.L., Baris, Y., Taylor, M.R.G., and Yeeles, J.T.P. (2021). Structure of a human replisome shows the organisation and interactions of a DNA replication machine. The EMBO Journal *n/a*, e108819. https://doi.org/10.15252/embj.2021108819.

S10. Yeeles, J.T., Deegan, T.D., Janska, A., Early, A., and Diffley, J.F. (2015). Regulated eukaryotic DNA replication origin firing with purified proteins. Nature *519*, 431-435. 10.1038/nature14285.

S11. Coster, G., Frigola, J., Beuron, F., Morris, E.P., and Diffley, J.F. (2014). Origin licensing requires ATP binding and hydrolysis by the MCM replicative helicase. Mol Cell *55*, 666-677. 10.1016/j.molcel.2014.06.034.

S12. Yeeles, J.T.P., Janska, A., Early, A., and Diffley, J.F.X. (2017). How the Eukaryotic Replisome Achieves Rapid and Efficient DNA Replication. Mol Cell *65*, 105-116. 10.1016/j.molcel.2016.11.017.

S13. On, K.F., Beuron, F., Frith, D., Snijders, A.P., Morris, E.P., and Diffley, J.F. (2014). Prereplicative complexes assembled in vitro support origin-dependent and independent DNA replication. EMBO J *33*, 605-620. 10.1002/embj.201387369.

S14. Frigola, J., Remus, D., Mehanna, A., and Diffley, J.F. (2013). ATPase-dependent quality control of DNA replication origin licensing. Nature *495*, 339-343. 10.1038/nature11920.

S15. Jenkyn-Bedford, M., Jones, M.L., Baris, Y., Labib, K.P.M., Cannone, G., Yeeles, J.T.P., and Deegan, T.D. (2021). A Conserved Mechanism for Regulating Replisome Disassembly in Eukaryotes. Nature. 10.1038/s41586-021-04145-3.

S16. Baris, Y., Taylor, M.R.G., Aria, V., and Yeeles, J.T.P. (2022). Fast and efficient DNA replication with purified human proteins. Nature *606*, 204-210. 10.1038/s41586-022-04759-1.
